# Genetic Markers of Adaptation of Plasmodium falciparum to Transmission by American Vectors Identified in the Genomes of Parasites from Haiti and South America

**DOI:** 10.1128/mSphere.00937-20

**Published:** 2020-10-21

**Authors:** Massimiliano S. Tagliamonte, Charles A. Yowell, Maha A. Elbadry, Jacques Boncy, Christian P. Raccurt, Bernard A. Okech, Erica M. Goss, Marco Salemi, John B. Dame

**Affiliations:** a Department of Infectious Diseases and Immunology, College of Veterinary Medicine, University of Florida, Gainesville, Florida, USA; b Department of Environmental and Global Health, College of Public Health and Health Professions, University of Florida, Gainesville, Florida, USA; c Laboratoire National de Santé Publique, Ministère de la Santé Publique et de la Population, Port-au-Prince, Haiti; d Department of Tropical Medicine and Infectious Diseases, Faculty of Medicine, University of Quisqueya, Port-au-Prince, Haiti; e Department of Plant Pathology, College of Agricultural and Life Sciences, University of Florida, Gainesville, Florida, USA; f Department of Pathology, Immunology, and Laboratory Medicine, College of Medicine, University of Florida, Gainesville, Florida, USA; g Emerging Pathogens Institute, University of Florida, Gainesville, Florida, USA; University of Copenhagen

**Keywords:** Haiti, *Plasmodium falciparum*, adaptive mutations, evolutionary biology, malaria, phylogenetics, vector-borne diseases

## Abstract

The malaria parasite, Plasmodium falciparum, was introduced into Hispaniola and other regions of the Americas through the slave trade spanning the 16th through the 19th centuries. During this period, more than 12 million Africans were brought across the Atlantic to the Caribbean and other regions of the Americas. Since malaria is holoendemic in West Africa, a substantial percentage of these individuals carried the parasite. St. Domingue on Hispaniola, now modern-day Haiti, was a major port of disembarkation, and malaria is still actively transmitted there. We undertook a detailed study of the phylogenetics of the Haitian parasites and those from Colombia and Peru utilizing whole-genome sequencing. Principal-component and phylogenetic analyses, based upon single nucleotide polymorphisms (SNPs) in protein coding regions, indicate that, despite the potential for millions of introductions from Africa, the Haitian parasites share an ancestral relationship within a well-supported monophyletic clade with parasites from South America, while belonging to a distinct lineage. This result, in stark contrast to the historical record of parasite introductions, is best explained by a severe population bottleneck experienced by the parasites introduced into the Americas. Here, evidence is presented for targeted selection of rare African alleles in genes which are expressed in the mosquito stages of the parasite’s life cycle. These genetic markers support the hypothesis that the severe population bottleneck was caused by the required adaptation of the parasite to transmission by new definitive hosts among the *Anopheles* (*Nyssorhynchus*) spp. found in the Caribbean and South America.

**IMPORTANCE** Historical data suggest that millions of P. falciparum parasite lineages were introduced into the Americas during the trans-Atlantic slave trade, which would suggest a paraphyletic origin of the extant isolates in the Western Hemisphere. Our analyses of whole-genome variants show that the American parasites belong to a well-supported monophyletic clade. We hypothesize that the required adaptation to American vectors created a severe bottleneck, reducing the effective introduction to a few lineages. In support of this hypothesis, we discovered genes expressed in the mosquito stages of the life cycle that have alleles with multiple, high-frequency or fixed, nonsynonymous mutations in the American populations which are rarely found in African isolates. These alleles appear to be in gene products critical for transmission through the anopheline vector. Thus, these results may inform efforts to develop novel transmission-blocking vaccines by identifying parasite proteins functionally interacting with the vector that are important for successful transmission. Further, to the best of our knowledge, these are the first whole-genome data available from Haitian P. falciparum isolates. Defining the genome of these parasites provides genetic markers useful for mapping parasite populations and monitoring parasite movements/introductions.

## Abstract (precis)

Historical data suggest that millions of P. falciparum parasite lineages were introduced into the Americas during the trans-Atlantic slave trade, which would suggest a paraphyletic origin of the extant isolates in the Western Hemisphere. Our analyses of whole-genome variants show that the American parasites belong to a well-supported monophyletic clade. We hypothesize that the required adaptation to American vectors created a severe bottleneck, reducing the effective introduction to a few lineages. In support of this hypothesis, we discovered genes expressed in the mosquito stages of the life cycle that have alleles with multiple, high-frequency or fixed, nonsynonymous mutations in the American populations which are rarely found in African isolates. These alleles appear to be in gene products critical for transmission through the anopheline vector. Thus, these results may inform efforts to develop novel transmission-blocking vaccines by identifying parasite proteins functionally interacting with the vector that are important for successful transmission. Further, to the best of our knowledge, these are the first whole-genome data available from Haitian P. falciparum isolates. Defining the genome of these parasites provides genetic markers useful for mapping parasite populations and monitoring parasite movements/introductions.

## INTRODUCTION

Accumulated evidence suggests that prior to the colonization of the Americas by nations of Western Europe, malaria caused by Plasmodium falciparum was not a disease in the Americas (1). P. falciparum infection is holoendemic in West Africa; thus, a large, diverse population of this parasite was introduced into the Americas via the trans-Atlantic slave trade; records describe the disembarkation of more than 12 million African slaves in the Americas during the period of 1500 to 1875 (2, 3). African mosquitos [*Anopheles* (*Cellia*) spp.], to which the parasite was highly adapted for efficient transmission, did not become established in the Americas (4); thus, transmission was sustained in the diverse geographical regions of the Western Hemisphere via native *Anopheles* (*Nyssorhynchus*) spp. Nearly half of the African slaves (>5.5 million) disembarked in the Caribbean islands, and more than 1 million of those disembarked on the island of Hispaniola, where ∼900,000 arrived between 1700 and 1800 (2). Approximately 5.8 million slaves disembarked in diverse ports in South America, where currently P. falciparum is actively transmitted in the 10 countries surrounding the Amazon River basin (5).

Vector control and better access to medical care in the 20th century, plus improvements to economic and housing conditions, have eliminated malaria from all Caribbean islands except Hispaniola, which encompasses Haiti and the Dominican Republic. In Haiti, this disease is endemic and becomes epidemic in the rainy seasons (6). Malaria is also transmitted in the Dominican Republic, with the highest risk in the western part of the country, near the Haitian border, where many cases are considered imported malaria, resulting in local transmission (7). Haiti is considered a low-transmission area, although it has foci with higher risk (8, 9). The Haiti Malaria Elimination Consortium (HaMEC), led by the CDC, is aiming to eliminate malaria from Hispaniola by 2022, but there are major obstacles to reaching this goal. Several studies have highlighted the high prevalence of asymptomatic infections, and their contribution to transmission is yet to be properly estimated (10–12). The malarious countries in South America have a higher incidence of malaria, and among them only Paraguay is in a pre-elimination phase (5).

Recent studies have investigated the Haitian P. falciparum population structure using microsatellite markers (13) or a limited number of single nucleotide polymorphisms (SNPs) (14) and found low diversity and evidence of focal transmission. Isolation of the Haitian parasite population from other populations in the Americas was suggested (14) using data from a limited number of SNP markers.

In the present study, we analyzed the phylogenetic relationship of the Haitian P. falciparum population to those from South America and other continents, utilizing data from whole-genome sequencing. The P. falciparum reference genome was made available in 2002 (15), providing a template for mapping the genomic sequences of field isolates for comparative studies. The analyses reported here were performed using newly obtained whole-genome sequence data from 21 Haitian isolates plus genome sequence data previously obtained for 16 Colombian and 11 Peruvian isolates.

The much larger number of informative characters available from whole-genome sequence data enhances the analytic power of the tools of population genetics, compared to microsatellites or a limited number of SNPs (14, 16), particularly for organisms, such as P. falciparum, which are characterized by low intraspecies diversity. The large sample of SNPs available from genomic data was used to perform a higher-resolution analysis of the ancestral relationship of the Haitian isolates to others from South America and Africa. In contrast to historical data, our results with data from Haitian and South American isolates describe an ancestral relationship within a well-supported monophyletic clade with parasites from Africa. Since a severe population bottleneck experienced by the parasites was likely responsible for the results, the hypothesis was tested that the American vectors, having evolved in isolation for ∼100 million years separately from those hosting P. falciparum in Africa (17), have exerted powerful selective pressures on the parasite. Thus, the data set was examined for the targeted selection of rare African alleles in genes expressed in the mosquito stages of the parasite’s life cycle.

## RESULTS

**FIG 1:**
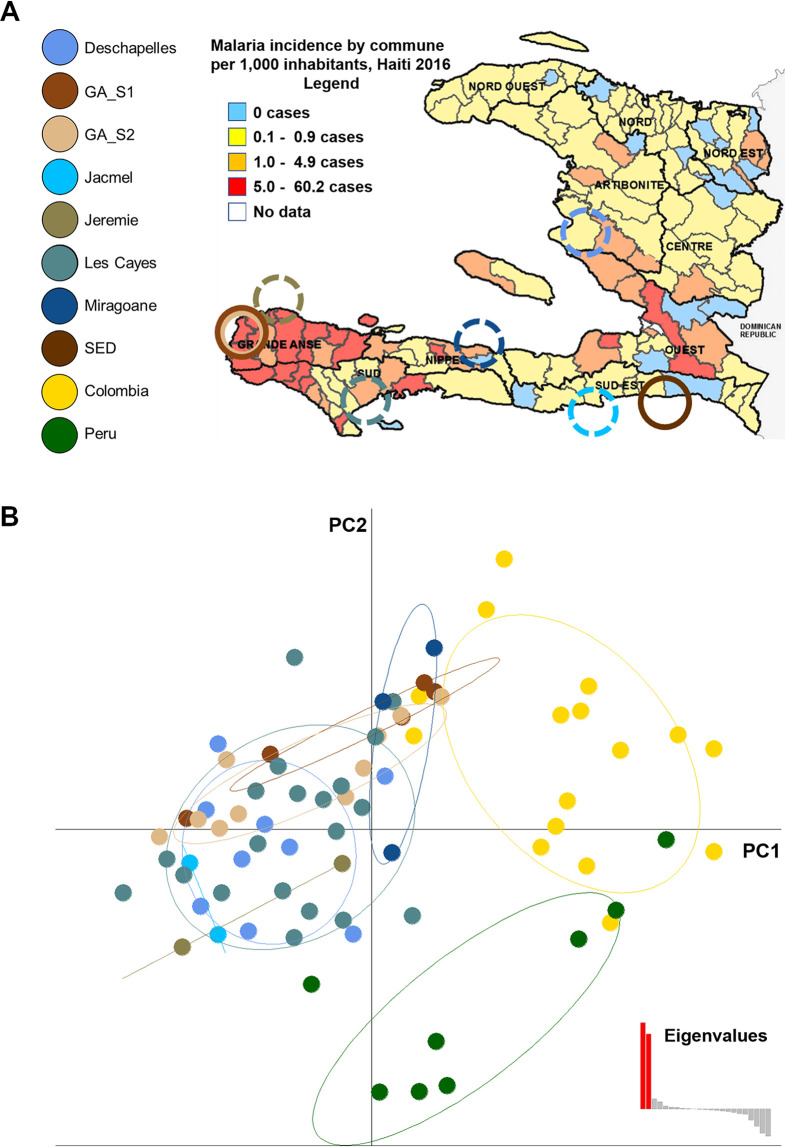
Clustering analyses on 24 SNP markers. (A) Map of Haiti and sampling sites. Incidence data are from reference 74. Dotted lines indicate origins of samples from reference 14; solid lines indicate origins of our samples, for which whole-genome data are available. (B) sPCA using 24 SNP barcodes from reference 14; following original paper filtering protocol for consistency, one of the isolates that were filtered out was our sample from the Sud-Est Department.

Twenty-two P. falciparum isolates were collected from active malaria cases, 21 of which came from the Department of Grand’Anse, with one from the Sud-Est (South-East) Department (Fig. 1A). Parasite DNA isolated from the patients’ blood samples was utilized for nearly all analyses, but four of the isolates from Grand’Anse were also adapted to long-term cultures. The primary samples for this study were thus obtained from the region of Haiti where clinical malaria is prevalent and where sufficient genetic material could be obtained for whole-genome sequencing.

### Whole-genome sequencing.

Parasite genomic DNA was recovered from 22 of the blood samples, and each was amplified to obtain sufficient DNA for Illumina library preparation for sequencing. Amplified material from six isolates was evaluated for possible uneven whole-genome amplification by quantitative PCR (qPCR) of six single-copy marker genes with varying A+T content and genomic locations (AMA1, CRT, GEX06, GST, MRP1, and SOAP). There was a 4- to 5-fold variation in the concentrations of these marker genes (data not shown), reflecting a known problem in obtaining uniform amplification of the P. falciparum genome (18). Since the concentrations of these different single-copy genes were on the same order of magnitude, the amplified DNA was deemed acceptable for genome sequencing, and a segment of the AMA1 gene was used for estimating by qPCR P. falciparum DNA content in all amplified samples (Text S1).

10.1128/mSphere.00937-20.5 TEXT S1 Supplemental methods. Download Text S1, DOCX file, 0.04 MB. Copyright © 2020 Tagliamonte et al. 2020 Tagliamonte et al. This content is distributed under the terms of the Creative Commons Attribution 4.0 International license.

DNA obtained from parasites in low-passage-number *in vitro* culture was compared with amplified DNA from the primary isolate for two of the isolates. Less than 2% of SNPs (1.4% and 1.8%) differed between the amplified primary genomic DNA and that of the cultured parasites, and in these cases, the differences were homozygous versus heterozygous genotypes. These differences were considered to be minor; thus, in two instances when cultured parasites were available and the primary isolate had a relatively high human mitochondrial DNA (mtDNA) contamination, unamplified genomic DNA from cultured parasites was used for library preparation, sequencing, and variant calling. The final whole-genomic sequence data set consisted of data from 21 Haitian isolates, from which we obtained at least 40× genome coverage (Table S1).

10.1128/mSphere.00937-20.6 TABLE S1 Sequencing and mapping statistics. Samples with at least 40× coverage were used for variant calling, with the reduced set indicated following postclonal analysis. Download Table S1, DOCX file, 0.02 MB. Copyright © 2020 Tagliamonte et al. 2020 Tagliamonte et al. This content is distributed under the terms of the Creative Commons Attribution 4.0 International license.

### SNP marker analysis.

A recent study on Haitian P. falciparum (14) used 24 SNP markers as a barcode to investigate its population structure and its relationship to South American strains. Their spatial principal-component analysis (sPCA) results indicated that Haiti strains mostly cluster independently of the continental strains, with possible gene flow between Colombia and Hispaniola. The 24-SNP analysis was repeated by obtaining these SNPs from whole-genome sequencing data from the Haitian isolates in this study, plus 11 isolates from Peru and 16 from Colombia, retrieved from the MalariaGEN database, compiled by the MalariaGEN Community Project (19, 20) on www.malariagen.net/ and stored on the European Nucleotide Archive (http://www.ebi.ac.uk/ena). To these data were added 40 published barcode sequences from Haitian parasites from the study by Charles et al. (14). For consistency with the original pipeline, isolates missing data from more than 5 markers or containing more than 1 multiallelic site were discarded. This led to dropping two Haitian isolates. Similar to Charles et al. (14), we found that two Colombian isolates clustered with the Haitian population (Fig. 1B), indicating the potential for gene flow between the two areas. These findings are consistent with previous analyses that compared other Haitian departments (13, 14), and together, the results indicate that the parasite population in Haiti is a single unstructured population with focal transmission. Thus, the isolates utilized for genomic SNP analyses appear to be suitably representative of P. falciparum in Haiti.

### Variant calling for population genetic analyses.

Two variant calling iterations were performed, one using the 21 Haitian isolates only (Haitian data set) and one using these data plus whole-genome sequencing data for 149 isolates from numerous malarious regions worldwide retrieved from the MalariaGEN database, for a total of 170 isolates (WW data set). Genome sequence data from a total of 27 samples were available from South America, 16 of which originated from Colombia and 11 from Peru. Based on historical data regarding the trans-Atlantic slave trade (3, 21), we downloaded genome sequence data from isolates recovered in West Africa: Gambia, Ghana, and Cameroon (10 samples from each country). We also downloaded data from 10 samples from Central Africa (Democratic Republic of Congo) and 10 from each Kenya, Malawi, and Tanzania to represent East Africa. Data on 22 genomes from Papua New Guinea were downloaded as representative of Oceania and 10 each from Cambodia, Myanmar, and Thailand to represent Southeast Asia. Data sets chosen were from paired-end Illumina libraries, with a minimum 100-nucleotide read length. The largest data sets from each country were chosen and whenever possible were larger than 2 Gb. An exception to the read length rule was made for South American data sets, due to the scarcity of samples available; thus, these libraries have 54- to 100-nucleotide paired-end reads. Details regarding the downloaded data are in the supplemental material (Table S2).

10.1128/mSphere.00937-20.7 TABLE S2 Data utilized from the MalariaGEN Plasmodium falciparum Community Project. Download Table S2, DOCX file, 0.04 MB. Copyright © 2020 Tagliamonte et al. 2020 Tagliamonte et al. This content is distributed under the terms of the Creative Commons Attribution 4.0 International license.

When variant calling was done on Haitian isolates versus the coding regions of the 3D7 genome, 447,339 variants were obtained. A filtering pipeline based on the one published by Manske et al. (19) was applied, and after filtering, 22,044 variant loci were retained as reliable for further analyses. These were located in 3,189 different genes. The P. falciparum genome is particularly difficult to map; thus, reliably mapped reads come from just 60% of its ∼5,300 annotated genes (15). About 90% (19,901) of the variants were SNPs, while the rest were indels, most of which were in frame. Almost three-quarters of the SNPs (14,148) were nonsynonymous mutations resulting in an amino acid change in the translated gene product. This is a known phenomenon, and it has been attributed to continuous positive selection exerted by the host (22). The complexity of infection was assessed using THE REAL McCOIL (23), and all Haitian isolates represented single infections with comparatively few heterozygous alleles consistent with the low-transmission setting.

For phylogenetic purposes, variant calling was repeated on the WW data set. Variant calling produced almost 1,400,000 variants, which were reduced to about 139,000 variants by the filtering process. In order to determine the ancestral relationship of the Haitian P. falciparum to isolates from Africa, Asia, and South America, sites with indels, conserved mutations (those found in all isolates), and singletons were removed, since they provide no phylogenetic information, leaving 50,469 sites in 3,106 genes representing almost 60% of the genes in the nuclear genome.

### Population genetics using whole-genome SNPs.

**FIG 2:**
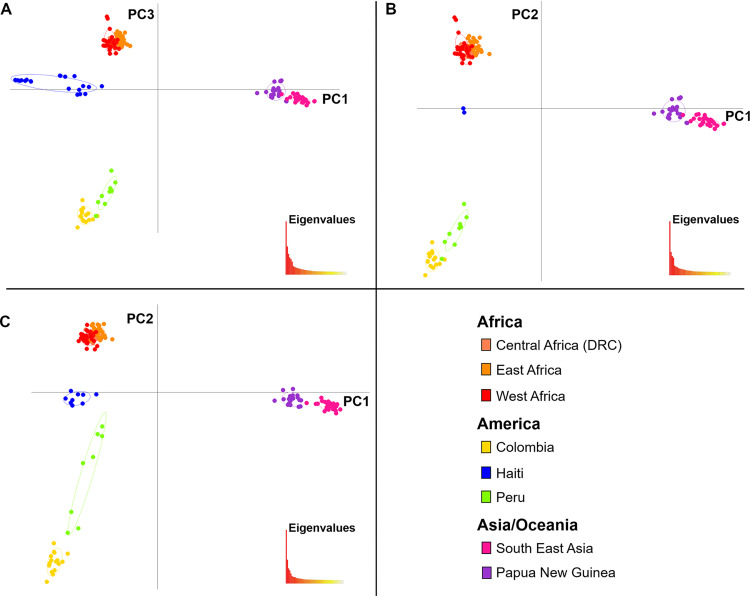
Two-dimensional plot of the principal-component analysis using 50,469 SNPs. (A) PCA performed using all samples. (B) To account for the possibility that covariation among parasites that share recent ancestry has a disproportionate impact on PC weightings, the PCA was repeated keeping only a single representative of the two dominant Haitian lineages. (C) PCA performed using a subset of 149 sequences created by removing samples resulting from apparent clonal (epidemic) expansion identified in Fig. S2 or having complexity of infection.

Principal-component analysis was used to study the clustering of the samples without assuming an evolutionary model. The PCA shows a clear separation between isolates from different continents (Fig. 2). Haitian isolates clustered separately from South American isolates, while the Peruvian and Colombian clusters partially overlapped. The Haitian isolates grouped into two different clusters, one of which consisted of nine virtually identical isolates, evidence of an apparent epidemic expansion (Fig. 2A). Discriminant analysis of the principal components (DAPC) was also performed, which minimizes within-group variance, while maximizing between-group variance (24). The results are similar to those of the PCA, yielding evidence for seven different population clusters (Fig. S1).

10.1128/mSphere.00937-20.1 FIG S1 DAPC plot. The graph shows the values of BIC (Bayesian information criterion) used to choose the number of clusters for P. falciparum populations. The seven clusters identified are listed on the right. There are two Haitian clusters, as also seen in the PCA and the NJ trees, likely due to a recent epidemic expansion. Download FIG S1, TIF file, 0.2 MB. Copyright © 2020 Tagliamonte et al. 2020 Tagliamonte et al. This content is distributed under the terms of the Creative Commons Attribution 4.0 International license.

10.1128/mSphere.00937-20.2 FIG S2 Clonal expansion analyses. (A) Clonal expansion analysis with POPPR. The genetic distance between samples was calculated with three different algorithms. These distances were plotted against the number of distinct samples identified when the distances were used as thresholds. The largest gap in the plateau (0.004 to 0.0205 [vertical red bars]) was used as a cutoff to define unique isolates. (B) Clonal expansion analysis with Isorelate. The minimum proportion of genome shared IBD (identity by descent) between a pair of isolates in order for the pair to be included in the network was set to 0.9. Results did not change when the threshold was increased to 0.99. The isolates identified by this analysis matched the ones identified by POPPR. Download FIG S2, TIF file, 0.3 MB. Copyright © 2020 Tagliamonte et al. 2020 Tagliamonte et al. This content is distributed under the terms of the Creative Commons Attribution 4.0 International license.

The sample set was reduced from 170 to 149 sequences by removing samples resulting from apparent clonal (epidemic) expansion (Fig. S2) or having complexity of infection (COI). The Haitian data set was reduced from 21 to nine isolates by selecting the six independent isolates and one representing each of the three epidemic expansions (Table S1). Three isolates from Peru were also removed from two epidemic expansions. When only one isolate representing each epidemic expansion among Haitian and Peruvian samples was utilized in the PCA analysis, similar results were obtained (Fig. 2B and C).

### Phylogenetic relationship of the Haitian parasite to the African, Asian, and South American strains using whole-genome SNPs.

Based on historical data, we would expect the P. falciparum strains found in the Americas to have a paraphyletic origin. This scenario was affirmed by Joy et al., using mtDNA sequences (25), and by Yalcindag et al. (26), using genomic SNPs consisting of a mix of coding and noncoding loci. The first data set we used to investigate the ancestry of American parasite populations was the whole-genome SNP alignment (50,469 loci). From these we removed SNPs under strong positive selection in multiple ways. The first alignment subset included putatively neutral SNPs only, as determined by Bayescan v.2.1 (27–29). This program identifies candidate loci under selection using differences in allele frequencies between populations. After removal of these loci, 48,194 remained. A subset consisting of 15,020 sites was generated by keeping synonymous mutations only. Multinucleotide variants (MNVs) were also eliminated, as different combinations of SNPs in the same codon might result in a nonsynonymous mutation in part of the samples. It is recognized that this approach does not ensure the exclusive selection of neutral mutations, since different synonymous codons could have an impact on gene expression regulation (30). Recognizing that one of the main drivers of selection on P. falciparum upon migration to new areas is the change of vector species (31–34), we also removed from the data sets for analysis the data from genes which are ≥10-fold upregulated in the late gametocyte (gametocyte V), ookinete, and sporozoite stages (rather than the asexual blood stages) (35, 36). The phylogenetic signal of these alignments was verified as shown in Fig. S3.

10.1128/mSphere.00937-20.3 FIG S3 Substitution saturation analysis of different subsets of whole-genome SNP alignment. For each quadrant, the graph represents the pairwise distance (calculated according to the general time-reversible [GTR] model) between sequences plotted versus transitions and transversions. If there is no saturation, both curves should be straight lines. A plateau indicates saturation of substitutions and loss of phylogenetic signal. The table below each graph shows the Xia test and relative *P* value, which takes a statistical approach, verifying that the information entropy-based index of substitution saturation (Iss) for the alignment is significantly lower than the critical value (Iss.cAsym), which would indicate loss of phylogenetic signal. In short, if Iss is not smaller than Iss.cAsym, the sequences have reached substitution saturation. The several iterations of the test were performed using from 4 to 32 randomly chosen operational taxonomic units (OTUs), isolates in our case. (A) Alignment of all SNPs (50,469 sites). (B) Alignment of neutral SNPs, as determined by Bayescan (48,194 sites retained). (C) Alignment after removal of SNPs from genes upregulated in the mosquito stage (44,875 sites retained). (D) Alignment of neutral SNPs. SNPs from genes upregulated in the mosquito stage were removed (42,802 sites retained). (E) Alignment of synonymous SNPs only; MNVs were also removed (15,020 sites retained). (F) Alignment of synonymous SNPs, after removal of loci from genes upregulated in the mosquito stage (13,597 sites were retained). Download FIG S3, TIF file, 0.5 MB. Copyright © 2020 Tagliamonte et al. 2020 Tagliamonte et al. This content is distributed under the terms of the Creative Commons Attribution 4.0 International license.

**FIG 3:**
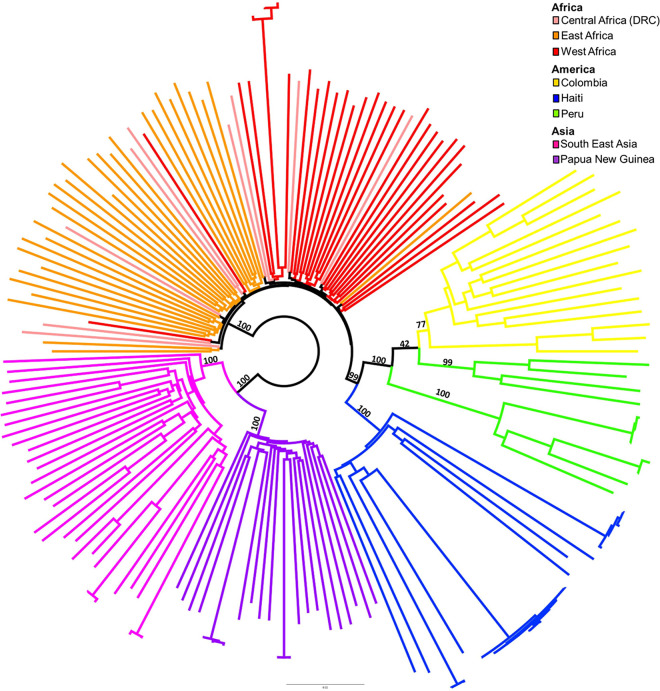
Neighbor-joining tree calculated using synonymous SNPs. MNVs and SNPs in genes upregulated in the mosquito stages of the parasite life cycle were removed (13,627 sites were retained). The tree was calculated using the log-det model.

The neighbor-joining (NJ) tree from the synonymous SNPs minus genes upregulated in the sexual and mosquito stages (13,597 loci) is depicted in Fig. 3. To calculate the tree, we used the log-det model, which has been shown to be robust to biased base composition (37). The other trees are reported in the supplemental material and have similar topologies (Fig. S4), with the separation of populations between continents having strong bootstrap support. The low support for African clades matches the parasite’s known high diversity and high transmission rates on that continent (19, 38). The monophyly of the American clade is always well supported, and the Haitian P. falciparum population is isolated. Looking at the different trees and bootstrap support values, the population structure of the Colombian and Peruvian parasites is not fully resolved and is probably partially mixed. The isolation of the Haitian population has important epidemiological implications, should a larger sample size, covering other areas of the Western Hemisphere, confirm these findings.

10.1128/mSphere.00937-20.4 FIG S4 Neighbor-joining tree calculated using the log-det model. (A) Alignment of all SNPs (50,469 sites). (B) Alignment of neutral SNPs, as determined by Bayescan (48,194 sites retained). (C) Alignment after removal of SNPs from genes upregulated in the mosquito stage (44,875 sites retained). (D) Alignment of neutral SNPs. SNPs from genes upregulated in the mosquito stage were removed (42,802 sites retained). (E) Alignment of synonymous SNPs only; MNVs were also removed (15,020 sites retained). Download FIG S4, TIF file, 1.4 MB. Copyright © 2020 Tagliamonte et al. 2020 Tagliamonte et al. This content is distributed under the terms of the Creative Commons Attribution 4.0 International license.

### Selective pressure of American mosquito vectors on P. falciparum genes.

The results of the phylogenetic analyses, showing a monophyletic American clade, are counterintuitive, because of the scenario involving continuous introduction from different parts of the African continent through the slave trade. Considering the extent to which we went to remove loci potentially under selection, these results likely derive from the use of coding SNPs and reflect a genuine bottleneck which the parasite went through after it was introduced into the Americas. The most dramatic change to which P. falciparum had to adapt was transmission by novel definitive hosts, as the American *Anopheles* (*Nyssorhynchus*) spp. diverged from the African *Anopheles* (*Cellia*) spp. ∼100 million years ago (17). Evidence has recently emerged regarding the impact that novel vectors have had on the Pfs47 gene (31–34, 39, 40). Allelic changes in Pfs47 partially control infectivity for different vectors, suggesting that this and perhaps additional genes are under selection during adaptation to novel vector species.

A preliminary test was performed on our alignment as a way to identify potential genes with mutations necessary for adaptation to the American vectors. For these analyses, the Haitian data set was reduced from 21 to the nine isolates representative of the Haitian subclades (Fig. 3) by selecting the six independent isolates and one representing each of three apparent epidemic expansions (Table S1). Three isolates from Peru were also removed from two apparent epidemic expansions. P. falciparum expression data (35, 36) were downloaded from PlasmoDB26 database (41), and we identified those genes that are upregulated in the gametocyte, ookinete, and sporozoite stages as opposed to the blood stages. We then split the alignment in two, depending on SNPs belonging to genes upregulated in the mosquito stages compared to the blood stages. We compared the ratio of nonsynonymous to synonymous substitutions (*dN*/*dS*); *dN*/*dS* is statistically higher in the subset data from genes upregulated in the mosquito stages (1.01 versus 0.53; *P* < 0.01), further suggestive of the impact that the vector might have had on the parasite population.

As evolutionary rates and substitution patterns may vary between genes, we tried to narrow our data set to a few more likely candidate genes for further, in-depth analyses. Genes under strong selection were preliminarily identified by filtering to identify nonsynonymous mutations having a frequency of ≥0.7 in the 33 American isolates (9 Haitian, 16 Colombian, and 8 Peruvian) and ≤0.3 in the 70 African isolates; 68 variant genes were retained of the 3,106-gene data set. In this data set, 397 genes (13.1%) were upregulated in one or more of these sexual/mosquito stages of the parasite life cycle, but among the retained 68 genes, the frequency of sexual/mosquito-stage genes was almost 2-fold higher, with 17 (25%) being upregulated in one or more of these stages (Table 1). Twelve of these genes contained a single variant codon with differential frequency; one had two such mutations, three had five, and one had seven. The four genes with the most mutations were TRAP (PF3D7_1335900), CTRP (PF3D7_0315200), PSOP26 (PF3D7_1244500), and Pfs47 (PF3D7_1346800).

**TABLE 1:** P. falciparum genes upregulated in the mosquito stage

Gene name	Gene ID	Product description	No. of variant codons in American strains^*a*^	Upregulation (fold) in stage^*b*^		
				Gametocyte V	Ookinete	Sporozoite
TRAP	PF3D7_1335900	Thrombospondin-related anonymous protein	7	0	0	3,235.7
CTRP	PF3D7_0315200	Circumsporozoite- and TRAP-related protein	5	0	582.1	0
PSOP26	PF3D7_1244500	Conserved *Plasmodium* protein, unknown function	5	0	133	0
P47	PF3D7_1346800	6-cysteine protein	5	37.6	27.8	0
NA	PF3D7_0511400	Conserved *Plasmodium* protein, unknown function	2	0	0	147.4
SIAP1	PF3D7_0408600	Sporozoite invasion-associated protein 1	1	0	0	270.7
NA	PF3D7_0515500	Amino acid transporter, putative	1	27.2	0	0
PBLP	PF3D7_0818600	BEM46-like protein, putative	1	0	0	33.4
CRMP1	PF3D7_0911300	Cysteine repeat modular protein 1	1	0	0	14
ICP	PF3D7_0911900	Falstatin	1	0	0	11.4
NA	PF3D7_0924600	Conserved *Plasmodium* protein, unknown function	1	29.4	19.8	0
NA	PF3D7_1020200	Conserved *Plasmodium* protein, unknown function	1	86.5	223.6	0
CRMP3	PF3D7_1208200	Cysteine repeat modular protein 3	1	0	0	16.8
P48/45	PF3D7_1346700	6-cysteine protein	1	25	15.8	0
NA	PF3D7_1348400	Conserved *Plasmodium* membrane protein, unknown function	1	20	0	0
NA	PF3D7_1403200	Conserved *Plasmodium* protein, unknown function	1	158.9	82.1	0
SOAP	PF3D7_1404300	Secreted ookinete adhesive protein, putative	1	0	70.8	0

a Variant codons which have ≥0.7 frequency in America and ≤0.3 frequency in Africa.

b Determined by comparison to blood stages.

The consensus sequences of these genes were compiled for a representative subset of the isolates, as described in Text S1. No sign of recombination was found by RDP4 analysis (42), performed as described by Mavian et al. (43). Selection analysis was performed by a fast, unconstrained Bayesian approximation algorithm (FUBAR) (44). Using a posterior probability cutoff of 0.8 and codon frequencies of ≥0.7 in the American isolates and ≤0.3 in African isolates, we identified 24 codons among these four genes under positive selection (Tables 2to 5). Complete FUBAR results are shown in Table S3.

**TABLE 2:** FUBAR results for CTRP^*a*^

Codon no.	Amino acids	Amino acid frequencies in:				FUBAR posterior probability of positive selection
		Haiti	South America	America	Africa	
17	H, P	1, 0	0.96, 0.04	0.97, 0.03	0, 1	0.95
319	N, D	1, 0	0.96, 0.04	0.97, 0.03	0, 1	0.82
659	R, Q	1, 0	1, 0	1, 0	0.3, 0.7	0.94
1046	K, N	1, 0	0.88, 0.12	0.91, 0.09	0, 1	0.83
1260	N, S	1, 0	0.92, 0.08	0.94, 0.06	0.27, 0.73	0.96
2093	P, S	1, 0	0.62, 0.38	0.73, 0.27	0, 1	0.94
2098	Q, E	1, 0	0.96, 0.04	0.97, 0.03	0.17, 0.83	0.96

a PF3D7_0315200 (circumsporozoite and TRAP-related protein; 2,114 amino acids [aa]). Only codons with differential frequencies between Haiti and Africa were retained.

**TABLE 3:** FUBAR results for PSOP26^*a*^

Codon no.	Amino acids	Amino acid frequencies in:				FUBAR posterior probability of positive selection
		Haiti	South America	America	Africa	
209	F, V	1, 0	0.88, 0.12	0.91, 0.09	0, 1	0.84
494	P, R	1, 0	1, 0	1, 0	0, 1	0.80
664	R, S	1, 0	1, 0	1, 0	0, 1	0.93
722	K, N	1, 0	1, 0	1, 0	0, 1	0.87
736	N, K	1, 0	1, 0	1, 0	0.07, 0.93	0.96

a PF3D7_1244500 (conserved *Plasmodium* protein, unknown function; 810 aa). Only codons with differential frequencies between Haiti and Africa were retained.

**TABLE 4:** FUBAR results for TRAP^*a*^

Codon no.	Amino acids	Amino acid frequencies in:				FUBAR posterior probability of positive selection
		Haiti	South America	America	Africa	
66	K, N	0.56, 0.44	0.92, 0.08	0.82, 0.18	0.1, 0.9	1.00
83	E, D	0.78, 0.22	1, 0	0.94, 0.06	0.23, 0.77	0.96
92	I, V	0.78, 0.22	0.92, 0.08	0.88, 0.12	0, 1	0.98
277	L, I, T	0.89, 0.11, 0	0.92, 0.08, 0	0.91, 0.09, 0	0.27, 0.7, 0.03	0.99
297	Q, H, D	0.67, 0.33, 0	0.92, 0.08, 0	0.85, 0.15, 0	0, 0.53, 0.47	0.97
509	R, K	0.67, 0.33	0.83, 0.17	0.79, 0.21	0, 1	0.92
541	F, Y	0.67, 0.33	0.92, 0.08	0.85, 0.15	0, 1	0.84

a PF3D7_1335900 (thrombospondin-related anonymous protein; 574 aa). Only codons with differential frequencies between Haiti and Africa were retained.

**TABLE 5:** FUBAR results for Pfs47^*a*^

Codon no.	Amino acids	Amino acid frequencies in:				FUBAR posterior probability of positive selection
		Haiti	South America	America	Africa	
178	V, I	1, 0	0.83, 0.17	0.87, 0.13	0, 1	0.86
236	I, T	1, 0	1, 0	1, 0	0, 1	0.85
242	L, S	1, 0	1, 0	1, 0	0, 1	0.86
247	A, V	1, 0	1, 0	1, 0	0, 1	0.86
248	L, I	1, 0	1, 0	1, 0	0.17, 0.83	0.94

a PF3D7_1346800 (6-cysteine protein; 439 aa). Only codons with differential frequencies between Haiti and Africa were retained.

10.1128/mSphere.00937-20.8 TABLE S3 FUBAR selection analysis. Complete table. Download Table S3, DOCX file, 0.6 MB. Copyright © 2020 Tagliamonte et al. 2020 Tagliamonte et al. This content is distributed under the terms of the Creative Commons Attribution 4.0 International license.

## DISCUSSION

Whole-genome analysis for P. falciparum is particularly complicated, due to the repetitive nature and high AT content of the genome, requiring an intense effort to eliminate analysis artifacts. Further, P. falciparum also regularly undergoes sexual recombination, which constitutes an additional obstacle to phylogenetic analyses. Despite these challenges and potential limitations, the resulting data can help answer a multiplicity of questions, including shedding light on the variety of evolutionary drivers acting on the parasite (19, 20, 45). The whole-genome sequence data reported here are the first available from Haitian P. falciparum isolates. These data were obtained from isolates obtained in Grand’Anse plus one in Sud-Est, two regions with the highest rates of transmission of malaria in Haiti. Comparisons of the genotypes of these isolates to those obtained by Charles et al. (14) indicate that data obtained from the isolates analyzed reasonably represent the entire Haitian P. falciparum population.

A large proportion of the coding SNPs common to the Haitian P. falciparum parasite population are now known, and there are many SNPs that are unique and private to the Haiti population, based on our analysis. This knowledge will aid in the elimination of malaria from the island, by offering the ability to discern imported infections from indigenous ones. Reassessing this scenario with data from additional strains from Central America and the eastern areas of South America will be essential to monitor movements of the parasite between these regions and consequent possible reintroductions.

The shape of the NJ tree (Fig. 3), with long terminal branches, could derive from bottlenecks resulting from multiple strategies to fight malaria but are more likely due to less recent ones resulting from the necessity of adapting to new definitive hosts following migration to Asia and the Americas, followed by genetic drift. The presence of such bottlenecks represents an obstacle difficult to overcome in any coalescent analysis. However, coding SNPs have been valuable for differentiating among populations (19, 20, 46, 47), and these data show that the Haitian population is clearly distinct from Colombian and Peruvian populations.

Based upon historical data, we would expect the American parasite population to be paraphyletic; however, this is not seen. The monophyly of the American clade as seen in our analyses likely derives from the use of coding SNPs. Our synonymous SNPs may be largely neutral (30, 48), but they have probably been subjected to repeated selective sweeps and were fixed along with mutations that are advantageous in Haiti and other regions of the Americas. Since the American isolates appear as a single clade despite millions of introductions in widely separated geographical regions, this suggests a common selective bottleneck. Branching within this clade into distinct subclades is then created by local bottlenecks and geographical isolation followed by genetic drift.

The greatest challenge to the parasite in the transition to the Americas was the sudden change in the definitive host, resulting from the transoceanic migration of the intermediate host. Finding the proportion of genes under strong selection expressed in the sexual/mosquito stages to be twice that which was expected provided further impetus to evaluate the hypothesis that the common bottleneck experienced was the required adaptation to transmission by *Anopheles* (*Nyssorhynchus*) spp. in all locales. This was followed by further adaptation to additional species-specific selective pressures presented by local vectors of this subgenus. Genes which are upregulated in the mosquito stages of the life cycle of the parasite are thus potentially under selective drive when the parasite shifts from one vector species to another, and they are scattered across the P. falciparum genome on different chromosomes, which would exacerbate the genetic bottleneck acting upon the parasite population.

It is estimated that the African and American anopheline vectors evolved independently for nearly 100 million years (17), offering an evolutionary basis for major differences in the genus. Further, evidence has recently emerged regarding the impact that the immune systems of novel vectors have had on the parasite genome. Key mutations in the Pfs47 gene allow P. falciparum to escape the complement-like immune system of its definitive host, where different optimal combinations of the amino acid substitutions are necessary for the successful infection of vector species in different regions of the world (31–34, 39, 40). The crucial role of this gene product was first identified through linkage analysis of the progeny of a cross between GB4 (an African isolate) and 7G8 (a Brazilian isolate). While multiple selective pressures have shaped the low-diversity American parasite populations, our research correctly identified Pfs47 and the four codons (codons 236, 242, 247, and 248) for which there is *in vivo* evidence of being under selection by the vector (31) The fifth codon identified here (codon 178) might play an additional role in the successful infection of Anopheles albimanus, the dominant vector on Hispaniola, since presumably progenitor strains giving rise to 7G8 were adapted for successful transmission via Anopheles darlingi, the dominant vector in Brazil.

The other three genes we identified have not been previously reported to contribute to adaptation to specific vectors. TRAP (PF3D7_1335900) is a protein with two adhesive domains (the A and TSR domains) and is essential for trafficking to the salivary glands of the mosquito (49). Five of the seven SNPs under selection in American isolates (three in the A domain and two in TSR) are found in these two regions of the protein. CTRP (PF3D7_0315200) is a conserved protein essential to ookinete motility and invasion of mosquito midgut epithelium (50). This protein has a COOH-terminal transmembrane domain and a short cytoplasmic domain with a possible rhomboid protease cleavage site adjacent to the external face of the transmembrane domain (50). The function of the fourth gene, PSOP26 (PF3D7_1244500), is unknown; however, our results suggest an important role for this protein, which is expressed in the ookinete stage, in the interface with the vector. Interestingly, both the Honduran isolate HB3 (NCBI accession no. GCA_900631985.1) and the Salvadoran isolate Santa Lucia (NCBI accession no. GCA_000150455.3) present all 24 of the mutations located in these four genes, which we found under selection in the American isolates evaluated here. Conversely, the isolate 7G8, originally from Brazil (NCBI accession no. GCA_000150435.3), presents only 17 of the 24 mutations, where the predominant African alleles are found: codon 178 in the Pfs47 gene; codons 1046, 1260, and 2093 in the CTRP gene; and codons 66, 509 and 541 in the TRAP gene. This suggests that different American vectors may exert different selective pressures on some of the codons. Another possibility is that not all of the codons that were positive in the *in silico* analyses are actually under selection. Some SNPs might be associated by chance with other positively selected codons and thus have “hitchhiked” on the selective drive. Also, low diversity and isolation of various American populations will have had an impact on selection analyses, thus potentially resulting in some false positives.

The present results constitute a starting point for investigating gene products which have a high likelihood of having a crucial interaction with the mosquito vector. The next step would be to assess the effect of such mutations *in vivo* on transmission efficiency in different vectors. This would help not only define the effect of specific alleles but also illustrate the functions of protein products that are poorly characterized thus far. This knowledge of relevant protein variants also might help in developing transmission blocking vaccines by identifying genes products critical for transmission. Such vaccines may target either a crucial parasite protein, as recently demonstrated for Pfs47 (51), or the functional contact between that parasite protein and the vector (52, 53).

Other genes, expressed in the human host, might also be under mosquito selective pressure, indirectly. As an example, *A. albimanus*, the vector on Hispaniola, has a strong (20:1) preference for livestock versus humans and is exophilic and exophagic (54), greatly reducing the intensity of transmission and severity of infection. Parasite alleles promoting enhanced transmission through the Haitian vector and persistent human infections with long-term production of gametocytes were probably under immediate selection. Further, these selective pressures would be expected to limit the productive gene flow from P. falciparum from other hemispheres, including drug resistance alleles, as these strains would not be competitive with the adapted local strains.

Our comparatively small sample and the small number of published sequences from American parasites limit the current phylogenetic reconstruction. Recent technologies, such as selective whole-genome amplification (sWGA) and single-cell sequencing, have been successfully implemented with *Plasmodium* spp. (55–57), and blood spots collected on filter paper are finally usable for whole-genome analysis. This will facilitate expanding access to isolates in the future, adding greatly to the data set of whole-genome sequences available from P. falciparum populations in the Americas.

## MATERIALS AND METHODS

### Sample collection and processing.

This study was conducted in accordance with institutional review board guidelines and requirements of the University of Florida and the ethical review board of the Haitian Ministry of Health, after all permits and approvals had been obtained (IRB201400225; MSPP reference no. 1314-62). Blood samples were collected with informed consent from patients who were positive for malaria by a rapid diagnostic test during the period of September 2014 through February 2015 and subsequently deidentified. Aliquots of some of the samples were also placed in *in vitro* culture (58). Leukocytes were removed using either CF11 cellulose columns (59) or Plasmodipur filters (catalog no. 8011Filter25u; EuroProxima BV). Following DNA extraction (genomic DNA midikit; Zymo, Inc.) and in preparation for constructing a sequencing library, the amount of parasite and human DNA recovered from each sample was estimated by TaqMan-based qPCR as described in Text S1. DNA from each primary isolate was utilized for microsatellite analysis and for sequencing library preparation, as described in Text S1.

### Genomic sequencing and data quality analysis.

Sequencing was performed on an Illumina MiSeq system using the Illumina MiSeq reagent kit v3 reagents according to the manufacturer’s instructions to generate 300-nucleotide paired-end reads.

The general quality of the sequence data was assessed using Fastqc v. 0.11.4 (60). Primer dimers and leftover insert sequences were removed from the Haitian sample sequences with Trimmomatic v.0.36 (61), and Trim Galore (62) was used for downloaded data sets.

### Variant calling and filtering.

Variant calling was performed by following directions from the SAMtools 1.3.1 pipeline (63) as described at http://www.htslib.org/workflow/#mapping_to_variant and in reference 64. Results were annotated using SnpEff v.4.2 (65). Since the P. falciparum genome has a high AT bias (∼82% AT content) and is rich in repetitive segments, it was necessary to further refine these results, using a protocol defined by Manske et al. (19), which we implemented with custom scripts in R language v.3.3.1 (66) through the RStudio shell (67) and Shustring (68). Details of this pipeline and the impact of filtering steps on the two data sets are reported at https://drive.google.com/file/d/1dA_TPvuJGEiz41w2fU8Q82bOCJNU4JIJ/view?usp=sharing.

Principal-component analysis (PCA) and spatial principal-component analysis (sPCA) were performed on the resulting data as described in Text S1. Genetic distance was calculated with the general time-reversible model versus transitions and transversions and the Xia test (69) was performed using DAMBE v. 6.4.81 (70). The final alignment (13,627 sites) was scanned for recombination using GARD (71) as implemented in HyPhy (72).

Phylogenetic analyses were performed as described in Text S1. *dN* and *dS* were calculated using DnaSP v.6.12.03 (73).

### Data availability.

The sequence data obtained from the 21 Haitian isolates used in this study were filtered to remove sequences not mapping to the P. falciparum genome. Read pairs for which at least one read mapped to the P. falciparum genome were uploaded to the SRA database with project number PRJNA603776.
