# Supplementary material for: Genetic Markers of Adaptation of Plasmodium falciparum to Transmission by American Vectors Identified in the Genomes of Parasites from Haiti and South America
Source: mSphere. 2020 Oct 21;5(5):e00937-20. doi: 10.1128/mSphere.00937-20 (PMC7580960; doi:10.1128/mSphere.00937-20)
Supplement: TEXT S1 [file mSphere.00937-20-s0001.docx]

Text S1. **Supplementary Methods**

Sequencing Library Preparation. The amounts of *P. falciparum* and human DNA in each sample were estimated by quantitative PCR using the gene for AMA1 (chromosome 11, gene GC content: 30.6%, forward primer: CGCATATCCAATAGACCACGA, probe: CGAACCCGCACCACAAGAACAAA, reverse primer: CCGTCCATGGATTACCCATATAA) and TLR9 (forward primer: CCCAGTCTTGGACTCAGAATTAG, probe TCTAGGTCTCAGTCCTGGTTCTGAAGC,; reverse primer: GGTATAGCCAGGGATTGGTTAAG), respectively. The standard curves were prepared by serial dilution of a quantified stock solution of *P. falciparum* 3D7 DNA from culture and human DNA, respectively. The NEBNext® Microbiome DNA Enrichment Kit was used to remove human DNA which is rich in methylated CpG islands, while the parasite genetic material remains unbound (1). Human mitochondrial DNA did not bind to the beads and remained a human DNA contaminant. Following purification, the parasite DNA was amplified using the REPLI-g Single Cell Kit (Qiagen). To reduce errors and biases in coverage, we modified the protocol by adding tetramethylammonium chloride (TMAC) at a final concentration of 60 mM shown to reduce indel errors and coverage bias in the amplification of the *P. falciparum* genome, especially in the presence of residual host genetic material (2). DNA from parasites propagated in vitro was not amplified. DNA selected for library preparation was sheared using a Covaris sonicator, to achieve fragment lengths of 600-1,000 nucleotides. Libraries were made using NEBNext DNA library Prep Master Mix Set for Illumina. The protocol was designed to ligate the sequencing adaptors via a small number of PCR cycles (up to 4 cycles), thus minimizing the introduction of polymerase biases and errors in our libraries.

Population Genetic and Phylogenetic Analyses. Principal Component Analysis (PCA) and spatial Principal Component Analysis (sPCA) were performed using Adegenet v. 2.0.1 (3, 4). Genetic distance calculated with the general time reversible model vs transitions and transversions, and the Xia test (5) were performed using DAMBE v. 6.4.81 (6).

Phylogenetic analyses were performed with PAUP* v. 4.0a165 (7), using the log-det estimator (8), which is robust to biased base composition. Trees were visualized using FigTree v.1.4.3 (9). The phylogenetic signal of alignments was tested with DAMBE v. 6.4.81 (6). Format conversions were either done with custom scripts or PGDSpider v.2.1.0.3 (10).

Samples resulting from clonal expansion were identified using POPPR v.2.8.0 (11, 12); Haitian and South American clonal samples were also confirmed using Isorelate (13). Then THE REAL McCOIL (14) was used to infer the number of clones within each sample (complexity of infection, COI). Following these steps, we kept one representative of each group of clones as identified by POPPR; also, samples which with a COI> 2 were removed. This filtering kept 149 out of the initial 170 samples.

The custom R scripts used for this work can be found at: <https://github.com/mstagliamonte/PfalciparumScripts> .

Selection analysis on *P. falciparum* genes expressed in the mosquito stages. Out of the 149 isolates subset, African sequences were further randomly downsampled to 30 so to have similar sample size by continent, resulting in 110 total sequences. Consensus sequences for genes CTRP, PSOP26, TRAP and Pf47 were created by calling the segment genotype with SAMtools v 1.3.1 pipeline (15). Custom R scripts were used to identify unreliable mutations in segments of low coverage associated with high intra-sample heterozygosity and to generate the fasta files. Codons 766-841 for CTRP gene, 355-368 for TRAP and codons 223-231 and 411-424 for the PSOP26 gene had low overall coverage and high percent of multiallelic calls. The calls at these loci were deemed unreliable for all isolates and coded as missing data. Sequences from Africa were randomly chosen (15 sequences from West Africa and 15 from East Africa) in order to have similar number of isolates from each continental branch. The final dataset included 9 sequences from Haiti, 16 from Colombia, 8 from Peru, 28 from South East Asia, 19 from Papua New Guinea and 30 from Africa, for a total of 120 sequences. The alignments were scanned for recombination using RDP4 (16) as in (17). Selection analysis was performed with FUBAR (18) using the Datamonkey webserver (19-21).

References

1. Oyola SO, Gu Y, Manske M, Otto TD, O'Brien J, Alcock D, et al. Efficient depletion of host DNA contamination in malaria clinical sequencing. J Clin Microbiol. 2013;51(3):745-51.

2. Oyola SO, Manske M, Campino S, Claessens A, Hamilton WL, Kekre M, et al. Optimized whole-genome amplification strategy for extremely AT-biased template. DNA Res. 2014;21(6):661-71.

3. Jombart T. adegenet: a R package for the multivariate analysis of genetic markers. Bioinformatics (Oxford, England). 2008;24(11):1403-5.

4. Jombart T, Ahmed I. adegenet 1.3-1: new tools for the analysis of genome-wide SNP data. Bioinformatics (Oxford, England). 2011;27(21):3070-1.

5. Xia X, Xie Z, Salemi M, Chen L, Wang Y. An index of substitution saturation and its application. Molecular phylogenetics and evolution. 2003;26(1):1-7.

6. Xia X. DAMBE6: New Tools for Microbial Genomics, Phylogenetics, and Molecular Evolution. J Hered. 2017;108(4):431-7.

7. Swofford DL. PAUP*. Phylogenetic Analysis Using Parsimony (*and Other Methods). Version 4.: Sinauer Associates, Sunderland, Massachusetts.; 2003.

8. Massingham T, Goldman N. Statistics of the log-det estimator. Mol Biol Evol. 2007;24(10):2277-85.

9. Rambaut A. FigTree 2006 [Available from: <http://tree.bio.ed.ac.uk/software/figtree/>.

10. Lischer HE, Excoffier L. PGDSpider: an automated data conversion tool for connecting population genetics and genomics programs. Bioinformatics. 2012;28(2):298-9.

11. Kamvar ZN, Tabima JF, Grunwald NJ. Poppr: an R package for genetic analysis of populations with clonal, partially clonal, and/or sexual reproduction. PeerJ. 2014;2:e281.

12. Kamvar ZN, Brooks JC, Grünwald NJ. Novel R tools for analysis of genome-wide population genetic data with emphasis on clonality. Front Genet. 2015;6:208.

13. Henden L, Lee S, Mueller I, Barry A, Bahlo M. Identity-by-descent analyses for measuring population dynamics and selection in recombining pathogens. PLoS Genet. 2018;14(5):e1007279.

14. Chang HH, Worby CJ, Yeka A, Nankabirwa J, Kamya MR, Staedke SG, et al. THE REAL McCOIL: A method for the concurrent estimation of the complexity of infection and SNP allele frequency for malaria parasites. PLoS Comput Biol. 2017;13(1):e1005348.

15. Li R, Li Y, Fang X, Yang H, Wang J, Kristiansen K. SNP detection for massively parallel whole-genome resequencing. Genome Res. 2009;19(6):1124-32.

16. Martin DP, Murrell B, Golden M, Khoosal A, Muhire B. RDP4: Detection and analysis of recombination patterns in virus genomes. Virus Evol. 2015;1(1):vev003.

17. Mavian C, Rife BD, Dollar JJ, Cella E, Ciccozzi M, Prosperi MCF, et al. Emergence of recombinant Mayaro virus strains from the Amazon basin. Sci Rep. 2017;7(1):8718.

18. Murrell B, Wertheim JO, Moola S, Weighill T, Scheffler K, Kosakovsky Pond SL. Detecting individual sites subject to episodic diversifying selection. PLoS Genet. 2012;8(7):e1002764.

19. Delport W, Poon AF, Frost SD, Kosakovsky Pond SL. Datamonkey 2010: a suite of phylogenetic analysis tools for evolutionary biology. Bioinformatics. 2010;26(19):2455-7.

20. Pond SL, Frost SD, Muse SV. HyPhy: hypothesis testing using phylogenies. Bioinformatics. 2005;21(5):676-9.

21. Pond SL, Frost SD. Datamonkey: rapid detection of selective pressure on individual sites of codon alignments. Bioinformatics. 2005;21(10):2531-3.
