# Supplementary material for: Genetic Markers of Adaptation of Plasmodium falciparum to Transmission by American Vectors Identified in the Genomes of Parasites from Haiti and South America
Source: mSphere. 2020 Oct 21;5(5):e00937-20. doi: 10.1128/mSphere.00937-20 (PMC7580960; doi:10.1128/mSphere.00937-20)
Supplement: TABLE S1 [file mSphere.00937-20-st001.docx]

Table S1. **Sequencing and mapping statistics.** Samples with at least 40X coverage were used for variant calling with the reduced set indicated following post clonal analysis.

| Sample Name | Origin | Post Mapping Average Genome X Coverage | Sample kept post clonal analyses (Y/N) |
| --- | --- | --- | --- |
| 14-3-0369 | GA_S1 | 55.62 | Y |
| 14-3-0435 | GA_S1 | 60.67 | N |
| 14-3-0436 | GA_S1 | 58.17 | N |
| 14-3-0437 | GA_S1 | 63.00 | Y |
| 14-3-0441_culture | GA_S1 | 61.51 | Y |
| 14-3-0443 | GA_S1 | 45.89 | N |
| 14-3-0444_culture | GA_S1 | 55.37 | N |
| 15-3-0747 | SED | 42.81 | Y |
| 15-3-0804 | GA_S2 | 82.92 | Y |
| 15-3-0805 | GA_S2 | 62.78 | N |
| 15-3-0806 | GA_S2 | 108.64 | Y |
| 15-3-0807 | GA_S2 | 63.86 | N |
| 15-3-1038 | GA_S2 | 59.51 | Y |
| 15-3-1039 | GA_S2 | 114.63 | Y |
| 15-3-1040 | GA_S2 | 60.00 | N |
| 15-3-1062 | GA_S2 | 67.53 | N |
| 15-3-1063 | GA_S2 | 59.44 | N |
| 15-3-1064 | GA_S2 | 57.91 | Y |
| 15-3-1065 | GA_S2 | 57.00 | N |
| 15-3-1066 | GA_S2 | 60.62 | N |
| 15-3-1067 | GA_S2 | 57.24 | N |
