# Supplementary material for: Genetic Markers of Adaptation of Plasmodium falciparum to Transmission by American Vectors Identified in the Genomes of Parasites from Haiti and South America
Source: mSphere. 2020 Oct 21;5(5):e00937-20. doi: 10.1128/mSphere.00937-20 (PMC7580960; doi:10.1128/mSphere.00937-20)
Supplement: TABLE S2 [file mSphere.00937-20-st002.docx]

Table S2. **Samples downloaded from the MalariaGEN Plasmodium falciparum Community Project.**

| Country of Origin | Area of Origin | Year of Collection | ENA Secondary sample accession | ENA Run accession | Study no. | Sample ID |
| --- | --- | --- | --- | --- | --- | --- |
| Cambodia | SEA | 2008 | ERS010054 | ERR018918 | 1031 | PH0044-C |
| Cambodia | SEA | 2008 | ERS010670 | ERR015336 | 1031 | PH0045-C |
| Cambodia | SEA | 2008 | ERS010776 | ERR022902 | 1031 | PH0105-C |
| Cambodia | SEA | 2008 | ERS024123 | ERR036560 | 1031 | PH0108-CW |
| Cambodia | SEA | 2008 | ERS009744 | ERR022517 | 1031 | PH0109‐C |
| Cambodia | SEA | 2008 | ERS009745 | ERR022518 | 1031 | PH0110-C |
| Cambodia | SEA | 2008 | ERS009740 | ERR020114 | 1031 | PH0111‐C |
| Cambodia | SEA | 2008 | ERS010778 | ERR022850 | 1031 | PH0112-C |
| Cambodia | SEA | 2008 | ERS017560 | ERR029970 | 1031 | PH0114-C |
| Cambodia | SEA | 2008 | ERS010155 | ERR020104 | 1031 | PH0116-C |
| Cameroon | WAf | 2013 | ERS418784 | ERR562878 | 1093 | QP0001‐C |
| Cameroon | WAf | 2013 | ERS418792 | ERR562854 | 1093 | QP0002‐C |
| Cameroon | WAf | 2013 | ERS418800 | ERR580560 | 1093 | QP0003‐C |
| Cameroon | WAf | 2013 | ERS418801 | ERR580561 | 1093 | QP0015‐C |
| Cameroon | WAf | 2013 | ERS418808 | ERR562866 | 1093 | QP0004-C |
| Cameroon | WAf | 2013 | ERS418816 | ERR580562 | 1093 | QP0005‐C |
| Cameroon | WAf | 2013 | ERS418817 | ERR580585 | 1093 | QP0017‐C |
| Cameroon | WAf | 2013 | ERS418825 | ERR562830 | 1093 | QP0018‐C |
| Cameroon | WAf | 2013 | ERS418833 | ERR562831 | 1093 | QP0019‐C |
| Cameroon | WAf | 2013 | ERS418856 | ERR562834 | 1093 | QP0010‐C |
| Colombia | SAm | 2011 | ERS032013 | ERR039903 | 1023 | PW0008‐C |
| Colombia | SAm | 2011 | ERS032040 | ERR039930 | 1023 | PW0002‐C |
| Colombia | SAm | 2011 | ERS032059 | ERR039986 | 1023 | PW0009‐C |
| Colombia | SAm | 2011 | ERS032061 | ERR039988 | 1023 | PW0016‐C |
| Colombia | SAm | 2011 | ERS032157 | ERR042222 | 1023 | PW0007‐C |
| Colombia | SAm | 2011 | ERS032158 | ERR042223 | 1023 | PW0017‐C |
| Colombia | SAm | 2011 | ERS032159 | ERR042224 | 1023 | PW0012‐C |
| Colombia | SAm | 2011 | ERS032161 | ERR042226 | 1023 | PW0004‐C |
| Colombia | SAm | 2011 | ERS032162 | ERR042227 | 1023 | PW0003‐C |
| Colombia | SAm | 2011 | ERS032163 | ERR042228 | 1023 | PW0001‐C |
| Colombia | SAm | 2011 | ERS032164 | ERR042229 | 1023 | PW0005‐C |
| Colombia | SAm | 2011 | ERS032165 | ERR042230 | 1023 | PW0006‐C |
| Colombia | SAm | 2011 | ERS032166 | ERR042231 | 1023 | PW0013‐C |
| Colombia | SAm | 2011 | ERS032167 | ERR042232 | 1023 | PW0015‐C |
| Colombia | SAm | 2011 | ERS032168 | ERR042233 | 1023 | PW0014‐C |
| Colombia | SAm | 2011 | ERS032646 | ERR042679 | 1023 | PW0010‐CW |
| DR Congo | CAf | 2013 | ERS347544 | ERR404199 | 1052 | QG0191‐C |
| DR Congo | CAf | 2013 | ERS347560 | ERR404188 | 1052 | QG0193‐C |
| DR Congo | CAf | 2013 | ERS347567 | ERR426017 | 1052 | QG0182‐C |
| DR Congo | CAf | 2013 | ERS347575 | ERR404214 | 1052 | QG0183‐C |
| DR Congo | CAf | 2013 | ERS347584 | ERR404200 | 1052 | QG0196-C |
| DR Congo | CAf | 2013 | ERS347592 | ERR404242 | 1052 | QG0197-C |
| DR Congo | CAf | 2013 | ERS347616 | ERR404202 | 1052 | QG0200-C |
| DR Congo | CAf | 2013 | ERS347623 | ERR404191 | 1052 | QG0189-C |
| DR Congo | CAf | 2013 | ERS347624 | ERR426021 | 1052 | QG0201-C |
| DR Congo | CAF | 2013 | ERS347631 | ERR404204 | 1052 | QG0190-C |
| Gambia | WAf | 2008 | ERS010047 | ERR015390 | 1006 | PA0020-C |
| Gambia | WAf | 2008 | ERS010126 | ERR018936 | 1006 | PA0008-C |
| Gambia | WAf | 2008 | ERS010127 | ERR021977 | 1006 | PA0012-C |
| Gambia | WAf | 2008 | ERS010048 | ERR015365 | 1006 | PA0021-C |
| Gambia | WAf | 2008 | ERS010116 | ERR018933 | 1006 | PA0007-C |
| Gambia | WAf | 2008 | ERS010129 | ERR018937 | 1006 | PA0022-C |
| Gambia | WAf | 2008 | ERS010049 | ERR015403 | 1006 | PA0027-C |
| Gambia | WAf | 2008 | ERS010038 | ERR015381 | 1006 | PA0029-C |
| Gambia | WAf | 2008 | ERS010117 | ERR021975 | 1006 | PA0034-C |
| Gambia | WAf | 2008 | ERS010040 | ERR015398 | 1006 | PA0036-C |
| Ghana | WAf | 2012 | ERS188107 | ERR248967 | 1083 | PF0578‐Cx |
| Ghana | WAf | 2012 | ERS188114 | ERR248968 | 1083 | PF0583‐C |
| Ghana | WAf | 2012 | ERS193670 | ERR234553 | 1083 | PF0590‐C |
| Ghana | WAf | 2012 | ERS193675 | ERR234554 | 1083 | PF0579‐C |
| Ghana | WAf | 2012 | ERS193680 | ERR234555 | 1083 | PF0584‐C |
| Ghana | WAf | 2012 | ERS224913 | ERR337556 | 1083 | PF0587‐C |
| Ghana | WAf | 2012 | ERS246737 | ERR343116 | 1083 | PF0596‐C |
| Ghana | WAf | 2012 | ERS246740 | ERR343093 | 1083 | PF0599‐C |
| Ghana | WAf | 2012 | ERS246744 | ERR343095 | 1083 | PF0603‐C |
| Ghana | WAf | 2012 | ERS246745 | ERR343096 | 1083 | PF0604‐C |
| Kenya | EAf | 2007 | ERS016375 | ERR029088 | 1015 | PC0077‐C |
| Kenya | EAf | 2007 | ERS016376 | ERR029089 | 1015 | PC0078‐C |
| Kenya | EAf | 2007 | ERS016377 | ERR029104 | 1015 | PC0079‐C |
| Kenya | EAf | 2007 | ERS017457 | ERR029412 | 1015 | PC0075‐C |
| Kenya | EAf | 2007 | ERS074223 | ERR205951 | 1027 | PC0104‐C |
| Kenya | EAf | 2007 | ERS074224 | ERR205952 | 1027 | PC0107‐C |
| Kenya | EAf | 2007 | ERS074225 | ERR205953 | 1027 | PC0113‐C |
| Kenya | EAf | 2007 | ERS074227 | ERR205955 | 1027 | PC0122‐C |
| Kenya | EAf | 2007 | ERS132626 | ERR205956 | 1027 | PC0085‐C |
| Kenya | EAf | 2007 | ERS132629 | ERR205959 | 1027 | PC0103‐C |
| Malawi | EAf | 2011 | ERS032647 | ERR042680 | 1022 | PT0001‐CW |
| Malawi | EAf | 2011 | ERS032648 | ERR045656 | 1022 | PT0002‐CW |
| Malawi | EAf | 2011 | ERS032650 | ERR045658 | 1022 | PT0004‐CW |
| Malawi | EAf | 2011 | ERS032654 | ERR045662 | 1022 | PT0008‐CW |
| Malawi | EAf | 2011 | ERS032655 | ERR045663 | 1022 | PT0009‐CW |
| Malawi | EAf | 2011 | ERS040097 | ERR054069 | 1022 | PT0029‐C |
| Malawi | EAf | 2011 | ERS040098 | ERR054070 | 1022 | PT0031‐C |
| Malawi | EAf | 2011 | ERS040102 | ERR054074 | 1022 | PT0041‐C |
| Malawi | EAf | 2011 | ERS164677 | ERR211564 | 1022 | PT0034‐C |
| Malawi | EAf | 2011 | ERS164686 | ERR216472 | 1022 | PT0032‐C |
| Myanmar | SEA | 2011 | ERS086880 | ERR126441 | 1008 | QC0118‐C |
| Myanmar | SEA | 2011 | ERS086902 | ERR126463 | 1008 | QC0115‐C |
| Myanmar | SEA | 2011 | ERS086914 | ERR126475 | 1008 | QC0122‐C |
| Myanmar | SEA | 2011 | ERS086926 | ERR126487 | 1008 | QC0112‐C |
| Myanmar | SEA | 2011 | ERS086939 | ERR126499 | 1008 | QC0116‐C |
| Myanmar | SEA | 2011 | ERS086940 | ERR126500 | 1008 | QC0119‐C |
| Myanmar | SEA | 2011 | ERS086941 | ERR126501 | 1008 | QC0120‐C |
| Myanmar | SEA | 2011 | ERS086942 | ERR126502 | 1008 | QC0121‐C |
| Myanmar | SEA | 2011 | ERS086943 | ERR126503 | 1008 | QC0123‐C |
| Myanmar | SEA | 2011 | ERS086963 | RR126523 | 1008 | QC0125‐C |
| Papua New Guinea | Oce | 2009 | ERS010055 | ERR015321 | 1021 | PN0057‐C |
| Papua New Guinea | Oce | 2009 | ERS010056 | ERR015402 | 1021 | PN0059‐C |
| Papua New Guinea | Oce | 2009 | ERS150789 | ERR175522 | 1021 | PN0073‐C |
| Papua New Guinea | Oce | 2009 | ERS150798 | ERR175531 | 1021 | PN0087‐C |
| Papua New Guinea | Oce | 2009 | ERS150805 | ERR175538 | 1021 | PN0090‐C |
| Papua New Guinea | Oce | 2009 | ERS150852 | ERR175527 | 1021 | PN0079‐C |
| Papua New Guinea | Oce | 2009 | ERS150853 | ERR175530 | 1021 | PN0077‐C |
| Papua New Guinea | Oce | 2009 | ERS150856 | ERR175539 | 1021 | PN0085‐C |
| Papua New Guinea | Oce | 2009 | ERS150859 | ERR175548 | 1021 | PN0063‐C |
| Papua New Guinea | Oce | 2009 | ERS150861 | ERR175554 | 1021 | PN0078‐C |
| Papua New Guinea | Oce | 2009 | ERS150896 | ERR175524 | 1021 | PN0074‐C |
| Papua New Guinea | Oce | 2009 | ERS150897 | ERR175533 | 1021 | PN0086‐C |
| Papua New Guinea | Oce | 2009 | ERS150984 | ERR175542 | 1021 | PN0058‐Cx |
| Papua New Guinea | Oce | 2008 | ERS010093 | ERR018910 | 1021 | PN0054‐C |
| Papua New Guinea | Oce | 2008 | ERS010094 | ERR018911 | 1021 | PN0056‐C |
| Papua New Guinea | Oce | 2008 | ERS150793 | ERR175526 | 1021 | PN0040‐C |
| Papua New Guinea | Oce | 2008 | ERS150796 | ERR175529 | 1021 | PN0014‐C |
| Papua New Guinea | Oce | 2008 | ERS150801 | ERR175534 | 1021 | PN0031‐C |
| Papua New Guinea | Oce | 2008 | ERS150802 | ERR175535 | 1021 | PN0043‐C |
| Papua New Guinea | Oce | 2008 | ERS150810 | ERR175543 | 1021 | PN0008‐C |
| Papua New Guinea | Oce | 2008 | ERS150813 | ERR175546 | 1021 | PN0037‐C |
| Papua New Guinea | Oce | 2008 | ERS157505 | ERR216461 | 1021 | PN0055‐Cx |
| Peru | SAm | 2011 | ERS010050 | ERR019545 | 1013 | PP0005‐C |
| Peru | SAm | 2011 | ERS010192 | ERR012671 | 1013 | PP0002‐C |
| Peru | SAm | 2011 | ERS010215 | ERR012297 | 1013 | PP0004‐C |
| Peru | SAm | 2011 | ERS011443 | ERR022855 | 1013 | PP0011‐C |
| Peru | SAm | 2011 | ERS011444 | ERR022856 | 1013 | PP0010‐C |
| Peru | SAm | 2011 | ERS016330 | ERR027113 | 1013 | PP0012‐C |
| Peru | SAm | 2011 | ERS071910 | ERR123901 | 1013 | PP0014‐C |
| Peru | SAm | 2011 | ERS071911 | ERR123902 | 1013 | PP0015‐C |
| Peru | SAm | 2011 | ERS071916 | ERR123900 | 1013 | PP0013‐C |
| Peru | SAm | 2011 | ERS071917 | ERR123903 | 1013 | PP0016‐C |
| Peru | SAm | 2011 | ERS071918 | ERR114340 | 1013 | PP0017‐C |
| Tanzania | EAf | 2013 | ERS348782 | ERR405238 | 1095 | PE0109‐C |
| Tanzania | EAf | 2013 | ERS348786 | ERR405240 | 1095 | PE0087‐C |
| Tanzania | EAf | 2013 | ERS348794 | ERR405244 | 1095 | PE0113‐C |
| Tanzania | EAf | 2013 | ERS348795 | ERR405245 | 1095 | PE0090‐C |
| Tanzania | EAf | 2013 | ERS348798 | ERR405248 | 1095 | PE0091‐C |
| Tanzania | EAf | 2013 | ERS348805 | ERR405252 | 1095 | PE0105‐C |
| Tanzania | EAf | 2013 | ERS348806 | ERR405253 | 1095 | PE0117‐C |
| Tanzania | EAf | 2010 | ERS354269 | ERR439524 | 1007 | PE0146‐C |
| Tanzania | EAf | 2010 | ERS354272 | ERR439527 | 1007 | PE0147‐C |
| Tanzania | EAf | 2010 | ERS354275 | ERR439530 | 1007 | PE0148‐C |
| Thailand | SEA | 2011 | ERS142830 | ERR164711 | 1052 | PD0477‐C |
| Thailand | SEA | 2011 | ERS142831 | ERR164712 | 1052 | PD0480‐C |
| Thailand | SEA | 2011 | ERS142861 | ERR164695 | 1052 | PD0479‐C |
| Thailand | SEA | 2011 | ERS142867 | ERR164713 | 1052 | PD0472‐C |
| Thailand | SEA | 2011 | ERS336327 | ERR388738 | 1052 | PD0459‐Cx |
| Thailand | SEA | 2005 | ERS010601 | ERR015334 | 1010 | PD0025‐C |
| Thailand | SEA | 2005 | ERS009968 | ERR019541 | 1010 | PD0032‐C |
| Thailand | SEA | 2005 | ERS010531 | ERR012414 | 1010 | PD0045‐C |
| Thailand | SEA | 2005 | ERS010602 | ERR018897 | 1010 | PD0027‐C |
| Thailand | SEA | 2005 | ERS010635 | ERR015355 | 1010 | PD0033‐C |

Area of Origin codes: CAf= Central Africa; EAf = East Africa; Waf = West Africa;

Oce= Oceania; SAm= South America; SEA= South East Asia
