# Supplementary figures and images for: Genetic Markers of Adaptation of Plasmodium falciparum to Transmission by American Vectors Identified in the Genomes of Parasites from Haiti and South America
Source: mSphere. 2020 Oct 21;5(5):e00937-20. doi: 10.1128/mSphere.00937-20 (PMC7580960; doi:10.1128/mSphere.00937-20)

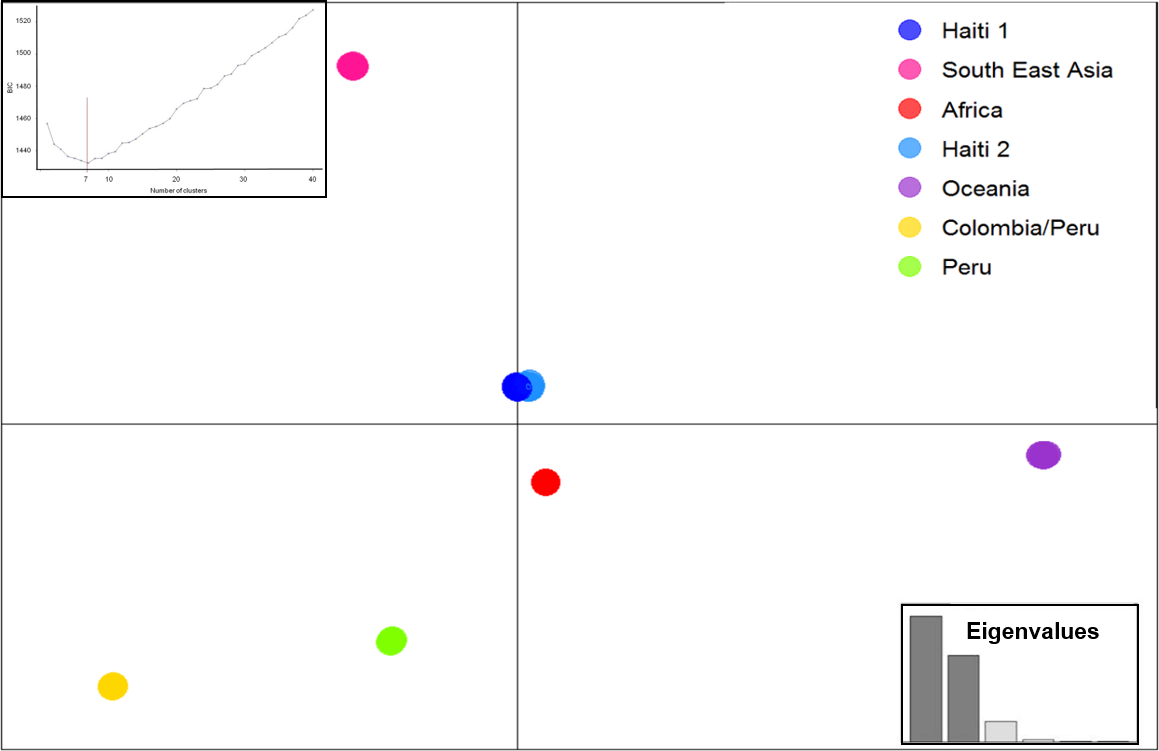

Supplement: FIG S1 [file mSphere.00937-20-sf001.tif]

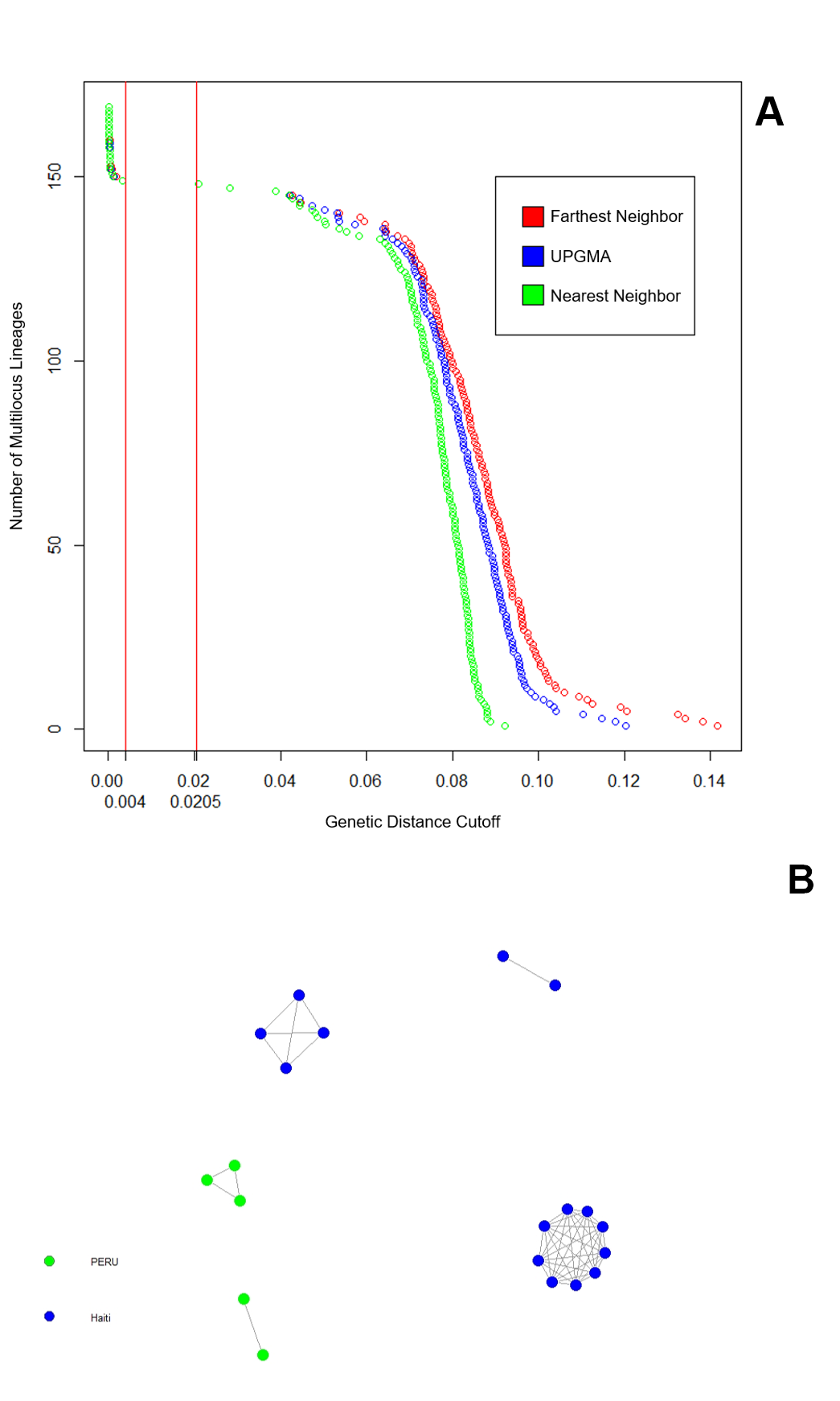

Supplement: FIG S2 [file mSphere.00937-20-sf002.tif]

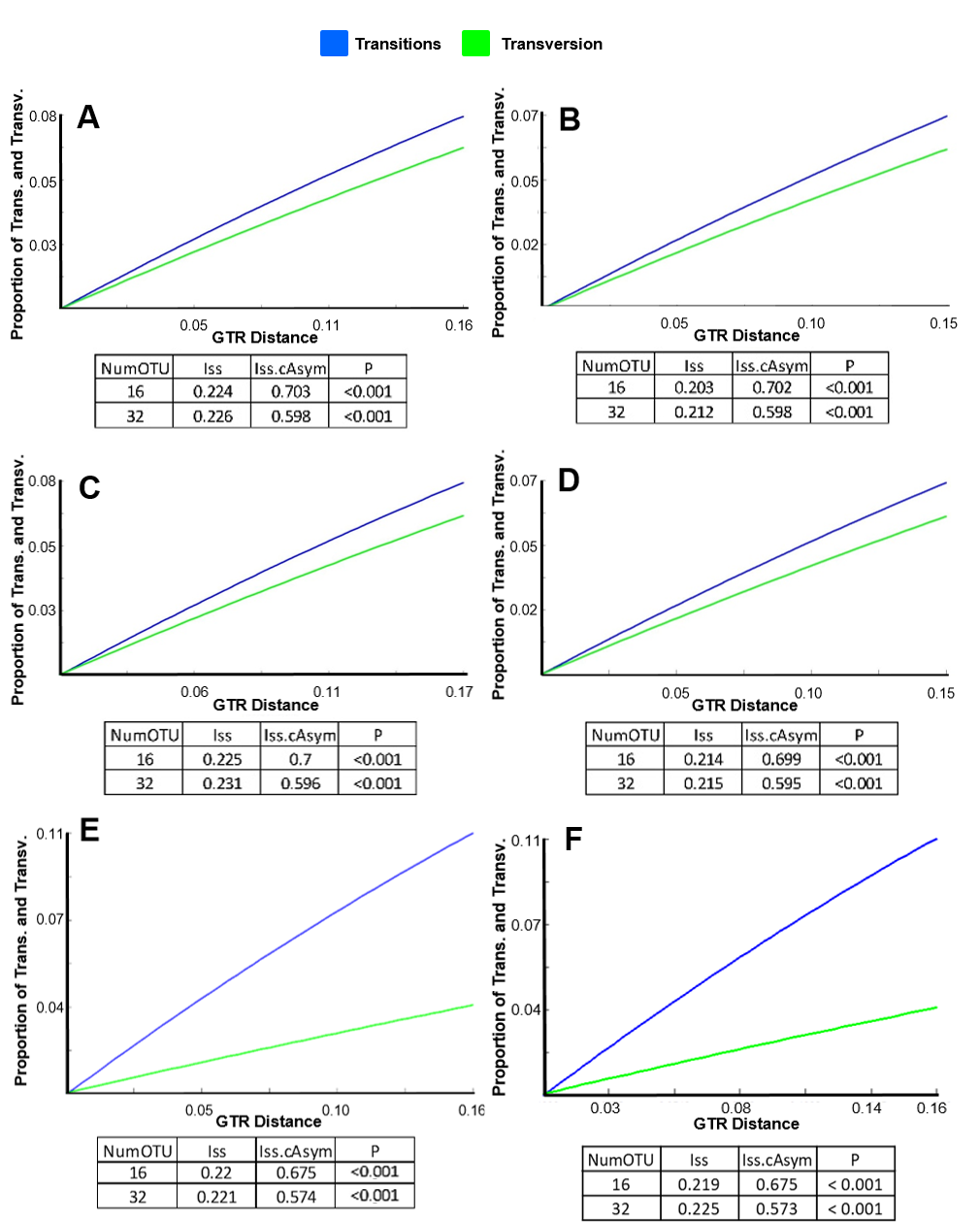

Supplement: FIG S3 [file mSphere.00937-20-sf003.tif]

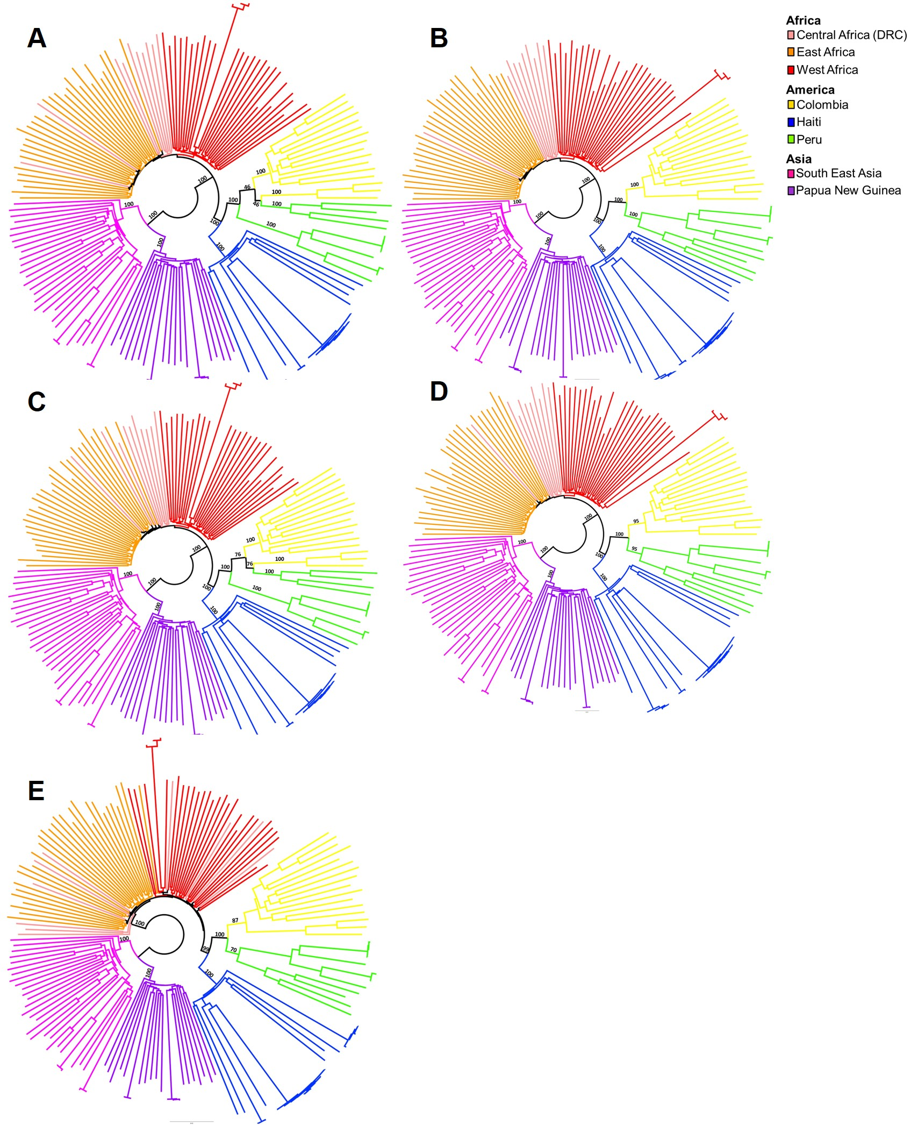

Supplement: FIG S4 [file mSphere.00937-20-sf004.tif]
