# Supplementary material for: Genetic Markers of Adaptation of Plasmodium falciparum to Transmission by American Vectors Identified in the Genomes of Parasites from Haiti and South America
Source: mSphere. 2020 Oct 21;5(5):e00937-20. doi: 10.1128/mSphere.00937-20 (PMC7580960; doi:10.1128/mSphere.00937-20)
Supplement: TABLE S3 [file mSphere.00937-20-st003.docx]

| Table S3. FUBAR selection analysis. Complete table.  PF3D7_0315200 | | | | | | |
| --- | --- | --- | --- | --- | --- | --- |
| Codon | **alpha** | **beta** | **beta-alpha** | **Posterior Prob Positive Selection** | **Posterior Prob Negative Selection** | **Empirical Bayes Factor** |
| 1 | 0.991861 | 0.499837 | -0.49202 | 0.424517 | 0.522062 | 0.943004 |
| 2 | 0.806702 | 0.558688 | -0.24801 | 0.440928 | 0.504893 | 1.00821 |
| 3 | 0.937451 | 0.56278 | -0.37467 | 0.432817 | 0.513608 | 0.975512 |
| 4 | 0.918493 | 0.533206 | -0.38529 | 0.431717 | 0.514568 | 0.971148 |
| 5 | 0.990556 | 0.681067 | -0.30949 | 0.438125 | 0.508702 | 0.996803 |
| 6 | 0.919878 | 0.590742 | -0.32914 | 0.436031 | 0.510368 | 0.988356 |
| 7 | 0.932982 | 0.692761 | -0.24022 | 0.442525 | 0.504113 | 1.01476 |
| 8 | 0.974294 | 0.582579 | -0.39172 | 0.432028 | 0.514591 | 0.972382 |
| 9 | 0.990556 | 0.681067 | -0.30949 | 0.438125 | 0.508702 | 0.996803 |
| 10 | 0.859272 | 0.615434 | -0.24384 | 0.441734 | 0.50446 | 1.01151 |
| 11 | 0.987409 | 0.558677 | -0.42873 | 0.429422 | 0.517219 | 0.962101 |
| 12 | 0.96093 | 0.618709 | -0.34222 | 0.435524 | 0.511096 | 0.986318 |
| 13 | 0.932982 | 0.692761 | -0.24022 | 0.442525 | 0.504113 | 1.01476 |
| 14 | 0.973775 | 0.602736 | -0.37104 | 0.433562 | 0.513086 | 0.978475 |
| 15 | 0.990556 | 0.681067 | -0.30949 | 0.438125 | 0.508702 | 0.996803 |
| 16 | 0.803087 | 0.684908 | -0.11818 | 0.450428 | 0.495711 | 1.04774 |
| 17 | 1.19242 | 11.1663 | 9.97389 | 0.949448 | 0.011625 | 24.0095 |
| 18 | 0.803087 | 0.684908 | -0.11818 | 0.450428 | 0.495711 | 1.04774 |
| 19 | 0.958093 | 0.507466 | -0.45063 | 0.4272 | 0.519228 | 0.95341 |
| 20 | 0.97633 | 0.612906 | -0.36342 | 0.434145 | 0.512528 | 0.9808 |
| 21 | 0.919878 | 0.590742 | -0.32914 | 0.436031 | 0.510368 | 0.988356 |
| 22 | 0.923215 | 0.505613 | -0.4176 | 0.429214 | 0.517042 | 0.961284 |
| 23 | 0.788402 | 0.550533 | -0.23787 | 0.441528 | 0.504185 | 1.01067 |
| 24 | 0.958093 | 0.507466 | -0.45063 | 0.4272 | 0.519228 | 0.95341 |
| 25 | 0.98943 | 0.63651 | -0.35292 | 0.435046 | 0.511714 | 0.984402 |
| 26 | 0.918493 | 0.533206 | -0.38529 | 0.431717 | 0.514568 | 0.971148 |
| 27 | 0.937451 | 0.56278 | -0.37467 | 0.432817 | 0.513608 | 0.975512 |
| 28 | 0.987485 | 0.561586 | -0.4259 | 0.429635 | 0.517011 | 0.962936 |
| 29 | 0.936932 | 0.54188 | -0.39505 | 0.431239 | 0.515148 | 0.969257 |
| 30 | 0.936932 | 0.54188 | -0.39505 | 0.431239 | 0.515148 | 0.969257 |
| 31 | 0.918956 | 0.552208 | -0.36675 | 0.433168 | 0.513155 | 0.976908 |
| 32 | 0.987485 | 0.561586 | -0.4259 | 0.429635 | 0.517011 | 0.962936 |
| 33 | 0.806702 | 0.558688 | -0.24801 | 0.440928 | 0.504893 | 1.00821 |
| 34 | 0.918919 | 5.90558 | 4.98667 | 0.846415 | 0.113891 | 7.04509 |
| 35 | 0.986965 | 0.542034 | -0.44493 | 0.428164 | 0.518452 | 0.95717 |
| 36 | 0.972492 | 0.55289 | -0.4196 | 0.429891 | 0.516675 | 0.963944 |
| 37 | 0.937451 | 0.56278 | -0.37467 | 0.432817 | 0.513608 | 0.975512 |
| 38 | 0.803087 | 0.684908 | -0.11818 | 0.450428 | 0.495711 | 1.04774 |
| 39 | 0.937451 | 0.56278 | -0.37467 | 0.432817 | 0.513608 | 0.975512 |
| 40 | 0.806702 | 0.558688 | -0.24801 | 0.440928 | 0.504893 | 1.00821 |
| 41 | 0.937451 | 0.56278 | -0.37467 | 0.432817 | 0.513608 | 0.975512 |
| 42 | 0.971005 | 0.496861 | -0.47414 | 0.425546 | 0.520927 | 0.946985 |
| 43 | 0.987485 | 0.561586 | -0.4259 | 0.429635 | 0.517011 | 0.962936 |
| 44 | 0.858723 | 0.589883 | -0.26884 | 0.439875 | 0.506259 | 1.00391 |
| 45 | 0.937451 | 0.56278 | -0.37467 | 0.432817 | 0.513608 | 0.975512 |
| 46 | 0.972492 | 0.55289 | -0.4196 | 0.429891 | 0.516675 | 0.963944 |
| 47 | 0.96093 | 0.618709 | -0.34222 | 0.435524 | 0.511096 | 0.986318 |
| 48 | 0.987485 | 0.561586 | -0.4259 | 0.429635 | 0.517011 | 0.962936 |
| 49 | 0.937451 | 0.56278 | -0.37467 | 0.432817 | 0.513608 | 0.975512 |
| 50 | 0.937451 | 0.56278 | -0.37467 | 0.432817 | 0.513608 | 0.975512 |
| 51 | 0.932982 | 0.692761 | -0.24022 | 0.442525 | 0.504113 | 1.01476 |
| 52 | 0.918956 | 0.552208 | -0.36675 | 0.433168 | 0.513155 | 0.976908 |
| 53 | 0.958316 | 0.515962 | -0.44235 | 0.427866 | 0.518577 | 0.956005 |
| 54 | 0.937451 | 0.56278 | -0.37467 | 0.432817 | 0.513608 | 0.975512 |
| 55 | 0.859272 | 0.615434 | -0.24384 | 0.441734 | 0.50446 | 1.01151 |
| 56 | 0.990556 | 0.681067 | -0.30949 | 0.438125 | 0.508702 | 0.996803 |
| 57 | 0.973775 | 0.602736 | -0.37104 | 0.433562 | 0.513086 | 0.978475 |
| 58 | 0.937479 | 0.563894 | -0.37359 | 0.432905 | 0.513523 | 0.975859 |
| 59 | 0.919878 | 0.590742 | -0.32914 | 0.436031 | 0.510368 | 0.988356 |
| 60 | 0.937479 | 0.563894 | -0.37359 | 0.432905 | 0.513523 | 0.975859 |
| 61 | 0.958093 | 0.507466 | -0.45063 | 0.4272 | 0.519228 | 0.95341 |
| 62 | 0.972492 | 0.55289 | -0.4196 | 0.429891 | 0.516675 | 0.963944 |
| 63 | 0.919878 | 0.590742 | -0.32914 | 0.436031 | 0.510368 | 0.988356 |
| 64 | 0.918493 | 0.533206 | -0.38529 | 0.431717 | 0.514568 | 0.971148 |
| 65 | 0.972492 | 0.55289 | -0.4196 | 0.429891 | 0.516675 | 0.963944 |
| 66 | 0.923215 | 0.505613 | -0.4176 | 0.429214 | 0.517042 | 0.961284 |
| 67 | 0.974139 | 0.617137 | -0.357 | 0.434592 | 0.512079 | 0.982586 |
| 68 | 6.9232 | 0.681394 | -6.2418 | 0.061696 | 0.910491 | 0.084055 |
| 69 | 0.918724 | 0.542619 | -0.3761 | 0.432444 | 0.51386 | 0.974028 |
| 70 | 0.78821 | 0.540984 | -0.24723 | 0.440798 | 0.504888 | 1.00768 |
| 71 | 0.918724 | 0.542619 | -0.3761 | 0.432444 | 0.51386 | 0.974028 |
| 72 | 0.972242 | 0.543285 | -0.42896 | 0.429169 | 0.517382 | 0.961107 |
| 73 | 0.987409 | 0.558677 | -0.42873 | 0.429422 | 0.517219 | 0.962101 |
| 74 | 0.973775 | 0.602736 | -0.37104 | 0.433562 | 0.513086 | 0.978475 |
| 75 | 0.917433 | 0.490551 | -0.42688 | 0.428342 | 0.517857 | 0.957865 |
| 76 | 0.986965 | 0.542034 | -0.44493 | 0.428164 | 0.518452 | 0.95717 |
| 77 | 0.986965 | 0.542034 | -0.44493 | 0.428164 | 0.518452 | 0.95717 |
| 78 | 0.958991 | 0.541922 | -0.41707 | 0.429875 | 0.516613 | 0.963881 |
| 79 | 0.96093 | 0.618709 | -0.34222 | 0.435524 | 0.511096 | 0.986318 |
| 80 | 0.958991 | 0.541922 | -0.41707 | 0.429875 | 0.516613 | 0.963881 |
| 81 | 0.937479 | 0.563894 | -0.37359 | 0.432905 | 0.513523 | 0.975859 |
| 82 | 0.987485 | 0.561586 | -0.4259 | 0.429635 | 0.517011 | 0.962936 |
| 83 | 0.98943 | 0.63651 | -0.35292 | 0.435046 | 0.511714 | 0.984402 |
| 84 | 0.961357 | 0.636079 | -0.32528 | 0.436747 | 0.509902 | 0.991236 |
| 85 | 0.986965 | 0.542034 | -0.44493 | 0.428164 | 0.518452 | 0.95717 |
| 86 | 0.932982 | 0.692761 | -0.24022 | 0.442525 | 0.504113 | 1.01476 |
| 87 | 0.918956 | 0.552208 | -0.36675 | 0.433168 | 0.513155 | 0.976908 |
| 88 | 0.932982 | 0.692761 | -0.24022 | 0.442525 | 0.504113 | 1.01476 |
| 89 | 0.97633 | 0.612906 | -0.36342 | 0.434145 | 0.512528 | 0.9808 |
| 90 | 0.803087 | 0.684908 | -0.11818 | 0.450428 | 0.495711 | 1.04774 |
| 91 | 0.957872 | 0.499115 | -0.45876 | 0.426532 | 0.519881 | 0.950809 |
| 92 | 0.936932 | 0.54188 | -0.39505 | 0.431239 | 0.515148 | 0.969257 |
| 93 | 0.974139 | 0.617137 | -0.357 | 0.434592 | 0.512079 | 0.982586 |
| 94 | 0.918493 | 0.533206 | -0.38529 | 0.431717 | 0.514568 | 0.971148 |
| 95 | 0.956877 | 0.462146 | -0.49473 | 0.423501 | 0.522846 | 0.93909 |
| 96 | 0.974294 | 0.582579 | -0.39172 | 0.432028 | 0.514591 | 0.972382 |
| 97 | 0.917433 | 0.490551 | -0.42688 | 0.428342 | 0.517857 | 0.957865 |
| 98 | 0.922331 | 0.696783 | -0.22555 | 0.443477 | 0.50313 | 1.01868 |
| 99 | 0.937451 | 0.56278 | -0.37467 | 0.432817 | 0.513608 | 0.975512 |
| 100 | 0.987485 | 0.561586 | -0.4259 | 0.429635 | 0.517011 | 0.962936 |
| 101 | 0.996136 | 0.661383 | -0.33475 | 0.436404 | 0.510416 | 0.989857 |
| 102 | 1.14345 | 12.1308 | 10.9874 | 0.961008 | 0.003262 | 31.5065 |
| 103 | 0.958316 | 0.515962 | -0.44235 | 0.427866 | 0.518577 | 0.956005 |
| 104 | 0.987224 | 0.551719 | -0.43551 | 0.4289 | 0.517731 | 0.960052 |
| 105 | 0.973488 | 0.591491 | -0.382 | 0.432745 | 0.513885 | 0.975223 |
| 106 | 0.97633 | 0.612906 | -0.36342 | 0.434145 | 0.512528 | 0.9808 |
| 107 | 0.918724 | 0.542619 | -0.3761 | 0.432444 | 0.51386 | 0.974028 |
| 108 | 0.990556 | 0.681067 | -0.30949 | 0.438125 | 0.508702 | 0.996803 |
| 109 | 0.972492 | 0.55289 | -0.4196 | 0.429891 | 0.516675 | 0.963944 |
| 110 | 0.986965 | 0.542034 | -0.44493 | 0.428164 | 0.518452 | 0.95717 |
| 111 | 0.937451 | 0.56278 | -0.37467 | 0.432817 | 0.513608 | 0.975512 |
| 112 | 0.97633 | 0.612906 | -0.36342 | 0.434145 | 0.512528 | 0.9808 |
| 113 | 0.974294 | 0.582579 | -0.39172 | 0.432028 | 0.514591 | 0.972382 |
| 114 | 0.806702 | 0.558688 | -0.24801 | 0.440928 | 0.504893 | 1.00821 |
| 115 | 0.986965 | 0.542034 | -0.44493 | 0.428164 | 0.518452 | 0.95717 |
| 116 | 0.932982 | 0.692761 | -0.24022 | 0.442525 | 0.504113 | 1.01476 |
| 117 | 0.918956 | 0.552208 | -0.36675 | 0.433168 | 0.513155 | 0.976908 |
| 118 | 0.974294 | 0.582579 | -0.39172 | 0.432028 | 0.514591 | 0.972382 |
| 119 | 0.956877 | 0.462146 | -0.49473 | 0.423501 | 0.522846 | 0.93909 |
| 120 | 0.937451 | 0.56278 | -0.37467 | 0.432817 | 0.513608 | 0.975512 |
| 121 | 0.987485 | 0.561586 | -0.4259 | 0.429635 | 0.517011 | 0.962936 |
| 122 | 0.936932 | 0.54188 | -0.39505 | 0.431239 | 0.515148 | 0.969257 |
| 123 | 0.919878 | 0.590742 | -0.32914 | 0.436031 | 0.510368 | 0.988356 |
| 124 | 0.987224 | 0.551719 | -0.43551 | 0.4289 | 0.517731 | 0.960052 |
| 125 | 0.919878 | 0.590742 | -0.32914 | 0.436031 | 0.510368 | 0.988356 |
| 126 | 0.917433 | 0.490551 | -0.42688 | 0.428342 | 0.517857 | 0.957865 |
| 127 | 0.97633 | 0.612906 | -0.36342 | 0.434145 | 0.512528 | 0.9808 |
| 128 | 0.803087 | 0.684908 | -0.11818 | 0.450428 | 0.495711 | 1.04774 |
| 129 | 0.932982 | 0.692761 | -0.24022 | 0.442525 | 0.504113 | 1.01476 |
| 130 | 0.990556 | 0.681067 | -0.30949 | 0.438125 | 0.508702 | 0.996803 |
| 131 | 0.974139 | 0.617137 | -0.357 | 0.434592 | 0.512079 | 0.982586 |
| 132 | 0.985802 | 0.499189 | -0.48661 | 0.424831 | 0.521718 | 0.944216 |
| 133 | 0.937451 | 0.56278 | -0.37467 | 0.432817 | 0.513608 | 0.975512 |
| 134 | 0.987485 | 0.561586 | -0.4259 | 0.429635 | 0.517011 | 0.962936 |
| 135 | 0.923215 | 0.505613 | -0.4176 | 0.429214 | 0.517042 | 0.961284 |
| 136 | 0.986965 | 0.542034 | -0.44493 | 0.428164 | 0.518452 | 0.95717 |
| 137 | 0.961357 | 0.636079 | -0.32528 | 0.436747 | 0.509902 | 0.991236 |
| 138 | 0.858723 | 0.589883 | -0.26884 | 0.439875 | 0.506259 | 1.00391 |
| 139 | 0.972492 | 0.55289 | -0.4196 | 0.429891 | 0.516675 | 0.963944 |
| 140 | 0.98943 | 0.63651 | -0.35292 | 0.435046 | 0.511714 | 0.984402 |
| 141 | 0.970786 | 0.488825 | -0.48196 | 0.424896 | 0.521564 | 0.944466 |
| 142 | 0.986965 | 0.542034 | -0.44493 | 0.428164 | 0.518452 | 0.95717 |
| 143 | 0.936932 | 0.54188 | -0.39505 | 0.431239 | 0.515148 | 0.969257 |
| 144 | 0.932982 | 0.692761 | -0.24022 | 0.442525 | 0.504113 | 1.01476 |
| 145 | 0.923215 | 0.505613 | -0.4176 | 0.429214 | 0.517042 | 0.961284 |
| 146 | 0.98943 | 0.63651 | -0.35292 | 0.435046 | 0.511714 | 0.984402 |
| 147 | 0.937479 | 0.563894 | -0.37359 | 0.432905 | 0.513523 | 0.975859 |
| 148 | 0.937451 | 0.56278 | -0.37467 | 0.432817 | 0.513608 | 0.975512 |
| 149 | 0.986965 | 0.542034 | -0.44493 | 0.428164 | 0.518452 | 0.95717 |
| 150 | 0.806284 | 0.538028 | -0.26826 | 0.439345 | 0.506419 | 1.00175 |
| 151 | 0.973775 | 0.602736 | -0.37104 | 0.433562 | 0.513086 | 0.978475 |
| 152 | 0.892729 | 0.619301 | -0.27343 | 0.439864 | 0.506478 | 1.00387 |
| 153 | 0.937451 | 0.56278 | -0.37467 | 0.432817 | 0.513608 | 0.975512 |
| 154 | 0.937451 | 0.56278 | -0.37467 | 0.432817 | 0.513608 | 0.975512 |
| 155 | 0.919878 | 0.590742 | -0.32914 | 0.436031 | 0.510368 | 0.988356 |
| 156 | 0.936932 | 0.54188 | -0.39505 | 0.431239 | 0.515148 | 0.969257 |
| 157 | 0.937451 | 0.56278 | -0.37467 | 0.432817 | 0.513608 | 0.975512 |
| 158 | 0.932982 | 0.692761 | -0.24022 | 0.442525 | 0.504113 | 1.01476 |
| 159 | 0.937451 | 0.56278 | -0.37467 | 0.432817 | 0.513608 | 0.975512 |
| 160 | 1.14992 | 11.1961 | 10.0462 | 0.952453 | 0.010712 | 25.608 |
| 161 | 0.986965 | 0.542034 | -0.44493 | 0.428164 | 0.518452 | 0.95717 |
| 162 | 0.98943 | 0.63651 | -0.35292 | 0.435046 | 0.511714 | 0.984402 |
| 163 | 0.98943 | 0.63651 | -0.35292 | 0.435046 | 0.511714 | 0.984402 |
| 164 | 0.958991 | 0.541922 | -0.41707 | 0.429875 | 0.516613 | 0.963881 |
| 165 | 0.917433 | 0.490551 | -0.42688 | 0.428342 | 0.517857 | 0.957865 |
| 166 | 0.917433 | 0.490551 | -0.42688 | 0.428342 | 0.517857 | 0.957865 |
| 167 | 0.917433 | 0.490551 | -0.42688 | 0.428342 | 0.517857 | 0.957865 |
| 168 | 0.918956 | 0.552208 | -0.36675 | 0.433168 | 0.513155 | 0.976908 |
| 169 | 0.937451 | 0.56278 | -0.37467 | 0.432817 | 0.513608 | 0.975512 |
| 170 | 0.974294 | 0.582579 | -0.39172 | 0.432028 | 0.514591 | 0.972382 |
| 171 | 0.932982 | 0.692761 | -0.24022 | 0.442525 | 0.504113 | 1.01476 |
| 172 | 0.970786 | 0.488825 | -0.48196 | 0.424896 | 0.521564 | 0.944466 |
| 173 | 0.918493 | 0.533206 | -0.38529 | 0.431717 | 0.514568 | 0.971148 |
| 174 | 0.803087 | 0.684908 | -0.11818 | 0.450428 | 0.495711 | 1.04774 |
| 175 | 0.937451 | 0.56278 | -0.37467 | 0.432817 | 0.513608 | 0.975512 |
| 176 | 0.98943 | 0.63651 | -0.35292 | 0.435046 | 0.511714 | 0.984402 |
| 177 | 0.974139 | 0.617137 | -0.357 | 0.434592 | 0.512079 | 0.982586 |
| 178 | 0.767505 | 0.617417 | -0.15009 | 0.448009 | 0.497799 | 1.03754 |
| 179 | 0.936932 | 0.54188 | -0.39505 | 0.431239 | 0.515148 | 0.969257 |
| 180 | 0.987485 | 0.561586 | -0.4259 | 0.429635 | 0.517011 | 0.962936 |
| 181 | 0.98943 | 0.63651 | -0.35292 | 0.435046 | 0.511714 | 0.984402 |
| 182 | 0.918956 | 0.552208 | -0.36675 | 0.433168 | 0.513155 | 0.976908 |
| 183 | 0.937451 | 0.56278 | -0.37467 | 0.432817 | 0.513608 | 0.975512 |
| 184 | 0.987224 | 0.551719 | -0.43551 | 0.4289 | 0.517731 | 0.960052 |
| 185 | 0.937451 | 0.56278 | -0.37467 | 0.432817 | 0.513608 | 0.975512 |
| 186 | 0.987485 | 0.561586 | -0.4259 | 0.429635 | 0.517011 | 0.962936 |
| 187 | 0.97633 | 0.612906 | -0.36342 | 0.434145 | 0.512528 | 0.9808 |
| 188 | 0.923215 | 0.505613 | -0.4176 | 0.429214 | 0.517042 | 0.961284 |
| 189 | 0.98943 | 0.63651 | -0.35292 | 0.435046 | 0.511714 | 0.984402 |
| 190 | 0.986965 | 0.542034 | -0.44493 | 0.428164 | 0.518452 | 0.95717 |
| 191 | 0.918493 | 0.533206 | -0.38529 | 0.431717 | 0.514568 | 0.971148 |
| 192 | 0.972242 | 0.543285 | -0.42896 | 0.429169 | 0.517382 | 0.961107 |
| 193 | 0.937451 | 0.56278 | -0.37467 | 0.432817 | 0.513608 | 0.975512 |
| 194 | 0.919878 | 0.590742 | -0.32914 | 0.436031 | 0.510368 | 0.988356 |
| 195 | 0.918956 | 0.552208 | -0.36675 | 0.433168 | 0.513155 | 0.976908 |
| 196 | 0.974294 | 0.582579 | -0.39172 | 0.432028 | 0.514591 | 0.972382 |
| 197 | 0.932982 | 0.692761 | -0.24022 | 0.442525 | 0.504113 | 1.01476 |
| 198 | 0.990556 | 0.681067 | -0.30949 | 0.438125 | 0.508702 | 0.996803 |
| 199 | 0.972492 | 0.55289 | -0.4196 | 0.429891 | 0.516675 | 0.963944 |
| 200 | 0.986965 | 0.542034 | -0.44493 | 0.428164 | 0.518452 | 0.95717 |
| 201 | 0.917433 | 0.490551 | -0.42688 | 0.428342 | 0.517857 | 0.957865 |
| 202 | 0.987485 | 0.561586 | -0.4259 | 0.429635 | 0.517011 | 0.962936 |
| 203 | 0.936932 | 0.54188 | -0.39505 | 0.431239 | 0.515148 | 0.969257 |
| 204 | 0.987485 | 0.561586 | -0.4259 | 0.429635 | 0.517011 | 0.962936 |
| 205 | 0.974139 | 0.617137 | -0.357 | 0.434592 | 0.512079 | 0.982586 |
| 206 | 0.918493 | 0.533206 | -0.38529 | 0.431717 | 0.514568 | 0.971148 |
| 207 | 0.974139 | 0.617137 | -0.357 | 0.434592 | 0.512079 | 0.982586 |
| 208 | 0.987485 | 0.561586 | -0.4259 | 0.429635 | 0.517011 | 0.962936 |
| 209 | 0.937451 | 0.56278 | -0.37467 | 0.432817 | 0.513608 | 0.975512 |
| 210 | 0.937479 | 0.563894 | -0.37359 | 0.432905 | 0.513523 | 0.975859 |
| 211 | 0.973775 | 0.602736 | -0.37104 | 0.433562 | 0.513086 | 0.978475 |
| 212 | 0.803087 | 0.684908 | -0.11818 | 0.450428 | 0.495711 | 1.04774 |
| 213 | 0.936932 | 0.54188 | -0.39505 | 0.431239 | 0.515148 | 0.969257 |
| 214 | 0.991861 | 0.499837 | -0.49202 | 0.424517 | 0.522062 | 0.943004 |
| 215 | 0.970786 | 0.488825 | -0.48196 | 0.424896 | 0.521564 | 0.944466 |
| 216 | 0.803087 | 0.684908 | -0.11818 | 0.450428 | 0.495711 | 1.04774 |
| 217 | 0.918956 | 0.552208 | -0.36675 | 0.433168 | 0.513155 | 0.976908 |
| 218 | 0.961357 | 0.636079 | -0.32528 | 0.436747 | 0.509902 | 0.991236 |
| 219 | 0.923215 | 0.505613 | -0.4176 | 0.429214 | 0.517042 | 0.961284 |
| 220 | 0.923215 | 0.505613 | -0.4176 | 0.429214 | 0.517042 | 0.961284 |
| 221 | 0.936932 | 0.54188 | -0.39505 | 0.431239 | 0.515148 | 0.969257 |
| 222 | 0.869858 | 0.532409 | -0.33745 | 0.434745 | 0.511309 | 0.9832 |
| 223 | 0.919878 | 0.590742 | -0.32914 | 0.436031 | 0.510368 | 0.988356 |
| 224 | 0.806702 | 0.558688 | -0.24801 | 0.440928 | 0.504893 | 1.00821 |
| 225 | 0.932982 | 0.692761 | -0.24022 | 0.442525 | 0.504113 | 1.01476 |
| 226 | 0.932982 | 0.692761 | -0.24022 | 0.442525 | 0.504113 | 1.01476 |
| 227 | 0.973488 | 0.591491 | -0.382 | 0.432745 | 0.513885 | 0.975223 |
| 228 | 0.973775 | 0.602736 | -0.37104 | 0.433562 | 0.513086 | 0.978475 |
| 229 | 0.970786 | 0.488825 | -0.48196 | 0.424896 | 0.521564 | 0.944466 |
| 230 | 0.919878 | 0.590742 | -0.32914 | 0.436031 | 0.510368 | 0.988356 |
| 231 | 0.971993 | 0.533855 | -0.43814 | 0.428445 | 0.51809 | 0.95827 |
| 232 | 0.971993 | 0.533855 | -0.43814 | 0.428445 | 0.51809 | 0.95827 |
| 233 | 0.971993 | 0.533855 | -0.43814 | 0.428445 | 0.51809 | 0.95827 |
| 234 | 0.936932 | 0.54188 | -0.39505 | 0.431239 | 0.515148 | 0.969257 |
| 235 | 0.986965 | 0.542034 | -0.44493 | 0.428164 | 0.518452 | 0.95717 |
| 236 | 0.987485 | 0.561586 | -0.4259 | 0.429635 | 0.517011 | 0.962936 |
| 237 | 0.806702 | 0.558688 | -0.24801 | 0.440928 | 0.504893 | 1.00821 |
| 238 | 0.932982 | 0.692761 | -0.24022 | 0.442525 | 0.504113 | 1.01476 |
| 239 | 0.937451 | 0.56278 | -0.37467 | 0.432817 | 0.513608 | 0.975512 |
| 240 | 0.932982 | 0.692761 | -0.24022 | 0.442525 | 0.504113 | 1.01476 |
| 241 | 0.974294 | 0.582579 | -0.39172 | 0.432028 | 0.514591 | 0.972382 |
| 242 | 0.971993 | 0.533855 | -0.43814 | 0.428445 | 0.51809 | 0.95827 |
| 243 | 0.787123 | 0.486854 | -0.30027 | 0.436473 | 0.509055 | 0.990134 |
| 244 | 0.987409 | 0.558677 | -0.42873 | 0.429422 | 0.517219 | 0.962101 |
| 245 | 0.936932 | 0.54188 | -0.39505 | 0.431239 | 0.515148 | 0.969257 |
| 246 | 0.936932 | 0.54188 | -0.39505 | 0.431239 | 0.515148 | 0.969257 |
| 247 | 0.987485 | 0.561586 | -0.4259 | 0.429635 | 0.517011 | 0.962936 |
| 248 | 0.857884 | 0.551426 | -0.30646 | 0.437001 | 0.50904 | 0.992262 |
| 249 | 0.987485 | 0.561586 | -0.4259 | 0.429635 | 0.517011 | 0.962936 |
| 250 | 0.987409 | 0.558677 | -0.42873 | 0.429422 | 0.517219 | 0.962101 |
| 251 | 0.918724 | 0.542619 | -0.3761 | 0.432444 | 0.51386 | 0.974028 |
| 252 | 0.961357 | 0.636079 | -0.32528 | 0.436747 | 0.509902 | 0.991236 |
| 253 | 0.859272 | 0.615434 | -0.24384 | 0.441734 | 0.50446 | 1.01151 |
| 254 | 0.991861 | 0.499837 | -0.49202 | 0.424517 | 0.522062 | 0.943004 |
| 255 | 0.937451 | 0.56278 | -0.37467 | 0.432817 | 0.513608 | 0.975512 |
| 256 | 0.918493 | 0.533206 | -0.38529 | 0.431717 | 0.514568 | 0.971148 |
| 257 | 0.936932 | 0.54188 | -0.39505 | 0.431239 | 0.515148 | 0.969257 |
| 258 | 0.996136 | 0.661383 | -0.33475 | 0.436404 | 0.510416 | 0.989857 |
| 259 | 0.936932 | 0.54188 | -0.39505 | 0.431239 | 0.515148 | 0.969257 |
| 260 | 0.972492 | 0.55289 | -0.4196 | 0.429891 | 0.516675 | 0.963944 |
| 261 | 0.974294 | 0.582579 | -0.39172 | 0.432028 | 0.514591 | 0.972382 |
| 262 | 1.13198 | 5.90216 | 4.77018 | 0.828658 | 0.126106 | 6.18248 |
| 263 | 0.957872 | 0.499115 | -0.45876 | 0.426532 | 0.519881 | 0.950809 |
| 264 | 0.974294 | 0.582579 | -0.39172 | 0.432028 | 0.514591 | 0.972382 |
| 265 | 0.918956 | 0.552208 | -0.36675 | 0.433168 | 0.513155 | 0.976908 |
| 266 | 0.806702 | 0.558688 | -0.24801 | 0.440928 | 0.504893 | 1.00821 |
| 267 | 0.923215 | 0.505613 | -0.4176 | 0.429214 | 0.517042 | 0.961284 |
| 268 | 0.932982 | 0.692761 | -0.24022 | 0.442525 | 0.504113 | 1.01476 |
| 269 | 0.972492 | 0.55289 | -0.4196 | 0.429891 | 0.516675 | 0.963944 |
| 270 | 0.987485 | 0.561586 | -0.4259 | 0.429635 | 0.517011 | 0.962936 |
| 271 | 0.937451 | 0.56278 | -0.37467 | 0.432817 | 0.513608 | 0.975512 |
| 272 | 0.974294 | 0.582579 | -0.39172 | 0.432028 | 0.514591 | 0.972382 |
| 273 | 0.987409 | 0.558677 | -0.42873 | 0.429422 | 0.517219 | 0.962101 |
| 274 | 0.987224 | 0.551719 | -0.43551 | 0.4289 | 0.517731 | 0.960052 |
| 275 | 0.918956 | 0.552208 | -0.36675 | 0.433168 | 0.513155 | 0.976908 |
| 276 | 0.936932 | 0.54188 | -0.39505 | 0.431239 | 0.515148 | 0.969257 |
| 277 | 0.790468 | 0.659182 | -0.13129 | 0.449464 | 0.496557 | 1.04366 |
| 278 | 0.936932 | 0.54188 | -0.39505 | 0.431239 | 0.515148 | 0.969257 |
| 279 | 0.974294 | 0.582579 | -0.39172 | 0.432028 | 0.514591 | 0.972382 |
| 280 | 0.936932 | 0.54188 | -0.39505 | 0.431239 | 0.515148 | 0.969257 |
| 281 | 0.918724 | 0.542619 | -0.3761 | 0.432444 | 0.51386 | 0.974028 |
| 282 | 0.936932 | 0.54188 | -0.39505 | 0.431239 | 0.515148 | 0.969257 |
| 283 | 0.918724 | 0.542619 | -0.3761 | 0.432444 | 0.51386 | 0.974028 |
| 284 | 0.937451 | 0.56278 | -0.37467 | 0.432817 | 0.513608 | 0.975512 |
| 285 | 0.918724 | 0.542619 | -0.3761 | 0.432444 | 0.51386 | 0.974028 |
| 286 | 0.936932 | 0.54188 | -0.39505 | 0.431239 | 0.515148 | 0.969257 |
| 287 | 0.972242 | 0.543285 | -0.42896 | 0.429169 | 0.517382 | 0.961107 |
| 288 | 0.859272 | 0.615434 | -0.24384 | 0.441734 | 0.50446 | 1.01151 |
| 289 | 0.918956 | 0.552208 | -0.36675 | 0.433168 | 0.513155 | 0.976908 |
| 290 | 0.918724 | 0.542619 | -0.3761 | 0.432444 | 0.51386 | 0.974028 |
| 291 | 0.918956 | 0.552208 | -0.36675 | 0.433168 | 0.513155 | 0.976908 |
| 292 | 0.987224 | 0.551719 | -0.43551 | 0.4289 | 0.517731 | 0.960052 |
| 293 | 0.958991 | 0.541922 | -0.41707 | 0.429875 | 0.516613 | 0.963881 |
| 294 | 0.937479 | 0.563894 | -0.37359 | 0.432905 | 0.513523 | 0.975859 |
| 295 | 0.970786 | 0.488825 | -0.48196 | 0.424896 | 0.521564 | 0.944466 |
| 296 | 0.986965 | 0.542034 | -0.44493 | 0.428164 | 0.518452 | 0.95717 |
| 297 | 0.986965 | 0.542034 | -0.44493 | 0.428164 | 0.518452 | 0.95717 |
| 298 | 0.958991 | 0.541922 | -0.41707 | 0.429875 | 0.516613 | 0.963881 |
| 299 | 0.990556 | 0.681067 | -0.30949 | 0.438125 | 0.508702 | 0.996803 |
| 300 | 0.987409 | 0.558677 | -0.42873 | 0.429422 | 0.517219 | 0.962101 |
| 301 | 0.806284 | 0.538028 | -0.26826 | 0.439345 | 0.506419 | 1.00175 |
| 302 | 0.986965 | 0.542034 | -0.44493 | 0.428164 | 0.518452 | 0.95717 |
| 303 | 0.98943 | 0.63651 | -0.35292 | 0.435046 | 0.511714 | 0.984402 |
| 304 | 0.961357 | 0.636079 | -0.32528 | 0.436747 | 0.509902 | 0.991236 |
| 305 | 0.986965 | 0.542034 | -0.44493 | 0.428164 | 0.518452 | 0.95717 |
| 306 | 0.932982 | 0.692761 | -0.24022 | 0.442525 | 0.504113 | 1.01476 |
| 307 | 0.972492 | 0.55289 | -0.4196 | 0.429891 | 0.516675 | 0.963944 |
| 308 | 0.932982 | 0.692761 | -0.24022 | 0.442525 | 0.504113 | 1.01476 |
| 309 | 0.97633 | 0.612906 | -0.36342 | 0.434145 | 0.512528 | 0.9808 |
| 310 | 0.932982 | 0.692761 | -0.24022 | 0.442525 | 0.504113 | 1.01476 |
| 311 | 0.986965 | 0.542034 | -0.44493 | 0.428164 | 0.518452 | 0.95717 |
| 312 | 0.806284 | 0.538028 | -0.26826 | 0.439345 | 0.506419 | 1.00175 |
| 313 | 0.922331 | 0.696783 | -0.22555 | 0.443477 | 0.50313 | 1.01868 |
| 314 | 0.869858 | 0.532409 | -0.33745 | 0.434745 | 0.511309 | 0.9832 |
| 315 | 0.985802 | 0.499189 | -0.48661 | 0.424831 | 0.521718 | 0.944216 |
| 316 | 0.974294 | 0.582579 | -0.39172 | 0.432028 | 0.514591 | 0.972382 |
| 317 | 0.972492 | 0.55289 | -0.4196 | 0.429891 | 0.516675 | 0.963944 |
| 318 | 0.858965 | 0.601086 | -0.25788 | 0.440698 | 0.505462 | 1.00727 |
| 319 | 1.09164 | 5.45249 | 4.36085 | 0.819302 | 0.13571 | 5.79619 |
| 320 | 0.937451 | 0.56278 | -0.37467 | 0.432817 | 0.513608 | 0.975512 |
| 321 | 0.996136 | 0.661383 | -0.33475 | 0.436404 | 0.510416 | 0.989857 |
| 322 | 0.806702 | 0.558688 | -0.24801 | 0.440928 | 0.504893 | 1.00821 |
| 323 | 0.937451 | 0.56278 | -0.37467 | 0.432817 | 0.513608 | 0.975512 |
| 324 | 0.986965 | 0.542034 | -0.44493 | 0.428164 | 0.518452 | 0.95717 |
| 325 | 0.919878 | 0.590742 | -0.32914 | 0.436031 | 0.510368 | 0.988356 |
| 326 | 0.973488 | 0.591491 | -0.382 | 0.432745 | 0.513885 | 0.975223 |
| 327 | 0.918724 | 0.542619 | -0.3761 | 0.432444 | 0.51386 | 0.974028 |
| 328 | 0.990556 | 0.681067 | -0.30949 | 0.438125 | 0.508702 | 0.996803 |
| 329 | 0.857464 | 0.532463 | -0.325 | 0.435545 | 0.510451 | 0.986402 |
| 330 | 0.936932 | 0.541886 | -0.39505 | 0.431239 | 0.515147 | 0.969258 |
| 331 | 0.806702 | 0.558688 | -0.24801 | 0.440928 | 0.504893 | 1.00821 |
| 332 | 0.973488 | 0.591491 | -0.382 | 0.432745 | 0.513885 | 0.975223 |
| 333 | 0.803087 | 0.684908 | -0.11818 | 0.450428 | 0.495711 | 1.04774 |
| 334 | 0.987485 | 0.561586 | -0.4259 | 0.429635 | 0.517011 | 0.962936 |
| 335 | 0.987485 | 0.561586 | -0.4259 | 0.429635 | 0.517011 | 0.962936 |
| 336 | 0.932982 | 0.692761 | -0.24022 | 0.442525 | 0.504113 | 1.01476 |
| 337 | 0.958316 | 0.515962 | -0.44235 | 0.427866 | 0.518577 | 0.956005 |
| 338 | 0.889845 | 0.592529 | -0.29732 | 0.438067 | 0.508207 | 0.996567 |
| 339 | 0.957872 | 0.49912 | -0.45875 | 0.426532 | 0.519881 | 0.95081 |
| 340 | 0.937451 | 0.56278 | -0.37467 | 0.432817 | 0.513608 | 0.975512 |
| 341 | 0.986965 | 0.542034 | -0.44493 | 0.428164 | 0.518452 | 0.95717 |
| 342 | 0.937451 | 0.56278 | -0.37467 | 0.432817 | 0.513608 | 0.975512 |
| 343 | 0.974294 | 0.582579 | -0.39172 | 0.432028 | 0.514591 | 0.972382 |
| 344 | 0.987224 | 0.551719 | -0.43551 | 0.4289 | 0.517731 | 0.960052 |
| 345 | 0.973488 | 0.591491 | -0.382 | 0.432745 | 0.513885 | 0.975223 |
| 346 | 0.917433 | 0.490551 | -0.42688 | 0.428342 | 0.517857 | 0.957865 |
| 347 | 0.97633 | 0.612906 | -0.36342 | 0.434145 | 0.512528 | 0.9808 |
| 348 | 0.991861 | 0.499837 | -0.49202 | 0.424517 | 0.522062 | 0.943004 |
| 349 | 0.971005 | 0.496857 | -0.47415 | 0.425546 | 0.520927 | 0.946984 |
| 350 | 0.96093 | 0.618709 | -0.34222 | 0.435524 | 0.511096 | 0.986318 |
| 351 | 0.857464 | 0.532463 | -0.325 | 0.435545 | 0.510451 | 0.986402 |
| 352 | 0.937451 | 0.56278 | -0.37467 | 0.432817 | 0.513608 | 0.975512 |
| 353 | 0.974139 | 0.617137 | -0.357 | 0.434592 | 0.512079 | 0.982586 |
| 354 | 0.991861 | 0.499837 | -0.49202 | 0.424517 | 0.522062 | 0.943004 |
| 355 | 0.937451 | 0.56278 | -0.37467 | 0.432817 | 0.513608 | 0.975512 |
| 356 | 0.788397 | 0.550531 | -0.23787 | 0.441528 | 0.504185 | 1.01067 |
| 357 | 0.986965 | 0.542034 | -0.44493 | 0.428164 | 0.518452 | 0.95717 |
| 358 | 0.974294 | 0.582579 | -0.39172 | 0.432028 | 0.514591 | 0.972382 |
| 359 | 0.970786 | 0.488825 | -0.48196 | 0.424896 | 0.521564 | 0.944466 |
| 360 | 0.98943 | 0.63651 | -0.35292 | 0.435046 | 0.511714 | 0.984402 |
| 361 | 0.806284 | 0.538028 | -0.26826 | 0.439345 | 0.506419 | 1.00175 |
| 362 | 0.806725 | 0.559789 | -0.24694 | 0.441016 | 0.504808 | 1.00857 |
| 363 | 0.936932 | 0.54188 | -0.39505 | 0.431239 | 0.515148 | 0.969257 |
| 364 | 0.918956 | 0.552208 | -0.36675 | 0.433168 | 0.513155 | 0.976908 |
| 365 | 0.923215 | 0.505613 | -0.4176 | 0.429214 | 0.517042 | 0.961284 |
| 366 | 0.98943 | 0.63651 | -0.35292 | 0.435046 | 0.511714 | 0.984402 |
| 367 | 0.991861 | 0.499837 | -0.49202 | 0.424517 | 0.522062 | 0.943004 |
| 368 | 0.937451 | 0.56278 | -0.37467 | 0.432817 | 0.513608 | 0.975512 |
| 369 | 0.958316 | 0.515962 | -0.44235 | 0.427866 | 0.518577 | 0.956005 |
| 370 | 0.986965 | 0.542034 | -0.44493 | 0.428164 | 0.518452 | 0.95717 |
| 371 | 0.803087 | 0.684908 | -0.11818 | 0.450428 | 0.495711 | 1.04774 |
| 372 | 0.974294 | 0.582579 | -0.39172 | 0.432028 | 0.514591 | 0.972382 |
| 373 | 0.937451 | 0.56278 | -0.37467 | 0.432817 | 0.513608 | 0.975512 |
| 374 | 0.892729 | 0.619301 | -0.27343 | 0.439864 | 0.506478 | 1.00387 |
| 375 | 0.973488 | 0.591491 | -0.382 | 0.432745 | 0.513885 | 0.975223 |
| 376 | 0.923215 | 0.505613 | -0.4176 | 0.429214 | 0.517042 | 0.961284 |
| 377 | 0.936932 | 0.54188 | -0.39505 | 0.431239 | 0.515148 | 0.969257 |
| 378 | 0.932982 | 0.692761 | -0.24022 | 0.442525 | 0.504113 | 1.01476 |
| 379 | 0.937451 | 0.56278 | -0.37467 | 0.432817 | 0.513608 | 0.975512 |
| 380 | 0.986965 | 0.542034 | -0.44493 | 0.428164 | 0.518452 | 0.95717 |
| 381 | 0.937451 | 0.56278 | -0.37467 | 0.432817 | 0.513608 | 0.975512 |
| 382 | 0.98943 | 0.63651 | -0.35292 | 0.435046 | 0.511714 | 0.984402 |
| 383 | 0.917433 | 0.490551 | -0.42688 | 0.428342 | 0.517857 | 0.957865 |
| 384 | 0.98943 | 0.63651 | -0.35292 | 0.435046 | 0.511714 | 0.984402 |
| 385 | 0.970786 | 0.488825 | -0.48196 | 0.424896 | 0.521564 | 0.944466 |
| 386 | 0.970786 | 0.488825 | -0.48196 | 0.424896 | 0.521564 | 0.944466 |
| 387 | 0.971993 | 0.533855 | -0.43814 | 0.428445 | 0.51809 | 0.95827 |
| 388 | 0.857884 | 0.551426 | -0.30646 | 0.437001 | 0.50904 | 0.992262 |
| 389 | 0.987224 | 0.551719 | -0.43551 | 0.4289 | 0.517731 | 0.960052 |
| 390 | 0.973775 | 0.602736 | -0.37104 | 0.433562 | 0.513086 | 0.978475 |
| 391 | 0.858723 | 0.589883 | -0.26884 | 0.439875 | 0.506259 | 1.00391 |
| 392 | 0.986965 | 0.542034 | -0.44493 | 0.428164 | 0.518452 | 0.95717 |
| 393 | 0.971993 | 0.533855 | -0.43814 | 0.428445 | 0.51809 | 0.95827 |
| 394 | 0.932982 | 0.692761 | -0.24022 | 0.442525 | 0.504113 | 1.01476 |
| 395 | 0.937479 | 0.563894 | -0.37359 | 0.432905 | 0.513523 | 0.975859 |
| 396 | 0.98943 | 0.63651 | -0.35292 | 0.435046 | 0.511714 | 0.984402 |
| 397 | 0.922331 | 0.696783 | -0.22555 | 0.443477 | 0.50313 | 1.01868 |
| 398 | 0.932982 | 0.692761 | -0.24022 | 0.442525 | 0.504113 | 1.01476 |
| 399 | 0.937451 | 0.56278 | -0.37467 | 0.432817 | 0.513608 | 0.975512 |
| 400 | 0.972492 | 0.55289 | -0.4196 | 0.429891 | 0.516675 | 0.963944 |
| 401 | 0.990556 | 0.681067 | -0.30949 | 0.438125 | 0.508702 | 0.996803 |
| 402 | 0.918956 | 0.552208 | -0.36675 | 0.433168 | 0.513155 | 0.976908 |
| 403 | 0.923215 | 0.505613 | -0.4176 | 0.429214 | 0.517042 | 0.961284 |
| 404 | 0.958093 | 0.507466 | -0.45063 | 0.4272 | 0.519228 | 0.95341 |
| 405 | 0.857673 | 0.541857 | -0.31582 | 0.436274 | 0.509744 | 0.989332 |
| 406 | 0.987485 | 0.561586 | -0.4259 | 0.429635 | 0.517011 | 0.962936 |
| 407 | 0.987485 | 0.561586 | -0.4259 | 0.429635 | 0.517011 | 0.962936 |
| 408 | 0.923215 | 0.505613 | -0.4176 | 0.429214 | 0.517042 | 0.961284 |
| 409 | 0.973488 | 0.591491 | -0.382 | 0.432745 | 0.513885 | 0.975223 |
| 410 | 0.986965 | 0.542034 | -0.44493 | 0.428164 | 0.518452 | 0.95717 |
| 411 | 0.918493 | 0.533206 | -0.38529 | 0.431717 | 0.514568 | 0.971148 |
| 412 | 0.857673 | 0.541857 | -0.31582 | 0.436274 | 0.509744 | 0.989332 |
| 413 | 0.806702 | 0.558688 | -0.24801 | 0.440928 | 0.504893 | 1.00821 |
| 414 | 0.919878 | 0.590742 | -0.32914 | 0.436031 | 0.510368 | 0.988356 |
| 415 | 0.918956 | 0.552208 | -0.36675 | 0.433168 | 0.513155 | 0.976908 |
| 416 | 0.974294 | 0.582579 | -0.39172 | 0.432028 | 0.514591 | 0.972382 |
| 417 | 0.932982 | 0.692761 | -0.24022 | 0.442525 | 0.504113 | 1.01476 |
| 418 | 0.990556 | 0.681067 | -0.30949 | 0.438125 | 0.508702 | 0.996803 |
| 419 | 0.788397 | 0.550531 | -0.23787 | 0.441528 | 0.504185 | 1.01067 |
| 420 | 0.986965 | 0.542034 | -0.44493 | 0.428164 | 0.518452 | 0.95717 |
| 421 | 0.970786 | 0.488825 | -0.48196 | 0.424896 | 0.521564 | 0.944466 |
| 422 | 0.987485 | 0.561586 | -0.4259 | 0.429635 | 0.517011 | 0.962936 |
| 423 | 0.936932 | 0.54188 | -0.39505 | 0.431239 | 0.515148 | 0.969257 |
| 424 | 0.972492 | 0.55289 | -0.4196 | 0.429891 | 0.516675 | 0.963944 |
| 425 | 0.974139 | 0.617137 | -0.357 | 0.434592 | 0.512079 | 0.982586 |
| 426 | 0.937451 | 0.56278 | -0.37467 | 0.432817 | 0.513608 | 0.975512 |
| 427 | 0.806702 | 0.558688 | -0.24801 | 0.440928 | 0.504893 | 1.00821 |
| 428 | 0.936932 | 0.54188 | -0.39505 | 0.431239 | 0.515148 | 0.969257 |
| 429 | 0.937451 | 0.56278 | -0.37467 | 0.432817 | 0.513608 | 0.975512 |
| 430 | 0.986965 | 0.542034 | -0.44493 | 0.428164 | 0.518452 | 0.95717 |
| 431 | 0.974294 | 0.582579 | -0.39172 | 0.432028 | 0.514591 | 0.972382 |
| 432 | 0.869858 | 0.532409 | -0.33745 | 0.434745 | 0.511309 | 0.9832 |
| 433 | 0.986965 | 0.542034 | -0.44493 | 0.428164 | 0.518452 | 0.95717 |
| 434 | 0.919878 | 0.590742 | -0.32914 | 0.436031 | 0.510368 | 0.988356 |
| 435 | 0.917433 | 0.490551 | -0.42688 | 0.428342 | 0.517857 | 0.957865 |
| 436 | 0.932982 | 0.692761 | -0.24022 | 0.442525 | 0.504113 | 1.01476 |
| 437 | 0.932982 | 0.692761 | -0.24022 | 0.442525 | 0.504113 | 1.01476 |
| 438 | 0.98943 | 0.63651 | -0.35292 | 0.435046 | 0.511714 | 0.984402 |
| 439 | 0.923215 | 0.505613 | -0.4176 | 0.429214 | 0.517042 | 0.961284 |
| 440 | 0.937451 | 0.56278 | -0.37467 | 0.432817 | 0.513608 | 0.975512 |
| 441 | 0.936932 | 0.54188 | -0.39505 | 0.431239 | 0.515148 | 0.969257 |
| 442 | 0.987485 | 0.561586 | -0.4259 | 0.429635 | 0.517011 | 0.962936 |
| 443 | 0.789141 | 0.5889 | -0.20024 | 0.444412 | 0.50141 | 1.02255 |
| 444 | 0.806702 | 0.558688 | -0.24801 | 0.440928 | 0.504893 | 1.00821 |
| 445 | 0.932982 | 0.692761 | -0.24022 | 0.442525 | 0.504113 | 1.01476 |
| 446 | 0.974294 | 0.582579 | -0.39172 | 0.432028 | 0.514591 | 0.972382 |
| 447 | 0.919878 | 0.590742 | -0.32914 | 0.436031 | 0.510368 | 0.988356 |
| 448 | 0.919878 | 0.590742 | -0.32914 | 0.436031 | 0.510368 | 0.988356 |
| 449 | 0.787123 | 0.486854 | -0.30027 | 0.436473 | 0.509055 | 0.990134 |
| 450 | 0.973488 | 0.591491 | -0.382 | 0.432745 | 0.513885 | 0.975223 |
| 451 | 0.987485 | 0.561586 | -0.4259 | 0.429635 | 0.517011 | 0.962936 |
| 452 | 0.932982 | 0.692761 | -0.24022 | 0.442525 | 0.504113 | 1.01476 |
| 453 | 0.971993 | 0.533855 | -0.43814 | 0.428445 | 0.51809 | 0.95827 |
| 454 | 0.788397 | 0.550531 | -0.23787 | 0.441528 | 0.504185 | 1.01067 |
| 455 | 0.806284 | 0.538028 | -0.26826 | 0.439345 | 0.506419 | 1.00175 |
| 456 | 0.937451 | 0.56278 | -0.37467 | 0.432817 | 0.513608 | 0.975512 |
| 457 | 0.985802 | 0.499189 | -0.48661 | 0.424831 | 0.521718 | 0.944216 |
| 458 | 0.932982 | 0.692761 | -0.24022 | 0.442525 | 0.504113 | 1.01476 |
| 459 | 0.937451 | 0.56278 | -0.37467 | 0.432817 | 0.513608 | 0.975512 |
| 460 | 0.932982 | 0.692761 | -0.24022 | 0.442525 | 0.504113 | 1.01476 |
| 461 | 0.932982 | 0.692761 | -0.24022 | 0.442525 | 0.504113 | 1.01476 |
| 462 | 0.971993 | 0.533855 | -0.43814 | 0.428445 | 0.51809 | 0.95827 |
| 463 | 0.970786 | 0.488825 | -0.48196 | 0.424896 | 0.521564 | 0.944466 |
| 464 | 0.987409 | 0.558677 | -0.42873 | 0.429422 | 0.517219 | 0.962101 |
| 465 | 0.972492 | 0.55289 | -0.4196 | 0.429891 | 0.516675 | 0.963944 |
| 466 | 0.936932 | 0.54188 | -0.39505 | 0.431239 | 0.515148 | 0.969257 |
| 467 | 0.987485 | 0.561586 | -0.4259 | 0.429635 | 0.517011 | 0.962936 |
| 468 | 0.936932 | 0.54188 | -0.39505 | 0.431239 | 0.515148 | 0.969257 |
| 469 | 0.936932 | 0.54188 | -0.39505 | 0.431239 | 0.515148 | 0.969257 |
| 470 | 0.987409 | 0.558677 | -0.42873 | 0.429422 | 0.517219 | 0.962101 |
| 471 | 0.892729 | 0.619301 | -0.27343 | 0.439864 | 0.506478 | 1.00387 |
| 472 | 0.869858 | 0.532409 | -0.33745 | 0.434745 | 0.511309 | 0.9832 |
| 473 | 0.973488 | 0.591491 | -0.382 | 0.432745 | 0.513885 | 0.975223 |
| 474 | 0.97633 | 0.612906 | -0.36342 | 0.434145 | 0.512528 | 0.9808 |
| 475 | 0.937451 | 0.56278 | -0.37467 | 0.432817 | 0.513608 | 0.975512 |
| 476 | 0.987409 | 0.558677 | -0.42873 | 0.429422 | 0.517219 | 0.962101 |
| 477 | 0.936932 | 0.54188 | -0.39505 | 0.431239 | 0.515148 | 0.969257 |
| 478 | 0.996136 | 0.661383 | -0.33475 | 0.436404 | 0.510416 | 0.989857 |
| 479 | 0.987485 | 0.561586 | -0.4259 | 0.429635 | 0.517011 | 0.962936 |
| 480 | 0.986965 | 0.542034 | -0.44493 | 0.428164 | 0.518452 | 0.95717 |
| 481 | 0.932982 | 0.692761 | -0.24022 | 0.442525 | 0.504113 | 1.01476 |
| 482 | 0.918956 | 0.552208 | -0.36675 | 0.433168 | 0.513155 | 0.976908 |
| 483 | 0.987485 | 0.561586 | -0.4259 | 0.429635 | 0.517011 | 0.962936 |
| 484 | 0.974294 | 0.582579 | -0.39172 | 0.432028 | 0.514591 | 0.972382 |
| 485 | 0.972492 | 0.55289 | -0.4196 | 0.429891 | 0.516675 | 0.963944 |
| 486 | 0.937451 | 0.56278 | -0.37467 | 0.432817 | 0.513608 | 0.975512 |
| 487 | 0.974294 | 0.582579 | -0.39172 | 0.432028 | 0.514591 | 0.972382 |
| 488 | 0.973775 | 0.602736 | -0.37104 | 0.433562 | 0.513086 | 0.978475 |
| 489 | 0.788397 | 0.550531 | -0.23787 | 0.441528 | 0.504185 | 1.01067 |
| 490 | 0.986965 | 0.542034 | -0.44493 | 0.428164 | 0.518452 | 0.95717 |
| 491 | 0.806702 | 0.558688 | -0.24801 | 0.440928 | 0.504893 | 1.00821 |
| 492 | 0.974294 | 0.582579 | -0.39172 | 0.432028 | 0.514591 | 0.972382 |
| 493 | 0.987409 | 0.558677 | -0.42873 | 0.429422 | 0.517219 | 0.962101 |
| 494 | 0.987485 | 0.561586 | -0.4259 | 0.429635 | 0.517011 | 0.962936 |
| 495 | 0.788397 | 0.550531 | -0.23787 | 0.441528 | 0.504185 | 1.01067 |
| 496 | 0.917433 | 0.490551 | -0.42688 | 0.428342 | 0.517857 | 0.957865 |
| 497 | 0.922331 | 0.696783 | -0.22555 | 0.443477 | 0.50313 | 1.01868 |
| 498 | 0.919878 | 0.590742 | -0.32914 | 0.436031 | 0.510368 | 0.988356 |
| 499 | 0.936932 | 0.54188 | -0.39505 | 0.431239 | 0.515148 | 0.969257 |
| 500 | 0.932982 | 0.692761 | -0.24022 | 0.442525 | 0.504113 | 1.01476 |
| 501 | 0.918724 | 0.542619 | -0.3761 | 0.432444 | 0.51386 | 0.974028 |
| 502 | 0.937451 | 0.56278 | -0.37467 | 0.432817 | 0.513608 | 0.975512 |
| 503 | 0.972242 | 0.543285 | -0.42896 | 0.429169 | 0.517382 | 0.961107 |
| 504 | 0.936932 | 0.54188 | -0.39505 | 0.431239 | 0.515148 | 0.969257 |
| 505 | 0.806284 | 0.538028 | -0.26826 | 0.439345 | 0.506419 | 1.00175 |
| 506 | 0.987485 | 0.561586 | -0.4259 | 0.429635 | 0.517011 | 0.962936 |
| 507 | 0.972242 | 0.543285 | -0.42896 | 0.429169 | 0.517382 | 0.961107 |
| 508 | 0.936932 | 0.54188 | -0.39505 | 0.431239 | 0.515148 | 0.969257 |
| 509 | 0.918724 | 0.542619 | -0.3761 | 0.432444 | 0.51386 | 0.974028 |
| 510 | 0.919878 | 0.590742 | -0.32914 | 0.436031 | 0.510368 | 0.988356 |
| 511 | 0.936932 | 0.54188 | -0.39505 | 0.431239 | 0.515148 | 0.969257 |
| 512 | 0.937451 | 0.56278 | -0.37467 | 0.432817 | 0.513608 | 0.975512 |
| 513 | 0.918724 | 0.542619 | -0.3761 | 0.432444 | 0.51386 | 0.974028 |
| 514 | 0.987485 | 0.561586 | -0.4259 | 0.429635 | 0.517011 | 0.962936 |
| 515 | 0.918724 | 0.542619 | -0.3761 | 0.432444 | 0.51386 | 0.974028 |
| 516 | 0.806284 | 0.538028 | -0.26826 | 0.439345 | 0.506419 | 1.00175 |
| 517 | 0.936932 | 0.54188 | -0.39505 | 0.431239 | 0.515148 | 0.969257 |
| 518 | 0.987485 | 0.561586 | -0.4259 | 0.429635 | 0.517011 | 0.962936 |
| 519 | 0.972242 | 0.543285 | -0.42896 | 0.429169 | 0.517382 | 0.961107 |
| 520 | 0.987485 | 0.561586 | -0.4259 | 0.429635 | 0.517011 | 0.962936 |
| 521 | 0.918724 | 0.542619 | -0.3761 | 0.432444 | 0.51386 | 0.974028 |
| 522 | 0.789141 | 0.5889 | -0.20024 | 0.444412 | 0.50141 | 1.02255 |
| 523 | 0.936932 | 0.54188 | -0.39505 | 0.431239 | 0.515148 | 0.969257 |
| 524 | 0.937451 | 0.56278 | -0.37467 | 0.432817 | 0.513608 | 0.975512 |
| 525 | 0.918724 | 0.542619 | -0.3761 | 0.432444 | 0.51386 | 0.974028 |
| 526 | 1.11384 | 5.84402 | 4.73018 | 0.828539 | 0.126594 | 6.17728 |
| 527 | 0.918724 | 0.542619 | -0.3761 | 0.432444 | 0.51386 | 0.974028 |
| 528 | 0.936932 | 0.54188 | -0.39505 | 0.431239 | 0.515148 | 0.969257 |
| 529 | 0.936932 | 0.54188 | -0.39505 | 0.431239 | 0.515148 | 0.969257 |
| 530 | 0.987485 | 0.561586 | -0.4259 | 0.429635 | 0.517011 | 0.962936 |
| 531 | 0.972242 | 0.543285 | -0.42896 | 0.429169 | 0.517382 | 0.961107 |
| 532 | 0.987485 | 0.561586 | -0.4259 | 0.429635 | 0.517011 | 0.962936 |
| 533 | 0.918724 | 0.542619 | -0.3761 | 0.432444 | 0.51386 | 0.974028 |
| 534 | 9.8504 | 0.724287 | -9.12612 | 0.011654 | 0.966914 | 0.015073 |
| 535 | 0.938008 | 0.544626 | -0.39338 | 0.431385 | 0.515011 | 0.969836 |
| 536 | 0.938522 | 0.565306 | -0.37322 | 0.432943 | 0.513492 | 0.976012 |
| 537 | 0.920106 | 0.545353 | -0.37475 | 0.43257 | 0.513746 | 0.974528 |
| 538 | 0.972943 | 0.555521 | -0.41742 | 0.430065 | 0.516507 | 0.964629 |
| 539 | 0.920106 | 0.545353 | -0.37475 | 0.43257 | 0.513746 | 0.974528 |
| 540 | 0.938008 | 0.544626 | -0.39338 | 0.431385 | 0.515011 | 0.969836 |
| 541 | 0.938008 | 0.544626 | -0.39338 | 0.431385 | 0.515011 | 0.969836 |
| 542 | 0.987665 | 0.564125 | -0.42354 | 0.429817 | 0.516833 | 0.963652 |
| 543 | 0.972696 | 0.546012 | -0.42668 | 0.429352 | 0.517204 | 0.961826 |
| 544 | 0.987665 | 0.564125 | -0.42354 | 0.429817 | 0.516833 | 0.963652 |
| 545 | 0.920106 | 0.545353 | -0.37475 | 0.43257 | 0.513746 | 0.974528 |
| 546 | 0.789183 | 0.591059 | -0.19812 | 0.444572 | 0.501257 | 1.02321 |
| 547 | 0.936932 | 0.54188 | -0.39505 | 0.431239 | 0.515148 | 0.969257 |
| 548 | 0.937451 | 0.56278 | -0.37467 | 0.432817 | 0.513608 | 0.975512 |
| 549 | 0.918724 | 0.542619 | -0.3761 | 0.432444 | 0.51386 | 0.974028 |
| 550 | 0.936932 | 0.54188 | -0.39505 | 0.431239 | 0.515148 | 0.969257 |
| 551 | 0.918724 | 0.542619 | -0.3761 | 0.432444 | 0.51386 | 0.974028 |
| 552 | 0.936932 | 0.54188 | -0.39505 | 0.431239 | 0.515148 | 0.969257 |
| 553 | 0.806702 | 0.558688 | -0.24801 | 0.440928 | 0.504893 | 1.00821 |
| 554 | 0.987485 | 0.561586 | -0.4259 | 0.429635 | 0.517011 | 0.962936 |
| 555 | 0.972242 | 0.543285 | -0.42896 | 0.429169 | 0.517382 | 0.961107 |
| 556 | 0.987409 | 0.558677 | -0.42873 | 0.429422 | 0.517219 | 0.962101 |
| 557 | 0.974294 | 0.582579 | -0.39172 | 0.432028 | 0.514591 | 0.972382 |
| 558 | 0.958316 | 0.515962 | -0.44235 | 0.427866 | 0.518577 | 0.956005 |
| 559 | 0.991861 | 0.499837 | -0.49202 | 0.424517 | 0.522062 | 0.943004 |
| 560 | 0.936932 | 0.54188 | -0.39505 | 0.431239 | 0.515148 | 0.969257 |
| 561 | 0.986965 | 0.542034 | -0.44493 | 0.428164 | 0.518452 | 0.95717 |
| 562 | 0.958991 | 0.541922 | -0.41707 | 0.429875 | 0.516613 | 0.963881 |
| 563 | 0.98943 | 0.63651 | -0.35292 | 0.435046 | 0.511714 | 0.984402 |
| 564 | 0.987409 | 0.558677 | -0.42873 | 0.429422 | 0.517219 | 0.962101 |
| 565 | 0.937451 | 0.56278 | -0.37467 | 0.432817 | 0.513608 | 0.975512 |
| 566 | 0.986965 | 0.542034 | -0.44493 | 0.428164 | 0.518452 | 0.95717 |
| 567 | 0.990556 | 0.681067 | -0.30949 | 0.438125 | 0.508702 | 0.996803 |
| 568 | 0.98943 | 0.63651 | -0.35292 | 0.435046 | 0.511714 | 0.984402 |
| 569 | 0.986965 | 0.542034 | -0.44493 | 0.428164 | 0.518452 | 0.95717 |
| 570 | 0.889845 | 0.592529 | -0.29732 | 0.438067 | 0.508207 | 0.996567 |
| 571 | 0.972492 | 0.55289 | -0.4196 | 0.429891 | 0.516675 | 0.963944 |
| 572 | 0.932982 | 0.692761 | -0.24022 | 0.442525 | 0.504113 | 1.01476 |
| 573 | 0.973488 | 0.591491 | -0.382 | 0.432745 | 0.513885 | 0.975223 |
| 574 | 0.932982 | 0.692761 | -0.24022 | 0.442525 | 0.504113 | 1.01476 |
| 575 | 0.986965 | 0.542034 | -0.44493 | 0.428164 | 0.518452 | 0.95717 |
| 576 | 0.936932 | 0.54188 | -0.39505 | 0.431239 | 0.515148 | 0.969257 |
| 577 | 0.922331 | 0.696783 | -0.22555 | 0.443477 | 0.50313 | 1.01868 |
| 578 | 0.971993 | 0.533855 | -0.43814 | 0.428445 | 0.51809 | 0.95827 |
| 579 | 0.985802 | 0.499189 | -0.48661 | 0.424831 | 0.521718 | 0.944216 |
| 580 | 0.974294 | 0.582579 | -0.39172 | 0.432028 | 0.514591 | 0.972382 |
| 581 | 0.974139 | 0.617137 | -0.357 | 0.434592 | 0.512079 | 0.982586 |
| 582 | 0.986965 | 0.542034 | -0.44493 | 0.428164 | 0.518452 | 0.95717 |
| 583 | 0.803087 | 0.684908 | -0.11818 | 0.450428 | 0.495711 | 1.04774 |
| 584 | 1.11934 | 6.02765 | 4.90831 | 0.833162 | 0.122132 | 6.38387 |
| 585 | 0.996136 | 0.661383 | -0.33475 | 0.436404 | 0.510416 | 0.989857 |
| 586 | 0.923215 | 0.505613 | -0.4176 | 0.429214 | 0.517042 | 0.961284 |
| 587 | 0.958316 | 0.515962 | -0.44235 | 0.427866 | 0.518577 | 0.956005 |
| 588 | 0.936932 | 0.54188 | -0.39505 | 0.431239 | 0.515148 | 0.969257 |
| 589 | 0.973488 | 0.591491 | -0.382 | 0.432745 | 0.513885 | 0.975223 |
| 590 | 0.97633 | 0.612906 | -0.36342 | 0.434145 | 0.512528 | 0.9808 |
| 591 | 0.918724 | 0.542619 | -0.3761 | 0.432444 | 0.51386 | 0.974028 |
| 592 | 0.990556 | 0.681067 | -0.30949 | 0.438125 | 0.508702 | 0.996803 |
| 593 | 0.974139 | 0.617137 | -0.357 | 0.434592 | 0.512079 | 0.982586 |
| 594 | 0.803087 | 0.684908 | -0.11818 | 0.450428 | 0.495711 | 1.04774 |
| 595 | 0.936932 | 0.54188 | -0.39505 | 0.431239 | 0.515148 | 0.969257 |
| 596 | 0.97633 | 0.612906 | -0.36342 | 0.434145 | 0.512528 | 0.9808 |
| 597 | 0.97633 | 0.612906 | -0.36342 | 0.434145 | 0.512528 | 0.9808 |
| 598 | 0.937451 | 0.56278 | -0.37467 | 0.432817 | 0.513608 | 0.975512 |
| 599 | 0.923215 | 0.505613 | -0.4176 | 0.429214 | 0.517042 | 0.961284 |
| 600 | 0.974294 | 0.582579 | -0.39172 | 0.432028 | 0.514591 | 0.972382 |
| 601 | 0.987485 | 0.561586 | -0.4259 | 0.429635 | 0.517011 | 0.962936 |
| 602 | 0.974294 | 0.582579 | -0.39172 | 0.432028 | 0.514591 | 0.972382 |
| 603 | 0.985802 | 0.499189 | -0.48661 | 0.424831 | 0.521718 | 0.944216 |
| 604 | 0.98943 | 0.63651 | -0.35292 | 0.435046 | 0.511714 | 0.984402 |
| 605 | 0.937451 | 0.56278 | -0.37467 | 0.432817 | 0.513608 | 0.975512 |
| 606 | 0.987485 | 0.561586 | -0.4259 | 0.429635 | 0.517011 | 0.962936 |
| 607 | 0.789141 | 0.5889 | -0.20024 | 0.444412 | 0.50141 | 1.02255 |
| 608 | 0.987224 | 0.551719 | -0.43551 | 0.4289 | 0.517731 | 0.960052 |
| 609 | 0.991861 | 0.499837 | -0.49202 | 0.424517 | 0.522062 | 0.943004 |
| 610 | 0.970786 | 0.488825 | -0.48196 | 0.424896 | 0.521564 | 0.944466 |
| 611 | 0.973488 | 0.591491 | -0.382 | 0.432745 | 0.513885 | 0.975223 |
| 612 | 0.803087 | 0.684908 | -0.11818 | 0.450428 | 0.495711 | 1.04774 |
| 613 | 0.973775 | 0.602736 | -0.37104 | 0.433562 | 0.513086 | 0.978475 |
| 614 | 0.96093 | 0.618709 | -0.34222 | 0.435524 | 0.511096 | 0.986318 |
| 615 | 0.974139 | 0.617137 | -0.357 | 0.434592 | 0.512079 | 0.982586 |
| 616 | 0.936932 | 0.54188 | -0.39505 | 0.431239 | 0.515148 | 0.969257 |
| 617 | 0.961357 | 0.636079 | -0.32528 | 0.436747 | 0.509902 | 0.991236 |
| 618 | 0.972492 | 0.55289 | -0.4196 | 0.429891 | 0.516675 | 0.963944 |
| 619 | 0.923215 | 0.505613 | -0.4176 | 0.429214 | 0.517042 | 0.961284 |
| 620 | 0.986965 | 0.542034 | -0.44493 | 0.428164 | 0.518452 | 0.95717 |
| 621 | 0.97633 | 0.612906 | -0.36342 | 0.434145 | 0.512528 | 0.9808 |
| 622 | 0.973488 | 0.591491 | -0.382 | 0.432745 | 0.513885 | 0.975223 |
| 623 | 0.923215 | 0.505613 | -0.4176 | 0.429214 | 0.517042 | 0.961284 |
| 624 | 0.990556 | 0.681067 | -0.30949 | 0.438125 | 0.508702 | 0.996803 |
| 625 | 0.98943 | 0.63651 | -0.35292 | 0.435046 | 0.511714 | 0.984402 |
| 626 | 0.957872 | 0.499115 | -0.45876 | 0.426532 | 0.519881 | 0.950809 |
| 627 | 0.987485 | 0.561586 | -0.4259 | 0.429635 | 0.517011 | 0.962936 |
| 628 | 0.918493 | 0.533206 | -0.38529 | 0.431717 | 0.514568 | 0.971148 |
| 629 | 0.923215 | 0.505613 | -0.4176 | 0.429214 | 0.517042 | 0.961284 |
| 630 | 0.98943 | 0.63651 | -0.35292 | 0.435046 | 0.511714 | 0.984402 |
| 631 | 0.936932 | 0.54188 | -0.39505 | 0.431239 | 0.515148 | 0.969257 |
| 632 | 0.937451 | 0.56278 | -0.37467 | 0.432817 | 0.513608 | 0.975512 |
| 633 | 0.787123 | 0.486854 | -0.30027 | 0.436473 | 0.509055 | 0.990134 |
| 634 | 0.918956 | 0.552208 | -0.36675 | 0.433168 | 0.513155 | 0.976908 |
| 635 | 0.892729 | 0.619301 | -0.27343 | 0.439864 | 0.506478 | 1.00387 |
| 636 | 0.806725 | 0.559789 | -0.24694 | 0.441016 | 0.504808 | 1.00857 |
| 637 | 0.918956 | 0.552208 | -0.36675 | 0.433168 | 0.513155 | 0.976908 |
| 638 | 0.937451 | 0.56278 | -0.37467 | 0.432817 | 0.513608 | 0.975512 |
| 639 | 0.974294 | 0.582579 | -0.39172 | 0.432028 | 0.514591 | 0.972382 |
| 640 | 0.987485 | 0.561586 | -0.4259 | 0.429635 | 0.517011 | 0.962936 |
| 641 | 0.986965 | 0.542034 | -0.44493 | 0.428164 | 0.518452 | 0.95717 |
| 642 | 0.892729 | 0.619301 | -0.27343 | 0.439864 | 0.506478 | 1.00387 |
| 643 | 0.937451 | 0.56278 | -0.37467 | 0.432817 | 0.513608 | 0.975512 |
| 644 | 0.923215 | 0.505613 | -0.4176 | 0.429214 | 0.517042 | 0.961284 |
| 645 | 0.986965 | 0.542034 | -0.44493 | 0.428164 | 0.518452 | 0.95717 |
| 646 | 0.98943 | 0.63651 | -0.35292 | 0.435046 | 0.511714 | 0.984402 |
| 647 | 0.923215 | 0.505613 | -0.4176 | 0.429214 | 0.517042 | 0.961284 |
| 648 | 0.985802 | 0.499189 | -0.48661 | 0.424831 | 0.521718 | 0.944216 |
| 649 | 0.917433 | 0.490551 | -0.42688 | 0.428342 | 0.517857 | 0.957865 |
| 650 | 0.806702 | 0.558688 | -0.24801 | 0.440928 | 0.504893 | 1.00821 |
| 651 | 0.806702 | 0.558688 | -0.24801 | 0.440928 | 0.504893 | 1.00821 |
| 652 | 0.918956 | 0.552208 | -0.36675 | 0.433168 | 0.513155 | 0.976908 |
| 653 | 0.98943 | 0.63651 | -0.35292 | 0.435046 | 0.511714 | 0.984402 |
| 654 | 0.974294 | 0.582579 | -0.39172 | 0.432028 | 0.514591 | 0.972382 |
| 655 | 0.974294 | 0.582579 | -0.39172 | 0.432028 | 0.514591 | 0.972382 |
| 656 | 0.937479 | 0.563894 | -0.37359 | 0.432905 | 0.513523 | 0.975859 |
| 657 | 0.971993 | 0.533855 | -0.43814 | 0.428445 | 0.51809 | 0.95827 |
| 658 | 0.892729 | 0.619301 | -0.27343 | 0.439864 | 0.506478 | 1.00387 |
| 659 | 1.09019 | 9.83424 | 8.74404 | 0.941295 | 0.022679 | 20.4974 |
| 660 | 0.98943 | 0.63651 | -0.35292 | 0.435046 | 0.511714 | 0.984402 |
| 661 | 0.918493 | 0.533206 | -0.38529 | 0.431717 | 0.514568 | 0.971148 |
| 662 | 0.932982 | 0.692761 | -0.24022 | 0.442525 | 0.504113 | 1.01476 |
| 663 | 0.918956 | 0.552208 | -0.36675 | 0.433168 | 0.513155 | 0.976908 |
| 664 | 0.98943 | 0.63651 | -0.35292 | 0.435046 | 0.511714 | 0.984402 |
| 665 | 0.98943 | 0.63651 | -0.35292 | 0.435046 | 0.511714 | 0.984402 |
| 666 | 0.956877 | 0.462146 | -0.49473 | 0.423501 | 0.522846 | 0.93909 |
| 667 | 0.937451 | 0.56278 | -0.37467 | 0.432817 | 0.513608 | 0.975512 |
| 668 | 0.973775 | 0.602736 | -0.37104 | 0.433562 | 0.513086 | 0.978475 |
| 669 | 0.922331 | 0.696783 | -0.22555 | 0.443477 | 0.50313 | 1.01868 |
| 670 | 0.987485 | 0.561586 | -0.4259 | 0.429635 | 0.517011 | 0.962936 |
| 671 | 0.923215 | 0.505613 | -0.4176 | 0.429214 | 0.517042 | 0.961284 |
| 672 | 0.806702 | 0.558688 | -0.24801 | 0.440928 | 0.504893 | 1.00821 |
| 673 | 0.936932 | 0.54188 | -0.39505 | 0.431239 | 0.515148 | 0.969257 |
| 674 | 0.918493 | 0.533206 | -0.38529 | 0.431717 | 0.514568 | 0.971148 |
| 675 | 0.972242 | 0.543285 | -0.42896 | 0.429169 | 0.517382 | 0.961107 |
| 676 | 0.937451 | 0.56278 | -0.37467 | 0.432817 | 0.513608 | 0.975512 |
| 677 | 0.919878 | 0.590742 | -0.32914 | 0.436031 | 0.510368 | 0.988356 |
| 678 | 0.857884 | 0.551426 | -0.30646 | 0.437001 | 0.50904 | 0.992262 |
| 679 | 0.991861 | 0.499837 | -0.49202 | 0.424517 | 0.522062 | 0.943004 |
| 680 | 0.932982 | 0.692761 | -0.24022 | 0.442525 | 0.504113 | 1.01476 |
| 681 | 0.990556 | 0.681067 | -0.30949 | 0.438125 | 0.508702 | 0.996803 |
| 682 | 0.918956 | 0.552208 | -0.36675 | 0.433168 | 0.513155 | 0.976908 |
| 683 | 0.986965 | 0.542034 | -0.44493 | 0.428164 | 0.518452 | 0.95717 |
| 684 | 0.917433 | 0.490551 | -0.42688 | 0.428342 | 0.517857 | 0.957865 |
| 685 | 0.987485 | 0.561586 | -0.4259 | 0.429635 | 0.517011 | 0.962936 |
| 686 | 0.986965 | 0.542034 | -0.44493 | 0.428164 | 0.518452 | 0.95717 |
| 687 | 0.984915 | 6.27855 | 5.29364 | 0.85094 | 0.108479 | 7.29774 |
| 688 | 0.98943 | 0.63651 | -0.35292 | 0.435046 | 0.511714 | 0.984402 |
| 689 | 0.936932 | 0.54188 | -0.39505 | 0.431239 | 0.515148 | 0.969257 |
| 690 | 0.922331 | 0.696783 | -0.22555 | 0.443477 | 0.50313 | 1.01868 |
| 691 | 0.936932 | 0.54188 | -0.39505 | 0.431239 | 0.515148 | 0.969257 |
| 692 | 0.806702 | 0.558688 | -0.24801 | 0.440928 | 0.504893 | 1.00821 |
| 693 | 0.917433 | 0.490551 | -0.42688 | 0.428342 | 0.517857 | 0.957865 |
| 694 | 0.932982 | 0.692761 | -0.24022 | 0.442525 | 0.504113 | 1.01476 |
| 695 | 0.806725 | 0.559789 | -0.24694 | 0.441016 | 0.504808 | 1.00857 |
| 696 | 0.986965 | 0.542034 | -0.44493 | 0.428164 | 0.518452 | 0.95717 |
| 697 | 0.97633 | 0.612906 | -0.36342 | 0.434145 | 0.512528 | 0.9808 |
| 698 | 0.918493 | 0.533206 | -0.38529 | 0.431717 | 0.514568 | 0.971148 |
| 699 | 0.932982 | 0.692761 | -0.24022 | 0.442525 | 0.504113 | 1.01476 |
| 700 | 0.932982 | 0.692761 | -0.24022 | 0.442525 | 0.504113 | 1.01476 |
| 701 | 0.98943 | 0.63651 | -0.35292 | 0.435046 | 0.511714 | 0.984402 |
| 702 | 0.923215 | 0.505613 | -0.4176 | 0.429214 | 0.517042 | 0.961284 |
| 703 | 0.937451 | 0.56278 | -0.37467 | 0.432817 | 0.513608 | 0.975512 |
| 704 | 0.936932 | 0.54188 | -0.39505 | 0.431239 | 0.515148 | 0.969257 |
| 705 | 0.987485 | 0.561586 | -0.4259 | 0.429635 | 0.517011 | 0.962936 |
| 706 | 0.858723 | 0.589883 | -0.26884 | 0.439875 | 0.506259 | 1.00391 |
| 707 | 0.937451 | 0.56278 | -0.37467 | 0.432817 | 0.513608 | 0.975512 |
| 708 | 0.932982 | 0.692761 | -0.24022 | 0.442525 | 0.504113 | 1.01476 |
| 709 | 0.803087 | 0.684908 | -0.11818 | 0.450428 | 0.495711 | 1.04774 |
| 710 | 0.919878 | 0.590742 | -0.32914 | 0.436031 | 0.510368 | 0.988356 |
| 711 | 0.919878 | 0.590742 | -0.32914 | 0.436031 | 0.510368 | 0.988356 |
| 712 | 0.917433 | 0.490551 | -0.42688 | 0.428342 | 0.517857 | 0.957865 |
| 713 | 0.973488 | 0.591491 | -0.382 | 0.432745 | 0.513885 | 0.975223 |
| 714 | 0.974139 | 0.617137 | -0.357 | 0.434592 | 0.512079 | 0.982586 |
| 715 | 0.918956 | 0.552208 | -0.36675 | 0.433168 | 0.513155 | 0.976908 |
| 716 | 0.918493 | 0.533206 | -0.38529 | 0.431717 | 0.514568 | 0.971148 |
| 717 | 0.985802 | 0.499189 | -0.48661 | 0.424831 | 0.521718 | 0.944216 |
| 718 | 0.806284 | 0.538028 | -0.26826 | 0.439345 | 0.506419 | 1.00175 |
| 719 | 0.987485 | 0.561586 | -0.4259 | 0.429635 | 0.517011 | 0.962936 |
| 720 | 0.937451 | 0.56278 | -0.37467 | 0.432817 | 0.513608 | 0.975512 |
| 721 | 0.803087 | 0.684908 | -0.11818 | 0.450428 | 0.495711 | 1.04774 |
| 722 | 0.937451 | 0.56278 | -0.37467 | 0.432817 | 0.513608 | 0.975512 |
| 723 | 0.991861 | 0.499837 | -0.49202 | 0.424517 | 0.522062 | 0.943004 |
| 724 | 0.932982 | 0.692761 | -0.24022 | 0.442525 | 0.504113 | 1.01476 |
| 725 | 0.973488 | 0.591491 | -0.382 | 0.432745 | 0.513885 | 0.975223 |
| 726 | 0.970786 | 0.488825 | -0.48196 | 0.424896 | 0.521564 | 0.944466 |
| 727 | 0.987409 | 0.558677 | -0.42873 | 0.429422 | 0.517219 | 0.962101 |
| 728 | 0.918493 | 0.533206 | -0.38529 | 0.431717 | 0.514568 | 0.971148 |
| 729 | 0.918724 | 0.542619 | -0.3761 | 0.432444 | 0.51386 | 0.974028 |
| 730 | 0.987485 | 0.561586 | -0.4259 | 0.429635 | 0.517011 | 0.962936 |
| 731 | 0.973488 | 0.591491 | -0.382 | 0.432745 | 0.513885 | 0.975223 |
| 732 | 0.973488 | 0.591491 | -0.382 | 0.432745 | 0.513885 | 0.975223 |
| 733 | 0.958991 | 0.541922 | -0.41707 | 0.429875 | 0.516613 | 0.963881 |
| 734 | 0.918724 | 0.542619 | -0.3761 | 0.432444 | 0.51386 | 0.974028 |
| 735 | 0.990556 | 0.681067 | -0.30949 | 0.438125 | 0.508702 | 0.996803 |
| 736 | 0.973488 | 0.591491 | -0.382 | 0.432745 | 0.513885 | 0.975223 |
| 737 | 0.97633 | 0.612906 | -0.36342 | 0.434145 | 0.512528 | 0.9808 |
| 738 | 0.937451 | 0.56278 | -0.37467 | 0.432817 | 0.513608 | 0.975512 |
| 739 | 0.788397 | 0.550531 | -0.23787 | 0.441528 | 0.504185 | 1.01067 |
| 740 | 0.936932 | 0.54188 | -0.39505 | 0.431239 | 0.515148 | 0.969257 |
| 741 | 0.996136 | 0.661383 | -0.33475 | 0.436404 | 0.510416 | 0.989857 |
| 742 | 0.917433 | 0.490551 | -0.42688 | 0.428342 | 0.517857 | 0.957865 |
| 743 | 0.932982 | 0.692761 | -0.24022 | 0.442525 | 0.504113 | 1.01476 |
| 744 | 0.803087 | 0.684908 | -0.11818 | 0.450428 | 0.495711 | 1.04774 |
| 745 | 0.937451 | 0.56278 | -0.37467 | 0.432817 | 0.513608 | 0.975512 |
| 746 | 1.20613 | 11.0843 | 9.87812 | 0.947793 | 0.012556 | 23.2079 |
| 747 | 0.919878 | 0.590742 | -0.32914 | 0.436031 | 0.510368 | 0.988356 |
| 748 | 0.922331 | 0.696783 | -0.22555 | 0.443477 | 0.50313 | 1.01868 |
| 749 | 0.936932 | 0.54188 | -0.39505 | 0.431239 | 0.515148 | 0.969257 |
| 750 | 0.789141 | 0.5889 | -0.20024 | 0.444412 | 0.50141 | 1.02255 |
| 751 | 0.990556 | 0.681067 | -0.30949 | 0.438125 | 0.508702 | 0.996803 |
| 752 | 0.919878 | 0.590742 | -0.32914 | 0.436031 | 0.510368 | 0.988356 |
| 753 | 0.937451 | 0.56278 | -0.37467 | 0.432817 | 0.513608 | 0.975512 |
| 754 | 0.937451 | 0.56278 | -0.37467 | 0.432817 | 0.513608 | 0.975512 |
| 755 | 0.974294 | 0.582579 | -0.39172 | 0.432028 | 0.514591 | 0.972382 |
| 756 | 0.987409 | 0.558677 | -0.42873 | 0.429422 | 0.517219 | 0.962101 |
| 757 | 0.957872 | 0.499115 | -0.45876 | 0.426532 | 0.519881 | 0.950809 |
| 758 | 0.987485 | 0.561586 | -0.4259 | 0.429635 | 0.517011 | 0.962936 |
| 759 | 0.917433 | 0.490551 | -0.42688 | 0.428342 | 0.517857 | 0.957865 |
| 760 | 0.789141 | 0.5889 | -0.20024 | 0.444412 | 0.50141 | 1.02255 |
| 761 | 0.919878 | 0.590742 | -0.32914 | 0.436031 | 0.510368 | 0.988356 |
| 762 | 0.803087 | 0.684908 | -0.11818 | 0.450428 | 0.495711 | 1.04774 |
| 763 | 0.918724 | 0.542619 | -0.3761 | 0.432444 | 0.51386 | 0.974028 |
| 764 | 0.918724 | 0.542619 | -0.3761 | 0.432444 | 0.51386 | 0.974028 |
| 765 | 0.917433 | 0.490551 | -0.42688 | 0.428342 | 0.517857 | 0.957865 |
| 766 | 0.994003 | 0.63539 | -0.35861 | 0.434685 | 0.512092 | 0.982958 |
| 767 | 0.997845 | 0.729543 | -0.2683 | 0.441001 | 0.505918 | 1.00851 |
| 768 | 0.997845 | 0.729543 | -0.2683 | 0.441001 | 0.505918 | 1.00851 |
| 769 | 0.997845 | 0.729543 | -0.2683 | 0.441001 | 0.505918 | 1.00851 |
| 770 | 0.997845 | 0.729543 | -0.2683 | 0.441001 | 0.505918 | 1.00851 |
| 771 | 0.997845 | 0.729543 | -0.2683 | 0.441001 | 0.505918 | 1.00851 |
| 772 | 0.997845 | 0.729543 | -0.2683 | 0.441001 | 0.505918 | 1.00851 |
| 773 | 0.997845 | 0.729543 | -0.2683 | 0.441001 | 0.505918 | 1.00851 |
| 774 | 0.997845 | 0.729543 | -0.2683 | 0.441001 | 0.505918 | 1.00851 |
| 775 | 0.997845 | 0.729543 | -0.2683 | 0.441001 | 0.505918 | 1.00851 |
| 776 | 0.997845 | 0.729543 | -0.2683 | 0.441001 | 0.505918 | 1.00851 |
| 777 | 0.997845 | 0.729543 | -0.2683 | 0.441001 | 0.505918 | 1.00851 |
| 778 | 0.997845 | 0.729543 | -0.2683 | 0.441001 | 0.505918 | 1.00851 |
| 779 | 0.997845 | 0.729543 | -0.2683 | 0.441001 | 0.505918 | 1.00851 |
| 780 | 0.997845 | 0.729543 | -0.2683 | 0.441001 | 0.505918 | 1.00851 |
| 781 | 0.997845 | 0.729543 | -0.2683 | 0.441001 | 0.505918 | 1.00851 |
| 782 | 0.997845 | 0.729543 | -0.2683 | 0.441001 | 0.505918 | 1.00851 |
| 783 | 0.997845 | 0.729543 | -0.2683 | 0.441001 | 0.505918 | 1.00851 |
| 784 | 0.997845 | 0.729543 | -0.2683 | 0.441001 | 0.505918 | 1.00851 |
| 785 | 0.997845 | 0.729543 | -0.2683 | 0.441001 | 0.505918 | 1.00851 |
| 786 | 0.997845 | 0.729543 | -0.2683 | 0.441001 | 0.505918 | 1.00851 |
| 787 | 0.997845 | 0.729543 | -0.2683 | 0.441001 | 0.505918 | 1.00851 |
| 788 | 0.997845 | 0.729543 | -0.2683 | 0.441001 | 0.505918 | 1.00851 |
| 789 | 0.997845 | 0.729543 | -0.2683 | 0.441001 | 0.505918 | 1.00851 |
| 790 | 0.997845 | 0.729543 | -0.2683 | 0.441001 | 0.505918 | 1.00851 |
| 791 | 0.997845 | 0.729543 | -0.2683 | 0.441001 | 0.505918 | 1.00851 |
| 792 | 0.997845 | 0.729543 | -0.2683 | 0.441001 | 0.505918 | 1.00851 |
| 793 | 0.997845 | 0.729543 | -0.2683 | 0.441001 | 0.505918 | 1.00851 |
| 794 | 0.997845 | 0.729543 | -0.2683 | 0.441001 | 0.505918 | 1.00851 |
| 795 | 0.997845 | 0.729543 | -0.2683 | 0.441001 | 0.505918 | 1.00851 |
| 796 | 0.997845 | 0.729543 | -0.2683 | 0.441001 | 0.505918 | 1.00851 |
| 797 | 0.997845 | 0.729543 | -0.2683 | 0.441001 | 0.505918 | 1.00851 |
| 798 | 0.997845 | 0.729543 | -0.2683 | 0.441001 | 0.505918 | 1.00851 |
| 799 | 0.997845 | 0.729543 | -0.2683 | 0.441001 | 0.505918 | 1.00851 |
| 800 | 0.997845 | 0.729543 | -0.2683 | 0.441001 | 0.505918 | 1.00851 |
| 801 | 0.997845 | 0.729543 | -0.2683 | 0.441001 | 0.505918 | 1.00851 |
| 802 | 0.997845 | 0.729543 | -0.2683 | 0.441001 | 0.505918 | 1.00851 |
| 803 | 0.997845 | 0.729543 | -0.2683 | 0.441001 | 0.505918 | 1.00851 |
| 804 | 0.997845 | 0.729543 | -0.2683 | 0.441001 | 0.505918 | 1.00851 |
| 805 | 0.997845 | 0.729543 | -0.2683 | 0.441001 | 0.505918 | 1.00851 |
| 806 | 0.997845 | 0.729543 | -0.2683 | 0.441001 | 0.505918 | 1.00851 |
| 807 | 0.997845 | 0.729543 | -0.2683 | 0.441001 | 0.505918 | 1.00851 |
| 808 | 0.997845 | 0.729543 | -0.2683 | 0.441001 | 0.505918 | 1.00851 |
| 809 | 0.997845 | 0.729543 | -0.2683 | 0.441001 | 0.505918 | 1.00851 |
| 810 | 0.997845 | 0.729543 | -0.2683 | 0.441001 | 0.505918 | 1.00851 |
| 811 | 0.997845 | 0.729543 | -0.2683 | 0.441001 | 0.505918 | 1.00851 |
| 812 | 0.997845 | 0.729543 | -0.2683 | 0.441001 | 0.505918 | 1.00851 |
| 813 | 0.997845 | 0.729543 | -0.2683 | 0.441001 | 0.505918 | 1.00851 |
| 814 | 0.997845 | 0.729543 | -0.2683 | 0.441001 | 0.505918 | 1.00851 |
| 815 | 0.997845 | 0.729543 | -0.2683 | 0.441001 | 0.505918 | 1.00851 |
| 816 | 0.997845 | 0.729543 | -0.2683 | 0.441001 | 0.505918 | 1.00851 |
| 817 | 0.997845 | 0.729543 | -0.2683 | 0.441001 | 0.505918 | 1.00851 |
| 818 | 0.997845 | 0.729543 | -0.2683 | 0.441001 | 0.505918 | 1.00851 |
| 819 | 0.997845 | 0.729543 | -0.2683 | 0.441001 | 0.505918 | 1.00851 |
| 820 | 0.997845 | 0.729543 | -0.2683 | 0.441001 | 0.505918 | 1.00851 |
| 821 | 0.997845 | 0.729543 | -0.2683 | 0.441001 | 0.505918 | 1.00851 |
| 822 | 0.997845 | 0.729543 | -0.2683 | 0.441001 | 0.505918 | 1.00851 |
| 823 | 0.997845 | 0.729543 | -0.2683 | 0.441001 | 0.505918 | 1.00851 |
| 824 | 0.997845 | 0.729543 | -0.2683 | 0.441001 | 0.505918 | 1.00851 |
| 825 | 0.997845 | 0.729543 | -0.2683 | 0.441001 | 0.505918 | 1.00851 |
| 826 | 0.997845 | 0.729543 | -0.2683 | 0.441001 | 0.505918 | 1.00851 |
| 827 | 0.997845 | 0.729543 | -0.2683 | 0.441001 | 0.505918 | 1.00851 |
| 828 | 0.997845 | 0.729543 | -0.2683 | 0.441001 | 0.505918 | 1.00851 |
| 829 | 0.997845 | 0.729543 | -0.2683 | 0.441001 | 0.505918 | 1.00851 |
| 830 | 0.997845 | 0.729543 | -0.2683 | 0.441001 | 0.505918 | 1.00851 |
| 831 | 0.997845 | 0.729543 | -0.2683 | 0.441001 | 0.505918 | 1.00851 |
| 832 | 0.997845 | 0.729543 | -0.2683 | 0.441001 | 0.505918 | 1.00851 |
| 833 | 0.997845 | 0.729543 | -0.2683 | 0.441001 | 0.505918 | 1.00851 |
| 834 | 0.997845 | 0.729543 | -0.2683 | 0.441001 | 0.505918 | 1.00851 |
| 835 | 0.997845 | 0.729543 | -0.2683 | 0.441001 | 0.505918 | 1.00851 |
| 836 | 0.997845 | 0.729543 | -0.2683 | 0.441001 | 0.505918 | 1.00851 |
| 837 | 0.997845 | 0.729543 | -0.2683 | 0.441001 | 0.505918 | 1.00851 |
| 838 | 0.997845 | 0.729543 | -0.2683 | 0.441001 | 0.505918 | 1.00851 |
| 839 | 0.997845 | 0.729543 | -0.2683 | 0.441001 | 0.505918 | 1.00851 |
| 840 | 0.997845 | 0.729543 | -0.2683 | 0.441001 | 0.505918 | 1.00851 |
| 841 | 0.937625 | 0.716835 | -0.22079 | 0.443889 | 0.502808 | 1.02038 |
| 842 | 0.987409 | 0.558677 | -0.42873 | 0.429422 | 0.517219 | 0.962101 |
| 843 | 0.932982 | 0.692761 | -0.24022 | 0.442525 | 0.504113 | 1.01476 |
| 844 | 0.958991 | 0.541922 | -0.41707 | 0.429875 | 0.516613 | 0.963881 |
| 845 | 0.958093 | 0.507466 | -0.45063 | 0.4272 | 0.519228 | 0.95341 |
| 846 | 0.987485 | 0.561586 | -0.4259 | 0.429635 | 0.517011 | 0.962936 |
| 847 | 0.857884 | 0.551426 | -0.30646 | 0.437001 | 0.50904 | 0.992262 |
| 848 | 0.961357 | 0.636079 | -0.32528 | 0.436747 | 0.509902 | 0.991236 |
| 849 | 0.957872 | 0.499115 | -0.45876 | 0.426532 | 0.519881 | 0.950809 |
| 850 | 0.932982 | 0.692761 | -0.24022 | 0.442525 | 0.504113 | 1.01476 |
| 851 | 0.918956 | 0.552208 | -0.36675 | 0.433168 | 0.513155 | 0.976908 |
| 852 | 0.932982 | 0.692761 | -0.24022 | 0.442525 | 0.504113 | 1.01476 |
| 853 | 0.97633 | 0.612906 | -0.36342 | 0.434145 | 0.512528 | 0.9808 |
| 854 | 0.974294 | 0.582579 | -0.39172 | 0.432028 | 0.514591 | 0.972382 |
| 855 | 0.957872 | 0.499115 | -0.45876 | 0.426532 | 0.519881 | 0.950809 |
| 856 | 0.936932 | 0.54188 | -0.39505 | 0.431239 | 0.515148 | 0.969257 |
| 857 | 0.974139 | 0.617137 | -0.357 | 0.434592 | 0.512079 | 0.982586 |
| 858 | 0.918493 | 0.533206 | -0.38529 | 0.431717 | 0.514568 | 0.971148 |
| 859 | 0.985802 | 0.499189 | -0.48661 | 0.424831 | 0.521718 | 0.944216 |
| 860 | 0.97633 | 0.612906 | -0.36342 | 0.434145 | 0.512528 | 0.9808 |
| 861 | 0.917433 | 0.490551 | -0.42688 | 0.428342 | 0.517857 | 0.957865 |
| 862 | 0.98943 | 0.63651 | -0.35292 | 0.435046 | 0.511714 | 0.984402 |
| 863 | 0.974139 | 0.617137 | -0.357 | 0.434592 | 0.512079 | 0.982586 |
| 864 | 0.958316 | 0.515962 | -0.44235 | 0.427866 | 0.518577 | 0.956005 |
| 865 | 0.996136 | 0.661383 | -0.33475 | 0.436404 | 0.510416 | 0.989857 |
| 866 | 0.936932 | 0.54188 | -0.39505 | 0.431239 | 0.515148 | 0.969257 |
| 867 | 0.806702 | 0.558688 | -0.24801 | 0.440928 | 0.504893 | 1.00821 |
| 868 | 0.936932 | 0.54188 | -0.39505 | 0.431239 | 0.515148 | 0.969257 |
| 869 | 0.919878 | 0.590742 | -0.32914 | 0.436031 | 0.510368 | 0.988356 |
| 870 | 0.973488 | 0.591491 | -0.382 | 0.432745 | 0.513885 | 0.975223 |
| 871 | 0.918724 | 0.542619 | -0.3761 | 0.432444 | 0.51386 | 0.974028 |
| 872 | 0.990556 | 0.681067 | -0.30949 | 0.438125 | 0.508702 | 0.996803 |
| 873 | 0.972492 | 0.55289 | -0.4196 | 0.429891 | 0.516675 | 0.963944 |
| 874 | 0.974294 | 0.582579 | -0.39172 | 0.432028 | 0.514591 | 0.972382 |
| 875 | 0.917433 | 0.490551 | -0.42688 | 0.428342 | 0.517857 | 0.957865 |
| 876 | 0.973775 | 0.602736 | -0.37104 | 0.433562 | 0.513086 | 0.978475 |
| 877 | 0.918493 | 0.533206 | -0.38529 | 0.431717 | 0.514568 | 0.971148 |
| 878 | 0.985802 | 0.499189 | -0.48661 | 0.424831 | 0.521718 | 0.944216 |
| 879 | 0.987485 | 0.561586 | -0.4259 | 0.429635 | 0.517011 | 0.962936 |
| 880 | 0.932982 | 0.692761 | -0.24022 | 0.442525 | 0.504113 | 1.01476 |
| 881 | 0.936932 | 0.54188 | -0.39505 | 0.431239 | 0.515148 | 0.969257 |
| 882 | 0.974294 | 0.582579 | -0.39172 | 0.432028 | 0.514591 | 0.972382 |
| 883 | 0.985802 | 0.499189 | -0.48661 | 0.424831 | 0.521718 | 0.944216 |
| 884 | 0.936932 | 0.54188 | -0.39505 | 0.431239 | 0.515148 | 0.969257 |
| 885 | 0.806702 | 0.558688 | -0.24801 | 0.440928 | 0.504893 | 1.00821 |
| 886 | 0.937451 | 0.56278 | -0.37467 | 0.432817 | 0.513608 | 0.975512 |
| 887 | 0.789141 | 0.5889 | -0.20024 | 0.444412 | 0.50141 | 1.02255 |
| 888 | 0.987485 | 0.561586 | -0.4259 | 0.429635 | 0.517011 | 0.962936 |
| 889 | 0.991861 | 0.499837 | -0.49202 | 0.424517 | 0.522062 | 0.943004 |
| 890 | 0.917433 | 0.490551 | -0.42688 | 0.428342 | 0.517857 | 0.957865 |
| 891 | 0.974294 | 0.582579 | -0.39172 | 0.432028 | 0.514591 | 0.972382 |
| 892 | 0.932982 | 0.692761 | -0.24022 | 0.442525 | 0.504113 | 1.01476 |
| 893 | 0.932982 | 0.692761 | -0.24022 | 0.442525 | 0.504113 | 1.01476 |
| 894 | 0.990556 | 0.681067 | -0.30949 | 0.438125 | 0.508702 | 0.996803 |
| 895 | 0.974139 | 0.617137 | -0.357 | 0.434592 | 0.512079 | 0.982586 |
| 896 | 0.986965 | 0.542034 | -0.44493 | 0.428164 | 0.518452 | 0.95717 |
| 897 | 0.937451 | 0.56278 | -0.37467 | 0.432817 | 0.513608 | 0.975512 |
| 898 | 0.974294 | 0.582579 | -0.39172 | 0.432028 | 0.514591 | 0.972382 |
| 899 | 0.923215 | 0.505613 | -0.4176 | 0.429214 | 0.517042 | 0.961284 |
| 900 | 0.806284 | 0.538028 | -0.26826 | 0.439345 | 0.506419 | 1.00175 |
| 901 | 0.96093 | 0.618709 | -0.34222 | 0.435524 | 0.511096 | 0.986318 |
| 902 | 0.97633 | 0.612906 | -0.36342 | 0.434145 | 0.512528 | 0.9808 |
| 903 | 0.937451 | 0.56278 | -0.37467 | 0.432817 | 0.513608 | 0.975512 |
| 904 | 0.98943 | 0.63651 | -0.35292 | 0.435046 | 0.511714 | 0.984402 |
| 905 | 0.917433 | 0.490551 | -0.42688 | 0.428342 | 0.517857 | 0.957865 |
| 906 | 0.937479 | 0.563894 | -0.37359 | 0.432905 | 0.513523 | 0.975859 |
| 907 | 0.937451 | 0.56278 | -0.37467 | 0.432817 | 0.513608 | 0.975512 |
| 908 | 0.936932 | 0.54188 | -0.39505 | 0.431239 | 0.515148 | 0.969257 |
| 909 | 0.985802 | 0.499189 | -0.48661 | 0.424831 | 0.521718 | 0.944216 |
| 910 | 0.98943 | 0.63651 | -0.35292 | 0.435046 | 0.511714 | 0.984402 |
| 911 | 0.986965 | 0.542034 | -0.44493 | 0.428164 | 0.518452 | 0.95717 |
| 912 | 0.937451 | 0.56278 | -0.37467 | 0.432817 | 0.513608 | 0.975512 |
| 913 | 0.987485 | 0.561586 | -0.4259 | 0.429635 | 0.517011 | 0.962936 |
| 914 | 0.958316 | 0.515962 | -0.44235 | 0.427866 | 0.518577 | 0.956005 |
| 915 | 0.932982 | 0.692761 | -0.24022 | 0.442525 | 0.504113 | 1.01476 |
| 916 | 0.919878 | 0.590742 | -0.32914 | 0.436031 | 0.510368 | 0.988356 |
| 917 | 0.923215 | 0.505613 | -0.4176 | 0.429214 | 0.517042 | 0.961284 |
| 918 | 0.923215 | 0.505613 | -0.4176 | 0.429214 | 0.517042 | 0.961284 |
| 919 | 0.974294 | 0.582579 | -0.39172 | 0.432028 | 0.514591 | 0.972382 |
| 920 | 0.958093 | 0.507466 | -0.45063 | 0.4272 | 0.519228 | 0.95341 |
| 921 | 0.986965 | 0.542034 | -0.44493 | 0.428164 | 0.518452 | 0.95717 |
| 922 | 0.803087 | 0.684908 | -0.11818 | 0.450428 | 0.495711 | 1.04774 |
| 923 | 0.937451 | 0.56278 | -0.37467 | 0.432817 | 0.513608 | 0.975512 |
| 924 | 0.937451 | 0.56278 | -0.37467 | 0.432817 | 0.513608 | 0.975512 |
| 925 | 0.98943 | 0.63651 | -0.35292 | 0.435046 | 0.511714 | 0.984402 |
| 926 | 0.98943 | 0.63651 | -0.35292 | 0.435046 | 0.511714 | 0.984402 |
| 927 | 0.937451 | 0.56278 | -0.37467 | 0.432817 | 0.513608 | 0.975512 |
| 928 | 0.922331 | 0.696783 | -0.22555 | 0.443477 | 0.50313 | 1.01868 |
| 929 | 0.917433 | 0.490551 | -0.42688 | 0.428342 | 0.517857 | 0.957865 |
| 930 | 0.917433 | 0.490551 | -0.42688 | 0.428342 | 0.517857 | 0.957865 |
| 931 | 0.990556 | 0.681067 | -0.30949 | 0.438125 | 0.508702 | 0.996803 |
| 932 | 0.922331 | 0.696783 | -0.22555 | 0.443477 | 0.50313 | 1.01868 |
| 933 | 0.98943 | 0.63651 | -0.35292 | 0.435046 | 0.511714 | 0.984402 |
| 934 | 0.974294 | 0.582579 | -0.39172 | 0.432028 | 0.514591 | 0.972382 |
| 935 | 0.919878 | 0.590742 | -0.32914 | 0.436031 | 0.510368 | 0.988356 |
| 936 | 0.936932 | 0.54188 | -0.39505 | 0.431239 | 0.515148 | 0.969257 |
| 937 | 0.971993 | 0.533855 | -0.43814 | 0.428445 | 0.51809 | 0.95827 |
| 938 | 0.803087 | 0.684908 | -0.11818 | 0.450428 | 0.495711 | 1.04774 |
| 939 | 0.937451 | 0.56278 | -0.37467 | 0.432817 | 0.513608 | 0.975512 |
| 940 | 0.98943 | 0.63651 | -0.35292 | 0.435046 | 0.511714 | 0.984402 |
| 941 | 0.917433 | 0.490551 | -0.42688 | 0.428342 | 0.517857 | 0.957865 |
| 942 | 0.932982 | 0.692761 | -0.24022 | 0.442525 | 0.504113 | 1.01476 |
| 943 | 0.98943 | 0.63651 | -0.35292 | 0.435046 | 0.511714 | 0.984402 |
| 944 | 0.922331 | 0.696783 | -0.22555 | 0.443477 | 0.50313 | 1.01868 |
| 945 | 0.98943 | 0.63651 | -0.35292 | 0.435046 | 0.511714 | 0.984402 |
| 946 | 0.918493 | 0.533206 | -0.38529 | 0.431717 | 0.514568 | 0.971148 |
| 947 | 0.937451 | 0.56278 | -0.37467 | 0.432817 | 0.513608 | 0.975512 |
| 948 | 0.985802 | 0.499189 | -0.48661 | 0.424831 | 0.521718 | 0.944216 |
| 949 | 0.918956 | 0.552208 | -0.36675 | 0.433168 | 0.513155 | 0.976908 |
| 950 | 0.974139 | 0.617137 | -0.357 | 0.434592 | 0.512079 | 0.982586 |
| 951 | 0.974139 | 0.617137 | -0.357 | 0.434592 | 0.512079 | 0.982586 |
| 952 | 0.923215 | 0.505613 | -0.4176 | 0.429214 | 0.517042 | 0.961284 |
| 953 | 0.932982 | 0.692761 | -0.24022 | 0.442525 | 0.504113 | 1.01476 |
| 954 | 0.987485 | 0.561586 | -0.4259 | 0.429635 | 0.517011 | 0.962936 |
| 955 | 0.973488 | 0.591491 | -0.382 | 0.432745 | 0.513885 | 0.975223 |
| 956 | 0.972242 | 0.543285 | -0.42896 | 0.429169 | 0.517382 | 0.961107 |
| 957 | 0.937451 | 0.56278 | -0.37467 | 0.432817 | 0.513608 | 0.975512 |
| 958 | 0.919878 | 0.590742 | -0.32914 | 0.436031 | 0.510368 | 0.988356 |
| 959 | 0.987485 | 0.561586 | -0.4259 | 0.429635 | 0.517011 | 0.962936 |
| 960 | 0.97633 | 0.612906 | -0.36342 | 0.434145 | 0.512528 | 0.9808 |
| 961 | 0.803087 | 0.684908 | -0.11818 | 0.450428 | 0.495711 | 1.04774 |
| 962 | 0.973775 | 0.602736 | -0.37104 | 0.433562 | 0.513086 | 0.978475 |
| 963 | 0.857884 | 0.551426 | -0.30646 | 0.437001 | 0.50904 | 0.992262 |
| 964 | 0.986965 | 0.542034 | -0.44493 | 0.428164 | 0.518452 | 0.95717 |
| 965 | 0.787123 | 0.486854 | -0.30027 | 0.436473 | 0.509055 | 0.990134 |
| 966 | 0.987485 | 0.561586 | -0.4259 | 0.429635 | 0.517011 | 0.962936 |
| 967 | 0.987485 | 0.561586 | -0.4259 | 0.429635 | 0.517011 | 0.962936 |
| 968 | 0.918956 | 0.552208 | -0.36675 | 0.433168 | 0.513155 | 0.976908 |
| 969 | 0.986965 | 0.542034 | -0.44493 | 0.428164 | 0.518452 | 0.95717 |
| 970 | 0.788397 | 0.550531 | -0.23787 | 0.441528 | 0.504185 | 1.01067 |
| 971 | 0.974139 | 0.617137 | -0.357 | 0.434592 | 0.512079 | 0.982586 |
| 972 | 0.986965 | 0.542034 | -0.44493 | 0.428164 | 0.518452 | 0.95717 |
| 973 | 0.990556 | 0.681067 | -0.30949 | 0.438125 | 0.508702 | 0.996803 |
| 974 | 0.97633 | 0.612906 | -0.36342 | 0.434145 | 0.512528 | 0.9808 |
| 975 | 0.932982 | 0.692761 | -0.24022 | 0.442525 | 0.504113 | 1.01476 |
| 976 | 0.972492 | 0.55289 | -0.4196 | 0.429891 | 0.516675 | 0.963944 |
| 977 | 0.936932 | 0.54188 | -0.39505 | 0.431239 | 0.515148 | 0.969257 |
| 978 | 0.789141 | 0.5889 | -0.20024 | 0.444412 | 0.50141 | 1.02255 |
| 979 | 0.985802 | 0.499189 | -0.48661 | 0.424831 | 0.521718 | 0.944216 |
| 980 | 0.859272 | 0.615434 | -0.24384 | 0.441734 | 0.50446 | 1.01151 |
| 981 | 0.803087 | 0.684908 | -0.11818 | 0.450428 | 0.495711 | 1.04774 |
| 982 | 0.98943 | 0.63651 | -0.35292 | 0.435046 | 0.511714 | 0.984402 |
| 983 | 0.806702 | 0.558688 | -0.24801 | 0.440928 | 0.504893 | 1.00821 |
| 984 | 0.937451 | 0.56278 | -0.37467 | 0.432817 | 0.513608 | 0.975512 |
| 985 | 0.936932 | 0.54188 | -0.39505 | 0.431239 | 0.515148 | 0.969257 |
| 986 | 0.987485 | 0.561586 | -0.4259 | 0.429635 | 0.517011 | 0.962936 |
| 987 | 0.919878 | 0.590742 | -0.32914 | 0.436031 | 0.510368 | 0.988356 |
| 988 | 0.806702 | 0.558688 | -0.24801 | 0.440928 | 0.504893 | 1.00821 |
| 989 | 0.932982 | 0.692761 | -0.24022 | 0.442525 | 0.504113 | 1.01476 |
| 990 | 0.932982 | 0.692761 | -0.24022 | 0.442525 | 0.504113 | 1.01476 |
| 991 | 0.803087 | 0.684908 | -0.11818 | 0.450428 | 0.495711 | 1.04774 |
| 992 | 0.97633 | 0.612906 | -0.36342 | 0.434145 | 0.512528 | 0.9808 |
| 993 | 0.970786 | 0.488825 | -0.48196 | 0.424896 | 0.521564 | 0.944466 |
| 994 | 0.97633 | 0.612906 | -0.36342 | 0.434145 | 0.512528 | 0.9808 |
| 995 | 0.917433 | 0.490551 | -0.42688 | 0.428342 | 0.517857 | 0.957865 |
| 996 | 0.970786 | 0.488825 | -0.48196 | 0.424896 | 0.521564 | 0.944466 |
| 997 | 0.972242 | 0.543285 | -0.42896 | 0.429169 | 0.517382 | 0.961107 |
| 998 | 0.972492 | 0.55289 | -0.4196 | 0.429891 | 0.516675 | 0.963944 |
| 999 | 0.97633 | 0.612906 | -0.36342 | 0.434145 | 0.512528 | 0.9808 |
| 1000 | 0.987224 | 0.551719 | -0.43551 | 0.4289 | 0.517731 | 0.960052 |
| 1001 | 0.937451 | 0.56278 | -0.37467 | 0.432817 | 0.513608 | 0.975512 |
| 1002 | 0.932982 | 0.692761 | -0.24022 | 0.442525 | 0.504113 | 1.01476 |
| 1003 | 0.923215 | 0.505613 | -0.4176 | 0.429214 | 0.517042 | 0.961284 |
| 1004 | 0.803087 | 0.684908 | -0.11818 | 0.450428 | 0.495711 | 1.04774 |
| 1005 | 0.932982 | 0.692761 | -0.24022 | 0.442525 | 0.504113 | 1.01476 |
| 1006 | 0.970786 | 0.488825 | -0.48196 | 0.424896 | 0.521564 | 0.944466 |
| 1007 | 0.917433 | 0.490551 | -0.42688 | 0.428342 | 0.517857 | 0.957865 |
| 1008 | 0.987409 | 0.558677 | -0.42873 | 0.429422 | 0.517219 | 0.962101 |
| 1009 | 0.986965 | 0.542034 | -0.44493 | 0.428164 | 0.518452 | 0.95717 |
| 1010 | 0.937451 | 0.56278 | -0.37467 | 0.432817 | 0.513608 | 0.975512 |
| 1011 | 0.790468 | 0.659182 | -0.13129 | 0.449464 | 0.496557 | 1.04366 |
| 1012 | 0.986965 | 0.542034 | -0.44493 | 0.428164 | 0.518452 | 0.95717 |
| 1013 | 0.970786 | 0.488825 | -0.48196 | 0.424896 | 0.521564 | 0.944466 |
| 1014 | 0.957872 | 0.499115 | -0.45876 | 0.426532 | 0.519881 | 0.950809 |
| 1015 | 0.987409 | 0.558677 | -0.42873 | 0.429422 | 0.517219 | 0.962101 |
| 1016 | 0.918724 | 0.542619 | -0.3761 | 0.432444 | 0.51386 | 0.974028 |
| 1017 | 0.98943 | 0.63651 | -0.35292 | 0.435046 | 0.511714 | 0.984402 |
| 1018 | 0.973488 | 0.591491 | -0.382 | 0.432745 | 0.513885 | 0.975223 |
| 1019 | 0.919878 | 0.590742 | -0.32914 | 0.436031 | 0.510368 | 0.988356 |
| 1020 | 0.937451 | 0.56278 | -0.37467 | 0.432817 | 0.513608 | 0.975512 |
| 1021 | 0.918493 | 0.533206 | -0.38529 | 0.431717 | 0.514568 | 0.971148 |
| 1022 | 0.936932 | 0.54188 | -0.39505 | 0.431239 | 0.515148 | 0.969257 |
| 1023 | 0.996136 | 0.661383 | -0.33475 | 0.436404 | 0.510416 | 0.989857 |
| 1024 | 0.987485 | 0.561586 | -0.4259 | 0.429635 | 0.517011 | 0.962936 |
| 1025 | 0.987485 | 0.561586 | -0.4259 | 0.429635 | 0.517011 | 0.962936 |
| 1026 | 0.932982 | 0.692761 | -0.24022 | 0.442525 | 0.504113 | 1.01476 |
| 1027 | 0.937451 | 0.56278 | -0.37467 | 0.432817 | 0.513608 | 0.975512 |
| 1028 | 0.98943 | 0.63651 | -0.35292 | 0.435046 | 0.511714 | 0.984402 |
| 1029 | 0.788397 | 0.550531 | -0.23787 | 0.441528 | 0.504185 | 1.01067 |
| 1030 | 0.922331 | 0.696783 | -0.22555 | 0.443477 | 0.50313 | 1.01868 |
| 1031 | 0.987485 | 0.561586 | -0.4259 | 0.429635 | 0.517011 | 0.962936 |
| 1032 | 0.932982 | 0.692761 | -0.24022 | 0.442525 | 0.504113 | 1.01476 |
| 1033 | 0.97633 | 0.612906 | -0.36342 | 0.434145 | 0.512528 | 0.9808 |
| 1034 | 0.97633 | 0.612906 | -0.36342 | 0.434145 | 0.512528 | 0.9808 |
| 1035 | 0.986965 | 0.542034 | -0.44493 | 0.428164 | 0.518452 | 0.95717 |
| 1036 | 0.937451 | 0.56278 | -0.37467 | 0.432817 | 0.513608 | 0.975512 |
| 1037 | 0.974294 | 0.582579 | -0.39172 | 0.432028 | 0.514591 | 0.972382 |
| 1038 | 0.987409 | 0.558677 | -0.42873 | 0.429422 | 0.517219 | 0.962101 |
| 1039 | 0.987224 | 0.551719 | -0.43551 | 0.4289 | 0.517731 | 0.960052 |
| 1040 | 0.918956 | 0.552208 | -0.36675 | 0.433168 | 0.513155 | 0.976908 |
| 1041 | 0.986965 | 0.542034 | -0.44493 | 0.428164 | 0.518452 | 0.95717 |
| 1042 | 0.806702 | 0.558688 | -0.24801 | 0.440928 | 0.504893 | 1.00821 |
| 1043 | 0.918724 | 0.542619 | -0.3761 | 0.432444 | 0.51386 | 0.974028 |
| 1044 | 0.919878 | 0.590742 | -0.32914 | 0.436031 | 0.510368 | 0.988356 |
| 1045 | 0.936932 | 0.54188 | -0.39505 | 0.431239 | 0.515148 | 0.969257 |
| 1046 | 1.08211 | 5.90461 | 4.8225 | 0.832826 | 0.123223 | 6.3685 |
| 1047 | 0.918724 | 0.542619 | -0.3761 | 0.432444 | 0.51386 | 0.974028 |
| 1048 | 0.917433 | 0.490551 | -0.42688 | 0.428342 | 0.517857 | 0.957865 |
| 1049 | 0.986965 | 0.542034 | -0.44493 | 0.428164 | 0.518452 | 0.95717 |
| 1050 | 0.987485 | 0.561586 | -0.4259 | 0.429635 | 0.517011 | 0.962936 |
| 1051 | 0.922331 | 0.696783 | -0.22555 | 0.443477 | 0.50313 | 1.01868 |
| 1052 | 0.974139 | 0.617137 | -0.357 | 0.434592 | 0.512079 | 0.982586 |
| 1053 | 0.919878 | 0.590742 | -0.32914 | 0.436031 | 0.510368 | 0.988356 |
| 1054 | 0.987409 | 0.558677 | -0.42873 | 0.429422 | 0.517219 | 0.962101 |
| 1055 | 0.987485 | 0.561586 | -0.4259 | 0.429635 | 0.517011 | 0.962936 |
| 1056 | 0.974139 | 0.617137 | -0.357 | 0.434592 | 0.512079 | 0.982586 |
| 1057 | 0.806702 | 0.558688 | -0.24801 | 0.440928 | 0.504893 | 1.00821 |
| 1058 | 0.986965 | 0.542034 | -0.44493 | 0.428164 | 0.518452 | 0.95717 |
| 1059 | 0.986965 | 0.542034 | -0.44493 | 0.428164 | 0.518452 | 0.95717 |
| 1060 | 0.987409 | 0.558677 | -0.42873 | 0.429422 | 0.517219 | 0.962101 |
| 1061 | 0.974294 | 0.582579 | -0.39172 | 0.432028 | 0.514591 | 0.972382 |
| 1062 | 0.987409 | 0.558677 | -0.42873 | 0.429422 | 0.517219 | 0.962101 |
| 1063 | 0.788024 | 0.531612 | -0.25641 | 0.440065 | 0.505593 | 1.00469 |
| 1064 | 0.987485 | 0.561586 | -0.4259 | 0.429635 | 0.517011 | 0.962936 |
| 1065 | 0.98943 | 0.63651 | -0.35292 | 0.435046 | 0.511714 | 0.984402 |
| 1066 | 0.990556 | 0.681067 | -0.30949 | 0.438125 | 0.508702 | 0.996803 |
| 1067 | 0.957872 | 0.499115 | -0.45876 | 0.426532 | 0.519881 | 0.950809 |
| 1068 | 0.932982 | 0.692761 | -0.24022 | 0.442525 | 0.504113 | 1.01476 |
| 1069 | 0.918956 | 0.552208 | -0.36675 | 0.433168 | 0.513155 | 0.976908 |
| 1070 | 0.98943 | 0.63651 | -0.35292 | 0.435046 | 0.511714 | 0.984402 |
| 1071 | 0.97633 | 0.612906 | -0.36342 | 0.434145 | 0.512528 | 0.9808 |
| 1072 | 0.936932 | 0.54188 | -0.39505 | 0.431239 | 0.515148 | 0.969257 |
| 1073 | 0.973488 | 0.591491 | -0.382 | 0.432745 | 0.513885 | 0.975223 |
| 1074 | 0.918724 | 0.542619 | -0.3761 | 0.432444 | 0.51386 | 0.974028 |
| 1075 | 0.918956 | 0.552208 | -0.36675 | 0.433168 | 0.513155 | 0.976908 |
| 1076 | 0.932982 | 0.692761 | -0.24022 | 0.442525 | 0.504113 | 1.01476 |
| 1077 | 0.987485 | 0.561586 | -0.4259 | 0.429635 | 0.517011 | 0.962936 |
| 1078 | 0.985802 | 0.499189 | -0.48661 | 0.424831 | 0.521718 | 0.944216 |
| 1079 | 0.918956 | 0.552208 | -0.36675 | 0.433168 | 0.513155 | 0.976908 |
| 1080 | 0.98943 | 0.63651 | -0.35292 | 0.435046 | 0.511714 | 0.984402 |
| 1081 | 0.918956 | 0.552208 | -0.36675 | 0.433168 | 0.513155 | 0.976908 |
| 1082 | 0.996136 | 0.661383 | -0.33475 | 0.436404 | 0.510416 | 0.989857 |
| 1083 | 0.923215 | 0.505613 | -0.4176 | 0.429214 | 0.517042 | 0.961284 |
| 1084 | 0.985802 | 0.499189 | -0.48661 | 0.424831 | 0.521718 | 0.944216 |
| 1085 | 0.986965 | 0.542034 | -0.44493 | 0.428164 | 0.518452 | 0.95717 |
| 1086 | 0.990556 | 0.681067 | -0.30949 | 0.438125 | 0.508702 | 0.996803 |
| 1087 | 0.991861 | 0.499837 | -0.49202 | 0.424517 | 0.522062 | 0.943004 |
| 1088 | 0.986965 | 0.542034 | -0.44493 | 0.428164 | 0.518452 | 0.95717 |
| 1089 | 0.987409 | 0.558677 | -0.42873 | 0.429422 | 0.517219 | 0.962101 |
| 1090 | 0.922331 | 0.696783 | -0.22555 | 0.443477 | 0.50313 | 1.01868 |
| 1091 | 0.937451 | 0.56278 | -0.37467 | 0.432817 | 0.513608 | 0.975512 |
| 1092 | 0.987485 | 0.561586 | -0.4259 | 0.429635 | 0.517011 | 0.962936 |
| 1093 | 0.974294 | 0.582579 | -0.39172 | 0.432028 | 0.514591 | 0.972382 |
| 1094 | 0.991861 | 0.499837 | -0.49202 | 0.424517 | 0.522062 | 0.943004 |
| 1095 | 0.958316 | 0.515962 | -0.44235 | 0.427866 | 0.518577 | 0.956005 |
| 1096 | 0.985802 | 0.499189 | -0.48661 | 0.424831 | 0.521718 | 0.944216 |
| 1097 | 0.932982 | 0.692761 | -0.24022 | 0.442525 | 0.504113 | 1.01476 |
| 1098 | 0.973488 | 0.591491 | -0.382 | 0.432745 | 0.513885 | 0.975223 |
| 1099 | 0.974294 | 0.582579 | -0.39172 | 0.432028 | 0.514591 | 0.972382 |
| 1100 | 0.986965 | 0.542034 | -0.44493 | 0.428164 | 0.518452 | 0.95717 |
| 1101 | 0.937451 | 0.56278 | -0.37467 | 0.432817 | 0.513608 | 0.975512 |
| 1102 | 0.987485 | 0.561586 | -0.4259 | 0.429635 | 0.517011 | 0.962936 |
| 1103 | 0.918546 | 5.88615 | 4.9676 | 0.84592 | 0.114347 | 7.01831 |
| 1104 | 0.919878 | 0.590742 | -0.32914 | 0.436031 | 0.510368 | 0.988356 |
| 1105 | 0.987224 | 0.551719 | -0.43551 | 0.4289 | 0.517731 | 0.960052 |
| 1106 | 0.973488 | 0.591491 | -0.382 | 0.432745 | 0.513885 | 0.975223 |
| 1107 | 0.790468 | 0.659182 | -0.13129 | 0.449464 | 0.496557 | 1.04366 |
| 1108 | 0.803087 | 0.684908 | -0.11818 | 0.450428 | 0.495711 | 1.04774 |
| 1109 | 0.974294 | 0.582579 | -0.39172 | 0.432028 | 0.514591 | 0.972382 |
| 1110 | 0.974294 | 0.582579 | -0.39172 | 0.432028 | 0.514591 | 0.972382 |
| 1111 | 0.974139 | 0.617137 | -0.357 | 0.434592 | 0.512079 | 0.982586 |
| 1112 | 0.932982 | 0.692761 | -0.24022 | 0.442525 | 0.504113 | 1.01476 |
| 1113 | 0.936932 | 0.54188 | -0.39505 | 0.431239 | 0.515148 | 0.969257 |
| 1114 | 0.806702 | 0.558688 | -0.24801 | 0.440928 | 0.504893 | 1.00821 |
| 1115 | 0.806702 | 0.558688 | -0.24801 | 0.440928 | 0.504893 | 1.00821 |
| 1116 | 0.974139 | 0.617137 | -0.357 | 0.434592 | 0.512079 | 0.982586 |
| 1117 | 0.973488 | 0.591491 | -0.382 | 0.432745 | 0.513885 | 0.975223 |
| 1118 | 0.987224 | 0.551719 | -0.43551 | 0.4289 | 0.517731 | 0.960052 |
| 1119 | 0.937479 | 0.563894 | -0.37359 | 0.432905 | 0.513523 | 0.975859 |
| 1120 | 0.856447 | 0.487601 | -0.36885 | 0.431968 | 0.513918 | 0.972142 |
| 1121 | 0.990556 | 0.681067 | -0.30949 | 0.438125 | 0.508702 | 0.996803 |
| 1122 | 0.986965 | 0.542034 | -0.44493 | 0.428164 | 0.518452 | 0.95717 |
| 1123 | 0.986965 | 0.542034 | -0.44493 | 0.428164 | 0.518452 | 0.95717 |
| 1124 | 0.973488 | 0.591491 | -0.382 | 0.432745 | 0.513885 | 0.975223 |
| 1125 | 0.987485 | 0.561586 | -0.4259 | 0.429635 | 0.517011 | 0.962936 |
| 1126 | 0.974139 | 0.617137 | -0.357 | 0.434592 | 0.512079 | 0.982586 |
| 1127 | 0.98943 | 0.63651 | -0.35292 | 0.435046 | 0.511714 | 0.984402 |
| 1128 | 0.987485 | 0.561586 | -0.4259 | 0.429635 | 0.517011 | 0.962936 |
| 1129 | 0.937451 | 0.56278 | -0.37467 | 0.432817 | 0.513608 | 0.975512 |
| 1130 | 0.987485 | 0.561586 | -0.4259 | 0.429635 | 0.517011 | 0.962936 |
| 1131 | 0.936932 | 0.54188 | -0.39505 | 0.431239 | 0.515148 | 0.969257 |
| 1132 | 0.932982 | 0.692761 | -0.24022 | 0.442525 | 0.504113 | 1.01476 |
| 1133 | 0.974294 | 0.582579 | -0.39172 | 0.432028 | 0.514591 | 0.972382 |
| 1134 | 0.806702 | 0.558688 | -0.24801 | 0.440928 | 0.504893 | 1.00821 |
| 1135 | 0.918956 | 0.552208 | -0.36675 | 0.433168 | 0.513155 | 0.976908 |
| 1136 | 0.932982 | 0.692761 | -0.24022 | 0.442525 | 0.504113 | 1.01476 |
| 1137 | 0.917433 | 0.490551 | -0.42688 | 0.428342 | 0.517857 | 0.957865 |
| 1138 | 0.806702 | 0.558688 | -0.24801 | 0.440928 | 0.504893 | 1.00821 |
| 1139 | 0.932982 | 0.692761 | -0.24022 | 0.442525 | 0.504113 | 1.01476 |
| 1140 | 0.806284 | 0.538028 | -0.26826 | 0.439345 | 0.506419 | 1.00175 |
| 1141 | 0.987485 | 0.561586 | -0.4259 | 0.429635 | 0.517011 | 0.962936 |
| 1142 | 6.39071 | 0.757941 | -5.63277 | 0.075299 | 0.893229 | 0.104097 |
| 1143 | 0.972492 | 0.55289 | -0.4196 | 0.429891 | 0.516675 | 0.963944 |
| 1144 | 0.973488 | 0.591491 | -0.382 | 0.432745 | 0.513885 | 0.975223 |
| 1145 | 0.932982 | 0.692761 | -0.24022 | 0.442525 | 0.504113 | 1.01476 |
| 1146 | 0.958316 | 0.515962 | -0.44235 | 0.427866 | 0.518577 | 0.956005 |
| 1147 | 0.937451 | 0.56278 | -0.37467 | 0.432817 | 0.513608 | 0.975512 |
| 1148 | 0.972492 | 0.55289 | -0.4196 | 0.429891 | 0.516675 | 0.963944 |
| 1149 | 0.987485 | 0.561586 | -0.4259 | 0.429635 | 0.517011 | 0.962936 |
| 1150 | 0.974294 | 0.582579 | -0.39172 | 0.432028 | 0.514591 | 0.972382 |
| 1151 | 0.932982 | 0.692761 | -0.24022 | 0.442525 | 0.504113 | 1.01476 |
| 1152 | 0.986965 | 0.542034 | -0.44493 | 0.428164 | 0.518452 | 0.95717 |
| 1153 | 0.790468 | 0.659182 | -0.13129 | 0.449464 | 0.496557 | 1.04366 |
| 1154 | 0.932982 | 0.692761 | -0.24022 | 0.442525 | 0.504113 | 1.01476 |
| 1155 | 0.919878 | 0.590742 | -0.32914 | 0.436031 | 0.510368 | 0.988356 |
| 1156 | 0.98943 | 0.63651 | -0.35292 | 0.435046 | 0.511714 | 0.984402 |
| 1157 | 0.970786 | 0.488825 | -0.48196 | 0.424896 | 0.521564 | 0.944466 |
| 1158 | 0.97633 | 0.612906 | -0.36342 | 0.434145 | 0.512528 | 0.9808 |
| 1159 | 0.937479 | 0.563894 | -0.37359 | 0.432905 | 0.513523 | 0.975859 |
| 1160 | 0.937479 | 0.563894 | -0.37359 | 0.432905 | 0.513523 | 0.975859 |
| 1161 | 0.974139 | 0.617137 | -0.357 | 0.434592 | 0.512079 | 0.982586 |
| 1162 | 0.990556 | 0.681067 | -0.30949 | 0.438125 | 0.508702 | 0.996803 |
| 1163 | 0.917433 | 0.490551 | -0.42688 | 0.428342 | 0.517857 | 0.957865 |
| 1164 | 0.937451 | 0.56278 | -0.37467 | 0.432817 | 0.513608 | 0.975512 |
| 1165 | 0.917433 | 0.490551 | -0.42688 | 0.428342 | 0.517857 | 0.957865 |
| 1166 | 0.987485 | 0.561586 | -0.4259 | 0.429635 | 0.517011 | 0.962936 |
| 1167 | 0.923215 | 0.505613 | -0.4176 | 0.429214 | 0.517042 | 0.961284 |
| 1168 | 0.936932 | 0.54188 | -0.39505 | 0.431239 | 0.515148 | 0.969257 |
| 1169 | 0.958316 | 0.515962 | -0.44235 | 0.427866 | 0.518577 | 0.956005 |
| 1170 | 0.918493 | 0.533206 | -0.38529 | 0.431717 | 0.514568 | 0.971148 |
| 1171 | 0.918724 | 0.542619 | -0.3761 | 0.432444 | 0.51386 | 0.974028 |
| 1172 | 0.937451 | 0.56278 | -0.37467 | 0.432817 | 0.513608 | 0.975512 |
| 1173 | 0.973488 | 0.591491 | -0.382 | 0.432745 | 0.513885 | 0.975223 |
| 1174 | 0.972492 | 0.55289 | -0.4196 | 0.429891 | 0.516675 | 0.963944 |
| 1175 | 0.991861 | 0.499837 | -0.49202 | 0.424517 | 0.522062 | 0.943004 |
| 1176 | 0.932982 | 0.692761 | -0.24022 | 0.442525 | 0.504113 | 1.01476 |
| 1177 | 0.932982 | 0.692761 | -0.24022 | 0.442525 | 0.504113 | 1.01476 |
| 1178 | 0.857884 | 0.551426 | -0.30646 | 0.437001 | 0.50904 | 0.992262 |
| 1179 | 0.958316 | 0.515962 | -0.44235 | 0.427866 | 0.518577 | 0.956005 |
| 1180 | 0.985802 | 0.499189 | -0.48661 | 0.424831 | 0.521718 | 0.944216 |
| 1181 | 0.987485 | 0.561586 | -0.4259 | 0.429635 | 0.517011 | 0.962936 |
| 1182 | 0.985802 | 0.499189 | -0.48661 | 0.424831 | 0.521718 | 0.944216 |
| 1183 | 0.986965 | 0.542034 | -0.44493 | 0.428164 | 0.518452 | 0.95717 |
| 1184 | 0.974294 | 0.582579 | -0.39172 | 0.432028 | 0.514591 | 0.972382 |
| 1185 | 0.974139 | 0.617137 | -0.357 | 0.434592 | 0.512079 | 0.982586 |
| 1186 | 0.986965 | 0.542034 | -0.44493 | 0.428164 | 0.518452 | 0.95717 |
| 1187 | 0.806284 | 0.538028 | -0.26826 | 0.439345 | 0.506419 | 1.00175 |
| 1188 | 0.806702 | 0.558688 | -0.24801 | 0.440928 | 0.504893 | 1.00821 |
| 1189 | 0.918493 | 0.533206 | -0.38529 | 0.431717 | 0.514568 | 0.971148 |
| 1190 | 0.932982 | 0.692761 | -0.24022 | 0.442525 | 0.504113 | 1.01476 |
| 1191 | 0.937479 | 0.563894 | -0.37359 | 0.432905 | 0.513523 | 0.975859 |
| 1192 | 0.986965 | 0.542034 | -0.44493 | 0.428164 | 0.518452 | 0.95717 |
| 1193 | 0.974294 | 0.582579 | -0.39172 | 0.432028 | 0.514591 | 0.972382 |
| 1194 | 0.961357 | 0.636079 | -0.32528 | 0.436747 | 0.509902 | 0.991236 |
| 1195 | 0.932982 | 0.692761 | -0.24022 | 0.442525 | 0.504113 | 1.01476 |
| 1196 | 0.987485 | 0.561586 | -0.4259 | 0.429635 | 0.517011 | 0.962936 |
| 1197 | 0.98943 | 0.63651 | -0.35292 | 0.435046 | 0.511714 | 0.984402 |
| 1198 | 0.937451 | 0.56278 | -0.37467 | 0.432817 | 0.513608 | 0.975512 |
| 1199 | 0.806284 | 0.538028 | -0.26826 | 0.439345 | 0.506419 | 1.00175 |
| 1200 | 0.937451 | 0.56278 | -0.37467 | 0.432817 | 0.513608 | 0.975512 |
| 1201 | 0.922331 | 0.696783 | -0.22555 | 0.443477 | 0.50313 | 1.01868 |
| 1202 | 0.97633 | 0.612906 | -0.36342 | 0.434145 | 0.512528 | 0.9808 |
| 1203 | 0.806702 | 0.558688 | -0.24801 | 0.440928 | 0.504893 | 1.00821 |
| 1204 | 0.932982 | 0.692761 | -0.24022 | 0.442525 | 0.504113 | 1.01476 |
| 1205 | 0.803087 | 0.684908 | -0.11818 | 0.450428 | 0.495711 | 1.04774 |
| 1206 | 0.97633 | 0.612906 | -0.36342 | 0.434145 | 0.512528 | 0.9808 |
| 1207 | 0.974294 | 0.582579 | -0.39172 | 0.432028 | 0.514591 | 0.972382 |
| 1208 | 0.970786 | 0.488825 | -0.48196 | 0.424896 | 0.521564 | 0.944466 |
| 1209 | 0.974294 | 0.582579 | -0.39172 | 0.432028 | 0.514591 | 0.972382 |
| 1210 | 0.970786 | 0.488825 | -0.48196 | 0.424896 | 0.521564 | 0.944466 |
| 1211 | 0.974294 | 0.582579 | -0.39172 | 0.432028 | 0.514591 | 0.972382 |
| 1212 | 0.788397 | 0.550531 | -0.23787 | 0.441528 | 0.504185 | 1.01067 |
| 1213 | 0.987485 | 0.561586 | -0.4259 | 0.429635 | 0.517011 | 0.962936 |
| 1214 | 0.788397 | 0.550531 | -0.23787 | 0.441528 | 0.504185 | 1.01067 |
| 1215 | 0.917433 | 0.490551 | -0.42688 | 0.428342 | 0.517857 | 0.957865 |
| 1216 | 0.937451 | 0.56278 | -0.37467 | 0.432817 | 0.513608 | 0.975512 |
| 1217 | 0.932982 | 0.692761 | -0.24022 | 0.442525 | 0.504113 | 1.01476 |
| 1218 | 0.990556 | 0.681067 | -0.30949 | 0.438125 | 0.508702 | 0.996803 |
| 1219 | 0.987485 | 0.561586 | -0.4259 | 0.429635 | 0.517011 | 0.962936 |
| 1220 | 0.971993 | 0.533855 | -0.43814 | 0.428445 | 0.51809 | 0.95827 |
| 1221 | 0.917433 | 0.490551 | -0.42688 | 0.428342 | 0.517857 | 0.957865 |
| 1222 | 0.917433 | 0.490551 | -0.42688 | 0.428342 | 0.517857 | 0.957865 |
| 1223 | 0.987409 | 0.558677 | -0.42873 | 0.429422 | 0.517219 | 0.962101 |
| 1224 | 0.987485 | 0.561586 | -0.4259 | 0.429635 | 0.517011 | 0.962936 |
| 1225 | 0.991861 | 0.499837 | -0.49202 | 0.424517 | 0.522062 | 0.943004 |
| 1226 | 0.987485 | 0.561586 | -0.4259 | 0.429635 | 0.517011 | 0.962936 |
| 1227 | 0.787123 | 0.486854 | -0.30027 | 0.436473 | 0.509055 | 0.990134 |
| 1228 | 0.986965 | 0.542034 | -0.44493 | 0.428164 | 0.518452 | 0.95717 |
| 1229 | 0.987485 | 0.561586 | -0.4259 | 0.429635 | 0.517011 | 0.962936 |
| 1230 | 0.987409 | 0.558677 | -0.42873 | 0.429422 | 0.517219 | 0.962101 |
| 1231 | 0.918724 | 0.542619 | -0.3761 | 0.432444 | 0.51386 | 0.974028 |
| 1232 | 0.987224 | 0.551719 | -0.43551 | 0.4289 | 0.517731 | 0.960052 |
| 1233 | 0.919878 | 0.590742 | -0.32914 | 0.436031 | 0.510368 | 0.988356 |
| 1234 | 0.98943 | 0.63651 | -0.35292 | 0.435046 | 0.511714 | 0.984402 |
| 1235 | 0.918493 | 0.533206 | -0.38529 | 0.431717 | 0.514568 | 0.971148 |
| 1236 | 0.974139 | 0.617137 | -0.357 | 0.434592 | 0.512079 | 0.982586 |
| 1237 | 0.937451 | 0.56278 | -0.37467 | 0.432817 | 0.513608 | 0.975512 |
| 1238 | 0.985802 | 0.499189 | -0.48661 | 0.424831 | 0.521718 | 0.944216 |
| 1239 | 0.990556 | 0.681067 | -0.30949 | 0.438125 | 0.508702 | 0.996803 |
| 1240 | 0.922331 | 0.696783 | -0.22555 | 0.443477 | 0.50313 | 1.01868 |
| 1241 | 0.98943 | 0.63651 | -0.35292 | 0.435046 | 0.511714 | 0.984402 |
| 1242 | 0.97633 | 0.612906 | -0.36342 | 0.434145 | 0.512528 | 0.9808 |
| 1243 | 0.970786 | 0.488825 | -0.48196 | 0.424896 | 0.521564 | 0.944466 |
| 1244 | 0.970786 | 0.488825 | -0.48196 | 0.424896 | 0.521564 | 0.944466 |
| 1245 | 0.973488 | 0.591491 | -0.382 | 0.432745 | 0.513885 | 0.975223 |
| 1246 | 0.986965 | 0.542034 | -0.44493 | 0.428164 | 0.518452 | 0.95717 |
| 1247 | 0.918956 | 0.552208 | -0.36675 | 0.433168 | 0.513155 | 0.976908 |
| 1248 | 0.990556 | 0.681067 | -0.30949 | 0.438125 | 0.508702 | 0.996803 |
| 1249 | 0.932982 | 0.692761 | -0.24022 | 0.442525 | 0.504113 | 1.01476 |
| 1250 | 0.936932 | 0.54188 | -0.39505 | 0.431239 | 0.515148 | 0.969257 |
| 1251 | 0.919878 | 0.590742 | -0.32914 | 0.436031 | 0.510368 | 0.988356 |
| 1252 | 0.987485 | 0.561586 | -0.4259 | 0.429635 | 0.517011 | 0.962936 |
| 1253 | 0.937451 | 0.56278 | -0.37467 | 0.432817 | 0.513608 | 0.975512 |
| 1254 | 0.987409 | 0.558677 | -0.42873 | 0.429422 | 0.517219 | 0.962101 |
| 1255 | 0.986965 | 0.542034 | -0.44493 | 0.428164 | 0.518452 | 0.95717 |
| 1256 | 0.985802 | 0.499189 | -0.48661 | 0.424831 | 0.521718 | 0.944216 |
| 1257 | 0.985802 | 0.499189 | -0.48661 | 0.424831 | 0.521718 | 0.944216 |
| 1258 | 0.889845 | 0.592529 | -0.29732 | 0.438067 | 0.508207 | 0.996567 |
| 1259 | 0.986965 | 0.542034 | -0.44493 | 0.428164 | 0.518452 | 0.95717 |
| 1260 | 1.20242 | 12.3037 | 11.1013 | 0.959018 | 0.002168 | 29.9149 |
| 1261 | 0.987485 | 0.561586 | -0.4259 | 0.429635 | 0.517011 | 0.962936 |
| 1262 | 0.917433 | 0.490551 | -0.42688 | 0.428342 | 0.517857 | 0.957865 |
| 1263 | 0.970786 | 0.488825 | -0.48196 | 0.424896 | 0.521564 | 0.944466 |
| 1264 | 0.957872 | 0.499115 | -0.45876 | 0.426532 | 0.519881 | 0.950809 |
| 1265 | 0.987485 | 0.561586 | -0.4259 | 0.429635 | 0.517011 | 0.962936 |
| 1266 | 0.787123 | 0.486854 | -0.30027 | 0.436473 | 0.509055 | 0.990134 |
| 1267 | 0.987485 | 0.561586 | -0.4259 | 0.429635 | 0.517011 | 0.962936 |
| 1268 | 0.787123 | 0.486854 | -0.30027 | 0.436473 | 0.509055 | 0.990134 |
| 1269 | 0.918956 | 0.552208 | -0.36675 | 0.433168 | 0.513155 | 0.976908 |
| 1270 | 0.936932 | 0.54188 | -0.39505 | 0.431239 | 0.515148 | 0.969257 |
| 1271 | 0.917433 | 0.490551 | -0.42688 | 0.428342 | 0.517857 | 0.957865 |
| 1272 | 0.987485 | 0.561586 | -0.4259 | 0.429635 | 0.517011 | 0.962936 |
| 1273 | 0.919878 | 0.590742 | -0.32914 | 0.436031 | 0.510368 | 0.988356 |
| 1274 | 0.987485 | 0.561586 | -0.4259 | 0.429635 | 0.517011 | 0.962936 |
| 1275 | 0.918724 | 0.542619 | -0.3761 | 0.432444 | 0.51386 | 0.974028 |
| 1276 | 0.986965 | 0.542034 | -0.44493 | 0.428164 | 0.518452 | 0.95717 |
| 1277 | 0.918956 | 0.552208 | -0.36675 | 0.433168 | 0.513155 | 0.976908 |
| 1278 | 0.972242 | 0.543285 | -0.42896 | 0.429169 | 0.517382 | 0.961107 |
| 1279 | 0.922331 | 0.696783 | -0.22555 | 0.443477 | 0.50313 | 1.01868 |
| 1280 | 0.987409 | 0.558677 | -0.42873 | 0.429422 | 0.517219 | 0.962101 |
| 1281 | 0.987485 | 0.561586 | -0.4259 | 0.429635 | 0.517011 | 0.962936 |
| 1282 | 0.986965 | 0.542034 | -0.44493 | 0.428164 | 0.518452 | 0.95717 |
| 1283 | 0.987485 | 0.561586 | -0.4259 | 0.429635 | 0.517011 | 0.962936 |
| 1284 | 0.986965 | 0.542034 | -0.44493 | 0.428164 | 0.518452 | 0.95717 |
| 1285 | 0.986965 | 0.542034 | -0.44493 | 0.428164 | 0.518452 | 0.95717 |
| 1286 | 0.986965 | 0.542034 | -0.44493 | 0.428164 | 0.518452 | 0.95717 |
| 1287 | 0.986965 | 0.542034 | -0.44493 | 0.428164 | 0.518452 | 0.95717 |
| 1288 | 0.937451 | 0.56278 | -0.37467 | 0.432817 | 0.513608 | 0.975512 |
| 1289 | 0.987409 | 0.558677 | -0.42873 | 0.429422 | 0.517219 | 0.962101 |
| 1290 | 0.987485 | 0.561586 | -0.4259 | 0.429635 | 0.517011 | 0.962936 |
| 1291 | 0.987485 | 0.561586 | -0.4259 | 0.429635 | 0.517011 | 0.962936 |
| 1292 | 0.986965 | 0.542034 | -0.44493 | 0.428164 | 0.518452 | 0.95717 |
| 1293 | 0.957872 | 0.499115 | -0.45876 | 0.426532 | 0.519881 | 0.950809 |
| 1294 | 0.985802 | 0.499189 | -0.48661 | 0.424831 | 0.521718 | 0.944216 |
| 1295 | 0.987485 | 0.561586 | -0.4259 | 0.429635 | 0.517011 | 0.962936 |
| 1296 | 0.97633 | 0.612906 | -0.36342 | 0.434145 | 0.512528 | 0.9808 |
| 1297 | 0.974294 | 0.582579 | -0.39172 | 0.432028 | 0.514591 | 0.972382 |
| 1298 | 0.987409 | 0.558677 | -0.42873 | 0.429422 | 0.517219 | 0.962101 |
| 1299 | 0.918956 | 0.552208 | -0.36675 | 0.433168 | 0.513155 | 0.976908 |
| 1300 | 0.806702 | 0.558688 | -0.24801 | 0.440928 | 0.504893 | 1.00821 |
| 1301 | 0.973488 | 0.591491 | -0.382 | 0.432745 | 0.513885 | 0.975223 |
| 1302 | 6.94464 | 0.803557 | -6.14108 | 0.067322 | 0.90106 | 0.092273 |
| 1303 | 0.986965 | 0.542034 | -0.44493 | 0.428164 | 0.518452 | 0.95717 |
| 1304 | 0.974294 | 0.582579 | -0.39172 | 0.432028 | 0.514591 | 0.972382 |
| 1305 | 0.971993 | 0.533855 | -0.43814 | 0.428445 | 0.51809 | 0.95827 |
| 1306 | 0.973488 | 0.591491 | -0.382 | 0.432745 | 0.513885 | 0.975223 |
| 1307 | 0.919878 | 0.590742 | -0.32914 | 0.436031 | 0.510368 | 0.988356 |
| 1308 | 0.932982 | 0.692761 | -0.24022 | 0.442525 | 0.504113 | 1.01476 |
| 1309 | 0.986965 | 0.542034 | -0.44493 | 0.428164 | 0.518452 | 0.95717 |
| 1310 | 0.806725 | 0.559789 | -0.24694 | 0.441016 | 0.504808 | 1.00857 |
| 1311 | 0.974139 | 0.617137 | -0.357 | 0.434592 | 0.512079 | 0.982586 |
| 1312 | 0.985802 | 0.499189 | -0.48661 | 0.424831 | 0.521718 | 0.944216 |
| 1313 | 0.987485 | 0.561586 | -0.4259 | 0.429635 | 0.517011 | 0.962936 |
| 1314 | 0.974294 | 0.582579 | -0.39172 | 0.432028 | 0.514591 | 0.972382 |
| 1315 | 0.859272 | 0.615434 | -0.24384 | 0.441734 | 0.50446 | 1.01151 |
| 1316 | 0.937451 | 0.56278 | -0.37467 | 0.432817 | 0.513608 | 0.975512 |
| 1317 | 0.986965 | 0.542034 | -0.44493 | 0.428164 | 0.518452 | 0.95717 |
| 1318 | 0.937479 | 0.563894 | -0.37359 | 0.432905 | 0.513523 | 0.975859 |
| 1319 | 0.996136 | 0.661383 | -0.33475 | 0.436404 | 0.510416 | 0.989857 |
| 1320 | 0.987485 | 0.561586 | -0.4259 | 0.429635 | 0.517011 | 0.962936 |
| 1321 | 0.919878 | 0.590742 | -0.32914 | 0.436031 | 0.510368 | 0.988356 |
| 1322 | 0.98943 | 0.63651 | -0.35292 | 0.435046 | 0.511714 | 0.984402 |
| 1323 | 0.974294 | 0.582579 | -0.39172 | 0.432028 | 0.514591 | 0.972382 |
| 1324 | 0.937451 | 0.56278 | -0.37467 | 0.432817 | 0.513608 | 0.975512 |
| 1325 | 0.806725 | 0.559789 | -0.24694 | 0.441016 | 0.504808 | 1.00857 |
| 1326 | 0.990556 | 0.681067 | -0.30949 | 0.438125 | 0.508702 | 0.996803 |
| 1327 | 0.973488 | 0.591491 | -0.382 | 0.432745 | 0.513885 | 0.975223 |
| 1328 | 0.974294 | 0.582579 | -0.39172 | 0.432028 | 0.514591 | 0.972382 |
| 1329 | 0.958316 | 0.515962 | -0.44235 | 0.427866 | 0.518577 | 0.956005 |
| 1330 | 0.918956 | 0.552208 | -0.36675 | 0.433168 | 0.513155 | 0.976908 |
| 1331 | 0.789141 | 0.5889 | -0.20024 | 0.444412 | 0.50141 | 1.02255 |
| 1332 | 0.958316 | 0.515962 | -0.44235 | 0.427866 | 0.518577 | 0.956005 |
| 1333 | 0.937479 | 0.563894 | -0.37359 | 0.432905 | 0.513523 | 0.975859 |
| 1334 | 0.958316 | 0.515962 | -0.44235 | 0.427866 | 0.518577 | 0.956005 |
| 1335 | 0.98943 | 0.63651 | -0.35292 | 0.435046 | 0.511714 | 0.984402 |
| 1336 | 0.803087 | 0.684908 | -0.11818 | 0.450428 | 0.495711 | 1.04774 |
| 1337 | 0.922331 | 0.696783 | -0.22555 | 0.443477 | 0.50313 | 1.01868 |
| 1338 | 0.937451 | 0.56278 | -0.37467 | 0.432817 | 0.513608 | 0.975512 |
| 1339 | 0.98943 | 0.63651 | -0.35292 | 0.435046 | 0.511714 | 0.984402 |
| 1340 | 0.869858 | 0.532409 | -0.33745 | 0.434745 | 0.511309 | 0.9832 |
| 1341 | 0.985802 | 0.499189 | -0.48661 | 0.424831 | 0.521718 | 0.944216 |
| 1342 | 0.987224 | 0.551719 | -0.43551 | 0.4289 | 0.517731 | 0.960052 |
| 1343 | 0.974294 | 0.582579 | -0.39172 | 0.432028 | 0.514591 | 0.972382 |
| 1344 | 0.972492 | 0.55289 | -0.4196 | 0.429891 | 0.516675 | 0.963944 |
| 1345 | 0.97633 | 0.612906 | -0.36342 | 0.434145 | 0.512528 | 0.9808 |
| 1346 | 0.919878 | 0.590742 | -0.32914 | 0.436031 | 0.510368 | 0.988356 |
| 1347 | 0.937451 | 0.56278 | -0.37467 | 0.432817 | 0.513608 | 0.975512 |
| 1348 | 0.991861 | 0.499837 | -0.49202 | 0.424517 | 0.522062 | 0.943004 |
| 1349 | 0.917433 | 0.490551 | -0.42688 | 0.428342 | 0.517857 | 0.957865 |
| 1350 | 0.806702 | 0.558688 | -0.24801 | 0.440928 | 0.504893 | 1.00821 |
| 1351 | 0.985802 | 0.499189 | -0.48661 | 0.424831 | 0.521718 | 0.944216 |
| 1352 | 0.972492 | 0.55289 | -0.4196 | 0.429891 | 0.516675 | 0.963944 |
| 1353 | 0.806702 | 0.558688 | -0.24801 | 0.440928 | 0.504893 | 1.00821 |
| 1354 | 0.936932 | 0.54188 | -0.39505 | 0.431239 | 0.515148 | 0.969257 |
| 1355 | 0.937451 | 0.56278 | -0.37467 | 0.432817 | 0.513608 | 0.975512 |
| 1356 | 0.996136 | 0.661383 | -0.33475 | 0.436404 | 0.510416 | 0.989857 |
| 1357 | 0.956877 | 0.462146 | -0.49473 | 0.423501 | 0.522846 | 0.93909 |
| 1358 | 0.932982 | 0.692761 | -0.24022 | 0.442525 | 0.504113 | 1.01476 |
| 1359 | 0.987485 | 0.561586 | -0.4259 | 0.429635 | 0.517011 | 0.962936 |
| 1360 | 0.937451 | 0.56278 | -0.37467 | 0.432817 | 0.513608 | 0.975512 |
| 1361 | 0.937451 | 0.56278 | -0.37467 | 0.432817 | 0.513608 | 0.975512 |
| 1362 | 0.97633 | 0.612906 | -0.36342 | 0.434145 | 0.512528 | 0.9808 |
| 1363 | 0.985802 | 0.499189 | -0.48661 | 0.424831 | 0.521718 | 0.944216 |
| 1364 | 0.98943 | 0.63651 | -0.35292 | 0.435046 | 0.511714 | 0.984402 |
| 1365 | 0.937479 | 0.563894 | -0.37359 | 0.432905 | 0.513523 | 0.975859 |
| 1366 | 0.806702 | 0.558688 | -0.24801 | 0.440928 | 0.504893 | 1.00821 |
| 1367 | 0.806702 | 0.558688 | -0.24801 | 0.440928 | 0.504893 | 1.00821 |
| 1368 | 0.806702 | 0.558688 | -0.24801 | 0.440928 | 0.504893 | 1.00821 |
| 1369 | 0.97633 | 0.612906 | -0.36342 | 0.434145 | 0.512528 | 0.9808 |
| 1370 | 0.97633 | 0.612906 | -0.36342 | 0.434145 | 0.512528 | 0.9808 |
| 1371 | 0.937451 | 0.56278 | -0.37467 | 0.432817 | 0.513608 | 0.975512 |
| 1372 | 1.13813 | 9.89278 | 8.75464 | 0.938545 | 0.023456 | 19.5231 |
| 1373 | 0.974294 | 0.582579 | -0.39172 | 0.432028 | 0.514591 | 0.972382 |
| 1374 | 0.958316 | 0.515962 | -0.44235 | 0.427866 | 0.518577 | 0.956005 |
| 1375 | 0.937451 | 0.56278 | -0.37467 | 0.432817 | 0.513608 | 0.975512 |
| 1376 | 0.932982 | 0.692761 | -0.24022 | 0.442525 | 0.504113 | 1.01476 |
| 1377 | 0.918724 | 0.542619 | -0.3761 | 0.432444 | 0.51386 | 0.974028 |
| 1378 | 0.974294 | 0.582579 | -0.39172 | 0.432028 | 0.514591 | 0.972382 |
| 1379 | 0.922331 | 0.696783 | -0.22555 | 0.443477 | 0.50313 | 1.01868 |
| 1380 | 0.98943 | 0.63651 | -0.35292 | 0.435046 | 0.511714 | 0.984402 |
| 1381 | 0.922331 | 0.696783 | -0.22555 | 0.443477 | 0.50313 | 1.01868 |
| 1382 | 0.937451 | 0.56278 | -0.37467 | 0.432817 | 0.513608 | 0.975512 |
| 1383 | 0.806702 | 0.558688 | -0.24801 | 0.440928 | 0.504893 | 1.00821 |
| 1384 | 0.806702 | 0.558688 | -0.24801 | 0.440928 | 0.504893 | 1.00821 |
| 1385 | 0.986965 | 0.542034 | -0.44493 | 0.428164 | 0.518452 | 0.95717 |
| 1386 | 0.974294 | 0.582579 | -0.39172 | 0.432028 | 0.514591 | 0.972382 |
| 1387 | 0.918493 | 0.533206 | -0.38529 | 0.431717 | 0.514568 | 0.971148 |
| 1388 | 0.918807 | 5.89717 | 4.97836 | 0.846196 | 0.114092 | 7.03324 |
| 1389 | 0.985802 | 0.499189 | -0.48661 | 0.424831 | 0.521718 | 0.944216 |
| 1390 | 0.932982 | 0.692761 | -0.24022 | 0.442525 | 0.504113 | 1.01476 |
| 1391 | 0.937451 | 0.56278 | -0.37467 | 0.432817 | 0.513608 | 0.975512 |
| 1392 | 0.98943 | 0.63651 | -0.35292 | 0.435046 | 0.511714 | 0.984402 |
| 1393 | 0.919878 | 0.590742 | -0.32914 | 0.436031 | 0.510368 | 0.988356 |
| 1394 | 0.795202 | 0.498709 | -0.29649 | 0.436918 | 0.508685 | 0.991924 |
| 1395 | 0.918956 | 0.552208 | -0.36675 | 0.433168 | 0.513155 | 0.976908 |
| 1396 | 0.937451 | 0.56278 | -0.37467 | 0.432817 | 0.513608 | 0.975512 |
| 1397 | 0.919878 | 0.590742 | -0.32914 | 0.436031 | 0.510368 | 0.988356 |
| 1398 | 0.990556 | 0.681067 | -0.30949 | 0.438125 | 0.508702 | 0.996803 |
| 1399 | 0.937451 | 0.56278 | -0.37467 | 0.432817 | 0.513608 | 0.975512 |
| 1400 | 0.937451 | 0.56278 | -0.37467 | 0.432817 | 0.513608 | 0.975512 |
| 1401 | 0.985802 | 0.499189 | -0.48661 | 0.424831 | 0.521718 | 0.944216 |
| 1402 | 0.936932 | 0.54188 | -0.39505 | 0.431239 | 0.515148 | 0.969257 |
| 1403 | 0.918956 | 0.552208 | -0.36675 | 0.433168 | 0.513155 | 0.976908 |
| 1404 | 0.987485 | 0.561586 | -0.4259 | 0.429635 | 0.517011 | 0.962936 |
| 1405 | 0.923215 | 0.505613 | -0.4176 | 0.429214 | 0.517042 | 0.961284 |
| 1406 | 0.937451 | 0.56278 | -0.37467 | 0.432817 | 0.513608 | 0.975512 |
| 1407 | 0.937451 | 0.56278 | -0.37467 | 0.432817 | 0.513608 | 0.975512 |
| 1408 | 0.932982 | 0.692761 | -0.24022 | 0.442525 | 0.504113 | 1.01476 |
| 1409 | 0.974294 | 0.582579 | -0.39172 | 0.432028 | 0.514591 | 0.972382 |
| 1410 | 0.974294 | 0.582579 | -0.39172 | 0.432028 | 0.514591 | 0.972382 |
| 1411 | 0.991861 | 0.499837 | -0.49202 | 0.424517 | 0.522062 | 0.943004 |
| 1412 | 0.932982 | 0.692761 | -0.24022 | 0.442525 | 0.504113 | 1.01476 |
| 1413 | 0.973488 | 0.591491 | -0.382 | 0.432745 | 0.513885 | 0.975223 |
| 1414 | 0.936932 | 0.54188 | -0.39505 | 0.431239 | 0.515148 | 0.969257 |
| 1415 | 0.917433 | 0.490551 | -0.42688 | 0.428342 | 0.517857 | 0.957865 |
| 1416 | 0.937451 | 0.56278 | -0.37467 | 0.432817 | 0.513608 | 0.975512 |
| 1417 | 0.922331 | 0.696783 | -0.22555 | 0.443477 | 0.50313 | 1.01868 |
| 1418 | 0.958316 | 0.515962 | -0.44235 | 0.427866 | 0.518577 | 0.956005 |
| 1419 | 0.985802 | 0.499189 | -0.48661 | 0.424831 | 0.521718 | 0.944216 |
| 1420 | 0.987485 | 0.561586 | -0.4259 | 0.429635 | 0.517011 | 0.962936 |
| 1421 | 0.991861 | 0.499837 | -0.49202 | 0.424517 | 0.522062 | 0.943004 |
| 1422 | 0.987485 | 0.561586 | -0.4259 | 0.429635 | 0.517011 | 0.962936 |
| 1423 | 0.986965 | 0.542034 | -0.44493 | 0.428164 | 0.518452 | 0.95717 |
| 1424 | 0.803087 | 0.684908 | -0.11818 | 0.450428 | 0.495711 | 1.04774 |
| 1425 | 0.923215 | 0.505613 | -0.4176 | 0.429214 | 0.517042 | 0.961284 |
| 1426 | 0.937451 | 0.56278 | -0.37467 | 0.432817 | 0.513608 | 0.975512 |
| 1427 | 0.936932 | 0.54188 | -0.39505 | 0.431239 | 0.515148 | 0.969257 |
| 1428 | 0.973488 | 0.591491 | -0.382 | 0.432745 | 0.513885 | 0.975223 |
| 1429 | 0.917433 | 0.490551 | -0.42688 | 0.428342 | 0.517857 | 0.957865 |
| 1430 | 0.932982 | 0.692761 | -0.24022 | 0.442525 | 0.504113 | 1.01476 |
| 1431 | 0.932982 | 0.692761 | -0.24022 | 0.442525 | 0.504113 | 1.01476 |
| 1432 | 0.937451 | 0.56278 | -0.37467 | 0.432817 | 0.513608 | 0.975512 |
| 1433 | 0.919878 | 0.590742 | -0.32914 | 0.436031 | 0.510368 | 0.988356 |
| 1434 | 0.958316 | 0.515962 | -0.44235 | 0.427866 | 0.518577 | 0.956005 |
| 1435 | 0.987485 | 0.561586 | -0.4259 | 0.429635 | 0.517011 | 0.962936 |
| 1436 | 0.97633 | 0.612906 | -0.36342 | 0.434145 | 0.512528 | 0.9808 |
| 1437 | 0.986965 | 0.542034 | -0.44493 | 0.428164 | 0.518452 | 0.95717 |
| 1438 | 0.990556 | 0.681067 | -0.30949 | 0.438125 | 0.508702 | 0.996803 |
| 1439 | 0.990556 | 0.681067 | -0.30949 | 0.438125 | 0.508702 | 0.996803 |
| 1440 | 0.918493 | 0.533206 | -0.38529 | 0.431717 | 0.514568 | 0.971148 |
| 1441 | 0.961357 | 0.636079 | -0.32528 | 0.436747 | 0.509902 | 0.991236 |
| 1442 | 0.918493 | 0.533206 | -0.38529 | 0.431717 | 0.514568 | 0.971148 |
| 1443 | 0.974294 | 0.582579 | -0.39172 | 0.432028 | 0.514591 | 0.972382 |
| 1444 | 0.986965 | 0.542034 | -0.44493 | 0.428164 | 0.518452 | 0.95717 |
| 1445 | 0.987485 | 0.561586 | -0.4259 | 0.429635 | 0.517011 | 0.962936 |
| 1446 | 0.974294 | 0.582579 | -0.39172 | 0.432028 | 0.514591 | 0.972382 |
| 1447 | 0.986965 | 0.542034 | -0.44493 | 0.428164 | 0.518452 | 0.95717 |
| 1448 | 0.936932 | 0.54188 | -0.39505 | 0.431239 | 0.515148 | 0.969257 |
| 1449 | 0.788397 | 0.550531 | -0.23787 | 0.441528 | 0.504185 | 1.01067 |
| 1450 | 0.936932 | 0.54188 | -0.39505 | 0.431239 | 0.515148 | 0.969257 |
| 1451 | 0.961357 | 0.636079 | -0.32528 | 0.436747 | 0.509902 | 0.991236 |
| 1452 | 0.937451 | 0.56278 | -0.37467 | 0.432817 | 0.513608 | 0.975512 |
| 1453 | 0.974294 | 0.582579 | -0.39172 | 0.432028 | 0.514591 | 0.972382 |
| 1454 | 0.973775 | 0.602736 | -0.37104 | 0.433562 | 0.513086 | 0.978475 |
| 1455 | 0.787123 | 0.486854 | -0.30027 | 0.436473 | 0.509055 | 0.990134 |
| 1456 | 0.986965 | 0.542034 | -0.44493 | 0.428164 | 0.518452 | 0.95717 |
| 1457 | 0.987409 | 0.558677 | -0.42873 | 0.429422 | 0.517219 | 0.962101 |
| 1458 | 0.936932 | 0.54188 | -0.39505 | 0.431239 | 0.515148 | 0.969257 |
| 1459 | 0.970786 | 0.488825 | -0.48196 | 0.424896 | 0.521564 | 0.944466 |
| 1460 | 0.922331 | 0.696783 | -0.22555 | 0.443477 | 0.50313 | 1.01868 |
| 1461 | 0.919878 | 0.590742 | -0.32914 | 0.436031 | 0.510368 | 0.988356 |
| 1462 | 0.986965 | 0.542034 | -0.44493 | 0.428164 | 0.518452 | 0.95717 |
| 1463 | 0.991861 | 0.499837 | -0.49202 | 0.424517 | 0.522062 | 0.943004 |
| 1464 | 0.917433 | 0.490551 | -0.42688 | 0.428342 | 0.517857 | 0.957865 |
| 1465 | 0.973775 | 0.602736 | -0.37104 | 0.433562 | 0.513086 | 0.978475 |
| 1466 | 0.991861 | 0.499837 | -0.49202 | 0.424517 | 0.522062 | 0.943004 |
| 1467 | 0.787123 | 0.486854 | -0.30027 | 0.436473 | 0.509055 | 0.990134 |
| 1468 | 0.987485 | 0.561586 | -0.4259 | 0.429635 | 0.517011 | 0.962936 |
| 1469 | 0.922331 | 0.696783 | -0.22555 | 0.443477 | 0.50313 | 1.01868 |
| 1470 | 0.918724 | 0.542619 | -0.3761 | 0.432444 | 0.51386 | 0.974028 |
| 1471 | 0.918724 | 0.542619 | -0.3761 | 0.432444 | 0.51386 | 0.974028 |
| 1472 | 0.974139 | 0.617137 | -0.357 | 0.434592 | 0.512079 | 0.982586 |
| 1473 | 0.972242 | 0.543285 | -0.42896 | 0.429169 | 0.517382 | 0.961107 |
| 1474 | 0.974139 | 0.617137 | -0.357 | 0.434592 | 0.512079 | 0.982586 |
| 1475 | 0.98943 | 0.63651 | -0.35292 | 0.435046 | 0.511714 | 0.984402 |
| 1476 | 0.803087 | 0.684908 | -0.11818 | 0.450428 | 0.495711 | 1.04774 |
| 1477 | 0.918724 | 0.542619 | -0.3761 | 0.432444 | 0.51386 | 0.974028 |
| 1478 | 0.987409 | 0.558677 | -0.42873 | 0.429422 | 0.517219 | 0.962101 |
| 1479 | 0.937451 | 0.56278 | -0.37467 | 0.432817 | 0.513608 | 0.975512 |
| 1480 | 0.987485 | 0.561586 | -0.4259 | 0.429635 | 0.517011 | 0.962936 |
| 1481 | 0.974294 | 0.582579 | -0.39172 | 0.432028 | 0.514591 | 0.972382 |
| 1482 | 0.973488 | 0.591491 | -0.382 | 0.432745 | 0.513885 | 0.975223 |
| 1483 | 0.806702 | 0.558688 | -0.24801 | 0.440928 | 0.504893 | 1.00821 |
| 1484 | 0.919878 | 0.590742 | -0.32914 | 0.436031 | 0.510368 | 0.988356 |
| 1485 | 0.922331 | 0.696783 | -0.22555 | 0.443477 | 0.50313 | 1.01868 |
| 1486 | 0.996136 | 0.661383 | -0.33475 | 0.436404 | 0.510416 | 0.989857 |
| 1487 | 0.986965 | 0.542034 | -0.44493 | 0.428164 | 0.518452 | 0.95717 |
| 1488 | 0.918956 | 0.552208 | -0.36675 | 0.433168 | 0.513155 | 0.976908 |
| 1489 | 0.932982 | 0.692761 | -0.24022 | 0.442525 | 0.504113 | 1.01476 |
| 1490 | 0.932982 | 0.692761 | -0.24022 | 0.442525 | 0.504113 | 1.01476 |
| 1491 | 0.974139 | 0.617137 | -0.357 | 0.434592 | 0.512079 | 0.982586 |
| 1492 | 0.922331 | 0.696783 | -0.22555 | 0.443477 | 0.50313 | 1.01868 |
| 1493 | 0.788397 | 0.550531 | -0.23787 | 0.441528 | 0.504185 | 1.01067 |
| 1494 | 0.986965 | 0.542034 | -0.44493 | 0.428164 | 0.518452 | 0.95717 |
| 1495 | 0.974294 | 0.582579 | -0.39172 | 0.432028 | 0.514591 | 0.972382 |
| 1496 | 0.987224 | 0.551719 | -0.43551 | 0.4289 | 0.517731 | 0.960052 |
| 1497 | 0.991861 | 0.499837 | -0.49202 | 0.424517 | 0.522062 | 0.943004 |
| 1498 | 0.806702 | 0.558688 | -0.24801 | 0.440928 | 0.504893 | 1.00821 |
| 1499 | 0.98943 | 0.63651 | -0.35292 | 0.435046 | 0.511714 | 0.984402 |
| 1500 | 0.97633 | 0.612906 | -0.36342 | 0.434145 | 0.512528 | 0.9808 |
| 1501 | 0.987409 | 0.558677 | -0.42873 | 0.429422 | 0.517219 | 0.962101 |
| 1502 | 0.987485 | 0.561586 | -0.4259 | 0.429635 | 0.517011 | 0.962936 |
| 1503 | 1.10459 | 5.29426 | 4.18967 | 0.813636 | 0.140807 | 5.58111 |
| 1504 | 0.98943 | 0.63651 | -0.35292 | 0.435046 | 0.511714 | 0.984402 |
| 1505 | 0.972242 | 0.543285 | -0.42896 | 0.429169 | 0.517382 | 0.961107 |
| 1506 | 0.936932 | 0.54188 | -0.39505 | 0.431239 | 0.515148 | 0.969257 |
| 1507 | 0.986965 | 0.542034 | -0.44493 | 0.428164 | 0.518452 | 0.95717 |
| 1508 | 0.857464 | 0.532463 | -0.325 | 0.435545 | 0.510451 | 0.986402 |
| 1509 | 0.936932 | 0.54188 | -0.39505 | 0.431239 | 0.515148 | 0.969257 |
| 1510 | 0.987409 | 0.558677 | -0.42873 | 0.429422 | 0.517219 | 0.962101 |
| 1511 | 0.974139 | 0.617137 | -0.357 | 0.434592 | 0.512079 | 0.982586 |
| 1512 | 0.936932 | 0.54188 | -0.39505 | 0.431239 | 0.515148 | 0.969257 |
| 1513 | 0.996136 | 0.661383 | -0.33475 | 0.436404 | 0.510416 | 0.989857 |
| 1514 | 0.806284 | 0.538028 | -0.26826 | 0.439345 | 0.506419 | 1.00175 |
| 1515 | 0.936932 | 0.54188 | -0.39505 | 0.431239 | 0.515148 | 0.969257 |
| 1516 | 0.996136 | 0.661383 | -0.33475 | 0.436404 | 0.510416 | 0.989857 |
| 1517 | 0.985802 | 0.499189 | -0.48661 | 0.424831 | 0.521718 | 0.944216 |
| 1518 | 0.857673 | 0.541857 | -0.31582 | 0.436274 | 0.509744 | 0.989332 |
| 1519 | 0.987409 | 0.558677 | -0.42873 | 0.429422 | 0.517219 | 0.962101 |
| 1520 | 0.972242 | 0.543285 | -0.42896 | 0.429169 | 0.517382 | 0.961107 |
| 1521 | 0.936932 | 0.54188 | -0.39505 | 0.431239 | 0.515148 | 0.969257 |
| 1522 | 0.788397 | 0.550531 | -0.23787 | 0.441528 | 0.504185 | 1.01067 |
| 1523 | 0.987409 | 0.558677 | -0.42873 | 0.429422 | 0.517219 | 0.962101 |
| 1524 | 0.972242 | 0.543285 | -0.42896 | 0.429169 | 0.517382 | 0.961107 |
| 1525 | 0.918956 | 0.552208 | -0.36675 | 0.433168 | 0.513155 | 0.976908 |
| 1526 | 0.97633 | 0.612906 | -0.36342 | 0.434145 | 0.512528 | 0.9808 |
| 1527 | 0.987485 | 0.561586 | -0.4259 | 0.429635 | 0.517011 | 0.962936 |
| 1528 | 0.987485 | 0.561586 | -0.4259 | 0.429635 | 0.517011 | 0.962936 |
| 1529 | 0.987485 | 0.561586 | -0.4259 | 0.429635 | 0.517011 | 0.962936 |
| 1530 | 0.987485 | 0.561586 | -0.4259 | 0.429635 | 0.517011 | 0.962936 |
| 1531 | 0.987485 | 0.561586 | -0.4259 | 0.429635 | 0.517011 | 0.962936 |
| 1532 | 0.987485 | 0.561586 | -0.4259 | 0.429635 | 0.517011 | 0.962936 |
| 1533 | 0.985802 | 0.499189 | -0.48661 | 0.424831 | 0.521718 | 0.944216 |
| 1534 | 0.985802 | 0.499189 | -0.48661 | 0.424831 | 0.521718 | 0.944216 |
| 1535 | 0.970786 | 0.488825 | -0.48196 | 0.424896 | 0.521564 | 0.944466 |
| 1536 | 0.985802 | 0.499189 | -0.48661 | 0.424831 | 0.521718 | 0.944216 |
| 1537 | 0.987485 | 0.561586 | -0.4259 | 0.429635 | 0.517011 | 0.962936 |
| 1538 | 0.958316 | 0.515962 | -0.44235 | 0.427866 | 0.518577 | 0.956005 |
| 1539 | 0.985802 | 0.499189 | -0.48661 | 0.424831 | 0.521718 | 0.944216 |
| 1540 | 1.19885 | 9.64112 | 8.44227 | 0.931266 | 0.027846 | 17.3202 |
| 1541 | 0.795202 | 0.498709 | -0.29649 | 0.436918 | 0.508685 | 0.991924 |
| 1542 | 0.972492 | 0.55289 | -0.4196 | 0.429891 | 0.516675 | 0.963944 |
| 1543 | 0.958316 | 0.515962 | -0.44235 | 0.427866 | 0.518577 | 0.956005 |
| 1544 | 0.97633 | 0.612906 | -0.36342 | 0.434145 | 0.512528 | 0.9808 |
| 1545 | 0.795202 | 0.498709 | -0.29649 | 0.436918 | 0.508685 | 0.991924 |
| 1546 | 0.918956 | 0.552208 | -0.36675 | 0.433168 | 0.513155 | 0.976908 |
| 1547 | 0.98943 | 0.63651 | -0.35292 | 0.435046 | 0.511714 | 0.984402 |
| 1548 | 0.990556 | 0.681067 | -0.30949 | 0.438125 | 0.508702 | 0.996803 |
| 1549 | 0.918724 | 0.542619 | -0.3761 | 0.432444 | 0.51386 | 0.974028 |
| 1550 | 0.806725 | 0.559789 | -0.24694 | 0.441016 | 0.504808 | 1.00857 |
| 1551 | 0.892729 | 0.619301 | -0.27343 | 0.439864 | 0.506478 | 1.00387 |
| 1552 | 0.956877 | 0.462146 | -0.49473 | 0.423501 | 0.522846 | 0.93909 |
| 1553 | 0.937451 | 0.56278 | -0.37467 | 0.432817 | 0.513608 | 0.975512 |
| 1554 | 0.795202 | 0.498709 | -0.29649 | 0.436918 | 0.508685 | 0.991924 |
| 1555 | 0.936932 | 0.54188 | -0.39505 | 0.431239 | 0.515148 | 0.969257 |
| 1556 | 0.795202 | 0.498709 | -0.29649 | 0.436918 | 0.508685 | 0.991924 |
| 1557 | 0.937451 | 0.56278 | -0.37467 | 0.432817 | 0.513608 | 0.975512 |
| 1558 | 0.917433 | 0.490551 | -0.42688 | 0.428342 | 0.517857 | 0.957865 |
| 1559 | 0.918724 | 0.542619 | -0.3761 | 0.432444 | 0.51386 | 0.974028 |
| 1560 | 0.961357 | 0.636079 | -0.32528 | 0.436747 | 0.509902 | 0.991236 |
| 1561 | 0.918956 | 0.552208 | -0.36675 | 0.433168 | 0.513155 | 0.976908 |
| 1562 | 0.932982 | 0.692761 | -0.24022 | 0.442525 | 0.504113 | 1.01476 |
| 1563 | 0.937451 | 0.56278 | -0.37467 | 0.432817 | 0.513608 | 0.975512 |
| 1564 | 0.917433 | 0.490551 | -0.42688 | 0.428342 | 0.517857 | 0.957865 |
| 1565 | 0.936932 | 0.54188 | -0.39505 | 0.431239 | 0.515148 | 0.969257 |
| 1566 | 1.07044 | 5.74668 | 4.67624 | 0.829404 | 0.126653 | 6.21509 |
| 1567 | 0.98943 | 0.63651 | -0.35292 | 0.435046 | 0.511714 | 0.984402 |
| 1568 | 0.991861 | 0.499837 | -0.49202 | 0.424517 | 0.522062 | 0.943004 |
| 1569 | 0.970786 | 0.488825 | -0.48196 | 0.424896 | 0.521564 | 0.944466 |
| 1570 | 0.806284 | 0.538028 | -0.26826 | 0.439345 | 0.506419 | 1.00175 |
| 1571 | 0.937451 | 0.56278 | -0.37467 | 0.432817 | 0.513608 | 0.975512 |
| 1572 | 0.98943 | 0.63651 | -0.35292 | 0.435046 | 0.511714 | 0.984402 |
| 1573 | 0.917433 | 0.490551 | -0.42688 | 0.428342 | 0.517857 | 0.957865 |
| 1574 | 0.985802 | 0.499189 | -0.48661 | 0.424831 | 0.521718 | 0.944216 |
| 1575 | 0.974139 | 0.617137 | -0.357 | 0.434592 | 0.512079 | 0.982586 |
| 1576 | 0.987409 | 0.558677 | -0.42873 | 0.429422 | 0.517219 | 0.962101 |
| 1577 | 0.991861 | 0.499837 | -0.49202 | 0.424517 | 0.522062 | 0.943004 |
| 1578 | 0.936932 | 0.54188 | -0.39505 | 0.431239 | 0.515148 | 0.969257 |
| 1579 | 0.803087 | 0.684908 | -0.11818 | 0.450428 | 0.495711 | 1.04774 |
| 1580 | 0.937451 | 0.56278 | -0.37467 | 0.432817 | 0.513608 | 0.975512 |
| 1581 | 0.974139 | 0.617137 | -0.357 | 0.434592 | 0.512079 | 0.982586 |
| 1582 | 0.889845 | 0.592529 | -0.29732 | 0.438067 | 0.508207 | 0.996567 |
| 1583 | 0.936932 | 0.54188 | -0.39505 | 0.431239 | 0.515148 | 0.969257 |
| 1584 | 0.98943 | 0.63651 | -0.35292 | 0.435046 | 0.511714 | 0.984402 |
| 1585 | 0.869858 | 0.532409 | -0.33745 | 0.434745 | 0.511309 | 0.9832 |
| 1586 | 0.922331 | 0.696783 | -0.22555 | 0.443477 | 0.50313 | 1.01868 |
| 1587 | 0.958991 | 0.541922 | -0.41707 | 0.429875 | 0.516613 | 0.963881 |
| 1588 | 0.972242 | 0.543285 | -0.42896 | 0.429169 | 0.517382 | 0.961107 |
| 1589 | 0.974294 | 0.582579 | -0.39172 | 0.432028 | 0.514591 | 0.972382 |
| 1590 | 0.987485 | 0.561586 | -0.4259 | 0.429635 | 0.517011 | 0.962936 |
| 1591 | 0.918493 | 0.533206 | -0.38529 | 0.431717 | 0.514568 | 0.971148 |
| 1592 | 0.970786 | 0.488825 | -0.48196 | 0.424896 | 0.521564 | 0.944466 |
| 1593 | 0.987409 | 0.558677 | -0.42873 | 0.429422 | 0.517219 | 0.962101 |
| 1594 | 0.987485 | 0.561586 | -0.4259 | 0.429635 | 0.517011 | 0.962936 |
| 1595 | 0.986965 | 0.542034 | -0.44493 | 0.428164 | 0.518452 | 0.95717 |
| 1596 | 0.991861 | 0.499837 | -0.49202 | 0.424517 | 0.522062 | 0.943004 |
| 1597 | 0.987409 | 0.558677 | -0.42873 | 0.429422 | 0.517219 | 0.962101 |
| 1598 | 0.917433 | 0.490551 | -0.42688 | 0.428342 | 0.517857 | 0.957865 |
| 1599 | 0.986965 | 0.542034 | -0.44493 | 0.428164 | 0.518452 | 0.95717 |
| 1600 | 0.96093 | 0.618709 | -0.34222 | 0.435524 | 0.511096 | 0.986318 |
| 1601 | 0.970786 | 0.488825 | -0.48196 | 0.424896 | 0.521564 | 0.944466 |
| 1602 | 0.936932 | 0.54188 | -0.39505 | 0.431239 | 0.515148 | 0.969257 |
| 1603 | 0.996136 | 0.661383 | -0.33475 | 0.436404 | 0.510416 | 0.989857 |
| 1604 | 0.985802 | 0.499189 | -0.48661 | 0.424831 | 0.521718 | 0.944216 |
| 1605 | 0.806284 | 0.538028 | -0.26826 | 0.439345 | 0.506419 | 1.00175 |
| 1606 | 0.987409 | 0.558677 | -0.42873 | 0.429422 | 0.517219 | 0.962101 |
| 1607 | 0.985802 | 0.499189 | -0.48661 | 0.424831 | 0.521718 | 0.944216 |
| 1608 | 0.918493 | 0.533206 | -0.38529 | 0.431717 | 0.514568 | 0.971148 |
| 1609 | 0.788397 | 0.550533 | -0.23786 | 0.441528 | 0.504185 | 1.01067 |
| 1610 | 0.987409 | 0.558677 | -0.42873 | 0.429422 | 0.517219 | 0.962101 |
| 1611 | 0.917433 | 0.490551 | -0.42688 | 0.428342 | 0.517857 | 0.957865 |
| 1612 | 0.936932 | 0.54188 | -0.39505 | 0.431239 | 0.515148 | 0.969257 |
| 1613 | 0.970786 | 0.488825 | -0.48196 | 0.424896 | 0.521564 | 0.944466 |
| 1614 | 0.974294 | 0.582579 | -0.39172 | 0.432028 | 0.514591 | 0.972382 |
| 1615 | 0.869858 | 0.532409 | -0.33745 | 0.434745 | 0.511309 | 0.9832 |
| 1616 | 0.919878 | 0.590742 | -0.32914 | 0.436031 | 0.510368 | 0.988356 |
| 1617 | 0.795202 | 0.498709 | -0.29649 | 0.436918 | 0.508685 | 0.991924 |
| 1618 | 0.987485 | 0.561586 | -0.4259 | 0.429635 | 0.517011 | 0.962936 |
| 1619 | 0.923215 | 0.505613 | -0.4176 | 0.429214 | 0.517042 | 0.961284 |
| 1620 | 0.986965 | 0.542034 | -0.44493 | 0.428164 | 0.518452 | 0.95717 |
| 1621 | 0.987485 | 0.561586 | -0.4259 | 0.429635 | 0.517011 | 0.962936 |
| 1622 | 0.974139 | 0.617137 | -0.357 | 0.434592 | 0.512079 | 0.982586 |
| 1623 | 0.932982 | 0.692761 | -0.24022 | 0.442525 | 0.504113 | 1.01476 |
| 1624 | 0.957872 | 0.499115 | -0.45876 | 0.426532 | 0.519881 | 0.950809 |
| 1625 | 0.987485 | 0.561586 | -0.4259 | 0.429635 | 0.517011 | 0.962936 |
| 1626 | 0.986965 | 0.542034 | -0.44493 | 0.428164 | 0.518452 | 0.95717 |
| 1627 | 0.957872 | 0.499115 | -0.45876 | 0.426532 | 0.519881 | 0.950809 |
| 1628 | 0.806702 | 0.558688 | -0.24801 | 0.440928 | 0.504893 | 1.00821 |
| 1629 | 0.987409 | 0.558677 | -0.42873 | 0.429422 | 0.517219 | 0.962101 |
| 1630 | 0.937451 | 0.56278 | -0.37467 | 0.432817 | 0.513608 | 0.975512 |
| 1631 | 0.932982 | 0.692761 | -0.24022 | 0.442525 | 0.504113 | 1.01476 |
| 1632 | 0.990556 | 0.681067 | -0.30949 | 0.438125 | 0.508702 | 0.996803 |
| 1633 | 0.987485 | 0.561586 | -0.4259 | 0.429635 | 0.517011 | 0.962936 |
| 1634 | 0.922331 | 0.696783 | -0.22555 | 0.443477 | 0.50313 | 1.01868 |
| 1635 | 0.972492 | 0.55289 | -0.4196 | 0.429891 | 0.516675 | 0.963944 |
| 1636 | 0.806284 | 0.538028 | -0.26826 | 0.439345 | 0.506419 | 1.00175 |
| 1637 | 0.991861 | 0.499837 | -0.49202 | 0.424517 | 0.522062 | 0.943004 |
| 1638 | 0.936932 | 0.54188 | -0.39505 | 0.431239 | 0.515148 | 0.969257 |
| 1639 | 0.918493 | 0.533206 | -0.38529 | 0.431717 | 0.514568 | 0.971148 |
| 1640 | 0.987409 | 0.558677 | -0.42873 | 0.429422 | 0.517219 | 0.962101 |
| 1641 | 0.987485 | 0.561586 | -0.4259 | 0.429635 | 0.517011 | 0.962936 |
| 1642 | 0.97633 | 0.612906 | -0.36342 | 0.434145 | 0.512528 | 0.9808 |
| 1643 | 0.937479 | 0.563894 | -0.37359 | 0.432905 | 0.513523 | 0.975859 |
| 1644 | 0.936932 | 0.54188 | -0.39505 | 0.431239 | 0.515148 | 0.969257 |
| 1645 | 0.987409 | 0.558677 | -0.42873 | 0.429422 | 0.517219 | 0.962101 |
| 1646 | 0.986965 | 0.542034 | -0.44493 | 0.428164 | 0.518452 | 0.95717 |
| 1647 | 0.986965 | 0.542034 | -0.44493 | 0.428164 | 0.518452 | 0.95717 |
| 1648 | 0.987485 | 0.561586 | -0.4259 | 0.429635 | 0.517011 | 0.962936 |
| 1649 | 0.987485 | 0.561586 | -0.4259 | 0.429635 | 0.517011 | 0.962936 |
| 1650 | 0.987485 | 0.561586 | -0.4259 | 0.429635 | 0.517011 | 0.962936 |
| 1651 | 0.919878 | 0.590742 | -0.32914 | 0.436031 | 0.510368 | 0.988356 |
| 1652 | 0.986965 | 0.542034 | -0.44493 | 0.428164 | 0.518452 | 0.95717 |
| 1653 | 0.974294 | 0.582579 | -0.39172 | 0.432028 | 0.514591 | 0.972382 |
| 1654 | 0.987409 | 0.558677 | -0.42873 | 0.429422 | 0.517219 | 0.962101 |
| 1655 | 0.806284 | 0.538028 | -0.26826 | 0.439345 | 0.506419 | 1.00175 |
| 1656 | 0.986965 | 0.542034 | -0.44493 | 0.428164 | 0.518452 | 0.95717 |
| 1657 | 0.97633 | 0.612906 | -0.36342 | 0.434145 | 0.512528 | 0.9808 |
| 1658 | 0.917433 | 0.490551 | -0.42688 | 0.428342 | 0.517857 | 0.957865 |
| 1659 | 0.936932 | 0.54188 | -0.39505 | 0.431239 | 0.515148 | 0.969257 |
| 1660 | 0.996136 | 0.661383 | -0.33475 | 0.436404 | 0.510416 | 0.989857 |
| 1661 | 0.985802 | 0.499189 | -0.48661 | 0.424831 | 0.521718 | 0.944216 |
| 1662 | 0.986965 | 0.542034 | -0.44493 | 0.428164 | 0.518452 | 0.95717 |
| 1663 | 0.996136 | 0.661383 | -0.33475 | 0.436404 | 0.510416 | 0.989857 |
| 1664 | 0.922331 | 0.696783 | -0.22555 | 0.443477 | 0.50313 | 1.01868 |
| 1665 | 0.974139 | 0.617137 | -0.357 | 0.434592 | 0.512079 | 0.982586 |
| 1666 | 0.987409 | 0.558677 | -0.42873 | 0.429422 | 0.517219 | 0.962101 |
| 1667 | 0.974139 | 0.617137 | -0.357 | 0.434592 | 0.512079 | 0.982586 |
| 1668 | 0.937451 | 0.56278 | -0.37467 | 0.432817 | 0.513608 | 0.975512 |
| 1669 | 0.972492 | 0.55289 | -0.4196 | 0.429891 | 0.516675 | 0.963944 |
| 1670 | 0.987409 | 0.558677 | -0.42873 | 0.429422 | 0.517219 | 0.962101 |
| 1671 | 0.917433 | 0.490551 | -0.42688 | 0.428342 | 0.517857 | 0.957865 |
| 1672 | 0.98943 | 0.63651 | -0.35292 | 0.435046 | 0.511714 | 0.984402 |
| 1673 | 0.922331 | 0.696783 | -0.22555 | 0.443477 | 0.50313 | 1.01868 |
| 1674 | 0.918956 | 0.552208 | -0.36675 | 0.433168 | 0.513155 | 0.976908 |
| 1675 | 0.923215 | 0.505613 | -0.4176 | 0.429214 | 0.517042 | 0.961284 |
| 1676 | 0.985802 | 0.499189 | -0.48661 | 0.424831 | 0.521718 | 0.944216 |
| 1677 | 0.923215 | 0.505613 | -0.4176 | 0.429214 | 0.517042 | 0.961284 |
| 1678 | 0.788397 | 0.550531 | -0.23787 | 0.441528 | 0.504185 | 1.01067 |
| 1679 | 0.990556 | 0.681067 | -0.30949 | 0.438125 | 0.508702 | 0.996803 |
| 1680 | 0.972492 | 0.55289 | -0.4196 | 0.429891 | 0.516675 | 0.963944 |
| 1681 | 0.97633 | 0.612906 | -0.36342 | 0.434145 | 0.512528 | 0.9808 |
| 1682 | 0.932982 | 0.692761 | -0.24022 | 0.442525 | 0.504113 | 1.01476 |
| 1683 | 0.918724 | 0.542619 | -0.3761 | 0.432444 | 0.51386 | 0.974028 |
| 1684 | 0.936932 | 0.54188 | -0.39505 | 0.431239 | 0.515148 | 0.969257 |
| 1685 | 0.98943 | 0.63651 | -0.35292 | 0.435046 | 0.511714 | 0.984402 |
| 1686 | 0.974294 | 0.582579 | -0.39172 | 0.432028 | 0.514591 | 0.972382 |
| 1687 | 0.917433 | 0.490551 | -0.42688 | 0.428342 | 0.517857 | 0.957865 |
| 1688 | 0.936932 | 0.54188 | -0.39505 | 0.431239 | 0.515148 | 0.969257 |
| 1689 | 0.98943 | 0.63651 | -0.35292 | 0.435046 | 0.511714 | 0.984402 |
| 1690 | 0.972242 | 0.543285 | -0.42896 | 0.429169 | 0.517382 | 0.961107 |
| 1691 | 0.987485 | 0.561586 | -0.4259 | 0.429635 | 0.517011 | 0.962936 |
| 1692 | 0.958991 | 0.541922 | -0.41707 | 0.429875 | 0.516613 | 0.963881 |
| 1693 | 0.937451 | 0.56278 | -0.37467 | 0.432817 | 0.513608 | 0.975512 |
| 1694 | 0.97633 | 0.612906 | -0.36342 | 0.434145 | 0.512528 | 0.9808 |
| 1695 | 0.990556 | 0.681067 | -0.30949 | 0.438125 | 0.508702 | 0.996803 |
| 1696 | 0.936932 | 0.54188 | -0.39505 | 0.431239 | 0.515148 | 0.969257 |
| 1697 | 0.795202 | 0.498709 | -0.29649 | 0.436918 | 0.508685 | 0.991924 |
| 1698 | 0.985802 | 0.499189 | -0.48661 | 0.424831 | 0.521718 | 0.944216 |
| 1699 | 0.936932 | 0.54188 | -0.39505 | 0.431239 | 0.515148 | 0.969257 |
| 1700 | 0.918956 | 0.552208 | -0.36675 | 0.433168 | 0.513155 | 0.976908 |
| 1701 | 0.936932 | 0.54188 | -0.39505 | 0.431239 | 0.515148 | 0.969257 |
| 1702 | 0.919878 | 0.590742 | -0.32914 | 0.436031 | 0.510368 | 0.988356 |
| 1703 | 0.987409 | 0.558677 | -0.42873 | 0.429422 | 0.517219 | 0.962101 |
| 1704 | 0.971993 | 0.533855 | -0.43814 | 0.428445 | 0.51809 | 0.95827 |
| 1705 | 0.990556 | 0.681067 | -0.30949 | 0.438125 | 0.508702 | 0.996803 |
| 1706 | 0.97633 | 0.612906 | -0.36342 | 0.434145 | 0.512528 | 0.9808 |
| 1707 | 0.972242 | 0.543285 | -0.42896 | 0.429169 | 0.517382 | 0.961107 |
| 1708 | 0.918493 | 0.533206 | -0.38529 | 0.431717 | 0.514568 | 0.971148 |
| 1709 | 0.987409 | 0.558677 | -0.42873 | 0.429422 | 0.517219 | 0.962101 |
| 1710 | 0.974139 | 0.617137 | -0.357 | 0.434592 | 0.512079 | 0.982586 |
| 1711 | 0.986965 | 0.542034 | -0.44493 | 0.428164 | 0.518452 | 0.95717 |
| 1712 | 0.936932 | 0.54188 | -0.39505 | 0.431239 | 0.515148 | 0.969257 |
| 1713 | 0.958316 | 0.515962 | -0.44235 | 0.427866 | 0.518577 | 0.956005 |
| 1714 | 0.987409 | 0.558677 | -0.42873 | 0.429422 | 0.517219 | 0.962101 |
| 1715 | 0.990556 | 0.681067 | -0.30949 | 0.438125 | 0.508702 | 0.996803 |
| 1716 | 0.936932 | 0.54188 | -0.39505 | 0.431239 | 0.515148 | 0.969257 |
| 1717 | 0.996136 | 0.661383 | -0.33475 | 0.436404 | 0.510416 | 0.989857 |
| 1718 | 0.936932 | 0.54188 | -0.39505 | 0.431239 | 0.515148 | 0.969257 |
| 1719 | 0.936932 | 0.54188 | -0.39505 | 0.431239 | 0.515148 | 0.969257 |
| 1720 | 0.996136 | 0.661383 | -0.33475 | 0.436404 | 0.510416 | 0.989857 |
| 1721 | 0.987485 | 0.561586 | -0.4259 | 0.429635 | 0.517011 | 0.962936 |
| 1722 | 0.936932 | 0.54188 | -0.39505 | 0.431239 | 0.515148 | 0.969257 |
| 1723 | 0.996136 | 0.661383 | -0.33475 | 0.436404 | 0.510416 | 0.989857 |
| 1724 | 0.974139 | 0.617137 | -0.357 | 0.434592 | 0.512079 | 0.982586 |
| 1725 | 0.974139 | 0.617137 | -0.357 | 0.434592 | 0.512079 | 0.982586 |
| 1726 | 0.918724 | 0.542619 | -0.3761 | 0.432444 | 0.51386 | 0.974028 |
| 1727 | 0.987409 | 0.558677 | -0.42873 | 0.429422 | 0.517219 | 0.962101 |
| 1728 | 0.985802 | 0.499189 | -0.48661 | 0.424831 | 0.521718 | 0.944216 |
| 1729 | 0.972242 | 0.543285 | -0.42896 | 0.429169 | 0.517382 | 0.961107 |
| 1730 | 0.795202 | 0.498709 | -0.29649 | 0.436918 | 0.508685 | 0.991924 |
| 1731 | 0.937451 | 0.56278 | -0.37467 | 0.432817 | 0.513608 | 0.975512 |
| 1732 | 0.923215 | 0.505613 | -0.4176 | 0.429214 | 0.517042 | 0.961284 |
| 1733 | 0.973488 | 0.591491 | -0.382 | 0.432745 | 0.513885 | 0.975223 |
| 1734 | 0.806725 | 0.559789 | -0.24694 | 0.441016 | 0.504808 | 1.00857 |
| 1735 | 0.937451 | 0.56278 | -0.37467 | 0.432817 | 0.513608 | 0.975512 |
| 1736 | 0.971993 | 0.533855 | -0.43814 | 0.428445 | 0.51809 | 0.95827 |
| 1737 | 0.869858 | 0.532409 | -0.33745 | 0.434745 | 0.511309 | 0.9832 |
| 1738 | 0.919878 | 0.590742 | -0.32914 | 0.436031 | 0.510368 | 0.988356 |
| 1739 | 0.932982 | 0.692761 | -0.24022 | 0.442525 | 0.504113 | 1.01476 |
| 1740 | 0.937451 | 0.56278 | -0.37467 | 0.432817 | 0.513608 | 0.975512 |
| 1741 | 0.806702 | 0.558688 | -0.24801 | 0.440928 | 0.504893 | 1.00821 |
| 1742 | 0.986965 | 0.542034 | -0.44493 | 0.428164 | 0.518452 | 0.95717 |
| 1743 | 0.986965 | 0.542034 | -0.44493 | 0.428164 | 0.518452 | 0.95717 |
| 1744 | 0.858723 | 0.589883 | -0.26884 | 0.439875 | 0.506259 | 1.00391 |
| 1745 | 0.97633 | 0.612906 | -0.36342 | 0.434145 | 0.512528 | 0.9808 |
| 1746 | 0.97633 | 0.612906 | -0.36342 | 0.434145 | 0.512528 | 0.9808 |
| 1747 | 0.985802 | 0.499189 | -0.48661 | 0.424831 | 0.521718 | 0.944216 |
| 1748 | 0.985802 | 0.499189 | -0.48661 | 0.424831 | 0.521718 | 0.944216 |
| 1749 | 0.970786 | 0.488825 | -0.48196 | 0.424896 | 0.521564 | 0.944466 |
| 1750 | 0.986965 | 0.542034 | -0.44493 | 0.428164 | 0.518452 | 0.95717 |
| 1751 | 0.987485 | 0.561586 | -0.4259 | 0.429635 | 0.517011 | 0.962936 |
| 1752 | 0.987485 | 0.561586 | -0.4259 | 0.429635 | 0.517011 | 0.962936 |
| 1753 | 0.987485 | 0.561586 | -0.4259 | 0.429635 | 0.517011 | 0.962936 |
| 1754 | 0.987485 | 0.561586 | -0.4259 | 0.429635 | 0.517011 | 0.962936 |
| 1755 | 0.987485 | 0.561586 | -0.4259 | 0.429635 | 0.517011 | 0.962936 |
| 1756 | 0.987485 | 0.561586 | -0.4259 | 0.429635 | 0.517011 | 0.962936 |
| 1757 | 0.987485 | 0.561586 | -0.4259 | 0.429635 | 0.517011 | 0.962936 |
| 1758 | 0.987485 | 0.561586 | -0.4259 | 0.429635 | 0.517011 | 0.962936 |
| AsparagineInsertion | 0.997638 | 0.725749 | -0.27189 | 0.440756 | 0.506157 | 1.00751 |
| 1759 | 0.971993 | 0.533855 | -0.43814 | 0.428445 | 0.51809 | 0.95827 |
| 1760 | 0.937451 | 0.56278 | -0.37467 | 0.432817 | 0.513608 | 0.975512 |
| 1761 | 0.923215 | 0.505613 | -0.4176 | 0.429214 | 0.517042 | 0.961284 |
| 1762 | 0.787123 | 0.486854 | -0.30027 | 0.436473 | 0.509055 | 0.990134 |
| 1763 | 0.991861 | 0.499837 | -0.49202 | 0.424517 | 0.522062 | 0.943004 |
| 1764 | 0.787123 | 0.486854 | -0.30027 | 0.436473 | 0.509055 | 0.990134 |
| 1765 | 0.987224 | 0.551719 | -0.43551 | 0.4289 | 0.517731 | 0.960052 |
| 1766 | 0.937451 | 0.56278 | -0.37467 | 0.432817 | 0.513608 | 0.975512 |
| 1767 | 0.987485 | 0.561586 | -0.4259 | 0.429635 | 0.517011 | 0.962936 |
| 1768 | 0.974139 | 0.617137 | -0.357 | 0.434592 | 0.512079 | 0.982586 |
| 1769 | 0.918956 | 0.552208 | -0.36675 | 0.433168 | 0.513155 | 0.976908 |
| 1770 | 0.990556 | 0.681067 | -0.30949 | 0.438125 | 0.508702 | 0.996803 |
| 1771 | 0.972492 | 0.55289 | -0.4196 | 0.429891 | 0.516675 | 0.963944 |
| 1772 | 0.974139 | 0.617137 | -0.357 | 0.434592 | 0.512079 | 0.982586 |
| 1773 | 0.98943 | 0.63651 | -0.35292 | 0.435046 | 0.511714 | 0.984402 |
| 1774 | 0.987485 | 0.561586 | -0.4259 | 0.429635 | 0.517011 | 0.962936 |
| 1775 | 0.987485 | 0.561586 | -0.4259 | 0.429635 | 0.517011 | 0.962936 |
| 1776 | 0.937451 | 0.56278 | -0.37467 | 0.432817 | 0.513608 | 0.975512 |
| 1777 | 0.937451 | 0.56278 | -0.37467 | 0.432817 | 0.513608 | 0.975512 |
| 1778 | 0.974139 | 0.617137 | -0.357 | 0.434592 | 0.512079 | 0.982586 |
| 1779 | 0.986965 | 0.542034 | -0.44493 | 0.428164 | 0.518452 | 0.95717 |
| 1780 | 0.974294 | 0.582579 | -0.39172 | 0.432028 | 0.514591 | 0.972382 |
| 1781 | 0.987409 | 0.558677 | -0.42873 | 0.429422 | 0.517219 | 0.962101 |
| 1782 | 0.936932 | 0.54188 | -0.39505 | 0.431239 | 0.515148 | 0.969257 |
| 1783 | 0.936932 | 0.54188 | -0.39505 | 0.431239 | 0.515148 | 0.969257 |
| 1784 | 0.936932 | 0.54188 | -0.39505 | 0.431239 | 0.515148 | 0.969257 |
| 1785 | 0.789141 | 0.5889 | -0.20024 | 0.444412 | 0.50141 | 1.02255 |
| 1786 | 0.869858 | 0.532409 | -0.33745 | 0.434745 | 0.511309 | 0.9832 |
| 1787 | 0.987224 | 0.551719 | -0.43551 | 0.4289 | 0.517731 | 0.960052 |
| 1788 | 0.98943 | 0.63651 | -0.35292 | 0.435046 | 0.511714 | 0.984402 |
| 1789 | 0.932982 | 0.692761 | -0.24022 | 0.442525 | 0.504113 | 1.01476 |
| 1790 | 0.986965 | 0.542034 | -0.44493 | 0.428164 | 0.518452 | 0.95717 |
| 1791 | 0.937451 | 0.56278 | -0.37467 | 0.432817 | 0.513608 | 0.975512 |
| 1792 | 0.789141 | 0.5889 | -0.20024 | 0.444412 | 0.50141 | 1.02255 |
| 1793 | 0.936932 | 0.54188 | -0.39505 | 0.431239 | 0.515148 | 0.969257 |
| 1794 | 0.98943 | 0.63651 | -0.35292 | 0.435046 | 0.511714 | 0.984402 |
| 1795 | 0.986965 | 0.542034 | -0.44493 | 0.428164 | 0.518452 | 0.95717 |
| 1796 | 0.936932 | 0.54188 | -0.39505 | 0.431239 | 0.515148 | 0.969257 |
| 1797 | 0.936932 | 0.54188 | -0.39505 | 0.431239 | 0.515148 | 0.969257 |
| 1798 | 0.974139 | 0.617137 | -0.357 | 0.434592 | 0.512079 | 0.982586 |
| 1799 | 0.918956 | 0.552208 | -0.36675 | 0.433168 | 0.513155 | 0.976908 |
| 1800 | 0.987409 | 0.558677 | -0.42873 | 0.429422 | 0.517219 | 0.962101 |
| 1801 | 0.936932 | 0.54188 | -0.39505 | 0.431239 | 0.515148 | 0.969257 |
| 1802 | 0.987485 | 0.561586 | -0.4259 | 0.429635 | 0.517011 | 0.962936 |
| 1803 | 0.937451 | 0.56278 | -0.37467 | 0.432817 | 0.513608 | 0.975512 |
| 1804 | 0.958316 | 0.515962 | -0.44235 | 0.427866 | 0.518577 | 0.956005 |
| 1805 | 0.972242 | 0.543285 | -0.42896 | 0.429169 | 0.517382 | 0.961107 |
| 1806 | 0.987409 | 0.558677 | -0.42873 | 0.429422 | 0.517219 | 0.962101 |
| 1807 | 0.970786 | 0.488825 | -0.48196 | 0.424896 | 0.521564 | 0.944466 |
| 1808 | 0.986965 | 0.542034 | -0.44493 | 0.428164 | 0.518452 | 0.95717 |
| 1809 | 0.996136 | 0.661383 | -0.33475 | 0.436404 | 0.510416 | 0.989857 |
| 1810 | 0.922331 | 0.696783 | -0.22555 | 0.443477 | 0.50313 | 1.01868 |
| 1811 | 0.986965 | 0.542034 | -0.44493 | 0.428164 | 0.518452 | 0.95717 |
| 1812 | 0.996136 | 0.661383 | -0.33475 | 0.436404 | 0.510416 | 0.989857 |
| 1813 | 0.974139 | 0.617137 | -0.357 | 0.434592 | 0.512079 | 0.982586 |
| 1814 | 0.806284 | 0.538028 | -0.26826 | 0.439345 | 0.506419 | 1.00175 |
| 1815 | 0.987409 | 0.558677 | -0.42873 | 0.429422 | 0.517219 | 0.962101 |
| 1816 | 0.986965 | 0.542034 | -0.44493 | 0.428164 | 0.518452 | 0.95717 |
| 1817 | 0.795202 | 0.498709 | -0.29649 | 0.436918 | 0.508685 | 0.991924 |
| 1818 | 0.918956 | 0.552208 | -0.36675 | 0.433168 | 0.513155 | 0.976908 |
| 1819 | 0.987409 | 0.558677 | -0.42873 | 0.429422 | 0.517219 | 0.962101 |
| 1820 | 0.987485 | 0.561586 | -0.4259 | 0.429635 | 0.517011 | 0.962936 |
| 1821 | 0.973488 | 0.591491 | -0.382 | 0.432745 | 0.513885 | 0.975223 |
| 1822 | 0.970786 | 0.488825 | -0.48196 | 0.424896 | 0.521564 | 0.944466 |
| 1823 | 0.973488 | 0.591491 | -0.382 | 0.432745 | 0.513885 | 0.975223 |
| 1824 | 0.869858 | 0.532409 | -0.33745 | 0.434745 | 0.511309 | 0.9832 |
| 1825 | 0.974294 | 0.582579 | -0.39172 | 0.432028 | 0.514591 | 0.972382 |
| 1826 | 0.869858 | 0.532409 | -0.33745 | 0.434745 | 0.511309 | 0.9832 |
| 1827 | 0.987224 | 0.551719 | -0.43551 | 0.4289 | 0.517731 | 0.960052 |
| 1828 | 0.990556 | 0.681067 | -0.30949 | 0.438125 | 0.508702 | 0.996803 |
| 1829 | 0.974294 | 0.582579 | -0.39172 | 0.432028 | 0.514591 | 0.972382 |
| 1830 | 0.974139 | 0.617137 | -0.357 | 0.434592 | 0.512079 | 0.982586 |
| 1831 | 0.958093 | 0.507466 | -0.45063 | 0.4272 | 0.519228 | 0.95341 |
| 1832 | 0.991861 | 0.499837 | -0.49202 | 0.424517 | 0.522062 | 0.943004 |
| 1833 | 0.990556 | 0.681067 | -0.30949 | 0.438125 | 0.508702 | 0.996803 |
| 1834 | 0.986965 | 0.542034 | -0.44493 | 0.428164 | 0.518452 | 0.95717 |
| 1835 | 0.991861 | 0.499837 | -0.49202 | 0.424517 | 0.522062 | 0.943004 |
| 1836 | 0.973488 | 0.591491 | -0.382 | 0.432745 | 0.513885 | 0.975223 |
| 1837 | 0.917433 | 0.490551 | -0.42688 | 0.428342 | 0.517857 | 0.957865 |
| 1838 | 0.986965 | 0.542034 | -0.44493 | 0.428164 | 0.518452 | 0.95717 |
| 1839 | 0.936932 | 0.54188 | -0.39505 | 0.431239 | 0.515148 | 0.969257 |
| 1840 | 0.986965 | 0.542034 | -0.44493 | 0.428164 | 0.518452 | 0.95717 |
| 1841 | 0.936932 | 0.54188 | -0.39505 | 0.431239 | 0.515148 | 0.969257 |
| 1842 | 0.937451 | 0.56278 | -0.37467 | 0.432817 | 0.513608 | 0.975512 |
| 1843 | 0.936932 | 0.54188 | -0.39505 | 0.431239 | 0.515148 | 0.969257 |
| 1844 | 0.987409 | 0.558677 | -0.42873 | 0.429422 | 0.517219 | 0.962101 |
| 1845 | 0.932982 | 0.692761 | -0.24022 | 0.442525 | 0.504113 | 1.01476 |
| 1846 | 0.936932 | 0.54188 | -0.39505 | 0.431239 | 0.515148 | 0.969257 |
| 1847 | 0.961357 | 0.636079 | -0.32528 | 0.436747 | 0.509902 | 0.991236 |
| 1848 | 0.98943 | 0.63651 | -0.35292 | 0.435046 | 0.511714 | 0.984402 |
| 1849 | 0.987485 | 0.561586 | -0.4259 | 0.429635 | 0.517011 | 0.962936 |
| 1850 | 0.937451 | 0.56278 | -0.37467 | 0.432817 | 0.513608 | 0.975512 |
| 1851 | 0.973488 | 0.591491 | -0.382 | 0.432745 | 0.513885 | 0.975223 |
| 1852 | 0.936932 | 0.54188 | -0.39505 | 0.431239 | 0.515148 | 0.969257 |
| 1853 | 0.918956 | 0.552208 | -0.36675 | 0.433168 | 0.513155 | 0.976908 |
| 1854 | 0.937479 | 0.563894 | -0.37359 | 0.432905 | 0.513523 | 0.975859 |
| 1855 | 0.986965 | 0.542034 | -0.44493 | 0.428164 | 0.518452 | 0.95717 |
| 1856 | 0.987409 | 0.558677 | -0.42873 | 0.429422 | 0.517219 | 0.962101 |
| 1857 | 0.803087 | 0.684908 | -0.11818 | 0.450428 | 0.495711 | 1.04774 |
| 1858 | 0.987224 | 0.551719 | -0.43551 | 0.4289 | 0.517731 | 0.960052 |
| 1859 | 0.932982 | 0.692761 | -0.24022 | 0.442525 | 0.504113 | 1.01476 |
| 1860 | 0.918724 | 0.542619 | -0.3761 | 0.432444 | 0.51386 | 0.974028 |
| 1861 | 0.918724 | 0.542619 | -0.3761 | 0.432444 | 0.51386 | 0.974028 |
| 1862 | 0.987409 | 0.558677 | -0.42873 | 0.429422 | 0.517219 | 0.962101 |
| 1863 | 0.957872 | 0.499115 | -0.45876 | 0.426532 | 0.519881 | 0.950809 |
| 1864 | 0.917433 | 0.490551 | -0.42688 | 0.428342 | 0.517857 | 0.957865 |
| 1865 | 0.970786 | 0.488825 | -0.48196 | 0.424896 | 0.521564 | 0.944466 |
| 1866 | 0.936932 | 0.54188 | -0.39505 | 0.431239 | 0.515148 | 0.969257 |
| 1867 | 0.987409 | 0.558677 | -0.42873 | 0.429422 | 0.517219 | 0.962101 |
| 1868 | 1.19872 | 9.61677 | 8.41804 | 0.930986 | 0.028106 | 17.2446 |
| 1869 | 0.986965 | 0.542034 | -0.44493 | 0.428164 | 0.518452 | 0.95717 |
| 1870 | 0.996136 | 0.661383 | -0.33475 | 0.436404 | 0.510416 | 0.989857 |
| 1871 | 0.936932 | 0.54188 | -0.39505 | 0.431239 | 0.515148 | 0.969257 |
| 1872 | 0.972492 | 0.55289 | -0.4196 | 0.429891 | 0.516675 | 0.963944 |
| 1873 | 0.996136 | 0.661383 | -0.33475 | 0.436404 | 0.510416 | 0.989857 |
| 1874 | 0.919878 | 0.590742 | -0.32914 | 0.436031 | 0.510368 | 0.988356 |
| 1875 | 0.936932 | 0.54188 | -0.39505 | 0.431239 | 0.515148 | 0.969257 |
| 1876 | 0.987409 | 0.558677 | -0.42873 | 0.429422 | 0.517219 | 0.962101 |
| 1877 | 0.937451 | 0.56278 | -0.37467 | 0.432817 | 0.513608 | 0.975512 |
| 1878 | 0.936932 | 0.54188 | -0.39505 | 0.431239 | 0.515148 | 0.969257 |
| 1879 | 0.936932 | 0.54188 | -0.39505 | 0.431239 | 0.515148 | 0.969257 |
| 1880 | 0.986965 | 0.542034 | -0.44493 | 0.428164 | 0.518452 | 0.95717 |
| 1881 | 0.991861 | 0.499837 | -0.49202 | 0.424517 | 0.522062 | 0.943004 |
| 1882 | 0.889846 | 0.592529 | -0.29732 | 0.438067 | 0.508207 | 0.996567 |
| 1883 | 0.917433 | 0.490551 | -0.42688 | 0.428342 | 0.517857 | 0.957865 |
| 1884 | 0.958316 | 0.515962 | -0.44235 | 0.427866 | 0.518577 | 0.956005 |
| 1885 | 0.987485 | 0.561586 | -0.4259 | 0.429635 | 0.517011 | 0.962936 |
| 1886 | 0.987409 | 0.558677 | -0.42873 | 0.429422 | 0.517219 | 0.962101 |
| 1887 | 0.987224 | 0.551719 | -0.43551 | 0.4289 | 0.517731 | 0.960052 |
| 1888 | 0.937451 | 0.56278 | -0.37467 | 0.432817 | 0.513608 | 0.975512 |
| 1889 | 0.869858 | 0.532409 | -0.33745 | 0.434745 | 0.511309 | 0.9832 |
| 1890 | 0.987485 | 0.561586 | -0.4259 | 0.429635 | 0.517011 | 0.962936 |
| 1891 | 0.937451 | 0.56278 | -0.37467 | 0.432817 | 0.513608 | 0.975512 |
| 1892 | 0.937451 | 0.56278 | -0.37467 | 0.432817 | 0.513608 | 0.975512 |
| 1893 | 0.974294 | 0.582579 | -0.39172 | 0.432028 | 0.514591 | 0.972382 |
| 1894 | 0.932982 | 0.692761 | -0.24022 | 0.442525 | 0.504113 | 1.01476 |
| 1895 | 0.972492 | 0.55289 | -0.4196 | 0.429891 | 0.516675 | 0.963944 |
| 1896 | 0.869858 | 0.532409 | -0.33745 | 0.434745 | 0.511309 | 0.9832 |
| 1897 | 0.806702 | 0.558688 | -0.24801 | 0.440928 | 0.504893 | 1.00821 |
| 1898 | 0.932982 | 0.692761 | -0.24022 | 0.442525 | 0.504113 | 1.01476 |
| 1899 | 0.936932 | 0.54188 | -0.39505 | 0.431239 | 0.515148 | 0.969257 |
| 1900 | 0.932982 | 0.692761 | -0.24022 | 0.442525 | 0.504113 | 1.01476 |
| 1901 | 0.803087 | 0.684908 | -0.11818 | 0.450428 | 0.495711 | 1.04774 |
| 1902 | 0.937451 | 0.56278 | -0.37467 | 0.432817 | 0.513608 | 0.975512 |
| 1903 | 0.987485 | 0.561586 | -0.4259 | 0.429635 | 0.517011 | 0.962936 |
| 1904 | 0.937451 | 0.56278 | -0.37467 | 0.432817 | 0.513608 | 0.975512 |
| 1905 | 0.986965 | 0.542034 | -0.44493 | 0.428164 | 0.518452 | 0.95717 |
| 1906 | 0.974294 | 0.582579 | -0.39172 | 0.432028 | 0.514591 | 0.972382 |
| 1907 | 0.918956 | 0.552208 | -0.36675 | 0.433168 | 0.513155 | 0.976908 |
| 1908 | 0.923215 | 0.505613 | -0.4176 | 0.429214 | 0.517042 | 0.961284 |
| 1909 | 0.918956 | 0.552208 | -0.36675 | 0.433168 | 0.513155 | 0.976908 |
| 1910 | 0.937451 | 0.56278 | -0.37467 | 0.432817 | 0.513608 | 0.975512 |
| 1911 | 0.987485 | 0.561586 | -0.4259 | 0.429635 | 0.517011 | 0.962936 |
| 1912 | 0.918956 | 0.552208 | -0.36675 | 0.433168 | 0.513155 | 0.976908 |
| 1913 | 0.790468 | 0.659182 | -0.13129 | 0.449464 | 0.496557 | 1.04366 |
| 1914 | 0.986965 | 0.542034 | -0.44493 | 0.428164 | 0.518452 | 0.95717 |
| 1915 | 0.973488 | 0.591491 | -0.382 | 0.432745 | 0.513885 | 0.975223 |
| 1916 | 0.987409 | 0.558677 | -0.42873 | 0.429422 | 0.517219 | 0.962101 |
| 1917 | 0.987485 | 0.561586 | -0.4259 | 0.429635 | 0.517011 | 0.962936 |
| 1918 | 0.957872 | 0.499115 | -0.45876 | 0.426532 | 0.519881 | 0.950809 |
| 1919 | 0.98943 | 0.63651 | -0.35292 | 0.435046 | 0.511714 | 0.984402 |
| 1920 | 0.918956 | 0.552208 | -0.36675 | 0.433168 | 0.513155 | 0.976908 |
| 1921 | 0.932982 | 0.692761 | -0.24022 | 0.442525 | 0.504113 | 1.01476 |
| 1922 | 0.990556 | 0.681067 | -0.30949 | 0.438125 | 0.508702 | 0.996803 |
| 1923 | 0.923215 | 0.505613 | -0.4176 | 0.429214 | 0.517042 | 0.961284 |
| 1924 | 0.936932 | 0.54188 | -0.39505 | 0.431239 | 0.515148 | 0.969257 |
| 1925 | 0.936932 | 0.54188 | -0.39505 | 0.431239 | 0.515148 | 0.969257 |
| 1926 | 0.986965 | 0.542034 | -0.44493 | 0.428164 | 0.518452 | 0.95717 |
| 1927 | 0.987409 | 0.558677 | -0.42873 | 0.429422 | 0.517219 | 0.962101 |
| 1928 | 0.972242 | 0.543285 | -0.42896 | 0.429169 | 0.517382 | 0.961107 |
| 1929 | 0.937479 | 0.563894 | -0.37359 | 0.432905 | 0.513523 | 0.975859 |
| 1930 | 0.922331 | 0.696783 | -0.22555 | 0.443477 | 0.50313 | 1.01868 |
| 1931 | 0.987485 | 0.561586 | -0.4259 | 0.429635 | 0.517011 | 0.962936 |
| 1932 | 0.986965 | 0.542034 | -0.44493 | 0.428164 | 0.518452 | 0.95717 |
| 1933 | 0.918956 | 0.552208 | -0.36675 | 0.433168 | 0.513155 | 0.976908 |
| 1934 | 0.987409 | 0.558677 | -0.42873 | 0.429422 | 0.517219 | 0.962101 |
| 1935 | 0.974294 | 0.582579 | -0.39172 | 0.432028 | 0.514591 | 0.972382 |
| 1936 | 0.987485 | 0.561586 | -0.4259 | 0.429635 | 0.517011 | 0.962936 |
| 1937 | 0.918493 | 0.533206 | -0.38529 | 0.431717 | 0.514568 | 0.971148 |
| 1938 | 0.932982 | 0.692761 | -0.24022 | 0.442525 | 0.504113 | 1.01476 |
| 1939 | 0.987409 | 0.558677 | -0.42873 | 0.429422 | 0.517219 | 0.962101 |
| 1940 | 0.987485 | 0.561586 | -0.4259 | 0.429635 | 0.517011 | 0.962936 |
| 1941 | 0.936932 | 0.54188 | -0.39505 | 0.431239 | 0.515148 | 0.969257 |
| 1942 | 0.996136 | 0.661383 | -0.33475 | 0.436404 | 0.510416 | 0.989857 |
| 1943 | 0.936932 | 0.54188 | -0.39505 | 0.431239 | 0.515148 | 0.969257 |
| 1944 | 0.936932 | 0.54188 | -0.39505 | 0.431239 | 0.515148 | 0.969257 |
| 1945 | 0.996136 | 0.661383 | -0.33475 | 0.436404 | 0.510416 | 0.989857 |
| 1946 | 0.917433 | 0.490551 | -0.42688 | 0.428342 | 0.517857 | 0.957865 |
| 1947 | 0.986965 | 0.542034 | -0.44493 | 0.428164 | 0.518452 | 0.95717 |
| 1948 | 0.987409 | 0.558677 | -0.42873 | 0.429422 | 0.517219 | 0.962101 |
| 1949 | 0.859272 | 0.615434 | -0.24384 | 0.441734 | 0.50446 | 1.01151 |
| 1950 | 0.985802 | 0.499189 | -0.48661 | 0.424831 | 0.521718 | 0.944216 |
| 1951 | 0.972492 | 0.55289 | -0.4196 | 0.429891 | 0.516675 | 0.963944 |
| 1952 | 0.987409 | 0.558677 | -0.42873 | 0.429422 | 0.517219 | 0.962101 |
| 1953 | 0.787123 | 0.486854 | -0.30027 | 0.436473 | 0.509055 | 0.990134 |
| 1954 | 0.936932 | 0.54188 | -0.39505 | 0.431239 | 0.515148 | 0.969257 |
| 1955 | 0.917433 | 0.490551 | -0.42688 | 0.428342 | 0.517857 | 0.957865 |
| 1956 | 0.922331 | 0.696783 | -0.22555 | 0.443477 | 0.50313 | 1.01868 |
| 1957 | 0.990556 | 0.681067 | -0.30949 | 0.438125 | 0.508702 | 0.996803 |
| 1958 | 0.937451 | 0.56278 | -0.37467 | 0.432817 | 0.513608 | 0.975512 |
| 1959 | 0.974294 | 0.582579 | -0.39172 | 0.432028 | 0.514591 | 0.972382 |
| 1960 | 0.795202 | 0.498709 | -0.29649 | 0.436918 | 0.508685 | 0.991924 |
| 1961 | 0.937451 | 0.56278 | -0.37467 | 0.432817 | 0.513608 | 0.975512 |
| 1962 | 0.923215 | 0.505613 | -0.4176 | 0.429214 | 0.517042 | 0.961284 |
| 1963 | 0.937451 | 0.56278 | -0.37467 | 0.432817 | 0.513608 | 0.975512 |
| 1964 | 0.806284 | 0.538028 | -0.26826 | 0.439345 | 0.506419 | 1.00175 |
| 1965 | 0.972242 | 0.543285 | -0.42896 | 0.429169 | 0.517382 | 0.961107 |
| 1966 | 0.803087 | 0.684908 | -0.11818 | 0.450428 | 0.495711 | 1.04774 |
| 1967 | 0.936932 | 0.54188 | -0.39505 | 0.431239 | 0.515148 | 0.969257 |
| 1968 | 0.803087 | 0.684908 | -0.11818 | 0.450428 | 0.495711 | 1.04774 |
| 1969 | 0.974294 | 0.582579 | -0.39172 | 0.432028 | 0.514591 | 0.972382 |
| 1970 | 0.972242 | 0.543285 | -0.42896 | 0.429169 | 0.517382 | 0.961107 |
| 1971 | 0.918493 | 0.533206 | -0.38529 | 0.431717 | 0.514568 | 0.971148 |
| 1972 | 0.974139 | 0.617137 | -0.357 | 0.434592 | 0.512079 | 0.982586 |
| 1973 | 0.937479 | 0.563894 | -0.37359 | 0.432905 | 0.513523 | 0.975859 |
| 1974 | 0.986965 | 0.542034 | -0.44493 | 0.428164 | 0.518452 | 0.95717 |
| 1975 | 0.974294 | 0.582579 | -0.39172 | 0.432028 | 0.514591 | 0.972382 |
| 1976 | 0.987485 | 0.561586 | -0.4259 | 0.429635 | 0.517011 | 0.962936 |
| 1977 | 0.917433 | 0.490551 | -0.42688 | 0.428342 | 0.517857 | 0.957865 |
| 1978 | 0.987485 | 0.561586 | -0.4259 | 0.429635 | 0.517011 | 0.962936 |
| 1979 | 0.97633 | 0.612906 | -0.36342 | 0.434145 | 0.512528 | 0.9808 |
| 1980 | 0.970786 | 0.488825 | -0.48196 | 0.424896 | 0.521564 | 0.944466 |
| 1981 | 0.803087 | 0.684908 | -0.11818 | 0.450428 | 0.495711 | 1.04774 |
| 1982 | 0.918956 | 0.552208 | -0.36675 | 0.433168 | 0.513155 | 0.976908 |
| 1983 | 0.987409 | 0.558677 | -0.42873 | 0.429422 | 0.517219 | 0.962101 |
| 1984 | 0.918493 | 0.533206 | -0.38529 | 0.431717 | 0.514568 | 0.971148 |
| 1985 | 0.937479 | 0.563894 | -0.37359 | 0.432905 | 0.513523 | 0.975859 |
| 1986 | 0.937479 | 0.563894 | -0.37359 | 0.432905 | 0.513523 | 0.975859 |
| 1987 | 0.987485 | 0.561586 | -0.4259 | 0.429635 | 0.517011 | 0.962936 |
| 1988 | 0.974294 | 0.582579 | -0.39172 | 0.432028 | 0.514591 | 0.972382 |
| 1989 | 0.937451 | 0.56278 | -0.37467 | 0.432817 | 0.513608 | 0.975512 |
| 1990 | 0.973488 | 0.591491 | -0.382 | 0.432745 | 0.513885 | 0.975223 |
| 1991 | 0.936932 | 0.54188 | -0.39505 | 0.431239 | 0.515148 | 0.969257 |
| 1992 | 0.936932 | 0.54188 | -0.39505 | 0.431239 | 0.515148 | 0.969257 |
| 1993 | 0.923215 | 0.505613 | -0.4176 | 0.429214 | 0.517042 | 0.961284 |
| 1994 | 0.936932 | 0.54188 | -0.39505 | 0.431239 | 0.515148 | 0.969257 |
| 1995 | 0.857464 | 0.532463 | -0.325 | 0.435545 | 0.510451 | 0.986402 |
| 1996 | 0.987409 | 0.558677 | -0.42873 | 0.429422 | 0.517219 | 0.962101 |
| 1997 | 0.974294 | 0.582579 | -0.39172 | 0.432028 | 0.514591 | 0.972382 |
| 1998 | 0.973488 | 0.591491 | -0.382 | 0.432745 | 0.513885 | 0.975223 |
| 1999 | 0.918724 | 0.542619 | -0.3761 | 0.432444 | 0.51386 | 0.974028 |
| 2000 | 0.918493 | 0.533206 | -0.38529 | 0.431717 | 0.514568 | 0.971148 |
| 2001 | 0.987409 | 0.558677 | -0.42873 | 0.429422 | 0.517219 | 0.962101 |
| 2002 | 0.936932 | 0.54188 | -0.39505 | 0.431239 | 0.515148 | 0.969257 |
| 2003 | 0.986965 | 0.542034 | -0.44493 | 0.428164 | 0.518452 | 0.95717 |
| 2004 | 0.936932 | 0.54188 | -0.39505 | 0.431239 | 0.515148 | 0.969257 |
| 2005 | 0.974139 | 0.617137 | -0.357 | 0.434592 | 0.512079 | 0.982586 |
| 2006 | 0.857884 | 0.551426 | -0.30646 | 0.437001 | 0.50904 | 0.992262 |
| 2007 | 0.958316 | 0.515962 | -0.44235 | 0.427866 | 0.518577 | 0.956005 |
| 2008 | 0.917433 | 0.490551 | -0.42688 | 0.428342 | 0.517857 | 0.957865 |
| 2009 | 0.917433 | 0.490551 | -0.42688 | 0.428342 | 0.517857 | 0.957865 |
| 2010 | 0.918956 | 0.552208 | -0.36675 | 0.433168 | 0.513155 | 0.976908 |
| 2011 | 0.936932 | 0.54188 | -0.39505 | 0.431239 | 0.515148 | 0.969257 |
| 2012 | 0.919878 | 0.590742 | -0.32914 | 0.436031 | 0.510368 | 0.988356 |
| 2013 | 0.936932 | 0.54188 | -0.39505 | 0.431239 | 0.515148 | 0.969257 |
| 2014 | 0.917433 | 0.490551 | -0.42688 | 0.428342 | 0.517857 | 0.957865 |
| 2015 | 0.974139 | 0.617137 | -0.357 | 0.434592 | 0.512079 | 0.982586 |
| 2016 | 0.972492 | 0.55289 | -0.4196 | 0.429891 | 0.516675 | 0.963944 |
| 2017 | 0.972242 | 0.543285 | -0.42896 | 0.429169 | 0.517382 | 0.961107 |
| 2018 | 0.974139 | 0.617137 | -0.357 | 0.434592 | 0.512079 | 0.982586 |
| 2019 | 0.974139 | 0.617137 | -0.357 | 0.434592 | 0.512079 | 0.982586 |
| 2020 | 0.918724 | 0.542619 | -0.3761 | 0.432444 | 0.51386 | 0.974028 |
| 2021 | 0.974139 | 0.617137 | -0.357 | 0.434592 | 0.512079 | 0.982586 |
| 2022 | 0.957872 | 0.499115 | -0.45876 | 0.426532 | 0.519881 | 0.950809 |
| 2023 | 0.985802 | 0.499189 | -0.48661 | 0.424831 | 0.521718 | 0.944216 |
| 2024 | 0.987485 | 0.561586 | -0.4259 | 0.429635 | 0.517011 | 0.962936 |
| 2025 | 0.987485 | 0.561586 | -0.4259 | 0.429635 | 0.517011 | 0.962936 |
| 2026 | 0.987485 | 0.561586 | -0.4259 | 0.429635 | 0.517011 | 0.962936 |
| 2027 | 0.986965 | 0.542034 | -0.44493 | 0.428164 | 0.518452 | 0.95717 |
| 2028 | 0.917433 | 0.490551 | -0.42688 | 0.428342 | 0.517857 | 0.957865 |
| 2029 | 0.922331 | 0.696783 | -0.22555 | 0.443477 | 0.50313 | 1.01868 |
| 2030 | 0.790468 | 0.659182 | -0.13129 | 0.449464 | 0.496557 | 1.04366 |
| 2031 | 0.970786 | 0.488825 | -0.48196 | 0.424896 | 0.521564 | 0.944466 |
| 2032 | 0.986965 | 0.542034 | -0.44493 | 0.428164 | 0.518452 | 0.95717 |
| 2033 | 0.987485 | 0.561586 | -0.4259 | 0.429635 | 0.517011 | 0.962936 |
| 2034 | 0.918956 | 0.552208 | -0.36675 | 0.433168 | 0.513155 | 0.976908 |
| 2035 | 0.917433 | 0.490551 | -0.42688 | 0.428342 | 0.517857 | 0.957865 |
| 2036 | 0.986965 | 0.542034 | -0.44493 | 0.428164 | 0.518452 | 0.95717 |
| 2037 | 0.922331 | 0.696783 | -0.22555 | 0.443477 | 0.50313 | 1.01868 |
| 2038 | 0.987485 | 0.561586 | -0.4259 | 0.429635 | 0.517011 | 0.962936 |
| 2039 | 0.986965 | 0.542034 | -0.44493 | 0.428164 | 0.518452 | 0.95717 |
| 2040 | 1.08186 | 5.92827 | 4.84641 | 0.8335 | 0.122599 | 6.39944 |
| 2041 | 0.951443 | 0.596942 | -0.3545 | 0.434512 | 0.512033 | 0.982266 |
| 2042 | 0.787123 | 0.486854 | -0.30027 | 0.436473 | 0.509055 | 0.990134 |
| 2043 | 0.991861 | 0.499837 | -0.49202 | 0.424517 | 0.522062 | 0.943004 |
| 2044 | 0.917433 | 0.490551 | -0.42688 | 0.428342 | 0.517857 | 0.957865 |
| 2045 | 0.918956 | 0.552208 | -0.36675 | 0.433168 | 0.513155 | 0.976908 |
| 2046 | 0.970786 | 0.488825 | -0.48196 | 0.424896 | 0.521564 | 0.944466 |
| 2047 | 1.15509 | 11.1485 | 9.99342 | 0.951694 | 0.011179 | 25.1853 |
| 2048 | 0.937451 | 0.56278 | -0.37467 | 0.432817 | 0.513608 | 0.975512 |
| 2049 | 0.973488 | 0.591491 | -0.382 | 0.432745 | 0.513885 | 0.975223 |
| 2050 | 0.790468 | 0.659182 | -0.13129 | 0.449464 | 0.496557 | 1.04366 |
| 2051 | 0.974294 | 0.582579 | -0.39172 | 0.432028 | 0.514591 | 0.972382 |
| 2052 | 0.971993 | 0.533855 | -0.43814 | 0.428445 | 0.51809 | 0.95827 |
| 2053 | 0.971993 | 0.533855 | -0.43814 | 0.428445 | 0.51809 | 0.95827 |
| 2054 | 0.917433 | 0.490551 | -0.42688 | 0.428342 | 0.517857 | 0.957865 |
| 2055 | 0.919878 | 0.590742 | -0.32914 | 0.436031 | 0.510368 | 0.988356 |
| 2056 | 0.974294 | 0.582579 | -0.39172 | 0.432028 | 0.514591 | 0.972382 |
| 2057 | 0.970786 | 0.488825 | -0.48196 | 0.424896 | 0.521564 | 0.944466 |
| 2058 | 0.932982 | 0.692761 | -0.24022 | 0.442525 | 0.504113 | 1.01476 |
| 2059 | 0.919878 | 0.590742 | -0.32914 | 0.436031 | 0.510368 | 0.988356 |
| 2060 | 0.857464 | 0.532463 | -0.325 | 0.435545 | 0.510451 | 0.986402 |
| 2061 | 0.932982 | 0.692761 | -0.24022 | 0.442525 | 0.504113 | 1.01476 |
| 2062 | 0.918493 | 0.533206 | -0.38529 | 0.431717 | 0.514568 | 0.971148 |
| 2063 | 0.918493 | 0.533206 | -0.38529 | 0.431717 | 0.514568 | 0.971148 |
| 2064 | 0.917433 | 0.490551 | -0.42688 | 0.428342 | 0.517857 | 0.957865 |
| 2065 | 0.970786 | 0.488825 | -0.48196 | 0.424896 | 0.521564 | 0.944466 |
| 2066 | 0.803087 | 0.684908 | -0.11818 | 0.450428 | 0.495711 | 1.04774 |
| 2067 | 0.974294 | 0.582579 | -0.39172 | 0.432028 | 0.514591 | 0.972382 |
| 2068 | 0.98943 | 0.63651 | -0.35292 | 0.435046 | 0.511714 | 0.984402 |
| 2069 | 0.917433 | 0.490551 | -0.42688 | 0.428342 | 0.517857 | 0.957865 |
| 2070 | 0.961357 | 0.636079 | -0.32528 | 0.436747 | 0.509902 | 0.991236 |
| 2071 | 0.987485 | 0.561586 | -0.4259 | 0.429635 | 0.517011 | 0.962936 |
| 2072 | 0.788402 | 0.550532 | -0.23787 | 0.441528 | 0.504185 | 1.01067 |
| 2073 | 0.932982 | 0.692761 | -0.24022 | 0.442525 | 0.504113 | 1.01476 |
| 2074 | 0.958316 | 0.515962 | -0.44235 | 0.427866 | 0.518577 | 0.956005 |
| 2075 | 0.970786 | 0.488829 | -0.48196 | 0.424896 | 0.521564 | 0.944467 |
| 2076 | 0.970786 | 0.488825 | -0.48196 | 0.424896 | 0.521564 | 0.944466 |
| 2077 | 0.806284 | 0.538028 | -0.26826 | 0.439345 | 0.506419 | 1.00175 |
| 2078 | 0.972242 | 0.543285 | -0.42896 | 0.429169 | 0.517382 | 0.961107 |
| 2079 | 0.972242 | 0.543285 | -0.42896 | 0.429169 | 0.517382 | 0.961107 |
| 2080 | 0.987224 | 0.551719 | -0.43551 | 0.4289 | 0.517731 | 0.960052 |
| 2081 | 0.985802 | 0.499189 | -0.48661 | 0.424831 | 0.521718 | 0.944216 |
| 2082 | 0.974139 | 0.617137 | -0.357 | 0.434592 | 0.512079 | 0.982586 |
| 2083 | 0.987485 | 0.561586 | -0.4259 | 0.429635 | 0.517011 | 0.962936 |
| 2084 | 0.991861 | 0.499837 | -0.49202 | 0.424517 | 0.522062 | 0.943004 |
| 2085 | 0.936932 | 0.54188 | -0.39505 | 0.431239 | 0.515148 | 0.969257 |
| 2086 | 0.990556 | 0.681067 | -0.30949 | 0.438125 | 0.508702 | 0.996803 |
| 2087 | 0.936932 | 0.54188 | -0.39505 | 0.431239 | 0.515148 | 0.969257 |
| 2088 | 0.987485 | 0.561586 | -0.4259 | 0.429635 | 0.517011 | 0.962936 |
| 2089 | 0.789141 | 0.5889 | -0.20024 | 0.444412 | 0.50141 | 1.02255 |
| 2090 | 0.806284 | 0.538028 | -0.26826 | 0.439345 | 0.506419 | 1.00175 |
| 2091 | 0.958316 | 0.515962 | -0.44235 | 0.427866 | 0.518577 | 0.956005 |
| 2092 | 0.958316 | 0.515962 | -0.44235 | 0.427866 | 0.518577 | 0.956005 |
| 2093 | 1.11707 | 10.2478 | 9.13073 | 0.943946 | 0.019453 | 21.5274 |
| 2094 | 0.970786 | 0.488825 | -0.48196 | 0.424896 | 0.521564 | 0.944466 |
| 2095 | 0.918956 | 0.552208 | -0.36675 | 0.433168 | 0.513155 | 0.976908 |
| 2096 | 0.936932 | 0.54188 | -0.39505 | 0.431239 | 0.515148 | 0.969257 |
| 2097 | 0.806284 | 0.538028 | -0.26826 | 0.439345 | 0.506419 | 1.00175 |
| 2098 | 1.1444 | 11.7952 | 10.6508 | 0.95816 | 0.005825 | 29.2747 |
| 2099 | 0.936932 | 0.54188 | -0.39505 | 0.431239 | 0.515148 | 0.969257 |
| 2100 | 0.987485 | 0.561586 | -0.4259 | 0.429635 | 0.517011 | 0.962936 |
| 2101 | 0.936932 | 0.54188 | -0.39505 | 0.431239 | 0.515148 | 0.969257 |
| 2102 | 0.958513 | 0.499971 | -0.45854 | 0.426562 | 0.519855 | 0.950927 |
| 2103 | 0.990556 | 0.681067 | -0.30949 | 0.438125 | 0.508702 | 0.996803 |
| 2104 | 0.806284 | 0.538028 | -0.26826 | 0.439345 | 0.506419 | 1.00175 |
| 2105 | 0.919878 | 0.590742 | -0.32914 | 0.436031 | 0.510368 | 0.988356 |
| 2106 | 0.789141 | 0.5889 | -0.20024 | 0.444412 | 0.50141 | 1.02255 |
| 2107 | 0.959147 | 0.50082 | -0.45833 | 0.426592 | 0.51983 | 0.951044 |
| 2108 | 0.791394 | 0.538978 | -0.25242 | 0.440421 | 0.505274 | 1.00614 |
| 2109 | 0.986965 | 0.542034 | -0.44493 | 0.428164 | 0.518452 | 0.95717 |
| 2110 | 0.957872 | 0.499115 | -0.45876 | 0.426532 | 0.519881 | 0.950809 |
| 2111 | 0.972242 | 0.543285 | -0.42896 | 0.429169 | 0.517382 | 0.961107 |
| 2112 | 0.991861 | 0.499837 | -0.49202 | 0.424517 | 0.522062 | 0.943004 |
| 2113 | 0.996136 | 0.661383 | -0.33475 | 0.436404 | 0.510416 | 0.989857 |
| 2114 | 0.958316 | 0.515962 | -0.44235 | 0.427866 | 0.518577 | 0.956005 |
| PF3D7_1244500 | | | | | | |
| Codon | **alpha** | **beta** | **beta-alpha** | **Posterior Prob Positive Selection** | **Posterior Prob Negative Selection** | **Empirical Bayes Factor** |
| 1 | 1.00702 | 0.508968 | -0.49805 | 0.431874 | 0.5167 | 0.902192 |
| 2 | 0.9371 | 0.545132 | -0.39197 | 0.439732 | 0.508574 | 0.93149 |
| 3 | 0.890364 | 0.625168 | -0.2652 | 0.449682 | 0.498575 | 0.96979 |
| 4 | 0.627901 | 0.527242 | -0.10066 | 0.460642 | 0.486148 | 1.01361 |
| 5 | 0.627798 | 0.514729 | -0.11307 | 0.459502 | 0.487236 | 1.00897 |
| 6 | 0.627605 | 0.491184 | -0.13642 | 0.457261 | 0.489376 | 0.999905 |
| 7 | 0.921714 | 0.564763 | -0.35695 | 0.442469 | 0.505802 | 0.941888 |
| 8 | 0.904336 | 0.609251 | -0.29509 | 0.447423 | 0.500858 | 0.960972 |
| 9 | 0.921714 | 0.564763 | -0.35695 | 0.442469 | 0.505802 | 0.941888 |
| 10 | 0.889689 | 0.41941 | -0.47028 | 0.430813 | 0.51701 | 0.898296 |
| 11 | 0.934182 | 0.351073 | -0.58311 | 0.419972 | 0.52796 | 0.859326 |
| 12 | 0.960912 | 0.604384 | -0.35653 | 0.44319 | 0.505311 | 0.944645 |
| 13 | 0.627605 | 0.491184 | -0.13642 | 0.457261 | 0.489376 | 0.999905 |
| 14 | 0.921714 | 0.564763 | -0.35695 | 0.442469 | 0.505802 | 0.941888 |
| 15 | 0.921714 | 0.564763 | -0.35695 | 0.442469 | 0.505802 | 0.941888 |
| 16 | 0.533342 | 0.548109 | 0.014767 | 0.470618 | 0.475786 | 1.05508 |
| 17 | 0.786362 | 0.596037 | -0.19033 | 0.454599 | 0.493162 | 0.989233 |
| 18 | 0.960912 | 0.604384 | -0.35653 | 0.44319 | 0.505311 | 0.944645 |
| 19 | 1.00797 | 0.565383 | -0.44259 | 0.436808 | 0.511837 | 0.920492 |
| 20 | 0.922879 | 0.642984 | -0.2799 | 0.448919 | 0.499495 | 0.966805 |
| 21 | 0.891537 | 0.550596 | -0.34094 | 0.443293 | 0.504818 | 0.94504 |
| 22 | 0.919919 | 0.443482 | -0.47644 | 0.431305 | 0.516731 | 0.900102 |
| 23 | 0.922879 | 0.642984 | -0.2799 | 0.448919 | 0.499495 | 0.966805 |
| 24 | 0.937188 | 0.550916 | -0.38627 | 0.44022 | 0.508096 | 0.933336 |
| 25 | 0.865573 | 0.644353 | -0.22122 | 0.45297 | 0.49523 | 0.982751 |
| 26 | 0.9371 | 0.545132 | -0.39197 | 0.439732 | 0.508574 | 0.93149 |
| 27 | 0.98361 | 0.571041 | -0.41257 | 0.438895 | 0.509653 | 0.92833 |
| 28 | 0.934182 | 0.351073 | -0.58311 | 0.419972 | 0.52796 | 0.859326 |
| 29 | 0.937218 | 0.552927 | -0.38429 | 0.440382 | 0.507937 | 0.93395 |
| 30 | 0.922109 | 0.591355 | -0.33075 | 0.444735 | 0.503586 | 0.950576 |
| 31 | 0.937188 | 0.550916 | -0.38627 | 0.44022 | 0.508096 | 0.933336 |
| 32 | 1.10528 | 11.3673 | 10.262 | 0.958824 | 0.003241 | 27.6366 |
| 33 | 0.923216 | 0.665619 | -0.2576 | 0.450718 | 0.497736 | 0.973859 |
| 34 | 0.793952 | 0.549915 | -0.24404 | 0.450038 | 0.497628 | 0.971188 |
| 35 | 0.944482 | 0.588393 | -0.35609 | 0.442967 | 0.505442 | 0.943793 |
| 36 | 0.922109 | 0.591355 | -0.33075 | 0.444735 | 0.503586 | 0.950576 |
| 37 | 0.786362 | 0.596037 | -0.19033 | 0.454599 | 0.493162 | 0.989233 |
| 38 | 0.960912 | 0.604384 | -0.35653 | 0.44319 | 0.505311 | 0.944645 |
| 39 | 0.98516 | 0.666199 | -0.31896 | 0.44659 | 0.502087 | 0.957741 |
| 40 | 0.804821 | 0.579215 | -0.22561 | 0.451834 | 0.495961 | 0.978257 |
| 41 | 0.921714 | 0.564763 | -0.35695 | 0.442469 | 0.505802 | 0.941888 |
| 42 | 0.984237 | 0.609548 | -0.37469 | 0.442085 | 0.506516 | 0.940424 |
| 43 | 0.923216 | 0.665619 | -0.2576 | 0.450718 | 0.497736 | 0.973859 |
| 44 | 0.793603 | 0.520671 | -0.27293 | 0.447416 | 0.500167 | 0.960948 |
| 45 | 0.923216 | 0.665619 | -0.2576 | 0.450718 | 0.497736 | 0.973859 |
| 46 | 0.984237 | 0.609548 | -0.37469 | 0.442085 | 0.506516 | 0.940424 |
| 47 | 0.937218 | 0.552927 | -0.38429 | 0.440382 | 0.507937 | 0.93395 |
| 48 | 0.891537 | 0.550596 | -0.34094 | 0.443293 | 0.504818 | 0.94504 |
| 49 | 0.923216 | 0.665619 | -0.2576 | 0.450718 | 0.497736 | 0.973859 |
| 50 | 0.8902 | 0.455904 | -0.4343 | 0.434536 | 0.51337 | 0.912026 |
| 51 | 0.923216 | 0.665619 | -0.2576 | 0.450718 | 0.497736 | 0.973859 |
| 52 | 0.923216 | 0.665619 | -0.2576 | 0.450718 | 0.497736 | 0.973859 |
| 53 | 0.923216 | 0.665619 | -0.2576 | 0.450718 | 0.497736 | 0.973859 |
| 54 | 1.03968 | 5.42066 | 4.38098 | 0.839625 | 0.11628 | 6.21348 |
| 55 | 0.921714 | 0.564763 | -0.35695 | 0.442469 | 0.505802 | 0.941888 |
| 56 | 0.923216 | 0.665619 | -0.2576 | 0.450718 | 0.497736 | 0.973859 |
| 57 | 0.855042 | 0.548882 | -0.30616 | 0.445651 | 0.502292 | 0.954108 |
| 58 | 0.923216 | 0.665619 | -0.2576 | 0.450718 | 0.497736 | 0.973859 |
| 59 | 0.628247 | 0.569198 | -0.05905 | 0.464386 | 0.482578 | 1.029 |
| 60 | 0.889149 | 0.539245 | -0.3499 | 0.442442 | 0.505635 | 0.941786 |
| 61 | 0.670282 | 0.536524 | -0.13376 | 0.458115 | 0.488921 | 1.00335 |
| 62 | 0.984237 | 0.609548 | -0.37469 | 0.442085 | 0.506516 | 0.940424 |
| 63 | 0.944482 | 0.588393 | -0.35609 | 0.442967 | 0.505442 | 0.943793 |
| 64 | 0.923216 | 0.665619 | -0.2576 | 0.450718 | 0.497736 | 0.973859 |
| 65 | 0.960912 | 0.604384 | -0.35653 | 0.44319 | 0.505311 | 0.944645 |
| 66 | 0.919919 | 0.443482 | -0.47644 | 0.431305 | 0.516731 | 0.900102 |
| 67 | 0.922879 | 0.642984 | -0.2799 | 0.448919 | 0.499495 | 0.966805 |
| 68 | 0.922109 | 0.591355 | -0.33075 | 0.444735 | 0.503586 | 0.950576 |
| 69 | 0.888741 | 0.350946 | -0.5378 | 0.422967 | 0.524691 | 0.869945 |
| 70 | 0.923216 | 0.665619 | -0.2576 | 0.450718 | 0.497736 | 0.973859 |
| 71 | 0.923216 | 0.665619 | -0.2576 | 0.450718 | 0.497736 | 0.973859 |
| 72 | 0.865573 | 0.644353 | -0.22122 | 0.45297 | 0.49523 | 0.982751 |
| 73 | 0.922879 | 0.642984 | -0.2799 | 0.448919 | 0.499495 | 0.966805 |
| 74 | 0.923216 | 0.665619 | -0.2576 | 0.450718 | 0.497736 | 0.973859 |
| 75 | 0.960912 | 0.604384 | -0.35653 | 0.44319 | 0.505311 | 0.944645 |
| 76 | 0.944482 | 0.588393 | -0.35609 | 0.442967 | 0.505442 | 0.943793 |
| 77 | 0.98361 | 0.571041 | -0.41257 | 0.438895 | 0.509653 | 0.92833 |
| 78 | 0.670981 | 5.19424 | 4.52326 | 0.867756 | 0.09818 | 7.78767 |
| 79 | 0.791603 | 0.350689 | -0.44092 | 0.429549 | 0.517515 | 0.893675 |
| 80 | 0.944482 | 0.588393 | -0.35609 | 0.442967 | 0.505442 | 0.943793 |
| 81 | 0.919919 | 0.443482 | -0.47644 | 0.431305 | 0.516731 | 0.900102 |
| 82 | 0.88964 | 0.57404 | -0.3156 | 0.445448 | 0.502703 | 0.953324 |
| 83 | 0.628247 | 0.569198 | -0.05905 | 0.464386 | 0.482578 | 1.029 |
| 84 | 0.904336 | 0.609251 | -0.29509 | 0.447423 | 0.500858 | 0.960972 |
| 85 | 0.889935 | 0.437014 | -0.45292 | 0.432649 | 0.515214 | 0.905046 |
| 86 | 0.9371 | 0.545132 | -0.39197 | 0.439732 | 0.508574 | 0.93149 |
| 87 | 0.944482 | 0.588393 | -0.35609 | 0.442967 | 0.505442 | 0.943793 |
| 88 | 0.793603 | 0.520671 | -0.27293 | 0.447416 | 0.500167 | 0.960948 |
| 89 | 0.922879 | 0.642984 | -0.2799 | 0.448919 | 0.499495 | 0.966805 |
| 90 | 0.937188 | 0.550916 | -0.38627 | 0.44022 | 0.508096 | 0.933336 |
| 91 | 0.92178 | 0.569216 | -0.35256 | 0.442876 | 0.505404 | 0.943443 |
| 92 | 0.944482 | 0.588393 | -0.35609 | 0.442967 | 0.505442 | 0.943793 |
| 93 | 0.923216 | 0.665619 | -0.2576 | 0.450718 | 0.497736 | 0.973859 |
| 94 | 0.934182 | 0.351073 | -0.58311 | 0.419972 | 0.52796 | 0.859326 |
| 95 | 0.923216 | 0.665619 | -0.2576 | 0.450718 | 0.497736 | 0.973859 |
| 96 | 0.984237 | 0.609548 | -0.37469 | 0.442085 | 0.506516 | 0.940424 |
| 97 | 0.921714 | 0.564763 | -0.35695 | 0.442469 | 0.505802 | 0.941888 |
| 98 | 0.8902 | 0.455904 | -0.4343 | 0.434536 | 0.51337 | 0.912026 |
| 99 | 0.92178 | 0.569216 | -0.35256 | 0.442876 | 0.505404 | 0.943443 |
| 100 | 0.922109 | 0.591355 | -0.33075 | 0.444735 | 0.503586 | 0.950576 |
| 101 | 0.904336 | 0.609251 | -0.29509 | 0.447423 | 0.500858 | 0.960972 |
| 102 | 1.00702 | 0.508968 | -0.49805 | 0.431874 | 0.5167 | 0.902192 |
| 103 | 0.960912 | 0.604384 | -0.35653 | 0.44319 | 0.505311 | 0.944645 |
| 104 | 0.923216 | 0.665619 | -0.2576 | 0.450718 | 0.497736 | 0.973859 |
| 105 | 0.944482 | 0.588393 | -0.35609 | 0.442967 | 0.505442 | 0.943793 |
| 106 | 0.923216 | 0.665619 | -0.2576 | 0.450718 | 0.497736 | 0.973859 |
| 107 | 0.923216 | 0.665619 | -0.2576 | 0.450718 | 0.497736 | 0.973859 |
| 108 | 0.793952 | 0.549915 | -0.24404 | 0.450038 | 0.497628 | 0.971188 |
| 109 | 0.919919 | 0.443482 | -0.47644 | 0.431305 | 0.516731 | 0.900102 |
| 110 | 0.922109 | 0.591355 | -0.33075 | 0.444735 | 0.503586 | 0.950576 |
| 111 | 0.984237 | 0.609548 | -0.37469 | 0.442085 | 0.506516 | 0.940424 |
| 112 | 0.98361 | 0.571041 | -0.41257 | 0.438895 | 0.509653 | 0.92833 |
| 113 | 0.889474 | 0.403981 | -0.48549 | 0.429167 | 0.51862 | 0.892285 |
| 114 | 0.793952 | 0.549915 | -0.24404 | 0.450038 | 0.497628 | 0.971188 |
| 115 | 6.70694 | 0.644831 | -6.06211 | 0.059207 | 0.913115 | 0.074691 |
| 116 | 0.922109 | 0.591355 | -0.33075 | 0.444735 | 0.503586 | 0.950576 |
| 117 | 0.984237 | 0.609548 | -0.37469 | 0.442085 | 0.506516 | 0.940424 |
| 118 | 0.9371 | 0.545132 | -0.39197 | 0.439732 | 0.508574 | 0.93149 |
| 119 | 0.944482 | 0.588393 | -0.35609 | 0.442967 | 0.505442 | 0.943793 |
| 120 | 0.984237 | 0.609548 | -0.37469 | 0.442085 | 0.506516 | 0.940424 |
| 121 | 0.628247 | 0.569198 | -0.05905 | 0.464386 | 0.482578 | 1.029 |
| 122 | 0.923216 | 0.665619 | -0.2576 | 0.450718 | 0.497736 | 0.973859 |
| 123 | 0.804821 | 0.579215 | -0.22561 | 0.451834 | 0.495961 | 0.978257 |
| 124 | 0.944482 | 0.588393 | -0.35609 | 0.442967 | 0.505442 | 0.943793 |
| 125 | 0.92178 | 0.569216 | -0.35256 | 0.442876 | 0.505404 | 0.943443 |
| 126 | 0.8902 | 0.455904 | -0.4343 | 0.434536 | 0.51337 | 0.912026 |
| 127 | 0.891537 | 0.550596 | -0.34094 | 0.443293 | 0.504818 | 0.94504 |
| 128 | 0.934182 | 0.351073 | -0.58311 | 0.419972 | 0.52796 | 0.859326 |
| 129 | 0.923216 | 0.665619 | -0.2576 | 0.450718 | 0.497736 | 0.973859 |
| 130 | 0.923216 | 0.665619 | -0.2576 | 0.450718 | 0.497736 | 0.973859 |
| 131 | 0.935467 | 0.437218 | -0.49825 | 0.429614 | 0.518493 | 0.893913 |
| 132 | 0.919919 | 0.443482 | -0.47644 | 0.431305 | 0.516731 | 0.900102 |
| 133 | 0.936102 | 0.479328 | -0.45677 | 0.433744 | 0.514443 | 0.909089 |
| 134 | 0.532767 | 0.454176 | -0.07859 | 0.461655 | 0.484272 | 1.01776 |
| 135 | 0.935467 | 0.437218 | -0.49825 | 0.429614 | 0.518493 | 0.893913 |
| 136 | 0.964282 | 5.56273 | 4.59844 | 0.850466 | 0.107825 | 6.75 |
| 137 | 1.00117 | 5.6468 | 4.64563 | 0.849446 | 0.10791 | 6.69623 |
| 138 | 0.922879 | 0.642984 | -0.2799 | 0.448919 | 0.499495 | 0.966805 |
| 139 | 0.533183 | 0.522322 | -0.01086 | 0.46829 | 0.477986 | 1.04526 |
| 140 | 0.92178 | 0.569216 | -0.35256 | 0.442876 | 0.505404 | 0.943443 |
| 141 | 0.627774 | 0.51183 | -0.11594 | 0.459232 | 0.487493 | 1.00788 |
| 142 | 0.98361 | 0.571041 | -0.41257 | 0.438895 | 0.509653 | 0.92833 |
| 143 | 0.88964 | 0.57404 | -0.3156 | 0.445448 | 0.502703 | 0.953324 |
| 144 | 0.904336 | 0.609251 | -0.29509 | 0.447423 | 0.500858 | 0.960972 |
| 145 | 1.00702 | 0.508968 | -0.49805 | 0.431874 | 0.5167 | 0.902192 |
| 146 | 0.98361 | 0.571041 | -0.41257 | 0.438895 | 0.509653 | 0.92833 |
| 147 | 0.923216 | 0.665619 | -0.2576 | 0.450718 | 0.497736 | 0.973859 |
| 148 | 0.890364 | 0.625168 | -0.2652 | 0.449682 | 0.498575 | 0.96979 |
| 149 | 0.888741 | 0.350946 | -0.5378 | 0.422967 | 0.524691 | 0.869945 |
| 150 | 0.891537 | 0.550596 | -0.34094 | 0.443293 | 0.504818 | 0.94504 |
| 151 | 0.923216 | 0.665619 | -0.2576 | 0.450718 | 0.497736 | 0.973859 |
| 152 | 0.923216 | 0.665619 | -0.2576 | 0.450718 | 0.497736 | 0.973859 |
| 153 | 0.865573 | 0.644353 | -0.22122 | 0.45297 | 0.49523 | 0.982751 |
| 154 | 0.919919 | 0.443482 | -0.47644 | 0.431305 | 0.516731 | 0.900102 |
| 155 | 0.865573 | 0.644353 | -0.22122 | 0.45297 | 0.49523 | 0.982751 |
| 156 | 1.00983 | 5.10136 | 4.09153 | 0.833167 | 0.123089 | 5.927 |
| 157 | 0.98516 | 0.666199 | -0.31896 | 0.44659 | 0.502087 | 0.957741 |
| 158 | 0.937218 | 0.552927 | -0.38429 | 0.440382 | 0.507937 | 0.93395 |
| 159 | 0.786362 | 0.596037 | -0.19033 | 0.454599 | 0.493162 | 0.989233 |
| 160 | 0.627774 | 0.51183 | -0.11594 | 0.459232 | 0.487493 | 1.00788 |
| 161 | 0.922109 | 0.591355 | -0.33075 | 0.444735 | 0.503586 | 0.950576 |
| 162 | 0.921714 | 0.564763 | -0.35695 | 0.442469 | 0.505802 | 0.941888 |
| 163 | 0.922879 | 0.642984 | -0.2799 | 0.448919 | 0.499495 | 0.966805 |
| 164 | 0.628247 | 0.569198 | -0.05905 | 0.464386 | 0.482578 | 1.029 |
| 165 | 0.923216 | 0.665619 | -0.2576 | 0.450718 | 0.497736 | 0.973859 |
| 166 | 0.865573 | 0.644353 | -0.22122 | 0.45297 | 0.49523 | 0.982751 |
| 167 | 0.891169 | 0.524596 | -0.36657 | 0.441013 | 0.507042 | 0.936346 |
| 168 | 0.891537 | 0.550596 | -0.34094 | 0.443293 | 0.504818 | 0.94504 |
| 169 | 0.92178 | 0.569216 | -0.35256 | 0.442876 | 0.505404 | 0.943443 |
| 170 | 0.937218 | 0.552927 | -0.38429 | 0.440382 | 0.507937 | 0.93395 |
| 171 | 0.627774 | 0.51183 | -0.11594 | 0.459232 | 0.487493 | 1.00788 |
| 172 | 0.922109 | 0.591355 | -0.33075 | 0.444735 | 0.503586 | 0.950576 |
| 173 | 0.921714 | 0.564763 | -0.35695 | 0.442469 | 0.505802 | 0.941888 |
| 174 | 0.984237 | 0.609548 | -0.37469 | 0.442085 | 0.506516 | 0.940424 |
| 175 | 0.922109 | 0.591355 | -0.33075 | 0.444735 | 0.503586 | 0.950576 |
| 176 | 0.922109 | 0.591355 | -0.33075 | 0.444735 | 0.503586 | 0.950576 |
| 177 | 0.984237 | 0.609548 | -0.37469 | 0.442085 | 0.506516 | 0.940424 |
| 178 | 0.98361 | 0.571041 | -0.41257 | 0.438895 | 0.509653 | 0.92833 |
| 179 | 0.891537 | 0.550596 | -0.34094 | 0.443293 | 0.504818 | 0.94504 |
| 180 | 0.923216 | 0.665619 | -0.2576 | 0.450718 | 0.497736 | 0.973859 |
| 181 | 0.922879 | 0.642984 | -0.2799 | 0.448919 | 0.499495 | 0.966805 |
| 182 | 0.923216 | 0.665619 | -0.2576 | 0.450718 | 0.497736 | 0.973859 |
| 183 | 0.984237 | 0.609548 | -0.37469 | 0.442085 | 0.506516 | 0.940424 |
| 184 | 0.923216 | 0.665619 | -0.2576 | 0.450718 | 0.497736 | 0.973859 |
| 185 | 0.922109 | 0.591355 | -0.33075 | 0.444735 | 0.503586 | 0.950576 |
| 186 | 0.889935 | 0.437014 | -0.45292 | 0.432649 | 0.515214 | 0.905046 |
| 187 | 0.627605 | 0.491184 | -0.13642 | 0.457261 | 0.489376 | 0.999905 |
| 188 | 0.92178 | 0.569216 | -0.35256 | 0.442876 | 0.505404 | 0.943443 |
| 189 | 0.8902 | 0.455904 | -0.4343 | 0.434536 | 0.51337 | 0.912026 |
| 190 | 0.921714 | 0.564763 | -0.35695 | 0.442469 | 0.505802 | 0.941888 |
| 191 | 0.937218 | 0.552927 | -0.38429 | 0.440382 | 0.507937 | 0.93395 |
| 192 | 0.919919 | 0.443482 | -0.47644 | 0.431305 | 0.516731 | 0.900102 |
| 193 | 0.923216 | 0.665619 | -0.2576 | 0.450718 | 0.497736 | 0.973859 |
| 194 | 0.628247 | 0.569198 | -0.05905 | 0.464386 | 0.482578 | 1.029 |
| 195 | 0.98361 | 0.571041 | -0.41257 | 0.438895 | 0.509653 | 0.92833 |
| 196 | 0.923216 | 0.665619 | -0.2576 | 0.450718 | 0.497736 | 0.973859 |
| 197 | 0.922109 | 0.591355 | -0.33075 | 0.444735 | 0.503586 | 0.950576 |
| 198 | 0.922109 | 0.591355 | -0.33075 | 0.444735 | 0.503586 | 0.950576 |
| 199 | 0.936102 | 0.479328 | -0.45677 | 0.433744 | 0.514443 | 0.909089 |
| 200 | 0.890526 | 0.479082 | -0.41144 | 0.436795 | 0.511162 | 0.920443 |
| 201 | 0.889689 | 0.41941 | -0.47028 | 0.430813 | 0.51701 | 0.898296 |
| 202 | 0.865573 | 0.644353 | -0.22122 | 0.45297 | 0.49523 | 0.982751 |
| 203 | 0.922879 | 0.642984 | -0.2799 | 0.448919 | 0.499495 | 0.966805 |
| 204 | 0.922109 | 0.591355 | -0.33075 | 0.444735 | 0.503586 | 0.950576 |
| 205 | 0.984237 | 0.609548 | -0.37469 | 0.442085 | 0.506516 | 0.940424 |
| 206 | 0.921714 | 0.564763 | -0.35695 | 0.442469 | 0.505802 | 0.941888 |
| 207 | 0.984237 | 0.609548 | -0.37469 | 0.442085 | 0.506516 | 0.940424 |
| 208 | 0.922879 | 0.642984 | -0.2799 | 0.448919 | 0.499495 | 0.966805 |
| 209 | 0.960273 | 5.09738 | 4.13711 | 0.837595 | 0.119971 | 6.12096 |
| 210 | 0.627774 | 0.51183 | -0.11594 | 0.459232 | 0.487493 | 1.00788 |
| 211 | 0.92178 | 0.569216 | -0.35256 | 0.442876 | 0.505404 | 0.943443 |
| 212 | 0.923216 | 0.665619 | -0.2576 | 0.450718 | 0.497736 | 0.973859 |
| 213 | 0.923216 | 0.665619 | -0.2576 | 0.450718 | 0.497736 | 0.973859 |
| 214 | 0.922879 | 0.642984 | -0.2799 | 0.448919 | 0.499495 | 0.966805 |
| 215 | 0.890526 | 0.479082 | -0.41144 | 0.436795 | 0.511162 | 0.920443 |
| 216 | 0.944482 | 0.588393 | -0.35609 | 0.442967 | 0.505442 | 0.943793 |
| 217 | 0.923216 | 0.665619 | -0.2576 | 0.450718 | 0.497736 | 0.973859 |
| 218 | 0.891169 | 0.524596 | -0.36657 | 0.441013 | 0.507042 | 0.936346 |
| 219 | 0.92178 | 0.569216 | -0.35256 | 0.442876 | 0.505404 | 0.943443 |
| 220 | 0.92178 | 0.569216 | -0.35256 | 0.442876 | 0.505404 | 0.943443 |
| 221 | 0.92178 | 0.569216 | -0.35256 | 0.442876 | 0.505404 | 0.943443 |
| 222 | 0.92178 | 0.569216 | -0.35256 | 0.442876 | 0.505404 | 0.943443 |
| 223 | 1.00886 | 0.758821 | -0.25004 | 0.452112 | 0.49675 | 0.979357 |
| 224 | 1.01254 | 0.836248 | -0.17629 | 0.457515 | 0.491437 | 1.00093 |
| 225 | 1.01254 | 0.836248 | -0.17629 | 0.457515 | 0.491437 | 1.00093 |
| 226 | 1.01254 | 0.836248 | -0.17629 | 0.457515 | 0.491437 | 1.00093 |
| 227 | 1.01254 | 0.836248 | -0.17629 | 0.457515 | 0.491437 | 1.00093 |
| 228 | 1.01254 | 0.836248 | -0.17629 | 0.457515 | 0.491437 | 1.00093 |
| 229 | 1.01254 | 0.836248 | -0.17629 | 0.457515 | 0.491437 | 1.00093 |
| 230 | 1.01254 | 0.836248 | -0.17629 | 0.457515 | 0.491437 | 1.00093 |
| 231 | 1.01254 | 0.836248 | -0.17629 | 0.457515 | 0.491437 | 1.00093 |
| 232 | 0.923216 | 0.665619 | -0.2576 | 0.450718 | 0.497736 | 0.973859 |
| 233 | 0.904336 | 0.609251 | -0.29509 | 0.447423 | 0.500858 | 0.960972 |
| 234 | 0.891169 | 0.524596 | -0.36657 | 0.441013 | 0.507042 | 0.936346 |
| 235 | 0.923216 | 0.665619 | -0.2576 | 0.450718 | 0.497736 | 0.973859 |
| 236 | 0.923216 | 0.665619 | -0.2576 | 0.450718 | 0.497736 | 0.973859 |
| 237 | 0.9371 | 0.545132 | -0.39197 | 0.439732 | 0.508574 | 0.93149 |
| 238 | 0.98516 | 0.666199 | -0.31896 | 0.44659 | 0.502087 | 0.957741 |
| 239 | 0.555143 | 0.586251 | 0.031108 | 0.472036 | 0.474659 | 1.0611 |
| 240 | 0.793102 | 0.478555 | -0.31455 | 0.443494 | 0.503966 | 0.94581 |
| 241 | 0.922879 | 0.642984 | -0.2799 | 0.448919 | 0.499495 | 0.966805 |
| 242 | 0.923216 | 0.665619 | -0.2576 | 0.450718 | 0.497736 | 0.973859 |
| 243 | 0.984237 | 0.609548 | -0.37469 | 0.442085 | 0.506516 | 0.940424 |
| 244 | 0.923216 | 0.665619 | -0.2576 | 0.450718 | 0.497736 | 0.973859 |
| 245 | 0.922109 | 0.591355 | -0.33075 | 0.444735 | 0.503586 | 0.950576 |
| 246 | 0.937188 | 0.550916 | -0.38627 | 0.44022 | 0.508096 | 0.933336 |
| 247 | 0.8902 | 0.455904 | -0.4343 | 0.434536 | 0.51337 | 0.912026 |
| 248 | 0.922879 | 0.642984 | -0.2799 | 0.448919 | 0.499495 | 0.966805 |
| 249 | 0.923216 | 0.665619 | -0.2576 | 0.450718 | 0.497736 | 0.973859 |
| 250 | 0.922109 | 0.591355 | -0.33075 | 0.444735 | 0.503586 | 0.950576 |
| 251 | 0.937218 | 0.552927 | -0.38429 | 0.440382 | 0.507937 | 0.93395 |
| 252 | 0.923216 | 0.665619 | -0.2576 | 0.450718 | 0.497736 | 0.973859 |
| 253 | 0.923216 | 0.665619 | -0.2576 | 0.450718 | 0.497736 | 0.973859 |
| 254 | 0.533183 | 0.522322 | -0.01086 | 0.46829 | 0.477986 | 1.04526 |
| 255 | 0.923216 | 0.665619 | -0.2576 | 0.450718 | 0.497736 | 0.973859 |
| 256 | 0.937218 | 0.552927 | -0.38429 | 0.440382 | 0.507937 | 0.93395 |
| 257 | 0.532907 | 0.477172 | -0.05573 | 0.463972 | 0.482075 | 1.02728 |
| 258 | 0.941243 | 3.87291 | 2.93167 | 0.800858 | 0.154948 | 4.77285 |
| 259 | 0.937218 | 0.552927 | -0.38429 | 0.440382 | 0.507937 | 0.93395 |
| 260 | 0.923216 | 0.665619 | -0.2576 | 0.450718 | 0.497736 | 0.973859 |
| 261 | 0.960912 | 0.604384 | -0.35653 | 0.44319 | 0.505311 | 0.944645 |
| 262 | 0.919919 | 0.443482 | -0.47644 | 0.431305 | 0.516731 | 0.900102 |
| 263 | 0.944482 | 0.588393 | -0.35609 | 0.442967 | 0.505442 | 0.943793 |
| 264 | 0.922109 | 0.591355 | -0.33075 | 0.444735 | 0.503586 | 0.950576 |
| 265 | 0.923216 | 0.665619 | -0.2576 | 0.450718 | 0.497736 | 0.973859 |
| 266 | 0.890364 | 0.625168 | -0.2652 | 0.449682 | 0.498575 | 0.96979 |
| 267 | 0.960912 | 0.604384 | -0.35653 | 0.44319 | 0.505311 | 0.944645 |
| 268 | 0.98516 | 0.666199 | -0.31896 | 0.44659 | 0.502087 | 0.957741 |
| 269 | 0.934182 | 0.351073 | -0.58311 | 0.419972 | 0.52796 | 0.859326 |
| 270 | 0.923216 | 0.665619 | -0.2576 | 0.450718 | 0.497736 | 0.973859 |
| 271 | 0.865573 | 0.644353 | -0.22122 | 0.45297 | 0.49523 | 0.982751 |
| 272 | 0.937218 | 0.552927 | -0.38429 | 0.440382 | 0.507937 | 0.93395 |
| 273 | 0.937218 | 0.552927 | -0.38429 | 0.440382 | 0.507937 | 0.93395 |
| 274 | 0.670282 | 0.536524 | -0.13376 | 0.458115 | 0.488921 | 1.00335 |
| 275 | 0.922879 | 0.642984 | -0.2799 | 0.448919 | 0.499495 | 0.966805 |
| 276 | 0.533183 | 0.522322 | -0.01086 | 0.46829 | 0.477986 | 1.04526 |
| 277 | 0.923216 | 0.665619 | -0.2576 | 0.450718 | 0.497736 | 0.973859 |
| 278 | 0.98516 | 0.666199 | -0.31896 | 0.44659 | 0.502087 | 0.957741 |
| 279 | 0.793102 | 0.478555 | -0.31455 | 0.443494 | 0.503966 | 0.94581 |
| 280 | 0.921714 | 0.564763 | -0.35695 | 0.442469 | 0.505802 | 0.941888 |
| 281 | 0.923216 | 0.665619 | -0.2576 | 0.450718 | 0.497736 | 0.973859 |
| 282 | 0.923216 | 0.665619 | -0.2576 | 0.450718 | 0.497736 | 0.973859 |
| 283 | 0.922879 | 0.642984 | -0.2799 | 0.448919 | 0.499495 | 0.966805 |
| 284 | 0.937218 | 0.552927 | -0.38429 | 0.440382 | 0.507937 | 0.93395 |
| 285 | 0.923216 | 0.665619 | -0.2576 | 0.450718 | 0.497736 | 0.973859 |
| 286 | 0.8902 | 0.455904 | -0.4343 | 0.434536 | 0.51337 | 0.912026 |
| 287 | 0.890526 | 0.479082 | -0.41144 | 0.436795 | 0.511162 | 0.920443 |
| 288 | 0.921714 | 0.564763 | -0.35695 | 0.442469 | 0.505802 | 0.941888 |
| 289 | 0.923216 | 0.665619 | -0.2576 | 0.450718 | 0.497736 | 0.973859 |
| 290 | 0.922109 | 0.591355 | -0.33075 | 0.444735 | 0.503586 | 0.950576 |
| 291 | 0.937188 | 0.550916 | -0.38627 | 0.44022 | 0.508096 | 0.933336 |
| 292 | 0.937218 | 0.552927 | -0.38429 | 0.440382 | 0.507937 | 0.93395 |
| 293 | 0.923216 | 0.665619 | -0.2576 | 0.450718 | 0.497736 | 0.973859 |
| 294 | 0.923216 | 0.665619 | -0.2576 | 0.450718 | 0.497736 | 0.973859 |
| 295 | 0.922879 | 0.642984 | -0.2799 | 0.448919 | 0.499495 | 0.966805 |
| 296 | 0.923216 | 0.665619 | -0.2576 | 0.450718 | 0.497736 | 0.973859 |
| 297 | 0.944482 | 0.588393 | -0.35609 | 0.442967 | 0.505442 | 0.943793 |
| 298 | 0.627774 | 0.51183 | -0.11594 | 0.459232 | 0.487493 | 1.00788 |
| 299 | 0.922109 | 0.591355 | -0.33075 | 0.444735 | 0.503586 | 0.950576 |
| 300 | 0.984237 | 0.609548 | -0.37469 | 0.442085 | 0.506516 | 0.940424 |
| 301 | 0.904336 | 0.609251 | -0.29509 | 0.447423 | 0.500858 | 0.960972 |
| 302 | 0.936102 | 0.479328 | -0.45677 | 0.433744 | 0.514443 | 0.909089 |
| 303 | 0.888741 | 0.350946 | -0.5378 | 0.422967 | 0.524691 | 0.869945 |
| 304 | 0.921714 | 0.564763 | -0.35695 | 0.442469 | 0.505802 | 0.941888 |
| 305 | 0.923216 | 0.665619 | -0.2576 | 0.450718 | 0.497736 | 0.973859 |
| 306 | 0.923216 | 0.665619 | -0.2576 | 0.450718 | 0.497736 | 0.973859 |
| 307 | 0.923216 | 0.665619 | -0.2576 | 0.450718 | 0.497736 | 0.973859 |
| 308 | 0.804821 | 0.579215 | -0.22561 | 0.451834 | 0.495961 | 0.978257 |
| 309 | 5.3454 | 0.614964 | -4.73043 | 0.084706 | 0.884251 | 0.109835 |
| 310 | 0.891537 | 0.550596 | -0.34094 | 0.443293 | 0.504818 | 0.94504 |
| 311 | 0.922879 | 0.642984 | -0.2799 | 0.448919 | 0.499495 | 0.966805 |
| 312 | 0.891537 | 0.550596 | -0.34094 | 0.443293 | 0.504818 | 0.94504 |
| 313 | 0.934182 | 0.351073 | -0.58311 | 0.419972 | 0.52796 | 0.859326 |
| 314 | 0.937218 | 0.552927 | -0.38429 | 0.440382 | 0.507937 | 0.93395 |
| 315 | 0.984237 | 0.609548 | -0.37469 | 0.442085 | 0.506516 | 0.940424 |
| 316 | 0.922879 | 0.642984 | -0.2799 | 0.448919 | 0.499495 | 0.966805 |
| 317 | 0.8902 | 0.455904 | -0.4343 | 0.434536 | 0.51337 | 0.912026 |
| 318 | 0.628247 | 0.569198 | -0.05905 | 0.464386 | 0.482578 | 1.029 |
| 319 | 0.931211 | 3.21588 | 2.28466 | 0.778227 | 0.176563 | 4.1647 |
| 320 | 0.865573 | 0.644353 | -0.22122 | 0.45297 | 0.49523 | 0.982751 |
| 321 | 0.627774 | 0.51183 | -0.11594 | 0.459232 | 0.487493 | 1.00788 |
| 322 | 0.889935 | 0.437014 | -0.45292 | 0.432649 | 0.515214 | 0.905046 |
| 323 | 0.922879 | 0.642984 | -0.2799 | 0.448919 | 0.499495 | 0.966805 |
| 324 | 0.793952 | 0.549915 | -0.24404 | 0.450038 | 0.497628 | 0.971188 |
| 325 | 0.919919 | 0.443482 | -0.47644 | 0.431305 | 0.516731 | 0.900102 |
| 326 | 0.934182 | 0.351073 | -0.58311 | 0.419972 | 0.52796 | 0.859326 |
| 327 | 0.923216 | 0.665619 | -0.2576 | 0.450718 | 0.497736 | 0.973859 |
| 328 | 0.923216 | 0.665619 | -0.2576 | 0.450718 | 0.497736 | 0.973859 |
| 329 | 0.98361 | 0.571041 | -0.41257 | 0.438895 | 0.509653 | 0.92833 |
| 330 | 0.889149 | 0.539245 | -0.3499 | 0.442442 | 0.505635 | 0.941786 |
| 331 | 0.921714 | 0.564763 | -0.35695 | 0.442469 | 0.505802 | 0.941888 |
| 332 | 0.8902 | 0.455904 | -0.4343 | 0.434536 | 0.51337 | 0.912026 |
| 333 | 0.936102 | 0.479328 | -0.45677 | 0.433744 | 0.514443 | 0.909089 |
| 334 | 0.98361 | 0.571041 | -0.41257 | 0.438895 | 0.509653 | 0.92833 |
| 335 | 0.889149 | 0.539245 | -0.3499 | 0.442442 | 0.505635 | 0.941786 |
| 336 | 0.628835 | 0.64031 | 0.011475 | 0.470349 | 0.476905 | 1.05394 |
| 337 | 0.891169 | 0.524596 | -0.36657 | 0.441013 | 0.507042 | 0.936346 |
| 338 | 0.923216 | 0.665619 | -0.2576 | 0.450718 | 0.497736 | 0.973859 |
| 339 | 0.923216 | 0.665619 | -0.2576 | 0.450718 | 0.497736 | 0.973859 |
| 340 | 1.00702 | 0.508968 | -0.49805 | 0.431874 | 0.5167 | 0.902192 |
| 341 | 0.984237 | 0.609548 | -0.37469 | 0.442085 | 0.506516 | 0.940424 |
| 342 | 0.984237 | 0.609548 | -0.37469 | 0.442085 | 0.506516 | 0.940424 |
| 343 | 1.00702 | 0.508968 | -0.49805 | 0.431874 | 0.5167 | 0.902192 |
| 344 | 0.923216 | 0.665619 | -0.2576 | 0.450718 | 0.497736 | 0.973859 |
| 345 | 1.12714 | 9.09549 | 7.96835 | 0.93313 | 0.024784 | 16.5613 |
| 346 | 0.865573 | 0.644353 | -0.22122 | 0.45297 | 0.49523 | 0.982751 |
| 347 | 0.944482 | 0.588393 | -0.35609 | 0.442967 | 0.505442 | 0.943793 |
| 348 | 0.944482 | 0.588393 | -0.35609 | 0.442967 | 0.505442 | 0.943793 |
| 349 | 0.98516 | 0.666199 | -0.31896 | 0.44659 | 0.502087 | 0.957741 |
| 350 | 0.937188 | 0.550916 | -0.38627 | 0.44022 | 0.508096 | 0.933336 |
| 351 | 0.923216 | 0.665619 | -0.2576 | 0.450718 | 0.497736 | 0.973859 |
| 352 | 0.627605 | 0.491184 | -0.13642 | 0.457261 | 0.489376 | 0.999905 |
| 353 | 0.935467 | 0.437218 | -0.49825 | 0.429614 | 0.518493 | 0.893913 |
| 354 | 0.934182 | 0.351073 | -0.58311 | 0.419972 | 0.52796 | 0.859326 |
| 355 | 0.889474 | 0.403981 | -0.48549 | 0.429167 | 0.51862 | 0.892285 |
| 356 | 0.922109 | 0.591355 | -0.33075 | 0.444735 | 0.503586 | 0.950576 |
| 357 | 0.904336 | 0.609251 | -0.29509 | 0.447423 | 0.500858 | 0.960972 |
| 358 | 0.922879 | 0.642984 | -0.2799 | 0.448919 | 0.499495 | 0.966805 |
| 359 | 0.890526 | 0.479082 | -0.41144 | 0.436795 | 0.511162 | 0.920443 |
| 360 | 0.793952 | 0.549915 | -0.24404 | 0.450038 | 0.497628 | 0.971188 |
| 361 | 0.923216 | 0.665619 | -0.2576 | 0.450718 | 0.497736 | 0.973859 |
| 362 | 0.923216 | 0.665619 | -0.2576 | 0.450718 | 0.497736 | 0.973859 |
| 363 | 0.8902 | 0.455904 | -0.4343 | 0.434536 | 0.51337 | 0.912026 |
| 364 | 0.865573 | 0.644353 | -0.22122 | 0.45297 | 0.49523 | 0.982751 |
| 365 | 0.922109 | 0.591355 | -0.33075 | 0.444735 | 0.503586 | 0.950576 |
| 366 | 0.9371 | 0.545132 | -0.39197 | 0.439732 | 0.508574 | 0.93149 |
| 367 | 0.97858 | 4.20422 | 3.22564 | 0.808339 | 0.147269 | 5.00549 |
| 368 | 0.937218 | 0.552927 | -0.38429 | 0.440382 | 0.507937 | 0.93395 |
| 369 | 0.960912 | 0.604384 | -0.35653 | 0.44319 | 0.505311 | 0.944645 |
| 370 | 0.923216 | 0.665619 | -0.2576 | 0.450718 | 0.497736 | 0.973859 |
| 371 | 0.923216 | 0.665619 | -0.2576 | 0.450718 | 0.497736 | 0.973859 |
| 372 | 0.923216 | 0.665619 | -0.2576 | 0.450718 | 0.497736 | 0.973859 |
| 373 | 0.936102 | 0.479328 | -0.45677 | 0.433744 | 0.514443 | 0.909089 |
| 374 | 0.934182 | 0.351073 | -0.58311 | 0.419972 | 0.52796 | 0.859326 |
| 375 | 0.865573 | 0.644353 | -0.22122 | 0.45297 | 0.49523 | 0.982751 |
| 376 | 0.923216 | 0.665619 | -0.2576 | 0.450718 | 0.497736 | 0.973859 |
| 377 | 0.923216 | 0.665619 | -0.2576 | 0.450718 | 0.497736 | 0.973859 |
| 378 | 1.00702 | 0.508968 | -0.49805 | 0.431874 | 0.5167 | 0.902192 |
| 379 | 0.922109 | 0.591355 | -0.33075 | 0.444735 | 0.503586 | 0.950576 |
| 380 | 0.889935 | 0.437014 | -0.45292 | 0.432649 | 0.515214 | 0.905046 |
| 381 | 0.891169 | 0.524596 | -0.36657 | 0.441013 | 0.507042 | 0.936346 |
| 382 | 0.53215 | 0.350004 | -0.18215 | 0.44974 | 0.495619 | 0.970018 |
| 383 | 0.92178 | 0.569216 | -0.35256 | 0.442876 | 0.505404 | 0.943443 |
| 384 | 0.628835 | 0.64031 | 0.011475 | 0.470349 | 0.476905 | 1.05394 |
| 385 | 1.00702 | 0.508968 | -0.49805 | 0.431874 | 0.5167 | 0.902192 |
| 386 | 0.936102 | 0.479328 | -0.45677 | 0.433744 | 0.514443 | 0.909089 |
| 387 | 0.984237 | 0.609548 | -0.37469 | 0.442085 | 0.506516 | 0.940424 |
| 388 | 0.9371 | 0.545132 | -0.39197 | 0.439732 | 0.508574 | 0.93149 |
| 389 | 0.923216 | 0.665619 | -0.2576 | 0.450718 | 0.497736 | 0.973859 |
| 390 | 0.98516 | 0.666199 | -0.31896 | 0.44659 | 0.502087 | 0.957741 |
| 391 | 0.923216 | 0.665619 | -0.2576 | 0.450718 | 0.497736 | 0.973859 |
| 392 | 0.937188 | 0.550916 | -0.38627 | 0.44022 | 0.508096 | 0.933336 |
| 393 | 0.923216 | 0.665619 | -0.2576 | 0.450718 | 0.497736 | 0.973859 |
| 394 | 0.98516 | 0.666199 | -0.31896 | 0.44659 | 0.502087 | 0.957741 |
| 395 | 0.923216 | 0.665619 | -0.2576 | 0.450718 | 0.497736 | 0.973859 |
| 396 | 0.960912 | 0.604384 | -0.35653 | 0.44319 | 0.505311 | 0.944645 |
| 397 | 0.628247 | 0.569198 | -0.05905 | 0.464386 | 0.482578 | 1.029 |
| 398 | 0.960912 | 0.604384 | -0.35653 | 0.44319 | 0.505311 | 0.944645 |
| 399 | 0.923216 | 0.665619 | -0.2576 | 0.450718 | 0.497736 | 0.973859 |
| 400 | 0.936102 | 0.479328 | -0.45677 | 0.433744 | 0.514443 | 0.909089 |
| 401 | 0.628247 | 0.569198 | -0.05905 | 0.464386 | 0.482578 | 1.029 |
| 402 | 0.936102 | 0.479328 | -0.45677 | 0.433744 | 0.514443 | 0.909089 |
| 403 | 0.923216 | 0.665619 | -0.2576 | 0.450718 | 0.497736 | 0.973859 |
| 404 | 0.936102 | 0.479328 | -0.45677 | 0.433744 | 0.514443 | 0.909089 |
| 405 | 0.923216 | 0.665619 | -0.2576 | 0.450718 | 0.497736 | 0.973859 |
| 406 | 0.936102 | 0.479328 | -0.45677 | 0.433744 | 0.514443 | 0.909089 |
| 407 | 5.34539 | 0.614963 | -4.73043 | 0.084706 | 0.884251 | 0.109835 |
| 408 | 0.936102 | 0.479328 | -0.45677 | 0.433744 | 0.514443 | 0.909089 |
| 409 | 6.60082 | 0.725657 | -5.87517 | 0.065248 | 0.903753 | 0.082844 |
| 410 | 0.936102 | 0.479328 | -0.45677 | 0.433744 | 0.514443 | 0.909089 |
| 411 | 1.01254 | 0.836248 | -0.17629 | 0.457515 | 0.491437 | 1.00093 |
| 412 | 1.01254 | 0.836248 | -0.17629 | 0.457515 | 0.491437 | 1.00093 |
| 413 | 1.01254 | 0.836248 | -0.17629 | 0.457515 | 0.491437 | 1.00093 |
| 414 | 1.01254 | 0.836248 | -0.17629 | 0.457515 | 0.491437 | 1.00093 |
| 415 | 1.01254 | 0.836248 | -0.17629 | 0.457515 | 0.491437 | 1.00093 |
| 416 | 1.01254 | 0.836248 | -0.17629 | 0.457515 | 0.491437 | 1.00093 |
| 417 | 1.01254 | 0.836248 | -0.17629 | 0.457515 | 0.491437 | 1.00093 |
| 418 | 1.01254 | 0.836248 | -0.17629 | 0.457515 | 0.491437 | 1.00093 |
| 419 | 1.01254 | 0.836248 | -0.17629 | 0.457515 | 0.491437 | 1.00093 |
| 420 | 1.01254 | 0.836248 | -0.17629 | 0.457515 | 0.491437 | 1.00093 |
| 421 | 1.01254 | 0.836248 | -0.17629 | 0.457515 | 0.491437 | 1.00093 |
| 422 | 1.01254 | 0.836248 | -0.17629 | 0.457515 | 0.491437 | 1.00093 |
| 423 | 1.01254 | 0.836248 | -0.17629 | 0.457515 | 0.491437 | 1.00093 |
| 424 | 0.88305 | 0.760196 | -0.12285 | 0.460777 | 0.487744 | 1.01417 |
| 425 | 0.92178 | 0.569216 | -0.35256 | 0.442876 | 0.505404 | 0.943443 |
| 426 | 0.98516 | 0.666199 | -0.31896 | 0.44659 | 0.502087 | 0.957741 |
| 427 | 0.890364 | 0.625168 | -0.2652 | 0.449682 | 0.498575 | 0.96979 |
| 428 | 0.944482 | 0.588393 | -0.35609 | 0.442967 | 0.505442 | 0.943793 |
| 429 | 0.937218 | 0.552927 | -0.38429 | 0.440382 | 0.507937 | 0.93395 |
| 430 | 0.922109 | 0.591355 | -0.33075 | 0.444735 | 0.503586 | 0.950576 |
| 431 | 0.8902 | 0.455904 | -0.4343 | 0.434536 | 0.51337 | 0.912026 |
| 432 | 0.923216 | 0.665619 | -0.2576 | 0.450718 | 0.497736 | 0.973859 |
| 433 | 0.921714 | 0.564763 | -0.35695 | 0.442469 | 0.505802 | 0.941888 |
| 434 | 0.921714 | 0.564763 | -0.35695 | 0.442469 | 0.505802 | 0.941888 |
| 435 | 0.937218 | 0.552927 | -0.38429 | 0.440382 | 0.507937 | 0.93395 |
| 436 | 0.98516 | 0.666199 | -0.31896 | 0.44659 | 0.502087 | 0.957741 |
| 437 | 0.923216 | 0.665619 | -0.2576 | 0.450718 | 0.497736 | 0.973859 |
| 438 | 0.98516 | 0.666199 | -0.31896 | 0.44659 | 0.502087 | 0.957741 |
| 439 | 0.98516 | 0.666199 | -0.31896 | 0.44659 | 0.502087 | 0.957741 |
| 440 | 0.923216 | 0.665619 | -0.2576 | 0.450718 | 0.497736 | 0.973859 |
| 441 | 0.923216 | 0.665619 | -0.2576 | 0.450718 | 0.497736 | 0.973859 |
| 442 | 0.92178 | 0.569216 | -0.35256 | 0.442876 | 0.505404 | 0.943443 |
| 443 | 0.889935 | 0.437014 | -0.45292 | 0.432649 | 0.515214 | 0.905046 |
| 444 | 0.92178 | 0.569216 | -0.35256 | 0.442876 | 0.505404 | 0.943443 |
| 445 | 0.936102 | 0.479328 | -0.45677 | 0.433744 | 0.514443 | 0.909089 |
| 446 | 0.923216 | 0.665619 | -0.2576 | 0.450718 | 0.497736 | 0.973859 |
| 447 | 0.984237 | 0.609548 | -0.37469 | 0.442085 | 0.506516 | 0.940424 |
| 448 | 0.865573 | 0.644353 | -0.22122 | 0.45297 | 0.49523 | 0.982751 |
| 449 | 0.92178 | 0.569216 | -0.35256 | 0.442876 | 0.505404 | 0.943443 |
| 450 | 0.944482 | 0.588393 | -0.35609 | 0.442967 | 0.505442 | 0.943793 |
| 451 | 0.628835 | 0.64031 | 0.011475 | 0.470349 | 0.476905 | 1.05394 |
| 452 | 0.98516 | 0.666199 | -0.31896 | 0.44659 | 0.502087 | 0.957741 |
| 453 | 0.922109 | 0.591355 | -0.33075 | 0.444735 | 0.503586 | 0.950576 |
| 454 | 0.891537 | 0.550596 | -0.34094 | 0.443293 | 0.504818 | 0.94504 |
| 455 | 0.8902 | 0.455904 | -0.4343 | 0.434536 | 0.51337 | 0.912026 |
| 456 | 0.98516 | 0.666199 | -0.31896 | 0.44659 | 0.502087 | 0.957741 |
| 457 | 0.98516 | 0.666199 | -0.31896 | 0.44659 | 0.502087 | 0.957741 |
| 458 | 0.960912 | 0.604384 | -0.35653 | 0.44319 | 0.505311 | 0.944645 |
| 459 | 0.627605 | 0.491184 | -0.13642 | 0.457261 | 0.489376 | 0.999905 |
| 460 | 0.98516 | 0.666199 | -0.31896 | 0.44659 | 0.502087 | 0.957741 |
| 461 | 0.922109 | 0.591355 | -0.33075 | 0.444735 | 0.503586 | 0.950576 |
| 462 | 1.2706 | 8.35794 | 7.08734 | 0.896266 | 0.062356 | 10.2542 |
| 463 | 1.00702 | 0.508968 | -0.49805 | 0.431874 | 0.5167 | 0.902192 |
| 464 | 0.922879 | 0.642984 | -0.2799 | 0.448919 | 0.499495 | 0.966805 |
| 465 | 0.923216 | 0.665619 | -0.2576 | 0.450718 | 0.497736 | 0.973859 |
| 466 | 0.944482 | 0.588393 | -0.35609 | 0.442967 | 0.505442 | 0.943793 |
| 467 | 0.922879 | 0.642984 | -0.2799 | 0.448919 | 0.499495 | 0.966805 |
| 468 | 0.984237 | 0.609548 | -0.37469 | 0.442085 | 0.506516 | 0.940424 |
| 469 | 0.923216 | 0.665619 | -0.2576 | 0.450718 | 0.497736 | 0.973859 |
| 470 | 0.890364 | 0.625168 | -0.2652 | 0.449682 | 0.498575 | 0.96979 |
| 471 | 0.628247 | 0.569198 | -0.05905 | 0.464386 | 0.482578 | 1.029 |
| 472 | 0.628835 | 0.64031 | 0.011475 | 0.470349 | 0.476905 | 1.05394 |
| 473 | 0.888741 | 0.350946 | -0.5378 | 0.422967 | 0.524691 | 0.869945 |
| 474 | 0.98361 | 0.571041 | -0.41257 | 0.438895 | 0.509653 | 0.92833 |
| 475 | 0.92178 | 0.569216 | -0.35256 | 0.442876 | 0.505404 | 0.943443 |
| 476 | 0.793952 | 0.549915 | -0.24404 | 0.450038 | 0.497628 | 0.971188 |
| 477 | 0.628835 | 0.64031 | 0.011475 | 0.470349 | 0.476905 | 1.05394 |
| 478 | 0.793952 | 0.549915 | -0.24404 | 0.450038 | 0.497628 | 0.971188 |
| 479 | 0.627605 | 0.491184 | -0.13642 | 0.457261 | 0.489376 | 0.999905 |
| 480 | 0.923216 | 0.665619 | -0.2576 | 0.450718 | 0.497736 | 0.973859 |
| 481 | 0.984237 | 0.609548 | -0.37469 | 0.442085 | 0.506516 | 0.940424 |
| 482 | 0.922879 | 0.642984 | -0.2799 | 0.448919 | 0.499495 | 0.966805 |
| 483 | 0.98361 | 0.571041 | -0.41257 | 0.438895 | 0.509653 | 0.92833 |
| 484 | 0.923216 | 0.665619 | -0.2576 | 0.450718 | 0.497736 | 0.973859 |
| 485 | 0.923216 | 0.665619 | -0.2576 | 0.450718 | 0.497736 | 0.973859 |
| 486 | 0.921714 | 0.564763 | -0.35695 | 0.442469 | 0.505802 | 0.941888 |
| 487 | 0.921714 | 0.564763 | -0.35695 | 0.442469 | 0.505802 | 0.941888 |
| 488 | 0.921714 | 0.564763 | -0.35695 | 0.442469 | 0.505802 | 0.941888 |
| 489 | 0.628247 | 0.569198 | -0.05905 | 0.464386 | 0.482578 | 1.029 |
| 490 | 0.98516 | 0.666199 | -0.31896 | 0.44659 | 0.502087 | 0.957741 |
| 491 | 0.960912 | 0.604384 | -0.35653 | 0.44319 | 0.505311 | 0.944645 |
| 492 | 0.889689 | 0.41941 | -0.47028 | 0.430813 | 0.51701 | 0.898296 |
| 493 | 0.98516 | 0.666199 | -0.31896 | 0.44659 | 0.502087 | 0.957741 |
| 494 | 0.939019 | 3.72666 | 2.78764 | 0.796057 | 0.159527 | 4.63257 |
| 495 | 0.937218 | 0.552927 | -0.38429 | 0.440382 | 0.507937 | 0.93395 |
| 496 | 0.8902 | 0.455904 | -0.4343 | 0.434536 | 0.51337 | 0.912026 |
| 497 | 0.92178 | 0.569216 | -0.35256 | 0.442876 | 0.505404 | 0.943443 |
| 498 | 0.923216 | 0.665619 | -0.2576 | 0.450718 | 0.497736 | 0.973859 |
| 499 | 0.92178 | 0.569216 | -0.35256 | 0.442876 | 0.505404 | 0.943443 |
| 500 | 1.0371 | 5.35989 | 4.3228 | 0.838125 | 0.117768 | 6.14492 |
| 501 | 0.98516 | 0.666199 | -0.31896 | 0.44659 | 0.502087 | 0.957741 |
| 502 | 0.98361 | 0.571041 | -0.41257 | 0.438895 | 0.509653 | 0.92833 |
| 503 | 0.960912 | 0.604384 | -0.35653 | 0.44319 | 0.505311 | 0.944645 |
| 504 | 0.921714 | 0.564763 | -0.35695 | 0.442469 | 0.505802 | 0.941888 |
| 505 | 0.98361 | 0.571041 | -0.41257 | 0.438895 | 0.509653 | 0.92833 |
| 506 | 0.890364 | 0.625168 | -0.2652 | 0.449682 | 0.498575 | 0.96979 |
| 507 | 0.923216 | 0.665619 | -0.2576 | 0.450718 | 0.497736 | 0.973859 |
| 508 | 0.936102 | 0.479328 | -0.45677 | 0.433744 | 0.514443 | 0.909089 |
| 509 | 0.98361 | 0.571041 | -0.41257 | 0.438895 | 0.509653 | 0.92833 |
| 510 | 0.626839 | 0.396367 | -0.23047 | 0.447425 | 0.498795 | 0.96098 |
| 511 | 0.98516 | 0.666199 | -0.31896 | 0.44659 | 0.502087 | 0.957741 |
| 512 | 0.923216 | 0.665619 | -0.2576 | 0.450718 | 0.497736 | 0.973859 |
| 513 | 0.793102 | 0.478555 | -0.31455 | 0.443494 | 0.503966 | 0.94581 |
| 514 | 0.628247 | 0.569198 | -0.05905 | 0.464386 | 0.482578 | 1.029 |
| 515 | 0.923216 | 0.665619 | -0.2576 | 0.450718 | 0.497736 | 0.973859 |
| 516 | 0.923216 | 0.665619 | -0.2576 | 0.450718 | 0.497736 | 0.973859 |
| 517 | 0.937188 | 0.550916 | -0.38627 | 0.44022 | 0.508096 | 0.933336 |
| 518 | 0.937218 | 0.552927 | -0.38429 | 0.440382 | 0.507937 | 0.93395 |
| 519 | 0.937188 | 0.550916 | -0.38627 | 0.44022 | 0.508096 | 0.933336 |
| 520 | 0.936102 | 0.479328 | -0.45677 | 0.433744 | 0.514443 | 0.909089 |
| 521 | 0.92178 | 0.569216 | -0.35256 | 0.442876 | 0.505404 | 0.943443 |
| 522 | 0.984237 | 0.609548 | -0.37469 | 0.442085 | 0.506516 | 0.940424 |
| 523 | 0.865573 | 0.644353 | -0.22122 | 0.45297 | 0.49523 | 0.982751 |
| 524 | 0.98361 | 0.571041 | -0.41257 | 0.438895 | 0.509653 | 0.92833 |
| 525 | 0.891537 | 0.550596 | -0.34094 | 0.443293 | 0.504818 | 0.94504 |
| 526 | 0.889935 | 0.437014 | -0.45292 | 0.432649 | 0.515214 | 0.905046 |
| 527 | 0.936102 | 0.479328 | -0.45677 | 0.433744 | 0.514443 | 0.909089 |
| 528 | 0.98361 | 0.571041 | -0.41257 | 0.438895 | 0.509653 | 0.92833 |
| 529 | 0.626839 | 0.396367 | -0.23047 | 0.447425 | 0.498795 | 0.96098 |
| 530 | 0.921434 | 0.545928 | -0.37551 | 0.440859 | 0.507377 | 0.93576 |
| 531 | 0.904336 | 0.609251 | -0.29509 | 0.447423 | 0.500858 | 0.960972 |
| 532 | 0.92178 | 0.569216 | -0.35256 | 0.442876 | 0.505404 | 0.943443 |
| 533 | 0.936102 | 0.479328 | -0.45677 | 0.433744 | 0.514443 | 0.909089 |
| 534 | 0.923216 | 0.665619 | -0.2576 | 0.450718 | 0.497736 | 0.973859 |
| 535 | 0.98516 | 0.666199 | -0.31896 | 0.44659 | 0.502087 | 0.957741 |
| 536 | 0.923216 | 0.665619 | -0.2576 | 0.450718 | 0.497736 | 0.973859 |
| 537 | 0.98516 | 0.666199 | -0.31896 | 0.44659 | 0.502087 | 0.957741 |
| 538 | 0.865573 | 0.644353 | -0.22122 | 0.45297 | 0.49523 | 0.982751 |
| 539 | 0.936102 | 0.479328 | -0.45677 | 0.433744 | 0.514443 | 0.909089 |
| 540 | 0.98516 | 0.666199 | -0.31896 | 0.44659 | 0.502087 | 0.957741 |
| 541 | 0.934182 | 0.351073 | -0.58311 | 0.419972 | 0.52796 | 0.859326 |
| 542 | 0.891537 | 0.550596 | -0.34094 | 0.443293 | 0.504818 | 0.94504 |
| 543 | 0.960912 | 0.604384 | -0.35653 | 0.44319 | 0.505311 | 0.944645 |
| 544 | 0.865573 | 0.644353 | -0.22122 | 0.45297 | 0.49523 | 0.982751 |
| 545 | 0.98516 | 0.666199 | -0.31896 | 0.44659 | 0.502087 | 0.957741 |
| 546 | 0.922879 | 0.642984 | -0.2799 | 0.448919 | 0.499495 | 0.966805 |
| 547 | 0.960912 | 0.604384 | -0.35653 | 0.44319 | 0.505311 | 0.944645 |
| 548 | 0.935467 | 0.437218 | -0.49825 | 0.429614 | 0.518493 | 0.893913 |
| 549 | 0.865573 | 0.644353 | -0.22122 | 0.45297 | 0.49523 | 0.982751 |
| 550 | 0.923216 | 0.665619 | -0.2576 | 0.450718 | 0.497736 | 0.973859 |
| 551 | 0.923216 | 0.665619 | -0.2576 | 0.450718 | 0.497736 | 0.973859 |
| 552 | 0.98516 | 0.666199 | -0.31896 | 0.44659 | 0.502087 | 0.957741 |
| 553 | 0.98516 | 0.666199 | -0.31896 | 0.44659 | 0.502087 | 0.957741 |
| 554 | 0.922879 | 0.642984 | -0.2799 | 0.448919 | 0.499495 | 0.966805 |
| 555 | 0.865573 | 0.644353 | -0.22122 | 0.45297 | 0.49523 | 0.982751 |
| 556 | 0.923216 | 0.665619 | -0.2576 | 0.450718 | 0.497736 | 0.973859 |
| 557 | 0.98516 | 0.666199 | -0.31896 | 0.44659 | 0.502087 | 0.957741 |
| 558 | 0.922879 | 0.642984 | -0.2799 | 0.448919 | 0.499495 | 0.966805 |
| 559 | 0.984237 | 0.609548 | -0.37469 | 0.442085 | 0.506516 | 0.940424 |
| 560 | 0.922879 | 0.642984 | -0.2799 | 0.448919 | 0.499495 | 0.966805 |
| 561 | 0.889935 | 0.437014 | -0.45292 | 0.432649 | 0.515214 | 0.905046 |
| 562 | 1.00702 | 0.508968 | -0.49805 | 0.431874 | 0.5167 | 0.902192 |
| 563 | 0.8902 | 0.455904 | -0.4343 | 0.434536 | 0.51337 | 0.912026 |
| 564 | 0.922879 | 0.642984 | -0.2799 | 0.448919 | 0.499495 | 0.966805 |
| 565 | 0.904336 | 0.609251 | -0.29509 | 0.447423 | 0.500858 | 0.960972 |
| 566 | 0.919919 | 0.443482 | -0.47644 | 0.431305 | 0.516731 | 0.900102 |
| 567 | 0.921714 | 0.564763 | -0.35695 | 0.442469 | 0.505802 | 0.941888 |
| 568 | 0.936102 | 0.479328 | -0.45677 | 0.433744 | 0.514443 | 0.909089 |
| 569 | 0.921434 | 0.545928 | -0.37551 | 0.440859 | 0.507377 | 0.93576 |
| 570 | 0.923216 | 0.665619 | -0.2576 | 0.450718 | 0.497736 | 0.973859 |
| 571 | 0.923216 | 0.665619 | -0.2576 | 0.450718 | 0.497736 | 0.973859 |
| 572 | 0.98516 | 0.666199 | -0.31896 | 0.44659 | 0.502087 | 0.957741 |
| 573 | 0.923216 | 0.665619 | -0.2576 | 0.450718 | 0.497736 | 0.973859 |
| 574 | 0.923216 | 0.665619 | -0.2576 | 0.450718 | 0.497736 | 0.973859 |
| 575 | 0.98516 | 0.666199 | -0.31896 | 0.44659 | 0.502087 | 0.957741 |
| 576 | 0.98516 | 0.666199 | -0.31896 | 0.44659 | 0.502087 | 0.957741 |
| 577 | 0.92178 | 0.569216 | -0.35256 | 0.442876 | 0.505404 | 0.943443 |
| 578 | 0.628247 | 0.569198 | -0.05905 | 0.464386 | 0.482578 | 1.029 |
| 579 | 1.00702 | 0.508968 | -0.49805 | 0.431874 | 0.5167 | 0.902192 |
| 580 | 0.921434 | 0.545928 | -0.37551 | 0.440859 | 0.507377 | 0.93576 |
| 581 | 0.670282 | 0.536524 | -0.13376 | 0.458115 | 0.488921 | 1.00335 |
| 582 | 0.922109 | 0.591355 | -0.33075 | 0.444735 | 0.503586 | 0.950576 |
| 583 | 0.960912 | 0.604384 | -0.35653 | 0.44319 | 0.505311 | 0.944645 |
| 584 | 0.98516 | 0.666199 | -0.31896 | 0.44659 | 0.502087 | 0.957741 |
| 585 | 0.888741 | 0.350946 | -0.5378 | 0.422967 | 0.524691 | 0.869945 |
| 586 | 1.00702 | 0.508968 | -0.49805 | 0.431874 | 0.5167 | 0.902192 |
| 587 | 0.889689 | 0.41941 | -0.47028 | 0.430813 | 0.51701 | 0.898296 |
| 588 | 0.922879 | 0.642984 | -0.2799 | 0.448919 | 0.499495 | 0.966805 |
| 589 | 0.936102 | 0.479328 | -0.45677 | 0.433744 | 0.514443 | 0.909089 |
| 590 | 0.984237 | 0.609548 | -0.37469 | 0.442085 | 0.506516 | 0.940424 |
| 591 | 0.923216 | 0.665619 | -0.2576 | 0.450718 | 0.497736 | 0.973859 |
| 592 | 0.922109 | 0.591355 | -0.33075 | 0.444735 | 0.503586 | 0.950576 |
| 593 | 0.98516 | 0.666199 | -0.31896 | 0.44659 | 0.502087 | 0.957741 |
| 594 | 1.00275 | 5.78243 | 4.77969 | 0.853044 | 0.104487 | 6.8892 |
| 595 | 0.960912 | 0.604384 | -0.35653 | 0.44319 | 0.505311 | 0.944645 |
| 596 | 0.92178 | 0.569216 | -0.35256 | 0.442876 | 0.505404 | 0.943443 |
| 597 | 0.922109 | 0.591355 | -0.33075 | 0.444735 | 0.503586 | 0.950576 |
| 598 | 1.00702 | 0.508968 | -0.49805 | 0.431874 | 0.5167 | 0.902192 |
| 599 | 0.960912 | 0.604384 | -0.35653 | 0.44319 | 0.505311 | 0.944645 |
| 600 | 0.923216 | 0.665619 | -0.2576 | 0.450718 | 0.497736 | 0.973859 |
| 601 | 0.944482 | 0.588393 | -0.35609 | 0.442967 | 0.505442 | 0.943793 |
| 602 | 0.865573 | 0.644353 | -0.22122 | 0.45297 | 0.49523 | 0.982751 |
| 603 | 0.934182 | 0.351073 | -0.58311 | 0.419972 | 0.52796 | 0.859326 |
| 604 | 0.923216 | 0.665619 | -0.2576 | 0.450718 | 0.497736 | 0.973859 |
| 605 | 0.937188 | 0.550916 | -0.38627 | 0.44022 | 0.508096 | 0.933336 |
| 606 | 0.922879 | 0.642984 | -0.2799 | 0.448919 | 0.499495 | 0.966805 |
| 607 | 0.960912 | 0.604384 | -0.35653 | 0.44319 | 0.505311 | 0.944645 |
| 608 | 0.865573 | 0.644353 | -0.22122 | 0.45297 | 0.49523 | 0.982751 |
| 609 | 0.923216 | 0.665619 | -0.2576 | 0.450718 | 0.497736 | 0.973859 |
| 610 | 0.936102 | 0.479328 | -0.45677 | 0.433744 | 0.514443 | 0.909089 |
| 611 | 0.960912 | 0.604384 | -0.35653 | 0.44319 | 0.505311 | 0.944645 |
| 612 | 0.984237 | 0.609548 | -0.37469 | 0.442085 | 0.506516 | 0.940424 |
| 613 | 0.922879 | 0.642984 | -0.2799 | 0.448919 | 0.499495 | 0.966805 |
| 614 | 0.904336 | 0.609251 | -0.29509 | 0.447423 | 0.500858 | 0.960972 |
| 615 | 0.865573 | 0.644353 | -0.22122 | 0.45297 | 0.49523 | 0.982751 |
| 616 | 0.936102 | 0.479328 | -0.45677 | 0.433744 | 0.514443 | 0.909089 |
| 617 | 0.627774 | 0.51183 | -0.11594 | 0.459232 | 0.487493 | 1.00788 |
| 618 | 0.888741 | 0.350946 | -0.5378 | 0.422967 | 0.524691 | 0.869945 |
| 619 | 0.98516 | 0.666199 | -0.31896 | 0.44659 | 0.502087 | 0.957741 |
| 620 | 0.936102 | 0.479328 | -0.45677 | 0.433744 | 0.514443 | 0.909089 |
| 621 | 0.92178 | 0.569216 | -0.35256 | 0.442876 | 0.505404 | 0.943443 |
| 622 | 0.92178 | 0.569216 | -0.35256 | 0.442876 | 0.505404 | 0.943443 |
| 623 | 0.793603 | 0.520671 | -0.27293 | 0.447416 | 0.500167 | 0.960948 |
| 624 | 0.922879 | 0.642984 | -0.2799 | 0.448919 | 0.499495 | 0.966805 |
| 625 | 0.98516 | 0.666199 | -0.31896 | 0.44659 | 0.502087 | 0.957741 |
| 626 | 0.937218 | 0.552927 | -0.38429 | 0.440382 | 0.507937 | 0.93395 |
| 627 | 0.865573 | 0.644353 | -0.22122 | 0.45297 | 0.49523 | 0.982751 |
| 628 | 0.904336 | 0.609251 | -0.29509 | 0.447423 | 0.500858 | 0.960972 |
| 629 | 0.98516 | 0.666199 | -0.31896 | 0.44659 | 0.502087 | 0.957741 |
| 630 | 0.923216 | 0.665619 | -0.2576 | 0.450718 | 0.497736 | 0.973859 |
| 631 | 0.923216 | 0.665619 | -0.2576 | 0.450718 | 0.497736 | 0.973859 |
| 632 | 0.960912 | 0.604384 | -0.35653 | 0.44319 | 0.505311 | 0.944645 |
| 633 | 0.98516 | 0.666199 | -0.31896 | 0.44659 | 0.502087 | 0.957741 |
| 634 | 0.923216 | 0.665619 | -0.2576 | 0.450718 | 0.497736 | 0.973859 |
| 635 | 0.919919 | 0.443482 | -0.47644 | 0.431305 | 0.516731 | 0.900102 |
| 636 | 0.919919 | 0.443482 | -0.47644 | 0.431305 | 0.516731 | 0.900102 |
| 637 | 0.865573 | 0.644353 | -0.22122 | 0.45297 | 0.49523 | 0.982751 |
| 638 | 0.936102 | 0.479328 | -0.45677 | 0.433744 | 0.514443 | 0.909089 |
| 639 | 0.937188 | 0.550916 | -0.38627 | 0.44022 | 0.508096 | 0.933336 |
| 640 | 0.936102 | 0.479328 | -0.45677 | 0.433744 | 0.514443 | 0.909089 |
| 641 | 0.922109 | 0.591355 | -0.33075 | 0.444735 | 0.503586 | 0.950576 |
| 642 | 0.904336 | 0.609251 | -0.29509 | 0.447423 | 0.500858 | 0.960972 |
| 643 | 0.937188 | 0.550916 | -0.38627 | 0.44022 | 0.508096 | 0.933336 |
| 644 | 0.865573 | 0.644353 | -0.22122 | 0.45297 | 0.49523 | 0.982751 |
| 645 | 0.960912 | 0.604384 | -0.35653 | 0.44319 | 0.505311 | 0.944645 |
| 646 | 0.889689 | 0.41941 | -0.47028 | 0.430813 | 0.51701 | 0.898296 |
| 647 | 0.937218 | 0.552927 | -0.38429 | 0.440382 | 0.507937 | 0.93395 |
| 648 | 0.923216 | 0.665619 | -0.2576 | 0.450718 | 0.497736 | 0.973859 |
| 649 | 0.923216 | 0.665619 | -0.2576 | 0.450718 | 0.497736 | 0.973859 |
| 650 | 0.922879 | 0.642984 | -0.2799 | 0.448919 | 0.499495 | 0.966805 |
| 651 | 0.921714 | 0.564763 | -0.35695 | 0.442469 | 0.505802 | 0.941888 |
| 652 | 0.98516 | 0.666199 | -0.31896 | 0.44659 | 0.502087 | 0.957741 |
| 653 | 0.923216 | 0.665619 | -0.2576 | 0.450718 | 0.497736 | 0.973859 |
| 654 | 0.960912 | 0.604384 | -0.35653 | 0.44319 | 0.505311 | 0.944645 |
| 655 | 0.628247 | 0.569198 | -0.05905 | 0.464386 | 0.482578 | 1.029 |
| 656 | 0.8902 | 0.455904 | -0.4343 | 0.434536 | 0.51337 | 0.912026 |
| 657 | 0.904336 | 0.609251 | -0.29509 | 0.447423 | 0.500858 | 0.960972 |
| 658 | 0.786362 | 0.596037 | -0.19033 | 0.454599 | 0.493162 | 0.989233 |
| 659 | 0.937188 | 0.550916 | -0.38627 | 0.44022 | 0.508096 | 0.933336 |
| 660 | 0.923216 | 0.665619 | -0.2576 | 0.450718 | 0.497736 | 0.973859 |
| 661 | 0.626839 | 0.396367 | -0.23047 | 0.447425 | 0.498795 | 0.96098 |
| 662 | 0.628835 | 0.64031 | 0.011475 | 0.470349 | 0.476905 | 1.05394 |
| 663 | 0.904336 | 0.609251 | -0.29509 | 0.447423 | 0.500858 | 0.960972 |
| 664 | 1.08995 | 8.77097 | 7.68102 | 0.932169 | 0.027076 | 16.3101 |
| 665 | 0.921714 | 0.564763 | -0.35695 | 0.442469 | 0.505802 | 0.941888 |
| 666 | 0.889689 | 0.41941 | -0.47028 | 0.430813 | 0.51701 | 0.898296 |
| 667 | 0.628247 | 0.569198 | -0.05905 | 0.464386 | 0.482578 | 1.029 |
| 668 | 0.890526 | 0.479082 | -0.41144 | 0.436795 | 0.511162 | 0.920443 |
| 669 | 0.98516 | 0.666199 | -0.31896 | 0.44659 | 0.502087 | 0.957741 |
| 670 | 0.923216 | 0.665619 | -0.2576 | 0.450718 | 0.497736 | 0.973859 |
| 671 | 0.984237 | 0.609548 | -0.37469 | 0.442085 | 0.506516 | 0.940424 |
| 672 | 0.936102 | 0.479328 | -0.45677 | 0.433744 | 0.514443 | 0.909089 |
| 673 | 0.922879 | 0.642984 | -0.2799 | 0.448919 | 0.499495 | 0.966805 |
| 674 | 0.786362 | 0.596037 | -0.19033 | 0.454599 | 0.493162 | 0.989233 |
| 675 | 0.865573 | 0.644353 | -0.22122 | 0.45297 | 0.49523 | 0.982751 |
| 676 | 0.98516 | 0.666199 | -0.31896 | 0.44659 | 0.502087 | 0.957741 |
| 677 | 0.922879 | 0.642984 | -0.2799 | 0.448919 | 0.499495 | 0.966805 |
| 678 | 0.922879 | 0.642984 | -0.2799 | 0.448919 | 0.499495 | 0.966805 |
| 679 | 0.865573 | 0.644353 | -0.22122 | 0.45297 | 0.49523 | 0.982751 |
| 680 | 0.937218 | 0.552927 | -0.38429 | 0.440382 | 0.507937 | 0.93395 |
| 681 | 0.935467 | 0.437218 | -0.49825 | 0.429614 | 0.518493 | 0.893913 |
| 682 | 0.865573 | 0.644353 | -0.22122 | 0.45297 | 0.49523 | 0.982751 |
| 683 | 0.960912 | 0.604384 | -0.35653 | 0.44319 | 0.505311 | 0.944645 |
| 684 | 0.937218 | 0.552927 | -0.38429 | 0.440382 | 0.507937 | 0.93395 |
| 685 | 0.921714 | 0.564763 | -0.35695 | 0.442469 | 0.505802 | 0.941888 |
| 686 | 0.891537 | 0.550596 | -0.34094 | 0.443293 | 0.504818 | 0.94504 |
| 687 | 0.888741 | 0.350946 | -0.5378 | 0.422967 | 0.524691 | 0.869945 |
| 688 | 0.921714 | 0.564763 | -0.35695 | 0.442469 | 0.505802 | 0.941888 |
| 689 | 0.555143 | 0.586251 | 0.031108 | 0.472036 | 0.474659 | 1.0611 |
| 690 | 0.98516 | 0.666199 | -0.31896 | 0.44659 | 0.502087 | 0.957741 |
| 691 | 0.934182 | 0.351073 | -0.58311 | 0.419972 | 0.52796 | 0.859326 |
| 692 | 0.935467 | 0.437218 | -0.49825 | 0.429614 | 0.518493 | 0.893913 |
| 693 | 1.00702 | 0.508968 | -0.49805 | 0.431874 | 0.5167 | 0.902192 |
| 694 | 0.98516 | 0.666199 | -0.31896 | 0.44659 | 0.502087 | 0.957741 |
| 695 | 0.960912 | 0.604384 | -0.35653 | 0.44319 | 0.505311 | 0.944645 |
| 696 | 0.922879 | 0.642984 | -0.2799 | 0.448919 | 0.499495 | 0.966805 |
| 697 | 0.922879 | 0.642984 | -0.2799 | 0.448919 | 0.499495 | 0.966805 |
| 698 | 0.627774 | 0.51183 | -0.11594 | 0.459232 | 0.487493 | 1.00788 |
| 699 | 0.98361 | 0.571041 | -0.41257 | 0.438895 | 0.509653 | 0.92833 |
| 700 | 0.889474 | 0.403981 | -0.48549 | 0.429167 | 0.51862 | 0.892285 |
| 701 | 0.922879 | 0.642984 | -0.2799 | 0.448919 | 0.499495 | 0.966805 |
| 702 | 0.960912 | 0.604384 | -0.35653 | 0.44319 | 0.505311 | 0.944645 |
| 703 | 0.923216 | 0.665619 | -0.2576 | 0.450718 | 0.497736 | 0.973859 |
| 704 | 0.865573 | 0.644353 | -0.22122 | 0.45297 | 0.49523 | 0.982751 |
| 705 | 0.92178 | 0.569216 | -0.35256 | 0.442876 | 0.505404 | 0.943443 |
| 706 | 0.889689 | 0.41941 | -0.47028 | 0.430813 | 0.51701 | 0.898296 |
| 707 | 0.922879 | 0.642984 | -0.2799 | 0.448919 | 0.499495 | 0.966805 |
| 708 | 0.936102 | 0.479328 | -0.45677 | 0.433744 | 0.514443 | 0.909089 |
| 709 | 0.865573 | 0.644353 | -0.22122 | 0.45297 | 0.49523 | 0.982751 |
| 710 | 0.923216 | 0.665619 | -0.2576 | 0.450718 | 0.497736 | 0.973859 |
| 711 | 0.865573 | 0.644353 | -0.22122 | 0.45297 | 0.49523 | 0.982751 |
| 712 | 0.88964 | 0.57404 | -0.3156 | 0.445448 | 0.502703 | 0.953324 |
| 713 | 0.960912 | 0.604384 | -0.35653 | 0.44319 | 0.505311 | 0.944645 |
| 714 | 0.532654 | 0.435436 | -0.09722 | 0.459719 | 0.486111 | 1.00985 |
| 715 | 0.98516 | 0.666199 | -0.31896 | 0.44659 | 0.502087 | 0.957741 |
| 716 | 0.937188 | 0.550916 | -0.38627 | 0.44022 | 0.508096 | 0.933336 |
| 717 | 0.921714 | 0.564763 | -0.35695 | 0.442469 | 0.505802 | 0.941888 |
| 718 | 0.923216 | 0.665619 | -0.2576 | 0.450718 | 0.497736 | 0.973859 |
| 719 | 0.9371 | 0.545132 | -0.39197 | 0.439732 | 0.508574 | 0.93149 |
| 720 | 0.865573 | 0.644353 | -0.22122 | 0.45297 | 0.49523 | 0.982751 |
| 721 | 0.670282 | 0.536524 | -0.13376 | 0.458115 | 0.488921 | 1.00335 |
| 722 | 0.700257 | 5.2534 | 4.55314 | 0.866541 | 0.098717 | 7.70599 |
| 723 | 0.937188 | 0.550916 | -0.38627 | 0.44022 | 0.508096 | 0.933336 |
| 724 | 0.904336 | 0.609251 | -0.29509 | 0.447423 | 0.500858 | 0.960972 |
| 725 | 0.670282 | 0.536524 | -0.13376 | 0.458115 | 0.488921 | 1.00335 |
| 726 | 0.98516 | 0.666199 | -0.31896 | 0.44659 | 0.502087 | 0.957741 |
| 727 | 1.00797 | 0.565383 | -0.44259 | 0.436808 | 0.511837 | 0.920492 |
| 728 | 0.937218 | 0.552927 | -0.38429 | 0.440382 | 0.507937 | 0.93395 |
| 729 | 0.98361 | 0.571041 | -0.41257 | 0.438895 | 0.509653 | 0.92833 |
| 730 | 0.935467 | 0.437218 | -0.49825 | 0.429614 | 0.518493 | 0.893913 |
| 731 | 0.627605 | 0.491184 | -0.13642 | 0.457261 | 0.489376 | 0.999905 |
| 732 | 0.98516 | 0.666199 | -0.31896 | 0.44659 | 0.502087 | 0.957741 |
| 733 | 0.923216 | 0.665619 | -0.2576 | 0.450718 | 0.497736 | 0.973859 |
| 734 | 0.984237 | 0.609548 | -0.37469 | 0.442085 | 0.506516 | 0.940424 |
| 735 | 0.923216 | 0.665619 | -0.2576 | 0.450718 | 0.497736 | 0.973859 |
| 736 | 0.800646 | 8.81034 | 8.0097 | 0.955246 | 0.017339 | 25.3318 |
| 737 | 0.977396 | 4.16183 | 3.18444 | 0.807064 | 0.148501 | 4.96457 |
| 738 | 0.960912 | 0.604384 | -0.35653 | 0.44319 | 0.505311 | 0.944645 |
| 739 | 0.936102 | 0.479328 | -0.45677 | 0.433744 | 0.514443 | 0.909089 |
| 740 | 0.923216 | 0.665619 | -0.2576 | 0.450718 | 0.497736 | 0.973859 |
| 741 | 0.98361 | 0.571041 | -0.41257 | 0.438895 | 0.509653 | 0.92833 |
| 742 | 0.960912 | 0.604384 | -0.35653 | 0.44319 | 0.505311 | 0.944645 |
| 743 | 0.533342 | 0.548109 | 0.014767 | 0.470618 | 0.475786 | 1.05508 |
| 744 | 0.904336 | 0.609251 | -0.29509 | 0.447423 | 0.500858 | 0.960972 |
| 745 | 0.98361 | 0.571041 | -0.41257 | 0.438895 | 0.509653 | 0.92833 |
| 746 | 1.00702 | 0.508968 | -0.49805 | 0.431874 | 0.5167 | 0.902192 |
| 747 | 0.628835 | 0.64031 | 0.011475 | 0.470349 | 0.476905 | 1.05394 |
| 748 | 0.98516 | 0.666199 | -0.31896 | 0.44659 | 0.502087 | 0.957741 |
| 749 | 0.923216 | 0.665619 | -0.2576 | 0.450718 | 0.497736 | 0.973859 |
| 750 | 0.889689 | 0.41941 | -0.47028 | 0.430813 | 0.51701 | 0.898296 |
| 751 | 0.8902 | 0.455904 | -0.4343 | 0.434536 | 0.51337 | 0.912026 |
| 752 | 0.532767 | 0.454176 | -0.07859 | 0.461655 | 0.484272 | 1.01776 |
| 753 | 0.921714 | 0.564763 | -0.35695 | 0.442469 | 0.505802 | 0.941888 |
| 754 | 0.8902 | 0.455904 | -0.4343 | 0.434536 | 0.51337 | 0.912026 |
| 755 | 0.919919 | 0.443482 | -0.47644 | 0.431305 | 0.516731 | 0.900102 |
| 756 | 0.628247 | 0.569198 | -0.05905 | 0.464386 | 0.482578 | 1.029 |
| 757 | 0.944482 | 0.588393 | -0.35609 | 0.442967 | 0.505442 | 0.943793 |
| 758 | 0.904336 | 0.609251 | -0.29509 | 0.447423 | 0.500858 | 0.960972 |
| 759 | 0.934182 | 0.351073 | -0.58311 | 0.419972 | 0.52796 | 0.859326 |
| 760 | 0.923216 | 0.665619 | -0.2576 | 0.450718 | 0.497736 | 0.973859 |
| 761 | 0.670282 | 0.536524 | -0.13376 | 0.458115 | 0.488921 | 1.00335 |
| 762 | 0.9371 | 0.545132 | -0.39197 | 0.439732 | 0.508574 | 0.93149 |
| 763 | 0.919919 | 0.443482 | -0.47644 | 0.431305 | 0.516731 | 0.900102 |
| 764 | 1.00702 | 0.508968 | -0.49805 | 0.431874 | 0.5167 | 0.902192 |
| 765 | 0.627605 | 0.491184 | -0.13642 | 0.457261 | 0.489376 | 0.999905 |
| 766 | 0.98516 | 0.666199 | -0.31896 | 0.44659 | 0.502087 | 0.957741 |
| 767 | 0.8902 | 0.455904 | -0.4343 | 0.434536 | 0.51337 | 0.912026 |
| 768 | 0.670282 | 0.536524 | -0.13376 | 0.458115 | 0.488921 | 1.00335 |
| 769 | 0.984237 | 0.609548 | -0.37469 | 0.442085 | 0.506516 | 0.940424 |
| 770 | 0.937188 | 0.550916 | -0.38627 | 0.44022 | 0.508096 | 0.933336 |
| 771 | 0.904336 | 0.609251 | -0.29509 | 0.447423 | 0.500858 | 0.960972 |
| 772 | 0.865573 | 0.644353 | -0.22122 | 0.45297 | 0.49523 | 0.982751 |
| 773 | 0.889474 | 0.403981 | -0.48549 | 0.429167 | 0.51862 | 0.892285 |
| 774 | 0.922879 | 0.642984 | -0.2799 | 0.448919 | 0.499495 | 0.966805 |
| 775 | 0.98361 | 0.571041 | -0.41257 | 0.438895 | 0.509653 | 0.92833 |
| 776 | 0.944482 | 0.588393 | -0.35609 | 0.442967 | 0.505442 | 0.943793 |
| 777 | 0.960912 | 0.604384 | -0.35653 | 0.44319 | 0.505311 | 0.944645 |
| 778 | 0.922879 | 0.642984 | -0.2799 | 0.448919 | 0.499495 | 0.966805 |
| 779 | 0.923216 | 0.665619 | -0.2576 | 0.450718 | 0.497736 | 0.973859 |
| 780 | 0.922109 | 0.591355 | -0.33075 | 0.444735 | 0.503586 | 0.950576 |
| 781 | 0.960912 | 0.604384 | -0.35653 | 0.44319 | 0.505311 | 0.944645 |
| 782 | 0.98516 | 0.666199 | -0.31896 | 0.44659 | 0.502087 | 0.957741 |
| 783 | 0.890364 | 0.625168 | -0.2652 | 0.449682 | 0.498575 | 0.96979 |
| 784 | 0.891169 | 0.524596 | -0.36657 | 0.441013 | 0.507042 | 0.936346 |
| 785 | 0.890364 | 0.625168 | -0.2652 | 0.449682 | 0.498575 | 0.96979 |
| 786 | 0.88964 | 0.57404 | -0.3156 | 0.445448 | 0.502703 | 0.953324 |
| 787 | 0.922109 | 0.591355 | -0.33075 | 0.444735 | 0.503586 | 0.950576 |
| 788 | 0.984237 | 0.609548 | -0.37469 | 0.442085 | 0.506516 | 0.940424 |
| 789 | 0.922879 | 0.642984 | -0.2799 | 0.448919 | 0.499495 | 0.966805 |
| 790 | 0.984237 | 0.609548 | -0.37469 | 0.442085 | 0.506516 | 0.940424 |
| 791 | 0.891537 | 0.550596 | -0.34094 | 0.443293 | 0.504818 | 0.94504 |
| 792 | 1.00702 | 0.508968 | -0.49805 | 0.431874 | 0.5167 | 0.902192 |
| 793 | 0.960912 | 0.604384 | -0.35653 | 0.44319 | 0.505311 | 0.944645 |
| 794 | 0.891169 | 0.524596 | -0.36657 | 0.441013 | 0.507042 | 0.936346 |
| 795 | 0.922879 | 0.642984 | -0.2799 | 0.448919 | 0.499495 | 0.966805 |
| 796 | 0.921714 | 0.564763 | -0.35695 | 0.442469 | 0.505802 | 0.941888 |
| 797 | 0.936102 | 0.479328 | -0.45677 | 0.433744 | 0.514443 | 0.909089 |
| 798 | 0.98516 | 0.666199 | -0.31896 | 0.44659 | 0.502087 | 0.957741 |
| 799 | 0.922879 | 0.642984 | -0.2799 | 0.448919 | 0.499495 | 0.966805 |
| 800 | 0.793952 | 0.549915 | -0.24404 | 0.450038 | 0.497628 | 0.971188 |
| 801 | 0.944482 | 0.588393 | -0.35609 | 0.442967 | 0.505442 | 0.943793 |
| 802 | 0.923216 | 0.665619 | -0.2576 | 0.450718 | 0.497736 | 0.973859 |
| 803 | 0.937188 | 0.550916 | -0.38627 | 0.44022 | 0.508096 | 0.933336 |
| 804 | 0.922879 | 0.642984 | -0.2799 | 0.448919 | 0.499495 | 0.966805 |
| 805 | 0.889935 | 0.437014 | -0.45292 | 0.432649 | 0.515214 | 0.905046 |
| 806 | 0.98516 | 0.666199 | -0.31896 | 0.44659 | 0.502087 | 0.957741 |
| 807 | 0.936102 | 0.479328 | -0.45677 | 0.433744 | 0.514443 | 0.909089 |
| 808 | 0.904336 | 0.609251 | -0.29509 | 0.447423 | 0.500858 | 0.960972 |
| 809 | 0.936102 | 0.479328 | -0.45677 | 0.433744 | 0.514443 | 0.909089 |
| 810 | 0.8902 | 0.455904 | -0.4343 | 0.434536 | 0.51337 | 0.912026 |
| PF3D7_1335900 | | | | | | |
| Site | **alpha** | **beta** | **beta-alpha** | **Prob[alpha>beta]** | **Prob[alpha<&beta]** | **BayesFactor[alpha<beta;]** |
| 1 | 1.933 | 0.349 | -1.584 | 0.637 | 0.293 | 0.596 |
| 2 | 1.151 | 0.406 | -0.745 | 0.598 | 0.33 | 0.708 |
| 3 | 1.127 | 0.349 | -0.779 | 0.621 | 0.306 | 0.634 |
| 4 | 0.696 | 0.35 | -0.346 | 0.603 | 0.321 | 0.681 |
| 5 | 0.503 | 0.337 | -0.166 | 0.581 | 0.338 | 0.736 |
| 6 | 1.151 | 0.406 | -0.745 | 0.598 | 0.33 | 0.708 |
| 7 | 0.701 | 0.377 | -0.324 | 0.591 | 0.334 | 0.722 |
| 8 | 1.228 | 0.42 | -0.808 | 0.596 | 0.332 | 0.716 |
| 9 | 1.16 | 0.441 | -0.72 | 0.59 | 0.339 | 0.738 |
| 10 | 0.697 | 0.478 | -0.219 | 0.564 | 0.362 | 0.818 |
| 11 | 0.496 | 0.372 | -0.123 | 0.56 | 0.36 | 0.808 |
| 12 | 0.741 | 0.408 | -0.333 | 0.583 | 0.343 | 0.753 |
| 13 | 0.506 | 0.373 | -0.133 | 0.563 | 0.357 | 0.799 |
| 14 | 1.148 | 0.397 | -0.751 | 0.601 | 0.327 | 0.699 |
| 15 | 0.528 | 0.417 | -0.111 | 0.554 | 0.368 | 0.839 |
| 16 | 0.741 | 0.408 | -0.333 | 0.583 | 0.343 | 0.753 |
| 17 | 0.58 | 0.369 | -0.211 | 0.581 | 0.342 | 0.747 |
| 18 | 1.148 | 0.397 | -0.751 | 0.601 | 0.327 | 0.699 |
| 19 | 1.139 | 0.374 | -0.765 | 0.609 | 0.318 | 0.671 |
| 20 | 0.528 | 0.417 | -0.111 | 0.554 | 0.368 | 0.839 |
| 21 | 1.148 | 0.397 | -0.751 | 0.601 | 0.327 | 0.699 |
| 22 | 0.569 | 0.367 | -0.202 | 0.58 | 0.342 | 0.749 |
| 23 | 0.701 | 0.377 | -0.324 | 0.591 | 0.334 | 0.722 |
| 24 | 1.151 | 0.406 | -0.745 | 0.598 | 0.33 | 0.708 |
| 25 | 0.695 | 0.341 | -0.354 | 0.608 | 0.316 | 0.665 |
| 26 | 0.865 | 0.362 | -0.503 | 0.606 | 0.32 | 0.677 |
| 27 | 1.139 | 0.374 | -0.765 | 0.609 | 0.318 | 0.671 |
| 28 | 0.506 | 0.373 | -0.133 | 0.563 | 0.357 | 0.799 |
| 29 | 1.208 | 0.37 | -0.838 | 0.613 | 0.315 | 0.661 |
| 30 | 0.58 | 0.375 | -0.205 | 0.579 | 0.344 | 0.756 |
| 31 | 1.151 | 0.406 | -0.745 | 0.598 | 0.33 | 0.708 |
| 32 | 0.883 | 0.404 | -0.479 | 0.591 | 0.336 | 0.729 |
| 33 | 0.506 | 0.373 | -0.133 | 0.563 | 0.357 | 0.799 |
| 34 | 1.139 | 0.374 | -0.765 | 0.609 | 0.318 | 0.671 |
| 35 | 1.215 | 0.386 | -0.83 | 0.607 | 0.321 | 0.681 |
| 36 | 0.883 | 0.404 | -0.479 | 0.591 | 0.336 | 0.729 |
| 37 | 1.228 | 0.42 | -0.808 | 0.596 | 0.332 | 0.716 |
| 38 | 1.16 | 0.441 | -0.72 | 0.59 | 0.339 | 0.738 |
| 39 | 0.92 | 10.365 | 9.446 | 0.007 | 0.976 | 58.77 |
| 40 | 1.215 | 0.386 | -0.83 | 0.607 | 0.321 | 0.681 |
| 41 | 1.215 | 0.386 | -0.83 | 0.607 | 0.321 | 0.681 |
| 42 | 0.745 | 0.377 | -0.367 | 0.593 | 0.332 | 0.715 |
| 43 | 1.135 | 0.364 | -0.771 | 0.613 | 0.314 | 0.658 |
| 44 | 1.419 | 1.272 | -0.147 | 0.345 | 0.598 | 2.14 |
| 45 | 1.139 | 0.374 | -0.765 | 0.609 | 0.318 | 0.671 |
| 46 | 0.697 | 16.563 | 15.866 | 0 | 0.997 | 449.46 |
| 47 | 0.745 | 0.377 | -0.367 | 0.593 | 0.332 | 0.715 |
| 48 | 1.139 | 0.374 | -0.765 | 0.609 | 0.318 | 0.671 |
| 49 | 0.79 | 0.832 | 0.042 | 0.357 | 0.582 | 2.007 |
| 50 | 0.584 | 0.433 | -0.151 | 0.561 | 0.363 | 0.821 |
| 51 | 0.696 | 0.35 | -0.346 | 0.603 | 0.321 | 0.681 |
| 52 | 0.569 | 0.367 | -0.202 | 0.58 | 0.342 | 0.749 |
| 53 | 1.933 | 0.349 | -1.584 | 0.637 | 0.293 | 0.596 |
| 54 | 1.139 | 0.374 | -0.765 | 0.609 | 0.318 | 0.671 |
| 55 | 1.135 | 0.364 | -0.771 | 0.613 | 0.314 | 0.658 |
| 56 | 0.7 | 0.368 | -0.332 | 0.594 | 0.33 | 0.71 |
| 57 | 0.739 | 0.347 | -0.392 | 0.607 | 0.317 | 0.668 |
| 58 | 1.129 | 0.352 | -0.777 | 0.619 | 0.308 | 0.639 |
| 59 | 0.883 | 0.404 | -0.479 | 0.591 | 0.336 | 0.729 |
| 60 | 0.691 | 0.324 | -0.367 | 0.618 | 0.305 | 0.633 |
| 61 | 0.69 | 0.323 | -0.368 | 0.619 | 0.304 | 0.63 |
| 62 | 1.127 | 0.349 | -0.779 | 0.621 | 0.306 | 0.634 |
| 63 | 1.151 | 0.406 | -0.745 | 0.598 | 0.33 | 0.708 |
| 64 | 1.967 | 0.382 | -1.585 | 0.623 | 0.307 | 0.639 |
| 65 | 0.506 | 0.373 | -0.133 | 0.563 | 0.357 | 0.799 |
| 66 | 0.727 | 20.276 | 19.549 | 0 | 0.998 | 671.474 |
| 67 | 1.127 | 0.349 | -0.779 | 0.621 | 0.306 | 0.634 |
| 68 | 0.738 | 0.345 | -0.393 | 0.608 | 0.316 | 0.665 |
| 69 | 0.745 | 0.377 | -0.367 | 0.593 | 0.332 | 0.715 |
| 70 | 0.691 | 0.326 | -0.366 | 0.617 | 0.306 | 0.636 |
| 71 | 0.569 | 0.367 | -0.202 | 0.58 | 0.342 | 0.749 |
| 72 | 0.695 | 0.345 | -0.351 | 0.606 | 0.318 | 0.672 |
| 73 | 1.933 | 0.349 | -1.584 | 0.637 | 0.293 | 0.596 |
| 74 | 1.228 | 0.42 | -0.808 | 0.596 | 0.332 | 0.716 |
| 75 | 0.528 | 0.417 | -0.111 | 0.554 | 0.368 | 0.839 |
| 76 | 0.883 | 0.404 | -0.479 | 0.591 | 0.336 | 0.729 |
| 77 | 1.208 | 0.37 | -0.838 | 0.613 | 0.315 | 0.661 |
| 78 | 1.208 | 0.37 | -0.838 | 0.613 | 0.315 | 0.661 |
| 79 | 0.697 | 0.478 | -0.219 | 0.564 | 0.362 | 0.818 |
| 80 | 1.151 | 0.406 | -0.745 | 0.598 | 0.33 | 0.708 |
| 81 | 0.696 | 0.35 | -0.346 | 0.603 | 0.321 | 0.681 |
| 82 | 1.151 | 0.406 | -0.745 | 0.598 | 0.33 | 0.708 |
| 83 | 2.481 | 25.174 | 22.693 | 0.016 | 0.962 | 36.348 |
| 84 | 1.692 | 8.466 | 6.774 | 0.052 | 0.916 | 15.662 |
| 85 | 0.846 | 0.805 | -0.042 | 0.365 | 0.574 | 1.939 |
| 86 | 0.741 | 0.408 | -0.333 | 0.583 | 0.343 | 0.753 |
| 87 | 0.575 | 0.331 | -0.244 | 0.601 | 0.32 | 0.679 |
| 88 | 0.697 | 0.478 | -0.219 | 0.564 | 0.362 | 0.818 |
| 89 | 1.16 | 0.441 | -0.72 | 0.59 | 0.339 | 0.738 |
| 90 | 1.07 | 30.304 | 29.234 | 0 | 0.997 | 427.797 |
| 91 | 1.693 | 8.658 | 6.965 | 0.051 | 0.918 | 16.032 |
| 92 | 0.92 | 13.001 | 12.081 | 0.005 | 0.982 | 78.04 |
| 93 | 1.148 | 0.397 | -0.751 | 0.601 | 0.327 | 0.699 |
| 94 | 0.754 | 0.451 | -0.303 | 0.573 | 0.354 | 0.788 |
| 95 | 0.58 | 0.375 | -0.205 | 0.579 | 0.344 | 0.756 |
| 96 | 1.151 | 0.406 | -0.745 | 0.598 | 0.33 | 0.708 |
| 97 | 0.738 | 0.345 | -0.393 | 0.608 | 0.316 | 0.665 |
| 98 | 1.789 | 31.451 | 29.662 | 0.004 | 0.985 | 96.358 |
| 99 | 1.215 | 0.386 | -0.83 | 0.607 | 0.321 | 0.681 |
| 100 | 0.741 | 0.408 | -0.333 | 0.583 | 0.343 | 0.753 |
| 101 | 0.741 | 0.408 | -0.333 | 0.583 | 0.343 | 0.753 |
| 102 | 0.865 | 0.362 | -0.503 | 0.606 | 0.32 | 0.677 |
| 103 | 0.697 | 0.478 | -0.219 | 0.564 | 0.362 | 0.818 |
| 104 | 1.127 | 0.349 | -0.779 | 0.621 | 0.306 | 0.634 |
| 105 | 1.129 | 0.352 | -0.777 | 0.619 | 0.308 | 0.639 |
| 106 | 1.139 | 0.374 | -0.765 | 0.609 | 0.318 | 0.671 |
| 107 | 0.738 | 0.345 | -0.393 | 0.608 | 0.316 | 0.665 |
| 108 | 0.7 | 0.368 | -0.332 | 0.594 | 0.33 | 0.71 |
| 109 | 1.228 | 0.42 | -0.808 | 0.596 | 0.332 | 0.716 |
| 110 | 0.58 | 0.375 | -0.205 | 0.579 | 0.344 | 0.756 |
| 111 | 1.228 | 0.42 | -0.808 | 0.596 | 0.332 | 0.716 |
| 112 | 0.576 | 0.364 | -0.212 | 0.583 | 0.34 | 0.741 |
| 113 | 0.578 | 0.39 | -0.188 | 0.572 | 0.351 | 0.777 |
| 114 | 0.695 | 0.345 | -0.351 | 0.606 | 0.318 | 0.672 |
| 115 | 0.697 | 0.478 | -0.219 | 0.564 | 0.362 | 0.818 |
| 116 | 1.097 | 23.923 | 22.825 | 0.002 | 0.992 | 178.941 |
| 117 | 0.741 | 0.408 | -0.333 | 0.583 | 0.343 | 0.753 |
| 118 | 0.883 | 0.404 | -0.479 | 0.591 | 0.336 | 0.729 |
| 119 | 0.669 | 3.732 | 3.064 | 0.122 | 0.84 | 7.558 |
| 120 | 0.754 | 0.451 | -0.303 | 0.573 | 0.354 | 0.788 |
| 121 | 0.494 | 0.346 | -0.148 | 0.573 | 0.346 | 0.762 |
| 122 | 0.88 | 8.476 | 7.596 | 0.062 | 0.912 | 14.914 |
| 123 | 1.699 | 15.01 | 13.311 | 0.018 | 0.961 | 35.336 |
| 124 | 0.743 | 0.366 | -0.377 | 0.598 | 0.327 | 0.698 |
| 125 | 1.151 | 0.406 | -0.745 | 0.598 | 0.33 | 0.708 |
| 126 | 0.696 | 0.35 | -0.346 | 0.603 | 0.321 | 0.681 |
| 127 | 0.733 | 0.326 | -0.407 | 0.619 | 0.304 | 0.63 |
| 128 | 1.74 | 14.423 | 12.683 | 0.022 | 0.955 | 30.469 |
| 129 | 0.695 | 0.341 | -0.354 | 0.608 | 0.316 | 0.665 |
| 130 | 1.21 | 5.585 | 4.375 | 0.067 | 0.897 | 12.578 |
| 131 | 0.743 | 0.366 | -0.377 | 0.598 | 0.327 | 0.698 |
| 132 | 0.58 | 0.375 | -0.205 | 0.579 | 0.344 | 0.756 |
| 133 | 0.697 | 0.478 | -0.219 | 0.564 | 0.362 | 0.818 |
| 134 | 1.088 | 19.137 | 18.049 | 0.003 | 0.988 | 117.818 |
| 135 | 1.139 | 0.374 | -0.765 | 0.609 | 0.318 | 0.671 |
| 136 | 0.738 | 0.345 | -0.393 | 0.608 | 0.316 | 0.665 |
| 137 | 0.472 | 0.364 | -0.108 | 0.555 | 0.363 | 0.821 |
| 138 | 0.697 | 0.478 | -0.219 | 0.564 | 0.362 | 0.818 |
| 139 | 1.208 | 0.37 | -0.838 | 0.613 | 0.315 | 0.661 |
| 140 | 0.745 | 0.377 | -0.367 | 0.593 | 0.332 | 0.715 |
| 141 | 0.865 | 0.362 | -0.503 | 0.606 | 0.32 | 0.677 |
| 142 | 1.228 | 0.42 | -0.808 | 0.596 | 0.332 | 0.716 |
| 143 | 1.127 | 0.349 | -0.779 | 0.621 | 0.306 | 0.634 |
| 144 | 0.697 | 0.478 | -0.219 | 0.564 | 0.362 | 0.818 |
| 145 | 1.151 | 0.406 | -0.745 | 0.598 | 0.33 | 0.708 |
| 146 | 0.578 | 0.352 | -0.226 | 0.589 | 0.333 | 0.718 |
| 147 | 0.617 | 0.344 | -0.273 | 0.599 | 0.324 | 0.689 |
| 148 | 0.522 | 0.387 | -0.135 | 0.562 | 0.359 | 0.807 |
| 149 | 1.151 | 0.406 | -0.745 | 0.598 | 0.33 | 0.708 |
| 150 | 0.865 | 0.362 | -0.503 | 0.606 | 0.32 | 0.677 |
| 151 | 0.576 | 0.364 | -0.212 | 0.583 | 0.34 | 0.741 |
| 152 | 1.151 | 0.406 | -0.745 | 0.598 | 0.33 | 0.708 |
| 153 | 0.695 | 0.345 | -0.351 | 0.606 | 0.318 | 0.672 |
| 154 | 1.417 | 1.236 | -0.18 | 0.347 | 0.595 | 2.117 |
| 155 | 1.208 | 0.37 | -0.838 | 0.613 | 0.315 | 0.661 |
| 156 | 0.697 | 0.478 | -0.219 | 0.564 | 0.362 | 0.818 |
| 157 | 0.701 | 0.377 | -0.324 | 0.591 | 0.334 | 0.722 |
| 158 | 0.701 | 0.377 | -0.324 | 0.591 | 0.334 | 0.722 |
| 159 | 0.883 | 0.404 | -0.479 | 0.591 | 0.336 | 0.729 |
| 160 | 0.697 | 0.478 | -0.219 | 0.564 | 0.362 | 0.818 |
| 161 | 0.743 | 0.366 | -0.377 | 0.598 | 0.327 | 0.698 |
| 162 | 1.139 | 0.374 | -0.765 | 0.609 | 0.318 | 0.671 |
| 163 | 0.739 | 0.347 | -0.392 | 0.607 | 0.317 | 0.668 |
| 164 | 0.741 | 0.408 | -0.333 | 0.583 | 0.343 | 0.753 |
| 165 | 0.733 | 0.326 | -0.407 | 0.619 | 0.304 | 0.63 |
| 166 | 1.581 | 3.016 | 1.434 | 0.188 | 0.764 | 4.653 |
| 167 | 1.129 | 0.352 | -0.777 | 0.619 | 0.308 | 0.639 |
| 168 | 0.741 | 0.408 | -0.333 | 0.583 | 0.343 | 0.753 |
| 169 | 1.208 | 0.37 | -0.838 | 0.613 | 0.315 | 0.661 |
| 170 | 1.381 | 0.973 | -0.408 | 0.368 | 0.573 | 1.935 |
| 171 | 0.754 | 0.451 | -0.303 | 0.573 | 0.354 | 0.788 |
| 172 | 0.697 | 0.478 | -0.219 | 0.564 | 0.362 | 0.818 |
| 173 | 1.543 | 1.422 | -0.121 | 0.34 | 0.604 | 2.194 |
| 174 | 1.215 | 0.386 | -0.83 | 0.607 | 0.321 | 0.681 |
| 175 | 0.754 | 0.451 | -0.303 | 0.573 | 0.354 | 0.788 |
| 176 | 0.865 | 0.362 | -0.503 | 0.606 | 0.32 | 0.677 |
| 177 | 1.228 | 0.42 | -0.808 | 0.596 | 0.332 | 0.716 |
| 178 | 0.697 | 0.478 | -0.219 | 0.564 | 0.362 | 0.818 |
| 179 | 1.717 | 14.062 | 12.345 | 0.022 | 0.956 | 31.219 |
| 180 | 1.139 | 0.374 | -0.765 | 0.609 | 0.318 | 0.671 |
| 181 | 0.898 | 10.72 | 9.822 | 0.007 | 0.978 | 64.543 |
| 182 | 0.695 | 0.341 | -0.354 | 0.608 | 0.316 | 0.665 |
| 183 | 0.701 | 0.377 | -0.324 | 0.591 | 0.334 | 0.722 |
| 184 | 1.228 | 0.42 | -0.808 | 0.596 | 0.332 | 0.716 |
| 185 | 0.883 | 0.404 | -0.479 | 0.591 | 0.336 | 0.729 |
| 186 | 0.695 | 0.345 | -0.351 | 0.606 | 0.318 | 0.672 |
| 187 | 0.701 | 0.377 | -0.324 | 0.591 | 0.334 | 0.722 |
| 188 | 1.148 | 0.397 | -0.751 | 0.601 | 0.327 | 0.699 |
| 189 | 0.695 | 0.341 | -0.354 | 0.608 | 0.316 | 0.665 |
| 190 | 0.741 | 0.408 | -0.333 | 0.583 | 0.343 | 0.753 |
| 191 | 0.739 | 0.347 | -0.392 | 0.607 | 0.317 | 0.668 |
| 192 | 1.208 | 0.37 | -0.838 | 0.613 | 0.315 | 0.661 |
| 193 | 0.695 | 0.341 | -0.354 | 0.608 | 0.316 | 0.665 |
| 194 | 0.741 | 0.408 | -0.333 | 0.583 | 0.343 | 0.753 |
| 195 | 1.151 | 0.406 | -0.745 | 0.598 | 0.33 | 0.708 |
| 196 | 0.745 | 0.377 | -0.367 | 0.593 | 0.332 | 0.715 |
| 197 | 0.695 | 0.345 | -0.351 | 0.606 | 0.318 | 0.672 |
| 198 | 0.58 | 0.369 | -0.211 | 0.581 | 0.342 | 0.747 |
| 199 | 0.58 | 0.375 | -0.205 | 0.579 | 0.344 | 0.756 |
| 200 | 0.865 | 0.362 | -0.503 | 0.606 | 0.32 | 0.677 |
| 201 | 1.148 | 0.397 | -0.751 | 0.601 | 0.327 | 0.699 |
| 202 | 0.696 | 0.35 | -0.346 | 0.603 | 0.321 | 0.681 |
| 203 | 0.856 | 1.012 | 0.156 | 0.341 | 0.6 | 2.156 |
| 204 | 0.695 | 0.341 | -0.354 | 0.608 | 0.316 | 0.665 |
| 205 | 1.135 | 0.364 | -0.771 | 0.613 | 0.314 | 0.658 |
| 206 | 1.127 | 0.349 | -0.779 | 0.621 | 0.306 | 0.634 |
| 207 | 0.733 | 0.326 | -0.407 | 0.619 | 0.304 | 0.63 |
| 208 | 0.754 | 0.451 | -0.303 | 0.573 | 0.354 | 0.788 |
| 209 | 1.139 | 0.374 | -0.765 | 0.609 | 0.318 | 0.671 |
| 210 | 0.695 | 0.341 | -0.354 | 0.608 | 0.316 | 0.665 |
| 211 | 1.228 | 0.42 | -0.808 | 0.596 | 0.332 | 0.716 |
| 212 | 1.135 | 0.364 | -0.771 | 0.613 | 0.314 | 0.658 |
| 213 | 0.58 | 0.375 | -0.205 | 0.579 | 0.344 | 0.756 |
| 214 | 0.528 | 0.417 | -0.111 | 0.554 | 0.368 | 0.839 |
| 215 | 1.16 | 0.441 | -0.72 | 0.59 | 0.339 | 0.738 |
| 216 | 0.695 | 0.345 | -0.351 | 0.606 | 0.318 | 0.672 |
| 217 | 1.139 | 0.374 | -0.765 | 0.609 | 0.318 | 0.671 |
| 218 | 0.7 | 0.368 | -0.332 | 0.594 | 0.33 | 0.71 |
| 219 | 0.915 | 1.873 | 0.958 | 0.207 | 0.741 | 4.121 |
| 220 | 1.967 | 0.382 | -1.585 | 0.623 | 0.307 | 0.639 |
| 221 | 1.489 | 1.07 | -0.419 | 0.362 | 0.58 | 1.985 |
| 222 | 1.151 | 0.406 | -0.745 | 0.598 | 0.33 | 0.708 |
| 223 | 0.745 | 0.377 | -0.367 | 0.593 | 0.332 | 0.715 |
| 224 | 1.228 | 0.42 | -0.808 | 0.596 | 0.332 | 0.716 |
| 225 | 1.151 | 0.406 | -0.745 | 0.598 | 0.33 | 0.708 |
| 226 | 0.701 | 0.377 | -0.324 | 0.591 | 0.334 | 0.722 |
| 227 | 0.525 | 0.387 | -0.138 | 0.563 | 0.359 | 0.806 |
| 228 | 0.848 | 0.831 | -0.017 | 0.361 | 0.578 | 1.972 |
| 229 | 0.492 | 0.322 | -0.17 | 0.587 | 0.331 | 0.711 |
| 230 | 1.161 | 0.399 | -0.762 | 0.601 | 0.327 | 0.7 |
| 231 | 1.936 | 0.352 | -1.584 | 0.635 | 0.294 | 0.599 |
| 232 | 0.583 | 0.393 | -0.19 | 0.572 | 0.351 | 0.779 |
| 233 | 0.703 | 0.347 | -0.355 | 0.605 | 0.319 | 0.675 |
| 234 | 0.701 | 0.378 | -0.323 | 0.59 | 0.334 | 0.723 |
| 235 | 1.148 | 0.366 | -0.781 | 0.613 | 0.314 | 0.66 |
| 236 | 0.701 | 0.379 | -0.323 | 0.59 | 0.335 | 0.724 |
| 237 | 1.216 | 0.387 | -0.829 | 0.606 | 0.322 | 0.683 |
| 238 | 0.745 | 0.377 | -0.367 | 0.593 | 0.332 | 0.715 |
| 239 | 1.215 | 0.386 | -0.83 | 0.607 | 0.321 | 0.681 |
| 240 | 1.228 | 0.42 | -0.808 | 0.596 | 0.332 | 0.716 |
| 241 | 0.743 | 0.366 | -0.377 | 0.598 | 0.327 | 0.698 |
| 242 | 0.738 | 0.345 | -0.393 | 0.608 | 0.316 | 0.665 |
| 243 | 1.38 | 0.842 | -0.538 | 0.383 | 0.557 | 1.814 |
| 244 | 1.135 | 0.364 | -0.771 | 0.613 | 0.314 | 0.658 |
| 245 | 0.695 | 0.341 | -0.354 | 0.608 | 0.316 | 0.665 |
| 246 | 0.701 | 0.377 | -0.324 | 0.591 | 0.334 | 0.722 |
| 247 | 1.967 | 0.382 | -1.585 | 0.623 | 0.307 | 0.639 |
| 248 | 0.582 | 0.352 | -0.23 | 0.59 | 0.333 | 0.717 |
| 249 | 1.215 | 0.386 | -0.83 | 0.607 | 0.321 | 0.681 |
| 250 | 1.967 | 0.382 | -1.585 | 0.623 | 0.307 | 0.639 |
| 251 | 0.7 | 0.368 | -0.332 | 0.594 | 0.33 | 0.71 |
| 252 | 0.734 | 0.327 | -0.406 | 0.618 | 0.305 | 0.633 |
| 253 | 1.135 | 0.364 | -0.771 | 0.613 | 0.314 | 0.658 |
| 254 | 1.129 | 0.352 | -0.777 | 0.619 | 0.308 | 0.639 |
| 255 | 0.745 | 0.377 | -0.367 | 0.593 | 0.332 | 0.715 |
| 256 | 0.699 | 0.365 | -0.334 | 0.596 | 0.329 | 0.705 |
| 257 | 1.135 | 0.364 | -0.771 | 0.613 | 0.314 | 0.658 |
| 258 | 0.695 | 0.341 | -0.354 | 0.608 | 0.316 | 0.665 |
| 259 | 1.228 | 0.42 | -0.808 | 0.596 | 0.332 | 0.716 |
| 260 | 0.695 | 0.341 | -0.354 | 0.608 | 0.316 | 0.665 |
| 261 | 0.495 | 0.361 | -0.134 | 0.565 | 0.354 | 0.79 |
| 262 | 0.549 | 0.342 | -0.207 | 0.589 | 0.332 | 0.715 |
| 263 | 0.754 | 0.451 | -0.303 | 0.573 | 0.354 | 0.788 |
| 264 | 0.865 | 0.362 | -0.503 | 0.606 | 0.32 | 0.677 |
| 265 | 1.228 | 0.42 | -0.808 | 0.596 | 0.332 | 0.716 |
| 266 | 0.865 | 0.362 | -0.503 | 0.606 | 0.32 | 0.677 |
| 267 | 1.215 | 0.386 | -0.83 | 0.607 | 0.321 | 0.681 |
| 268 | 0.522 | 0.387 | -0.135 | 0.562 | 0.359 | 0.807 |
| 269 | 0.801 | 2.32 | 1.519 | 0.269 | 0.679 | 3.041 |
| 270 | 0.581 | 0.332 | -0.25 | 0.601 | 0.32 | 0.678 |
| 271 | 1.215 | 0.386 | -0.83 | 0.607 | 0.321 | 0.681 |
| 272 | 0.739 | 0.347 | -0.392 | 0.607 | 0.317 | 0.668 |
| 273 | 1.135 | 0.364 | -0.771 | 0.613 | 0.314 | 0.658 |
| 274 | 0.743 | 0.366 | -0.377 | 0.598 | 0.327 | 0.698 |
| 275 | 1.129 | 0.352 | -0.777 | 0.619 | 0.308 | 0.639 |
| 276 | 1.215 | 0.386 | -0.83 | 0.607 | 0.321 | 0.681 |
| 277 | 1.103 | 21.635 | 20.532 | 0.003 | 0.989 | 133.957 |
| 278 | 1.721 | 8.724 | 7.003 | 0.04 | 0.93 | 19.099 |
| 279 | 1.215 | 0.386 | -0.83 | 0.607 | 0.321 | 0.681 |
| 280 | 1.47 | 0.94 | -0.53 | 0.374 | 0.567 | 1.887 |
| 281 | 1.135 | 0.364 | -0.771 | 0.613 | 0.314 | 0.658 |
| 282 | 1.215 | 0.386 | -0.83 | 0.607 | 0.321 | 0.681 |
| 283 | 1.215 | 0.386 | -0.83 | 0.607 | 0.321 | 0.681 |
| 284 | 1.215 | 0.386 | -0.83 | 0.607 | 0.321 | 0.681 |
| 285 | 1.022 | 0.9 | -0.121 | 0.362 | 0.578 | 1.974 |
| 286 | 1.135 | 0.364 | -0.771 | 0.613 | 0.314 | 0.658 |
| 287 | 0.783 | 0.72 | -0.063 | 0.376 | 0.562 | 1.847 |
| 288 | 0.733 | 0.326 | -0.407 | 0.619 | 0.304 | 0.63 |
| 289 | 1.228 | 0.42 | -0.808 | 0.596 | 0.332 | 0.716 |
| 290 | 0.591 | 6.313 | 5.722 | 0.012 | 0.973 | 52.504 |
| 291 | 1.215 | 0.386 | -0.83 | 0.607 | 0.321 | 0.681 |
| 292 | 0.733 | 0.326 | -0.407 | 0.619 | 0.304 | 0.63 |
| 293 | 0.697 | 0.478 | -0.219 | 0.564 | 0.362 | 0.818 |
| 294 | 1.139 | 0.374 | -0.765 | 0.609 | 0.318 | 0.671 |
| 295 | 0.701 | 0.377 | -0.324 | 0.591 | 0.334 | 0.722 |
| 296 | 0.733 | 0.326 | -0.407 | 0.619 | 0.304 | 0.63 |
| 297 | 1.956 | 21.581 | 19.625 | 0.013 | 0.968 | 43.914 |
| 298 | 1.526 | 1.063 | -0.464 | 0.364 | 0.578 | 1.971 |
| 299 | 0.495 | 0.322 | -0.173 | 0.588 | 0.33 | 0.709 |
| 300 | 1.215 | 0.386 | -0.83 | 0.607 | 0.321 | 0.681 |
| 301 | 1.139 | 0.374 | -0.765 | 0.609 | 0.318 | 0.671 |
| 302 | 1.139 | 0.374 | -0.765 | 0.609 | 0.318 | 0.671 |
| 303 | 1.835 | 6.159 | 4.324 | 0.08 | 0.882 | 10.811 |
| 304 | 0.698 | 0.328 | -0.37 | 0.616 | 0.308 | 0.64 |
| 305 | 0.876 | 0.365 | -0.511 | 0.605 | 0.321 | 0.68 |
| 306 | 0.734 | 0.329 | -0.406 | 0.618 | 0.306 | 0.635 |
| 307 | 0.865 | 0.362 | -0.503 | 0.606 | 0.32 | 0.677 |
| 308 | 0.739 | 0.349 | -0.39 | 0.607 | 0.318 | 0.671 |
| 309 | 1.139 | 0.374 | -0.765 | 0.609 | 0.318 | 0.671 |
| 310 | 1.151 | 0.406 | -0.745 | 0.598 | 0.33 | 0.708 |
| 311 | 1.396 | 3.651 | 2.256 | 0.162 | 0.792 | 5.492 |
| 312 | 0.9 | 13.454 | 12.554 | 0.004 | 0.985 | 93.354 |
| 313 | 0.532 | 1.022 | 0.49 | 0.296 | 0.646 | 2.628 |
| 314 | 1.827 | 12.484 | 10.658 | 0.028 | 0.947 | 25.725 |
| 315 | 1.754 | 5.498 | 3.745 | 0.132 | 0.827 | 6.888 |
| 316 | 0.733 | 0.326 | -0.407 | 0.619 | 0.304 | 0.63 |
| 317 | 1.696 | 20.278 | 18.582 | 0.011 | 0.972 | 50.559 |
| 318 | 1.215 | 0.386 | -0.83 | 0.607 | 0.321 | 0.681 |
| 319 | 1.151 | 0.406 | -0.745 | 0.598 | 0.33 | 0.708 |
| 320 | 0.883 | 0.404 | -0.479 | 0.591 | 0.336 | 0.729 |
| 321 | 0.883 | 0.404 | -0.479 | 0.591 | 0.336 | 0.729 |
| 322 | 1.139 | 0.374 | -0.765 | 0.609 | 0.318 | 0.671 |
| 323 | 1.151 | 0.406 | -0.745 | 0.598 | 0.33 | 0.708 |
| 324 | 1.151 | 0.406 | -0.745 | 0.598 | 0.33 | 0.708 |
| 325 | 0.733 | 0.326 | -0.407 | 0.619 | 0.304 | 0.63 |
| 326 | 1.208 | 0.37 | -0.838 | 0.613 | 0.315 | 0.661 |
| 327 | 1.215 | 0.386 | -0.83 | 0.607 | 0.321 | 0.681 |
| 328 | 0.691 | 0.326 | -0.366 | 0.617 | 0.306 | 0.636 |
| 329 | 0.754 | 0.451 | -0.303 | 0.573 | 0.354 | 0.788 |
| 330 | 0.733 | 0.326 | -0.407 | 0.619 | 0.304 | 0.63 |
| 331 | 1.151 | 0.406 | -0.745 | 0.598 | 0.33 | 0.708 |
| 332 | 0.733 | 0.326 | -0.407 | 0.619 | 0.304 | 0.63 |
| 333 | 1.215 | 0.386 | -0.83 | 0.607 | 0.321 | 0.681 |
| 334 | 1.215 | 0.386 | -0.83 | 0.607 | 0.321 | 0.681 |
| 335 | 0.739 | 0.347 | -0.392 | 0.607 | 0.317 | 0.668 |
| 336 | 0.578 | 0.39 | -0.188 | 0.572 | 0.351 | 0.777 |
| 337 | 0.815 | 0.799 | -0.016 | 0.364 | 0.575 | 1.949 |
| 338 | 1.714 | 3.233 | 1.52 | 0.185 | 0.767 | 4.736 |
| 339 | 1.151 | 0.406 | -0.745 | 0.598 | 0.33 | 0.708 |
| 340 | 0.852 | 1.466 | 0.614 | 0.228 | 0.718 | 3.672 |
| 341 | 0.639 | 1.018 | 0.378 | 0.319 | 0.622 | 2.366 |
| 342 | 0.739 | 0.348 | -0.391 | 0.607 | 0.318 | 0.67 |
| 343 | 1.148 | 0.397 | -0.751 | 0.601 | 0.327 | 0.699 |
| 344 | 1.139 | 0.374 | -0.765 | 0.609 | 0.318 | 0.671 |
| 345 | 0.697 | 0.478 | -0.219 | 0.564 | 0.362 | 0.818 |
| 346 | 1.139 | 0.374 | -0.765 | 0.609 | 0.318 | 0.671 |
| 347 | 1.215 | 0.386 | -0.83 | 0.607 | 0.321 | 0.681 |
| 348 | 1.151 | 0.406 | -0.745 | 0.598 | 0.33 | 0.708 |
| 349 | 0.733 | 0.326 | -0.407 | 0.619 | 0.304 | 0.63 |
| 350 | 1.217 | 0.386 | -0.831 | 0.607 | 0.321 | 0.681 |
| 351 | 1.153 | 0.406 | -0.747 | 0.598 | 0.33 | 0.708 |
| 352 | 0.735 | 0.326 | -0.408 | 0.619 | 0.305 | 0.631 |
| 353 | 0.962 | 3.859 | 2.897 | 0.09 | 0.87 | 9.666 |
| 354 | 1.153 | 0.406 | -0.747 | 0.598 | 0.33 | 0.708 |
| 355 | 1.914 | 0.334 | -1.58 | 0.645 | 0.284 | 0.572 |
| 356 | 2.248 | 2.567 | 0.319 | 0.526 | 0.41 | 1 |
| 357 | 2.248 | 2.567 | 0.319 | 0.526 | 0.41 | 1 |
| 358 | 2.248 | 2.567 | 0.319 | 0.526 | 0.41 | 1 |
| 359 | 2.248 | 2.567 | 0.319 | 0.526 | 0.41 | 1 |
| 360 | 2.248 | 2.567 | 0.319 | 0.526 | 0.41 | 1 |
| 361 | 2.248 | 2.567 | 0.319 | 0.526 | 0.41 | 1 |
| 362 | 2.248 | 2.567 | 0.319 | 0.526 | 0.41 | 1 |
| 363 | 2.248 | 2.567 | 0.319 | 0.526 | 0.41 | 1 |
| 364 | 2.248 | 2.567 | 0.319 | 0.526 | 0.41 | 1 |
| 365 | 2.248 | 2.567 | 0.319 | 0.526 | 0.41 | 1 |
| 366 | 2.248 | 2.567 | 0.319 | 0.526 | 0.41 | 1 |
| 367 | 2.248 | 2.567 | 0.319 | 0.526 | 0.41 | 1 |
| 368 | 1.063 | 1.514 | 0.452 | 0.527 | 0.406 | 0.983 |
| 369 | 14.587 | 0.505 | -14.082 | 0.906 | 0.073 | 0.114 |
| 370 | 0.742 | 0.329 | -0.413 | 0.618 | 0.306 | 0.634 |
| 371 | 1.152 | 0.377 | -0.775 | 0.609 | 0.319 | 0.674 |
| 372 | 0.749 | 0.411 | -0.338 | 0.582 | 0.344 | 0.755 |
| 373 | 0.698 | 0.326 | -0.372 | 0.617 | 0.306 | 0.635 |
| 374 | 2.196 | 24.001 | 21.805 | 0.014 | 0.966 | 41.508 |
| 375 | 1.208 | 0.37 | -0.838 | 0.613 | 0.315 | 0.661 |
| 376 | 1.71 | 3.405 | 1.695 | 0.18 | 0.773 | 4.898 |
| 377 | 0.733 | 0.326 | -0.407 | 0.619 | 0.304 | 0.63 |
| 378 | 1.151 | 0.406 | -0.745 | 0.598 | 0.33 | 0.708 |
| 379 | 0.883 | 0.404 | -0.479 | 0.591 | 0.336 | 0.729 |
| 380 | 0.691 | 0.326 | -0.366 | 0.617 | 0.306 | 0.636 |
| 381 | 1.215 | 0.386 | -0.83 | 0.607 | 0.321 | 0.681 |
| 382 | 1.139 | 0.374 | -0.765 | 0.609 | 0.318 | 0.671 |
| 383 | 0.754 | 0.451 | -0.303 | 0.573 | 0.354 | 0.788 |
| 384 | 1.215 | 0.386 | -0.83 | 0.607 | 0.321 | 0.681 |
| 385 | 1.228 | 0.42 | -0.808 | 0.596 | 0.332 | 0.716 |
| 386 | 1.215 | 0.386 | -0.83 | 0.607 | 0.321 | 0.681 |
| 387 | 0.745 | 0.377 | -0.367 | 0.593 | 0.332 | 0.715 |
| 388 | 0.691 | 0.326 | -0.366 | 0.617 | 0.306 | 0.636 |
| 389 | 0.7 | 0.368 | -0.332 | 0.594 | 0.33 | 0.71 |
| 390 | 1.139 | 0.374 | -0.765 | 0.609 | 0.318 | 0.671 |
| 391 | 0.701 | 0.377 | -0.324 | 0.591 | 0.334 | 0.722 |
| 392 | 0.733 | 0.326 | -0.407 | 0.619 | 0.304 | 0.63 |
| 393 | 1.228 | 0.42 | -0.808 | 0.596 | 0.332 | 0.716 |
| 394 | 1.151 | 0.406 | -0.745 | 0.598 | 0.33 | 0.708 |
| 395 | 0.733 | 0.326 | -0.407 | 0.619 | 0.304 | 0.63 |
| 396 | 1.215 | 0.386 | -0.83 | 0.607 | 0.321 | 0.681 |
| 397 | 0.578 | 0.352 | -0.226 | 0.589 | 0.333 | 0.718 |
| 398 | 1.139 | 0.374 | -0.765 | 0.609 | 0.318 | 0.671 |
| 399 | 0.617 | 0.344 | -0.273 | 0.599 | 0.324 | 0.689 |
| 400 | 1.215 | 0.386 | -0.83 | 0.607 | 0.321 | 0.681 |
| 401 | 1.215 | 0.386 | -0.83 | 0.607 | 0.321 | 0.681 |
| 402 | 0.58 | 0.375 | -0.205 | 0.579 | 0.344 | 0.756 |
| 403 | 1.148 | 0.397 | -0.751 | 0.601 | 0.327 | 0.699 |
| 404 | 1.139 | 0.374 | -0.765 | 0.609 | 0.318 | 0.671 |
| 405 | 0.741 | 0.408 | -0.333 | 0.583 | 0.343 | 0.753 |
| 406 | 0.733 | 0.326 | -0.407 | 0.619 | 0.304 | 0.63 |
| 407 | 0.578 | 0.39 | -0.188 | 0.572 | 0.351 | 0.777 |
| 408 | 1.228 | 0.42 | -0.808 | 0.596 | 0.332 | 0.716 |
| 409 | 0.492 | 0.322 | -0.17 | 0.587 | 0.331 | 0.711 |
| 410 | 1.215 | 0.386 | -0.83 | 0.607 | 0.321 | 0.681 |
| 411 | 1.151 | 0.406 | -0.745 | 0.598 | 0.33 | 0.708 |
| 412 | 0.578 | 0.39 | -0.188 | 0.572 | 0.351 | 0.777 |
| 413 | 0.58 | 0.332 | -0.248 | 0.601 | 0.32 | 0.679 |
| 414 | 1.139 | 0.374 | -0.765 | 0.609 | 0.318 | 0.671 |
| 415 | 1.151 | 0.406 | -0.745 | 0.598 | 0.33 | 0.708 |
| 416 | 1.208 | 0.37 | -0.838 | 0.613 | 0.315 | 0.661 |
| 417 | 1.151 | 0.406 | -0.745 | 0.598 | 0.33 | 0.708 |
| 418 | 1.151 | 0.406 | -0.745 | 0.598 | 0.33 | 0.708 |
| 419 | 0.697 | 0.478 | -0.219 | 0.564 | 0.362 | 0.818 |
| 420 | 0.733 | 0.326 | -0.407 | 0.619 | 0.304 | 0.63 |
| 421 | 1.151 | 0.406 | -0.745 | 0.598 | 0.33 | 0.708 |
| 422 | 1.139 | 0.374 | -0.765 | 0.609 | 0.318 | 0.671 |
| 423 | 1.228 | 0.42 | -0.808 | 0.596 | 0.332 | 0.716 |
| 424 | 1.129 | 0.352 | -0.777 | 0.619 | 0.308 | 0.639 |
| 425 | 1.139 | 0.374 | -0.765 | 0.609 | 0.318 | 0.671 |
| 426 | 0.865 | 0.362 | -0.503 | 0.606 | 0.32 | 0.677 |
| 427 | 1.569 | 21.551 | 19.983 | 0.008 | 0.978 | 62.741 |
| 428 | 0.741 | 0.408 | -0.333 | 0.583 | 0.343 | 0.753 |
| 429 | 0.733 | 0.326 | -0.407 | 0.619 | 0.304 | 0.63 |
| 430 | 1.16 | 0.441 | -0.72 | 0.59 | 0.339 | 0.738 |
| 431 | 0.754 | 0.451 | -0.303 | 0.573 | 0.354 | 0.788 |
| 432 | 0.733 | 0.326 | -0.407 | 0.619 | 0.304 | 0.63 |
| 433 | 0.697 | 0.478 | -0.219 | 0.564 | 0.362 | 0.818 |
| 434 | 0.784 | 0.724 | -0.059 | 0.375 | 0.563 | 1.854 |
| 435 | 0.733 | 0.326 | -0.407 | 0.619 | 0.304 | 0.63 |
| 436 | 14.829 | 31.102 | 16.273 | 0.12 | 0.716 | 3.638 |
| 437 | 0.701 | 0.377 | -0.324 | 0.591 | 0.334 | 0.722 |
| 438 | 0.528 | 0.417 | -0.111 | 0.554 | 0.368 | 0.839 |
| 439 | 1.139 | 0.374 | -0.765 | 0.609 | 0.318 | 0.671 |
| 440 | 1.151 | 0.406 | -0.745 | 0.598 | 0.33 | 0.708 |
| 441 | 1.215 | 0.386 | -0.83 | 0.607 | 0.321 | 0.681 |
| 442 | 0.549 | 0.342 | -0.207 | 0.589 | 0.332 | 0.715 |
| 443 | 1.228 | 0.42 | -0.808 | 0.596 | 0.332 | 0.716 |
| 444 | 1.208 | 0.37 | -0.838 | 0.613 | 0.315 | 0.661 |
| 445 | 1.129 | 0.352 | -0.777 | 0.619 | 0.308 | 0.639 |
| 446 | 0.636 | 0.851 | 0.215 | 0.337 | 0.602 | 2.179 |
| 447 | 0.492 | 0.322 | -0.17 | 0.587 | 0.331 | 0.711 |
| 448 | 1.208 | 0.37 | -0.838 | 0.613 | 0.315 | 0.661 |
| 449 | 1.129 | 0.352 | -0.777 | 0.619 | 0.308 | 0.639 |
| 450 | 1.208 | 0.37 | -0.838 | 0.613 | 0.315 | 0.661 |
| 451 | 1.139 | 0.374 | -0.765 | 0.609 | 0.318 | 0.671 |
| 452 | 1.151 | 0.406 | -0.745 | 0.598 | 0.33 | 0.708 |
| 453 | 1.151 | 0.406 | -0.745 | 0.598 | 0.33 | 0.708 |
| 454 | 0.739 | 0.347 | -0.392 | 0.607 | 0.317 | 0.668 |
| 455 | 1.151 | 0.406 | -0.745 | 0.598 | 0.33 | 0.708 |
| 456 | 0.549 | 0.342 | -0.207 | 0.589 | 0.332 | 0.715 |
| 457 | 0.581 | 0.332 | -0.25 | 0.601 | 0.32 | 0.678 |
| 458 | 0.745 | 0.379 | -0.366 | 0.593 | 0.332 | 0.717 |
| 459 | 0.691 | 0.326 | -0.366 | 0.617 | 0.306 | 0.636 |
| 460 | 1.151 | 0.406 | -0.745 | 0.598 | 0.33 | 0.708 |
| 461 | 1.129 | 0.352 | -0.777 | 0.619 | 0.308 | 0.639 |
| 462 | 1.215 | 0.386 | -0.83 | 0.607 | 0.321 | 0.681 |
| 463 | 1.139 | 0.374 | -0.765 | 0.609 | 0.318 | 0.671 |
| 464 | 0.865 | 0.362 | -0.503 | 0.606 | 0.32 | 0.677 |
| 465 | 1.215 | 0.386 | -0.83 | 0.607 | 0.321 | 0.681 |
| 466 | 0.743 | 0.366 | -0.377 | 0.598 | 0.327 | 0.698 |
| 467 | 0.836 | 1.364 | 0.528 | 0.234 | 0.711 | 3.549 |
| 468 | 0.733 | 0.326 | -0.407 | 0.619 | 0.304 | 0.63 |
| 469 | 1.127 | 0.349 | -0.779 | 0.621 | 0.306 | 0.634 |
| 470 | 0.695 | 0.341 | -0.354 | 0.608 | 0.316 | 0.665 |
| 471 | 0.865 | 0.362 | -0.503 | 0.606 | 0.32 | 0.677 |
| 472 | 1.151 | 0.406 | -0.745 | 0.598 | 0.33 | 0.708 |
| 473 | 1.151 | 0.406 | -0.745 | 0.598 | 0.33 | 0.708 |
| 474 | 1.215 | 0.386 | -0.83 | 0.607 | 0.321 | 0.681 |
| 475 | 1.151 | 0.406 | -0.745 | 0.598 | 0.33 | 0.708 |
| 476 | 0.865 | 0.362 | -0.503 | 0.606 | 0.32 | 0.677 |
| 477 | 0.754 | 0.451 | -0.303 | 0.573 | 0.354 | 0.788 |
| 478 | 0.584 | 0.433 | -0.151 | 0.561 | 0.363 | 0.821 |
| 479 | 1.418 | 1.265 | -0.154 | 0.345 | 0.597 | 2.135 |
| 480 | 1.191 | 5.62 | 4.429 | 0.066 | 0.899 | 12.784 |
| 481 | 1.228 | 0.42 | -0.808 | 0.596 | 0.332 | 0.716 |
| 482 | 2.061 | 24.773 | 22.713 | 0.011 | 0.971 | 48.178 |
| 483 | 0.58 | 0.375 | -0.205 | 0.579 | 0.344 | 0.756 |
| 484 | 1.745 | 16.306 | 14.561 | 0.017 | 0.962 | 36.897 |
| 485 | 0.699 | 0.365 | -0.334 | 0.596 | 0.329 | 0.705 |
| 486 | 0.733 | 0.326 | -0.407 | 0.619 | 0.304 | 0.63 |
| 487 | 1.228 | 0.42 | -0.808 | 0.596 | 0.332 | 0.716 |
| 488 | 1.7 | 14.462 | 12.762 | 0.02 | 0.959 | 33.521 |
| 489 | 0.913 | 3.216 | 2.303 | 0.105 | 0.853 | 8.324 |
| 490 | 1.215 | 0.386 | -0.83 | 0.607 | 0.321 | 0.681 |
| 491 | 0.549 | 0.342 | -0.207 | 0.589 | 0.332 | 0.715 |
| 492 | 1.215 | 0.386 | -0.83 | 0.607 | 0.321 | 0.681 |
| 493 | 1.215 | 0.386 | -0.83 | 0.607 | 0.321 | 0.681 |
| 494 | 1.127 | 0.349 | -0.779 | 0.621 | 0.306 | 0.634 |
| 495 | 1.215 | 0.386 | -0.83 | 0.607 | 0.321 | 0.681 |
| 496 | 0.578 | 0.39 | -0.188 | 0.572 | 0.351 | 0.777 |
| 497 | 0.733 | 0.326 | -0.407 | 0.619 | 0.304 | 0.63 |
| 498 | 1.139 | 0.374 | -0.765 | 0.609 | 0.318 | 0.671 |
| 499 | 1.151 | 0.406 | -0.745 | 0.598 | 0.33 | 0.708 |
| 500 | 1.151 | 0.406 | -0.745 | 0.598 | 0.33 | 0.708 |
| 501 | 1.228 | 0.42 | -0.808 | 0.596 | 0.332 | 0.716 |
| 502 | 2.277 | 25.983 | 23.706 | 0.013 | 0.968 | 43.067 |
| 503 | 1.228 | 0.42 | -0.808 | 0.596 | 0.332 | 0.716 |
| 504 | 0.979 | 7.105 | 6.126 | 0.024 | 0.95 | 27.248 |
| 505 | 2.92 | 11.595 | 8.674 | 0.057 | 0.911 | 14.817 |
| SerineInsertion | 0.792 | 1.891 | 1.099 | 0.505 | 0.427 | 1.074 |
| 506 | 0.754 | 0.451 | -0.303 | 0.573 | 0.354 | 0.788 |
| 507 | 1.139 | 0.374 | -0.765 | 0.609 | 0.318 | 0.671 |
| 508 | 1.151 | 0.406 | -0.745 | 0.598 | 0.33 | 0.708 |
| 509 | 1.741 | 9.341 | 7.599 | 0.048 | 0.922 | 16.969 |
| 510 | 14.968 | 0.549 | -14.419 | 0.904 | 0.076 | 0.118 |
| 511 | 1.228 | 0.42 | -0.808 | 0.596 | 0.332 | 0.716 |
| 512 | 0.741 | 0.408 | -0.333 | 0.583 | 0.343 | 0.753 |
| 513 | 0.738 | 0.345 | -0.393 | 0.608 | 0.316 | 0.665 |
| 514 | 0.695 | 0.341 | -0.354 | 0.608 | 0.316 | 0.665 |
| 515 | 0.739 | 0.347 | -0.392 | 0.607 | 0.317 | 0.668 |
| 516 | 0.883 | 0.404 | -0.479 | 0.591 | 0.336 | 0.729 |
| 517 | 0.695 | 0.345 | -0.351 | 0.606 | 0.318 | 0.672 |
| 518 | 0.739 | 0.347 | -0.392 | 0.607 | 0.317 | 0.668 |
| 519 | 0.739 | 0.347 | -0.392 | 0.607 | 0.317 | 0.668 |
| 520 | 0.697 | 0.478 | -0.219 | 0.564 | 0.362 | 0.818 |
| 521 | 0.695 | 0.345 | -0.351 | 0.606 | 0.318 | 0.672 |
| 522 | 0.697 | 0.478 | -0.219 | 0.564 | 0.362 | 0.818 |
| 523 | 0.494 | 0.346 | -0.148 | 0.573 | 0.346 | 0.762 |
| 524 | 0.739 | 0.346 | -0.392 | 0.608 | 0.317 | 0.667 |
| 525 | 1.135 | 0.364 | -0.771 | 0.613 | 0.314 | 0.658 |
| 526 | 0.695 | 0.345 | -0.351 | 0.606 | 0.318 | 0.672 |
| 527 | 0.739 | 0.347 | -0.392 | 0.607 | 0.317 | 0.668 |
| 528 | 0.696 | 0.35 | -0.346 | 0.603 | 0.321 | 0.681 |
| 529 | 0.696 | 0.346 | -0.35 | 0.605 | 0.319 | 0.674 |
| 530 | 1.16 | 0.441 | -0.72 | 0.59 | 0.339 | 0.738 |
| 531 | 1.24 | 0.422 | -0.818 | 0.596 | 0.332 | 0.717 |
| 532 | 0.584 | 0.372 | -0.212 | 0.581 | 0.342 | 0.749 |
| 533 | 0.753 | 0.38 | -0.373 | 0.593 | 0.333 | 0.717 |
| 534 | 0.753 | 0.38 | -0.373 | 0.593 | 0.333 | 0.717 |
| 535 | 0.742 | 0.329 | -0.413 | 0.618 | 0.306 | 0.634 |
| 536 | 0.747 | 0.35 | -0.397 | 0.606 | 0.318 | 0.672 |
| 537 | 0.747 | 0.348 | -0.399 | 0.607 | 0.317 | 0.668 |
| 538 | 0.747 | 0.348 | -0.399 | 0.607 | 0.317 | 0.668 |
| 539 | 0.751 | 0.369 | -0.382 | 0.598 | 0.328 | 0.701 |
| 540 | 0.495 | 0.324 | -0.171 | 0.586 | 0.332 | 0.714 |
| 541 | 1.66 | 6.139 | 4.479 | 0.118 | 0.842 | 7.69 |
| 542 | 0.55 | 1.909 | 1.359 | 0.165 | 0.79 | 5.41 |
| 543 | 0.747 | 0.35 | -0.397 | 0.606 | 0.318 | 0.672 |
| 544 | 1.228 | 0.389 | -0.84 | 0.606 | 0.322 | 0.684 |
| 545 | 0.698 | 0.328 | -0.37 | 0.616 | 0.308 | 0.64 |
| 546 | 0.747 | 0.348 | -0.399 | 0.607 | 0.317 | 0.668 |
| 547 | 0.698 | 0.328 | -0.37 | 0.616 | 0.308 | 0.64 |
| 548 | 1.161 | 0.401 | -0.761 | 0.6 | 0.328 | 0.702 |
| 549 | 1.152 | 0.377 | -0.775 | 0.609 | 0.319 | 0.674 |
| 550 | 1.228 | 0.389 | -0.84 | 0.606 | 0.322 | 0.684 |
| 551 | 0.743 | 0.367 | -0.376 | 0.598 | 0.327 | 0.7 |
| 552 | 0.697 | 0.478 | -0.219 | 0.564 | 0.362 | 0.818 |
| 553 | 0.695 | 0.341 | -0.354 | 0.608 | 0.316 | 0.665 |
| 554 | 1.215 | 0.386 | -0.83 | 0.607 | 0.321 | 0.681 |
| 555 | 1.215 | 0.386 | -0.83 | 0.607 | 0.321 | 0.681 |
| 556 | 1.139 | 0.374 | -0.765 | 0.609 | 0.318 | 0.671 |
| 557 | 1.228 | 0.42 | -0.808 | 0.596 | 0.332 | 0.716 |
| 558 | 1.139 | 0.374 | -0.765 | 0.609 | 0.318 | 0.671 |
| 559 | 0.528 | 0.417 | -0.111 | 0.554 | 0.368 | 0.839 |
| 560 | 0.582 | 0.352 | -0.23 | 0.59 | 0.333 | 0.717 |
| 561 | 1.216 | 0.387 | -0.829 | 0.606 | 0.322 | 0.683 |
| 562 | 0.691 | 0.326 | -0.366 | 0.617 | 0.306 | 0.636 |
| 563 | 1.215 | 0.386 | -0.83 | 0.607 | 0.321 | 0.681 |
| 564 | 1.208 | 0.37 | -0.838 | 0.613 | 0.315 | 0.661 |
| 565 | 0.58 | 0.369 | -0.211 | 0.581 | 0.342 | 0.747 |
| 566 | 0.865 | 0.362 | -0.503 | 0.606 | 0.32 | 0.677 |
| 567 | 0.697 | 0.478 | -0.219 | 0.564 | 0.362 | 0.818 |
| 568 | 0.691 | 0.326 | -0.366 | 0.617 | 0.306 | 0.636 |
| 569 | 1.215 | 0.386 | -0.83 | 0.607 | 0.321 | 0.681 |
| 570 | 1.215 | 0.386 | -0.83 | 0.607 | 0.321 | 0.681 |
| 571 | 0.58 | 0.375 | -0.205 | 0.579 | 0.344 | 0.756 |
| 572 | 0.576 | 0.364 | -0.212 | 0.583 | 0.34 | 0.741 |
| 573 | 1.967 | 0.382 | -1.585 | 0.623 | 0.307 | 0.639 |
| 574 | 1.151 | 0.406 | -0.745 | 0.598 | 0.33 | 0.708 |
| PF3D7_1346800 | | | | | |  |
| Codon | **alpha** | **beta** | **beta-alpha** | **Posterior Prob Positive Selection** | **Posterior Prob Negative Selection** | **Empirical Bayes Factor** |
| 1 | 0.980099 | 0.535565 | -0.44453 | 0.434077 | 0.515253 | 0.876626 |
| 2 | 0.936952 | 0.617372 | -0.31958 | 0.44495 | 0.504289 | 0.916187 |
| 3 | 0.980099 | 0.535565 | -0.44453 | 0.434077 | 0.515253 | 0.876626 |
| 4 | 0.835937 | 0.633048 | -0.20289 | 0.454062 | 0.494798 | 0.950555 |
| 5 | 0.92519 | 0.622991 | -0.3022 | 0.446335 | 0.502865 | 0.921337 |
| 6 | 0.980099 | 0.535565 | -0.44453 | 0.434077 | 0.515253 | 0.876626 |
| 7 | 0.624482 | 0.574231 | -0.05025 | 0.465959 | 0.481799 | 0.997192 |
| 8 | 0.756063 | 0.497653 | -0.25841 | 0.447307 | 0.500828 | 0.92497 |
| 9 | 0.804696 | 0.607913 | -0.19678 | 0.454195 | 0.494476 | 0.951064 |
| 10 | 0.849233 | 0.599697 | -0.24954 | 0.449962 | 0.498879 | 0.934949 |
| 11 | 0.936553 | 0.564173 | -0.37238 | 0.440023 | 0.509139 | 0.898071 |
| 12 | 0.849233 | 0.599697 | -0.24954 | 0.449962 | 0.498879 | 0.934949 |
| 13 | 0.849233 | 0.599697 | -0.24954 | 0.449962 | 0.498879 | 0.934949 |
| 14 | 0.901672 | 0.637314 | -0.26436 | 0.449392 | 0.499737 | 0.932797 |
| 15 | 0.755969 | 0.481371 | -0.2746 | 0.445605 | 0.50248 | 0.918621 |
| 16 | 0.937081 | 0.634793 | -0.30229 | 0.446507 | 0.502757 | 0.921978 |
| 17 | 0.755969 | 0.481371 | -0.2746 | 0.445605 | 0.50248 | 0.918621 |
| 18 | 0.755969 | 0.481371 | -0.2746 | 0.445605 | 0.50248 | 0.918621 |
| 19 | 0.803733 | 0.452771 | -0.35096 | 0.438792 | 0.509475 | 0.893592 |
| 20 | 0.980944 | 0.640751 | -0.34019 | 0.443796 | 0.505647 | 0.911914 |
| 21 | 0.835832 | 0.616266 | -0.21957 | 0.452532 | 0.49629 | 0.944703 |
| 22 | 0.964412 | 0.618489 | -0.34592 | 0.443001 | 0.506352 | 0.908981 |
| 23 | 0.964412 | 0.618489 | -0.34592 | 0.443001 | 0.506352 | 0.908981 |
| 24 | 0.936443 | 0.549733 | -0.38671 | 0.438657 | 0.510485 | 0.893103 |
| 25 | 0.804696 | 0.607913 | -0.19678 | 0.454195 | 0.494476 | 0.951064 |
| 26 | 0.936443 | 0.549733 | -0.38671 | 0.438657 | 0.510485 | 0.893103 |
| 27 | 0.995642 | 4.81929 | 3.82364 | 0.83613 | 0.118312 | 5.8315 |
| 28 | 0.734136 | 4.65415 | 3.92001 | 0.858413 | 0.103565 | 6.9291 |
| 29 | 0.901672 | 0.637314 | -0.26436 | 0.449392 | 0.499737 | 0.932797 |
| 30 | 0.756063 | 0.497653 | -0.25841 | 0.447307 | 0.500828 | 0.92497 |
| 31 | 0.835836 | 0.616993 | -0.21884 | 0.452599 | 0.496224 | 0.94496 |
| 32 | 0.835004 | 0.487675 | -0.34733 | 0.440093 | 0.50843 | 0.898325 |
| 33 | 0.963496 | 0.503109 | -0.46039 | 0.432065 | 0.517147 | 0.869473 |
| 34 | 0.756808 | 0.634029 | -0.12278 | 0.460398 | 0.488147 | 0.975136 |
| 35 | 0.835832 | 0.616266 | -0.21957 | 0.452532 | 0.49629 | 0.944703 |
| 36 | 0.936952 | 0.617372 | -0.31958 | 0.44495 | 0.504289 | 0.916187 |
| 37 | 0.936608 | 0.571463 | -0.36515 | 0.440713 | 0.50846 | 0.90059 |
| 38 | 0.936295 | 0.530465 | -0.40583 | 0.436783 | 0.51233 | 0.886331 |
| 39 | 0.937081 | 0.634793 | -0.30229 | 0.446507 | 0.502757 | 0.921978 |
| 40 | 0.936295 | 0.530465 | -0.40583 | 0.436783 | 0.51233 | 0.886331 |
| 41 | 0.936553 | 0.564173 | -0.37238 | 0.440023 | 0.509139 | 0.898071 |
| 42 | 0.58984 | 0.378788 | -0.21105 | 0.447798 | 0.498893 | 0.926809 |
| 43 | 0.679459 | 0.534057 | -0.1454 | 0.457293 | 0.490572 | 0.96302 |
| 44 | 0.803962 | 0.487579 | -0.31638 | 0.442485 | 0.505874 | 0.907083 |
| 45 | 0.936553 | 0.564173 | -0.37238 | 0.440023 | 0.509139 | 0.898071 |
| 46 | 0.804743 | 0.616137 | -0.18861 | 0.454953 | 0.493739 | 0.953978 |
| 47 | 0.884432 | 0.668717 | -0.21572 | 0.45349 | 0.495629 | 0.948362 |
| 48 | 0.834252 | 0.379268 | -0.45498 | 0.427925 | 0.52034 | 0.854909 |
| 49 | 0.803962 | 0.487579 | -0.31638 | 0.442485 | 0.505874 | 0.907083 |
| 50 | 0.835832 | 0.616266 | -0.21957 | 0.452532 | 0.49629 | 0.944703 |
| 51 | 0.804743 | 0.616137 | -0.18861 | 0.454953 | 0.493739 | 0.953978 |
| 52 | 5.83325 | 0.656321 | -5.17693 | 0.065094 | 0.90371 | 0.079575 |
| 53 | 0.835836 | 0.616993 | -0.21884 | 0.452599 | 0.496224 | 0.94496 |
| 54 | 0.803931 | 0.482594 | -0.32134 | 0.441964 | 0.506381 | 0.90517 |
| 55 | 1.01003 | 7.66218 | 6.65215 | 0.926395 | 0.030852 | 14.3845 |
| 56 | 0.849233 | 0.599697 | -0.24954 | 0.449962 | 0.498879 | 0.934949 |
| 57 | 0.756808 | 0.634029 | -0.12278 | 0.460398 | 0.488147 | 0.975136 |
| 58 | 0.964086 | 0.576662 | -0.38742 | 0.439167 | 0.510135 | 0.894954 |
| 59 | 0.964086 | 0.576662 | -0.38742 | 0.439167 | 0.510135 | 0.894954 |
| 60 | 0.804743 | 0.616137 | -0.18861 | 0.454953 | 0.493739 | 0.953978 |
| 61 | 0.835937 | 0.633048 | -0.20289 | 0.454062 | 0.494798 | 0.950555 |
| 62 | 0.936952 | 0.617372 | -0.31958 | 0.44495 | 0.504289 | 0.916187 |
| 63 | 0.803962 | 0.487579 | -0.31638 | 0.442485 | 0.505874 | 0.907083 |
| 64 | 0.849233 | 0.599697 | -0.24954 | 0.449962 | 0.498879 | 0.934949 |
| 65 | 0.756132 | 0.509832 | -0.2463 | 0.448544 | 0.499628 | 0.929609 |
| 66 | 0.756132 | 0.509832 | -0.2463 | 0.448544 | 0.499628 | 0.929609 |
| 67 | 0.803252 | 0.3792 | -0.42405 | 0.430282 | 0.517788 | 0.863175 |
| 68 | 0.866476 | 4.28018 | 3.4137 | 0.831818 | 0.125382 | 5.65269 |
| 69 | 0.828505 | 0.689985 | -0.13852 | 0.459688 | 0.489274 | 0.972353 |
| 70 | 0.804842 | 0.632913 | -0.17193 | 0.456487 | 0.492248 | 0.959895 |
| 71 | 0.756166 | 0.515985 | -0.24018 | 0.44917 | 0.499021 | 0.931962 |
| 72 | 0.935464 | 0.426232 | -0.50923 | 0.425945 | 0.523015 | 0.848017 |
| 73 | 0.849233 | 0.599697 | -0.24954 | 0.449962 | 0.498879 | 0.934949 |
| 74 | 0.835832 | 0.616266 | -0.21957 | 0.452532 | 0.49629 | 0.944703 |
| 75 | 0.901672 | 0.637314 | -0.26436 | 0.449392 | 0.499737 | 0.932797 |
| 76 | 0.849233 | 0.599697 | -0.24954 | 0.449962 | 0.498879 | 0.934949 |
| 77 | 0.936952 | 0.617372 | -0.31958 | 0.44495 | 0.504289 | 0.916187 |
| 78 | 0.803252 | 0.3792 | -0.42405 | 0.430282 | 0.517788 | 0.863175 |
| 79 | 0.884432 | 0.668717 | -0.21572 | 0.45349 | 0.495629 | 0.948362 |
| 80 | 0.964412 | 0.618489 | -0.34592 | 0.443001 | 0.506352 | 0.908981 |
| 81 | 0.936553 | 0.564173 | -0.37238 | 0.440023 | 0.509139 | 0.898071 |
| 82 | 0.936553 | 0.564173 | -0.37238 | 0.440023 | 0.509139 | 0.898071 |
| 83 | 0.835937 | 0.633048 | -0.20289 | 0.454062 | 0.494798 | 0.950555 |
| 84 | 0.936608 | 0.571463 | -0.36515 | 0.440713 | 0.50846 | 0.90059 |
| 85 | 0.936295 | 0.530465 | -0.40583 | 0.436783 | 0.51233 | 0.886331 |
| 86 | 0.936443 | 0.549733 | -0.38671 | 0.438657 | 0.510485 | 0.893103 |
| 87 | 0.936553 | 0.564173 | -0.37238 | 0.440023 | 0.509139 | 0.898071 |
| 88 | 0.849233 | 0.599697 | -0.24954 | 0.449962 | 0.498879 | 0.934949 |
| 89 | 0.964086 | 0.576662 | -0.38742 | 0.439167 | 0.510135 | 0.894954 |
| 90 | 0.849233 | 0.599697 | -0.24954 | 0.449962 | 0.498879 | 0.934949 |
| 91 | 0.835832 | 0.616266 | -0.21957 | 0.452532 | 0.49629 | 0.944703 |
| 92 | 0.803252 | 0.3792 | -0.42405 | 0.430282 | 0.517788 | 0.863175 |
| 93 | 0.803962 | 0.487579 | -0.31638 | 0.442485 | 0.505874 | 0.907083 |
| 94 | 0.756132 | 0.509832 | -0.2463 | 0.448544 | 0.499628 | 0.929609 |
| 95 | 0.936952 | 0.617372 | -0.31958 | 0.44495 | 0.504289 | 0.916187 |
| 96 | 0.936295 | 0.530465 | -0.40583 | 0.436783 | 0.51233 | 0.886331 |
| 97 | 0.964086 | 0.576662 | -0.38742 | 0.439167 | 0.510135 | 0.894954 |
| 98 | 0.803931 | 0.482594 | -0.32134 | 0.441964 | 0.506381 | 0.90517 |
| 99 | 0.935464 | 0.426232 | -0.50923 | 0.425945 | 0.523015 | 0.848017 |
| 100 | 0.901672 | 0.637314 | -0.26436 | 0.449392 | 0.499737 | 0.932797 |
| 101 | 0.937081 | 0.634793 | -0.30229 | 0.446507 | 0.502757 | 0.921978 |
| 102 | 0.803931 | 0.482594 | -0.32134 | 0.441964 | 0.506381 | 0.90517 |
| 103 | 0.803998 | 0.493274 | -0.31072 | 0.443077 | 0.505297 | 0.909262 |
| 104 | 0.937081 | 0.634793 | -0.30229 | 0.446507 | 0.502757 | 0.921978 |
| 105 | 0.964412 | 0.618489 | -0.34592 | 0.443001 | 0.506352 | 0.908981 |
| 106 | 0.936553 | 0.564173 | -0.37238 | 0.440023 | 0.509139 | 0.898071 |
| 107 | 0.936608 | 0.571463 | -0.36515 | 0.440713 | 0.50846 | 0.90059 |
| 108 | 0.59025 | 0.48199 | -0.10826 | 0.459695 | 0.487483 | 0.972379 |
| 109 | 0.756063 | 0.497653 | -0.25841 | 0.447307 | 0.500828 | 0.92497 |
| 110 | 0.835042 | 0.493367 | -0.34168 | 0.440683 | 0.507853 | 0.900479 |
| 111 | 0.937081 | 0.634793 | -0.30229 | 0.446507 | 0.502757 | 0.921978 |
| 112 | 0.935464 | 0.426232 | -0.50923 | 0.425945 | 0.523015 | 0.848017 |
| 113 | 0.901672 | 0.637314 | -0.26436 | 0.449392 | 0.499737 | 0.932797 |
| 114 | 0.964086 | 0.576662 | -0.38742 | 0.439167 | 0.510135 | 0.894954 |
| 115 | 0.964412 | 0.618489 | -0.34592 | 0.443001 | 0.506352 | 0.908981 |
| 116 | 0.901672 | 0.637314 | -0.26436 | 0.449392 | 0.499737 | 0.932797 |
| 117 | 0.936608 | 0.571463 | -0.36515 | 0.440713 | 0.50846 | 0.90059 |
| 118 | 0.624482 | 0.574231 | -0.05025 | 0.465959 | 0.481799 | 0.997192 |
| 119 | 0.936608 | 0.571463 | -0.36515 | 0.440713 | 0.50846 | 0.90059 |
| 120 | 0.964412 | 0.618489 | -0.34592 | 0.443001 | 0.506352 | 0.908981 |
| 121 | 0.884432 | 0.668717 | -0.21572 | 0.45349 | 0.495629 | 0.948362 |
| 122 | 0.937081 | 0.634793 | -0.30229 | 0.446507 | 0.502757 | 0.921978 |
| 123 | 0.835042 | 0.493367 | -0.34168 | 0.440683 | 0.507853 | 0.900479 |
| 124 | 0.849233 | 0.599697 | -0.24954 | 0.449962 | 0.498879 | 0.934949 |
| 125 | 0.936553 | 0.564173 | -0.37238 | 0.440023 | 0.509139 | 0.898071 |
| 126 | 0.835836 | 0.616993 | -0.21884 | 0.452599 | 0.496224 | 0.94496 |
| 127 | 0.838224 | 0.563594 | -0.27463 | 0.447394 | 0.501318 | 0.925295 |
| 128 | 0.755969 | 0.481371 | -0.2746 | 0.445605 | 0.50248 | 0.918621 |
| 129 | 0.936443 | 0.549733 | -0.38671 | 0.438657 | 0.510485 | 0.893103 |
| 130 | 0.936608 | 0.571463 | -0.36515 | 0.440713 | 0.50846 | 0.90059 |
| 131 | 0.755969 | 0.481371 | -0.2746 | 0.445605 | 0.50248 | 0.918621 |
| 132 | 0.937081 | 0.634793 | -0.30229 | 0.446507 | 0.502757 | 0.921978 |
| 133 | 0.901672 | 0.637314 | -0.26436 | 0.449392 | 0.499737 | 0.932797 |
| 134 | 0.964412 | 0.618489 | -0.34592 | 0.443001 | 0.506352 | 0.908981 |
| 135 | 0.804696 | 0.607913 | -0.19678 | 0.454195 | 0.494476 | 0.951064 |
| 136 | 0.901672 | 0.637314 | -0.26436 | 0.449392 | 0.499737 | 0.932797 |
| 137 | 0.804743 | 0.616137 | -0.18861 | 0.454953 | 0.493739 | 0.953978 |
| 138 | 0.849233 | 0.599697 | -0.24954 | 0.449962 | 0.498879 | 0.934949 |
| 139 | 0.803252 | 0.3792 | -0.42405 | 0.430282 | 0.517788 | 0.863175 |
| 140 | 0.936553 | 0.564173 | -0.37238 | 0.440023 | 0.509139 | 0.898071 |
| 141 | 0.964086 | 0.576662 | -0.38742 | 0.439167 | 0.510135 | 0.894954 |
| 142 | 0.937081 | 0.634793 | -0.30229 | 0.446507 | 0.502757 | 0.921978 |
| 143 | 0.964412 | 0.618489 | -0.34592 | 0.443001 | 0.506352 | 0.908981 |
| 144 | 0.936443 | 0.549733 | -0.38671 | 0.438657 | 0.510485 | 0.893103 |
| 145 | 0.935464 | 0.426232 | -0.50923 | 0.425945 | 0.523015 | 0.848017 |
| 146 | 0.964412 | 0.618489 | -0.34592 | 0.443001 | 0.506352 | 0.908981 |
| 147 | 0.803962 | 0.487579 | -0.31638 | 0.442485 | 0.505874 | 0.907083 |
| 148 | 0.849233 | 0.599697 | -0.24954 | 0.449962 | 0.498879 | 0.934949 |
| 149 | 0.937081 | 0.634793 | -0.30229 | 0.446507 | 0.502757 | 0.921978 |
| 150 | 0.936952 | 0.617372 | -0.31958 | 0.44495 | 0.504289 | 0.916187 |
| 151 | 0.92519 | 0.622991 | -0.3022 | 0.446335 | 0.502865 | 0.921337 |
| 152 | 0.936952 | 0.617372 | -0.31958 | 0.44495 | 0.504289 | 0.916187 |
| 153 | 0.936608 | 0.571463 | -0.36515 | 0.440713 | 0.50846 | 0.90059 |
| 154 | 0.936553 | 0.564173 | -0.37238 | 0.440023 | 0.509139 | 0.898071 |
| 155 | 0.936443 | 0.549733 | -0.38671 | 0.438657 | 0.510485 | 0.893103 |
| 156 | 0.964412 | 0.618489 | -0.34592 | 0.443001 | 0.506352 | 0.908981 |
| 157 | 0.837726 | 0.487681 | -0.35005 | 0.43987 | 0.508667 | 0.897514 |
| 158 | 0.964086 | 0.576662 | -0.38742 | 0.439167 | 0.510135 | 0.894954 |
| 159 | 0.964412 | 0.618489 | -0.34592 | 0.443001 | 0.506352 | 0.908981 |
| 160 | 0.936553 | 0.564173 | -0.37238 | 0.440023 | 0.509139 | 0.898071 |
| 161 | 0.624482 | 0.574231 | -0.05025 | 0.465959 | 0.481799 | 0.997192 |
| 162 | 0.834252 | 0.379268 | -0.45498 | 0.427925 | 0.52034 | 0.854909 |
| 163 | 0.835937 | 0.633048 | -0.20289 | 0.454062 | 0.494798 | 0.950555 |
| 164 | 0.963496 | 0.503109 | -0.46039 | 0.432065 | 0.517147 | 0.869473 |
| 165 | 0.936608 | 0.571463 | -0.36515 | 0.440713 | 0.50846 | 0.90059 |
| 166 | 0.964412 | 0.618489 | -0.34592 | 0.443001 | 0.506352 | 0.908981 |
| 167 | 0.849233 | 0.599697 | -0.24954 | 0.449962 | 0.498879 | 0.934949 |
| 168 | 0.901672 | 0.637314 | -0.26436 | 0.449392 | 0.499737 | 0.932797 |
| 169 | 0.964412 | 0.618489 | -0.34592 | 0.443001 | 0.506352 | 0.908981 |
| 170 | 0.835937 | 0.633048 | -0.20289 | 0.454062 | 0.494798 | 0.950555 |
| 171 | 0.964412 | 0.618489 | -0.34592 | 0.443001 | 0.506352 | 0.908981 |
| 172 | 6.02462 | 0.666764 | -5.35786 | 0.06177 | 0.907564 | 0.075244 |
| 173 | 0.835937 | 0.633048 | -0.20289 | 0.454062 | 0.494798 | 0.950555 |
| 174 | 0.901672 | 0.637314 | -0.26436 | 0.449392 | 0.499737 | 0.932797 |
| 175 | 0.835832 | 0.616266 | -0.21957 | 0.452532 | 0.49629 | 0.944703 |
| 176 | 0.964412 | 0.618489 | -0.34592 | 0.443001 | 0.506352 | 0.908981 |
| 177 | 0.849233 | 0.599697 | -0.24954 | 0.449962 | 0.498879 | 0.934949 |
| 178 | 0.831969 | 5.00138 | 4.16941 | 0.858875 | 0.10095 | 6.95557 |
| 179 | 0.901672 | 0.637314 | -0.26436 | 0.449392 | 0.499737 | 0.932797 |
| 180 | 0.92519 | 0.622991 | -0.3022 | 0.446335 | 0.502865 | 0.921337 |
| 181 | 0.756132 | 0.509832 | -0.2463 | 0.448544 | 0.499628 | 0.929609 |
| 182 | 0.963496 | 0.503109 | -0.46039 | 0.432065 | 0.517147 | 0.869473 |
| 183 | 0.937081 | 0.634793 | -0.30229 | 0.446507 | 0.502757 | 0.921978 |
| 184 | 0.936553 | 0.564173 | -0.37238 | 0.440023 | 0.509139 | 0.898071 |
| 185 | 0.936553 | 0.564173 | -0.37238 | 0.440023 | 0.509139 | 0.898071 |
| 186 | 0.882167 | 4.96171 | 4.07954 | 0.852418 | 0.105721 | 6.60124 |
| 187 | 0.849233 | 0.599697 | -0.24954 | 0.449962 | 0.498879 | 0.934949 |
| 188 | 0.997708 | 4.82137 | 3.82366 | 0.835984 | 0.1184 | 5.82527 |
| 189 | 0.901672 | 0.637314 | -0.26436 | 0.449392 | 0.499737 | 0.932797 |
| 190 | 0.964086 | 0.576662 | -0.38742 | 0.439167 | 0.510135 | 0.894954 |
| 191 | 0.964412 | 0.618489 | -0.34592 | 0.443001 | 0.506352 | 0.908981 |
| 192 | 0.835004 | 0.487675 | -0.34733 | 0.440093 | 0.50843 | 0.898325 |
| 193 | 0.964412 | 0.618489 | -0.34592 | 0.443001 | 0.506352 | 0.908981 |
| 194 | 0.955316 | 3.79144 | 2.83613 | 0.804844 | 0.149296 | 4.7134 |
| 195 | 0.804696 | 0.607913 | -0.19678 | 0.454195 | 0.494476 | 0.951064 |
| 196 | 0.849233 | 0.599697 | -0.24954 | 0.449962 | 0.498879 | 0.934949 |
| 197 | 0.935464 | 0.426232 | -0.50923 | 0.425945 | 0.523015 | 0.848017 |
| 198 | 0.936553 | 0.564173 | -0.37238 | 0.440023 | 0.509139 | 0.898071 |
| 199 | 0.884432 | 0.668717 | -0.21572 | 0.45349 | 0.495629 | 0.948362 |
| 200 | 0.884432 | 0.668717 | -0.21572 | 0.45349 | 0.495629 | 0.948362 |
| 201 | 0.936608 | 0.571463 | -0.36515 | 0.440713 | 0.50846 | 0.90059 |
| 202 | 0.835004 | 0.487675 | -0.34733 | 0.440093 | 0.50843 | 0.898325 |
| 203 | 0.937081 | 0.634793 | -0.30229 | 0.446507 | 0.502757 | 0.921978 |
| 204 | 0.884432 | 0.668717 | -0.21572 | 0.45349 | 0.495629 | 0.948362 |
| 205 | 0.937081 | 0.634793 | -0.30229 | 0.446507 | 0.502757 | 0.921978 |
| 206 | 0.936608 | 0.571463 | -0.36515 | 0.440713 | 0.50846 | 0.90059 |
| 207 | 0.849233 | 0.599697 | -0.24954 | 0.449962 | 0.498879 | 0.934949 |
| 208 | 0.964412 | 0.618489 | -0.34592 | 0.443001 | 0.506352 | 0.908981 |
| 209 | 0.901672 | 0.637314 | -0.26436 | 0.449392 | 0.499737 | 0.932797 |
| 210 | 0.964412 | 0.618489 | -0.34592 | 0.443001 | 0.506352 | 0.908981 |
| 211 | 0.964086 | 0.576662 | -0.38742 | 0.439167 | 0.510135 | 0.894954 |
| 212 | 0.936443 | 0.549733 | -0.38671 | 0.438657 | 0.510485 | 0.893103 |
| 213 | 0.936608 | 0.571463 | -0.36515 | 0.440713 | 0.50846 | 0.90059 |
| 214 | 0.849233 | 0.599697 | -0.24954 | 0.449962 | 0.498879 | 0.934949 |
| 215 | 0.803733 | 0.452771 | -0.35096 | 0.438792 | 0.509475 | 0.893592 |
| 216 | 0.980099 | 0.535565 | -0.44453 | 0.434077 | 0.515253 | 0.876626 |
| 217 | 0.936295 | 0.530465 | -0.40583 | 0.436783 | 0.51233 | 0.886331 |
| 218 | 0.937081 | 0.634793 | -0.30229 | 0.446507 | 0.502757 | 0.921978 |
| 219 | 1.01231 | 4.58833 | 3.57603 | 0.826833 | 0.126879 | 5.45704 |
| 220 | 0.964412 | 0.618489 | -0.34592 | 0.443001 | 0.506352 | 0.908981 |
| 221 | 0.964086 | 0.576662 | -0.38742 | 0.439167 | 0.510135 | 0.894954 |
| 222 | 0.964086 | 0.576662 | -0.38742 | 0.439167 | 0.510135 | 0.894954 |
| 223 | 0.803962 | 0.487579 | -0.31638 | 0.442485 | 0.505874 | 0.907083 |
| 224 | 0.854323 | 4.96964 | 4.11532 | 0.855567 | 0.103472 | 6.77005 |
| 225 | 0.835832 | 0.616266 | -0.21957 | 0.452532 | 0.49629 | 0.944703 |
| 226 | 0.964086 | 0.576662 | -0.38742 | 0.439167 | 0.510135 | 0.894954 |
| 227 | 0.803931 | 0.482594 | -0.32134 | 0.441964 | 0.506381 | 0.90517 |
| 228 | 0.835937 | 0.633048 | -0.20289 | 0.454062 | 0.494798 | 0.950555 |
| 229 | 0.936553 | 0.564173 | -0.37238 | 0.440023 | 0.509139 | 0.898071 |
| 230 | 0.936952 | 0.617372 | -0.31958 | 0.44495 | 0.504289 | 0.916187 |
| 231 | 0.964086 | 0.576662 | -0.38742 | 0.439167 | 0.510135 | 0.894954 |
| 232 | 0.964086 | 0.576662 | -0.38742 | 0.439167 | 0.510135 | 0.894954 |
| 233 | 0.849233 | 0.599697 | -0.24954 | 0.449962 | 0.498879 | 0.934949 |
| 234 | 0.901672 | 0.637314 | -0.26436 | 0.449392 | 0.499737 | 0.932797 |
| 235 | 0.936553 | 0.564173 | -0.37238 | 0.440023 | 0.509139 | 0.898071 |
| 236 | 0.830552 | 4.64104 | 3.81049 | 0.847661 | 0.111367 | 6.35938 |
| 237 | 0.964412 | 0.618489 | -0.34592 | 0.443001 | 0.506352 | 0.908981 |
| 238 | 0.849233 | 0.599697 | -0.24954 | 0.449962 | 0.498879 | 0.934949 |
| 239 | 0.936553 | 0.564173 | -0.37238 | 0.440023 | 0.509139 | 0.898071 |
| 240 | 0.734136 | 4.65415 | 3.92001 | 0.858413 | 0.103565 | 6.9291 |
| 241 | 0.901672 | 0.637314 | -0.26436 | 0.449392 | 0.499737 | 0.932797 |
| 242 | 0.873939 | 5.04947 | 4.17553 | 0.856 | 0.102577 | 6.79385 |
| 243 | 0.936553 | 0.564173 | -0.37238 | 0.440023 | 0.509139 | 0.898071 |
| 244 | 0.936553 | 0.564173 | -0.37238 | 0.440023 | 0.509139 | 0.898071 |
| 245 | 0.936553 | 0.564173 | -0.37238 | 0.440023 | 0.509139 | 0.898071 |
| 246 | 0.838558 | 0.616277 | -0.22228 | 0.452307 | 0.496526 | 0.943845 |
| 247 | 0.831714 | 4.93316 | 4.10145 | 0.856798 | 0.102877 | 6.83811 |
| 248 | 0.93013 | 7.80327 | 6.87314 | 0.935594 | 0.025858 | 16.6021 |
| 249 | 0.964412 | 0.618489 | -0.34592 | 0.443001 | 0.506352 | 0.908981 |
| 250 | 0.980099 | 0.535565 | -0.44453 | 0.434077 | 0.515253 | 0.876626 |
| 251 | 0.803252 | 0.3792 | -0.42405 | 0.430282 | 0.517788 | 0.863175 |
| 252 | 0.59025 | 0.48199 | -0.10826 | 0.459695 | 0.487483 | 0.972379 |
| 253 | 0.849233 | 0.599697 | -0.24954 | 0.449962 | 0.498879 | 0.934949 |
| 254 | 0.936295 | 0.530465 | -0.40583 | 0.436783 | 0.51233 | 0.886331 |
| 255 | 0.849233 | 0.599697 | -0.24954 | 0.449962 | 0.498879 | 0.934949 |
| 256 | 0.936553 | 0.564173 | -0.37238 | 0.440023 | 0.509139 | 0.898071 |
| 257 | 0.936553 | 0.564173 | -0.37238 | 0.440023 | 0.509139 | 0.898071 |
| 258 | 0.804696 | 0.607913 | -0.19678 | 0.454195 | 0.494476 | 0.951064 |
| 259 | 0.936553 | 0.564173 | -0.37238 | 0.440023 | 0.509139 | 0.898071 |
| 260 | 0.936952 | 0.617372 | -0.31958 | 0.44495 | 0.504289 | 0.916187 |
| 261 | 0.980099 | 0.535565 | -0.44453 | 0.434077 | 0.515253 | 0.876626 |
| 262 | 0.834676 | 4.04782 | 3.21315 | 0.827203 | 0.130309 | 5.47118 |
| 263 | 0.835836 | 0.616993 | -0.21884 | 0.452599 | 0.496224 | 0.94496 |
| 264 | 0.803931 | 0.482594 | -0.32134 | 0.441964 | 0.506381 | 0.90517 |
| 265 | 0.963496 | 0.503109 | -0.46039 | 0.432065 | 0.517147 | 0.869473 |
| 266 | 0.936608 | 0.571463 | -0.36515 | 0.440713 | 0.50846 | 0.90059 |
| 267 | 0.963496 | 0.503109 | -0.46039 | 0.432065 | 0.517147 | 0.869473 |
| 268 | 0.936553 | 0.564173 | -0.37238 | 0.440023 | 0.509139 | 0.898071 |
| 269 | 0.936553 | 0.564173 | -0.37238 | 0.440023 | 0.509139 | 0.898071 |
| 270 | 0.997059 | 4.81584 | 3.81878 | 0.835871 | 0.118525 | 5.8205 |
| 271 | 0.964412 | 0.618489 | -0.34592 | 0.443001 | 0.506352 | 0.908981 |
| 272 | 0.972177 | 9.75481 | 8.78263 | 0.955766 | 0.004905 | 24.6944 |
| 273 | 0.937081 | 0.634793 | -0.30229 | 0.446507 | 0.502757 | 0.921978 |
| 274 | 0.849233 | 0.599697 | -0.24954 | 0.449962 | 0.498879 | 0.934949 |
| 275 | 0.936553 | 0.564173 | -0.37238 | 0.440023 | 0.509139 | 0.898071 |
| 276 | 0.67743 | 0.53405 | -0.14338 | 0.457477 | 0.490378 | 0.963733 |
| 277 | 0.964412 | 0.618489 | -0.34592 | 0.443001 | 0.506352 | 0.908981 |
| 278 | 0.803998 | 0.493274 | -0.31072 | 0.443077 | 0.505297 | 0.909262 |
| 279 | 0.756166 | 0.515985 | -0.24018 | 0.44917 | 0.499021 | 0.931962 |
| 280 | 0.964412 | 0.618489 | -0.34592 | 0.443001 | 0.506352 | 0.908981 |
| 281 | 0.835004 | 0.487675 | -0.34733 | 0.440093 | 0.50843 | 0.898325 |
| 282 | 0.964412 | 0.618489 | -0.34592 | 0.443001 | 0.506352 | 0.908981 |
| 283 | 0.935464 | 0.426232 | -0.50923 | 0.425945 | 0.523015 | 0.848017 |
| 284 | 0.849233 | 0.599697 | -0.24954 | 0.449962 | 0.498879 | 0.934949 |
| 285 | 0.936608 | 0.571463 | -0.36515 | 0.440713 | 0.50846 | 0.90059 |
| 286 | 0.838663 | 0.633056 | -0.20561 | 0.453837 | 0.495034 | 0.949691 |
| 287 | 0.936952 | 0.617372 | -0.31958 | 0.44495 | 0.504289 | 0.916187 |
| 288 | 0.936608 | 0.571463 | -0.36515 | 0.440713 | 0.50846 | 0.90059 |
| 289 | 0.936295 | 0.530465 | -0.40583 | 0.436783 | 0.51233 | 0.886331 |
| 290 | 0.837726 | 0.487681 | -0.35005 | 0.43987 | 0.508667 | 0.897514 |
| 291 | 0.964412 | 0.618489 | -0.34592 | 0.443001 | 0.506352 | 0.908981 |
| 292 | 0.803252 | 0.3792 | -0.42405 | 0.430282 | 0.517788 | 0.863175 |
| 293 | 0.964412 | 0.618489 | -0.34592 | 0.443001 | 0.506352 | 0.908981 |
| 294 | 0.804842 | 0.632913 | -0.17193 | 0.456487 | 0.492248 | 0.959895 |
| 295 | 0.964412 | 0.618489 | -0.34592 | 0.443001 | 0.506352 | 0.908981 |
| 296 | 0.804842 | 0.632913 | -0.17193 | 0.456487 | 0.492248 | 0.959895 |
| 297 | 0.849233 | 0.599697 | -0.24954 | 0.449962 | 0.498879 | 0.934949 |
| 298 | 0.756808 | 0.634029 | -0.12278 | 0.460398 | 0.488147 | 0.975136 |
| 299 | 0.964412 | 0.618489 | -0.34592 | 0.443001 | 0.506352 | 0.908981 |
| 300 | 0.936553 | 0.564173 | -0.37238 | 0.440023 | 0.509139 | 0.898071 |
| 301 | 0.835937 | 0.633048 | -0.20289 | 0.454062 | 0.494798 | 0.950555 |
| 302 | 0.755969 | 0.481371 | -0.2746 | 0.445605 | 0.50248 | 0.918621 |
| 303 | 0.849233 | 0.599697 | -0.24954 | 0.449962 | 0.498879 | 0.934949 |
| 304 | 0.849233 | 0.599697 | -0.24954 | 0.449962 | 0.498879 | 0.934949 |
| 305 | 0.936553 | 0.564173 | -0.37238 | 0.440023 | 0.509139 | 0.898071 |
| 306 | 0.936608 | 0.571463 | -0.36515 | 0.440713 | 0.50846 | 0.90059 |
| 307 | 0.849233 | 0.599697 | -0.24954 | 0.449962 | 0.498879 | 0.934949 |
| 308 | 0.834252 | 0.379268 | -0.45498 | 0.427925 | 0.52034 | 0.854909 |
| 309 | 0.936553 | 0.564173 | -0.37238 | 0.440023 | 0.509139 | 0.898071 |
| 310 | 0.964086 | 0.576662 | -0.38742 | 0.439167 | 0.510135 | 0.894954 |
| 311 | 0.964086 | 0.576662 | -0.38742 | 0.439167 | 0.510135 | 0.894954 |
| 312 | 0.964086 | 0.576662 | -0.38742 | 0.439167 | 0.510135 | 0.894954 |
| 313 | 0.803799 | 0.462724 | -0.34108 | 0.439873 | 0.50842 | 0.897525 |
| 314 | 0.849233 | 0.599697 | -0.24954 | 0.449962 | 0.498879 | 0.934949 |
| 315 | 0.936952 | 0.617372 | -0.31958 | 0.44495 | 0.504289 | 0.916187 |
| 316 | 0.803962 | 0.487579 | -0.31638 | 0.442485 | 0.505874 | 0.907083 |
| 317 | 0.804743 | 0.616137 | -0.18861 | 0.454953 | 0.493739 | 0.953978 |
| 318 | 0.935464 | 0.426232 | -0.50923 | 0.425945 | 0.523015 | 0.848017 |
| 319 | 0.803733 | 0.452771 | -0.35096 | 0.438792 | 0.509475 | 0.893592 |
| 320 | 0.980944 | 0.640751 | -0.34019 | 0.443796 | 0.505647 | 0.911914 |
| 321 | 0.936553 | 0.564173 | -0.37238 | 0.440023 | 0.509139 | 0.898071 |
| 322 | 0.964412 | 0.618489 | -0.34592 | 0.443001 | 0.506352 | 0.908981 |
| 323 | 0.964412 | 0.618489 | -0.34592 | 0.443001 | 0.506352 | 0.908981 |
| 324 | 0.936553 | 0.564173 | -0.37238 | 0.440023 | 0.509139 | 0.898071 |
| 325 | 0.963496 | 0.503109 | -0.46039 | 0.432065 | 0.517147 | 0.869473 |
| 326 | 0.803962 | 0.487579 | -0.31638 | 0.442485 | 0.505874 | 0.907083 |
| 327 | 0.804696 | 0.607913 | -0.19678 | 0.454195 | 0.494476 | 0.951064 |
| 328 | 0.835042 | 0.493367 | -0.34168 | 0.440683 | 0.507853 | 0.900479 |
| 329 | 0.590794 | 0.632044 | 0.041251 | 0.474426 | 0.473433 | 1.03167 |
| 330 | 0.804696 | 0.607913 | -0.19678 | 0.454195 | 0.494476 | 0.951064 |
| 331 | 0.964412 | 0.618489 | -0.34592 | 0.443001 | 0.506352 | 0.908981 |
| 332 | 0.936952 | 0.617372 | -0.31958 | 0.44495 | 0.504289 | 0.916187 |
| 333 | 0.834252 | 0.379268 | -0.45498 | 0.427925 | 0.52034 | 0.854909 |
| 334 | 0.937081 | 0.634793 | -0.30229 | 0.446507 | 0.502757 | 0.921978 |
| 335 | 0.964412 | 0.618489 | -0.34592 | 0.443001 | 0.506352 | 0.908981 |
| 336 | 0.901672 | 0.637314 | -0.26436 | 0.449392 | 0.499737 | 0.932797 |
| 337 | 0.849233 | 0.599697 | -0.24954 | 0.449962 | 0.498879 | 0.934949 |
| 338 | 0.834252 | 0.379268 | -0.45498 | 0.427925 | 0.52034 | 0.854909 |
| 339 | 0.834252 | 0.379268 | -0.45498 | 0.427925 | 0.52034 | 0.854909 |
| 340 | 0.937081 | 0.634793 | -0.30229 | 0.446507 | 0.502757 | 0.921978 |
| 341 | 0.936952 | 0.617372 | -0.31958 | 0.44495 | 0.504289 | 0.916187 |
| 342 | 0.936295 | 0.530465 | -0.40583 | 0.436783 | 0.51233 | 0.886331 |
| 343 | 0.964412 | 0.618489 | -0.34592 | 0.443001 | 0.506352 | 0.908981 |
| 344 | 0.935464 | 0.426232 | -0.50923 | 0.425945 | 0.523015 | 0.848017 |
| 345 | 0.835832 | 0.616266 | -0.21957 | 0.452532 | 0.49629 | 0.944703 |
| 346 | 0.901672 | 0.637314 | -0.26436 | 0.449392 | 0.499737 | 0.932797 |
| 347 | 0.937081 | 0.634793 | -0.30229 | 0.446507 | 0.502757 | 0.921978 |
| 348 | 0.964086 | 0.576662 | -0.38742 | 0.439167 | 0.510135 | 0.894954 |
| 349 | 0.964412 | 0.618489 | -0.34592 | 0.443001 | 0.506352 | 0.908981 |
| 350 | 0.964086 | 0.576662 | -0.38742 | 0.439167 | 0.510135 | 0.894954 |
| 351 | 0.624482 | 0.574231 | -0.05025 | 0.465959 | 0.481799 | 0.997192 |
| 352 | 0.936608 | 0.571463 | -0.36515 | 0.440713 | 0.50846 | 0.90059 |
| 353 | 0.838224 | 0.563594 | -0.27463 | 0.447394 | 0.501318 | 0.925295 |
| 354 | 0.963496 | 0.503109 | -0.46039 | 0.432065 | 0.517147 | 0.869473 |
| 355 | 0.884432 | 0.668717 | -0.21572 | 0.45349 | 0.495629 | 0.948362 |
| 356 | 0.835004 | 0.487675 | -0.34733 | 0.440093 | 0.50843 | 0.898325 |
| 357 | 0.937081 | 0.634793 | -0.30229 | 0.446507 | 0.502757 | 0.921978 |
| 358 | 0.964412 | 0.618489 | -0.34592 | 0.443001 | 0.506352 | 0.908981 |
| 359 | 0.835004 | 0.487675 | -0.34733 | 0.440093 | 0.50843 | 0.898325 |
| 360 | 0.936295 | 0.530465 | -0.40583 | 0.436783 | 0.51233 | 0.886331 |
| 361 | 0.67743 | 0.53405 | -0.14338 | 0.457477 | 0.490378 | 0.963733 |
| 362 | 0.901672 | 0.637314 | -0.26436 | 0.449392 | 0.499737 | 0.932797 |
| 363 | 0.936443 | 0.549733 | -0.38671 | 0.438657 | 0.510485 | 0.893103 |
| 364 | 0.936608 | 0.571463 | -0.36515 | 0.440713 | 0.50846 | 0.90059 |
| 365 | 0.835832 | 0.616266 | -0.21957 | 0.452532 | 0.49629 | 0.944703 |
| 366 | 0.901672 | 0.637314 | -0.26436 | 0.449392 | 0.499737 | 0.932797 |
| 367 | 0.936608 | 0.571463 | -0.36515 | 0.440713 | 0.50846 | 0.90059 |
| 368 | 0.835004 | 0.487675 | -0.34733 | 0.440093 | 0.50843 | 0.898325 |
| 369 | 0.857749 | 6.6325 | 5.77475 | 0.926444 | 0.036663 | 14.3948 |
| 370 | 0.936553 | 0.564173 | -0.37238 | 0.440023 | 0.509139 | 0.898071 |
| 371 | 0.849233 | 0.599697 | -0.24954 | 0.449962 | 0.498879 | 0.934949 |
| 372 | 0.964086 | 0.576662 | -0.38742 | 0.439167 | 0.510135 | 0.894954 |
| 373 | 0.937081 | 0.634793 | -0.30229 | 0.446507 | 0.502757 | 0.921978 |
| 374 | 0.937081 | 0.634793 | -0.30229 | 0.446507 | 0.502757 | 0.921978 |
| 375 | 0.835937 | 0.633048 | -0.20289 | 0.454062 | 0.494798 | 0.950555 |
| 376 | 0.936553 | 0.564173 | -0.37238 | 0.440023 | 0.509139 | 0.898071 |
| 377 | 0.936553 | 0.564173 | -0.37238 | 0.440023 | 0.509139 | 0.898071 |
| 378 | 0.884432 | 0.668717 | -0.21572 | 0.45349 | 0.495629 | 0.948362 |
| 379 | 0.964086 | 0.576662 | -0.38742 | 0.439167 | 0.510135 | 0.894954 |
| 380 | 0.590794 | 0.632044 | 0.041251 | 0.474426 | 0.473433 | 1.03167 |
| 381 | 0.980099 | 0.535565 | -0.44453 | 0.434077 | 0.515253 | 0.876626 |
| 382 | 0.937081 | 0.634793 | -0.30229 | 0.446507 | 0.502757 | 0.921978 |
| 383 | 0.980099 | 0.535565 | -0.44453 | 0.434077 | 0.515253 | 0.876626 |
| 384 | 0.901672 | 0.637314 | -0.26436 | 0.449392 | 0.499737 | 0.932797 |
| 385 | 0.590298 | 0.49485 | -0.09545 | 0.461056 | 0.486182 | 0.97772 |
| 386 | 0.901672 | 0.637314 | -0.26436 | 0.449392 | 0.499737 | 0.932797 |
| 387 | 0.834252 | 0.379268 | -0.45498 | 0.427925 | 0.52034 | 0.854909 |
| 388 | 0.835004 | 0.487675 | -0.34733 | 0.440093 | 0.50843 | 0.898325 |
| 389 | 0.964412 | 0.618489 | -0.34592 | 0.443001 | 0.506352 | 0.908981 |
| 390 | 0.834252 | 0.379268 | -0.45498 | 0.427925 | 0.52034 | 0.854909 |
| 391 | 0.964086 | 0.576662 | -0.38742 | 0.439167 | 0.510135 | 0.894954 |
| 392 | 0.964412 | 0.618489 | -0.34592 | 0.443001 | 0.506352 | 0.908981 |
| 393 | 0.835004 | 0.487675 | -0.34733 | 0.440093 | 0.50843 | 0.898325 |
| 394 | 0.936553 | 0.564173 | -0.37238 | 0.440023 | 0.509139 | 0.898071 |
| 395 | 0.964412 | 0.618489 | -0.34592 | 0.443001 | 0.506352 | 0.908981 |
| 396 | 0.804696 | 0.607913 | -0.19678 | 0.454195 | 0.494476 | 0.951064 |
| 397 | 0.92519 | 0.622991 | -0.3022 | 0.446335 | 0.502865 | 0.921337 |
| 398 | 0.93698 | 0.621354 | -0.31563 | 0.445309 | 0.503935 | 0.917521 |
| 399 | 0.804696 | 0.607913 | -0.19678 | 0.454195 | 0.494476 | 0.951064 |
| 400 | 0.936952 | 0.617372 | -0.31958 | 0.44495 | 0.504289 | 0.916187 |
| 401 | 0.964086 | 0.576662 | -0.38742 | 0.439167 | 0.510135 | 0.894954 |
| 402 | 0.963535 | 0.507814 | -0.45572 | 0.432536 | 0.516682 | 0.871143 |
| 403 | 0.804887 | 0.640392 | -0.1645 | 0.457161 | 0.491593 | 0.962504 |
| 404 | 0.835958 | 0.636362 | -0.1996 | 0.45436 | 0.494507 | 0.9517 |
| 405 | 0.964412 | 0.618489 | -0.34592 | 0.443001 | 0.506352 | 0.908981 |
| 406 | 0.964412 | 0.618489 | -0.34592 | 0.443001 | 0.506352 | 0.908981 |
| 407 | 0.835105 | 0.502579 | -0.33253 | 0.441624 | 0.506934 | 0.903923 |
| 408 | 0.835852 | 0.61945 | -0.2164 | 0.452822 | 0.496007 | 0.94581 |
| 409 | 0.980119 | 0.537983 | -0.44214 | 0.43431 | 0.515022 | 0.87746 |
| 410 | 0.964086 | 0.576662 | -0.38742 | 0.439167 | 0.510135 | 0.894954 |
| 411 | 0.901672 | 0.637314 | -0.26436 | 0.449392 | 0.499737 | 0.932797 |
| 412 | 0.935512 | 0.432072 | -0.50344 | 0.426594 | 0.522374 | 0.850273 |
| 413 | 0.643299 | 0.576588 | -0.06671 | 0.464539 | 0.483317 | 0.991514 |
| 414 | 0.835057 | 0.495569 | -0.33949 | 0.440909 | 0.507633 | 0.901304 |
| 415 | 0.803978 | 0.489285 | -0.31469 | 0.44266 | 0.505703 | 0.907726 |
| 416 | 0.835052 | 0.494497 | -0.34056 | 0.440795 | 0.507743 | 0.90089 |
| 417 | 0.803978 | 0.489285 | -0.31469 | 0.44266 | 0.505703 | 0.907726 |
| 418 | 0.835004 | 0.487675 | -0.34733 | 0.440093 | 0.50843 | 0.898325 |
| 419 | 0.964412 | 0.618489 | -0.34592 | 0.443001 | 0.506352 | 0.908981 |
| 420 | 0.937081 | 0.634793 | -0.30229 | 0.446507 | 0.502757 | 0.921978 |
| 421 | 0.804696 | 0.607913 | -0.19678 | 0.454195 | 0.494476 | 0.951064 |
| 422 | 0.936467 | 0.553043 | -0.38343 | 0.438973 | 0.510173 | 0.894252 |
| 423 | 0.980119 | 0.537983 | -0.44214 | 0.43431 | 0.515022 | 0.87746 |
| 424 | 0.936295 | 0.530465 | -0.40583 | 0.436783 | 0.51233 | 0.886331 |
| 425 | 0.803777 | 0.459294 | -0.34448 | 0.439498 | 0.508786 | 0.896158 |
| 426 | 0.849233 | 0.599697 | -0.24954 | 0.449962 | 0.498879 | 0.934949 |
| 427 | 0.936295 | 0.530465 | -0.40583 | 0.436783 | 0.51233 | 0.886331 |
| 428 | 0.936295 | 0.530465 | -0.40583 | 0.436783 | 0.51233 | 0.886331 |
| 429 | 0.684502 | 0.539278 | -0.14522 | 0.457388 | 0.490521 | 0.963388 |
| 430 | 0.804696 | 0.607913 | -0.19678 | 0.454195 | 0.494476 | 0.951064 |
| 431 | 0.835852 | 0.61945 | -0.2164 | 0.452822 | 0.496007 | 0.94581 |
| 432 | 0.804696 | 0.607913 | -0.19678 | 0.454195 | 0.494476 | 0.951064 |
| 433 | 0.936295 | 0.530465 | -0.40583 | 0.436783 | 0.51233 | 0.886331 |
| 434 | 0.763697 | 0.634057 | -0.12964 | 0.459844 | 0.488729 | 0.972964 |
| 435 | 0.980119 | 0.537983 | -0.44214 | 0.43431 | 0.515022 | 0.87746 |
| 436 | 0.937081 | 0.634793 | -0.30229 | 0.446507 | 0.502757 | 0.921978 |
| 437 | 0.804762 | 0.619321 | -0.18544 | 0.455244 | 0.493456 | 0.955097 |
| 438 | 0.762979 | 0.504364 | -0.25862 | 0.447445 | 0.500746 | 0.925484 |
| 439 | 0.849233 | 0.599697 | -0.24954 | 0.449962 | 0.498879 | 0.934949 |
